# Transcriptomic atlas throughout *Coccidioides* development reveals key phase-enriched transcripts of this important fungal pathogen

**DOI:** 10.1371/journal.pbio.3003066

**Published:** 2025-04-15

**Authors:** Christina M. Homer, Mark Voorhies, Keith Walcott, Elena Ochoa, Anita Sil

**Affiliations:** 1 Division of Infectious Diseases, University of California San Francisco, San Francisco, California, United States of America; 2 Department of Microbiology and Immunology, University of California San Francisco, San Francisco, California, United States of America; 3 Chan Zuckerberg Biohub – San Francisco, San Francisco, California, United States of America; University of Georgia, United States of America

## Abstract

*Coccidioides spp*. are highly understudied but significant dimorphic fungal pathogens that can infect both immunocompetent and immunocompromised people. In the environment, they grow as multicellular filaments (hyphae) that produce vegetative spores called arthroconidia. Upon inhalation by mammals, arthroconidia undergo a process called spherulation. They enlarge and undergo numerous nuclear divisions to form a spherical structure, and then internally segment until the spherule is filled with multiple cells called endospores. Mature spherules rupture and release endospores, each of which can form another spherule, in a process thought to facilitate dissemination. Spherulation is unique to *Coccidioides*, and its molecular determinants remain largely unknown. Here, we report the first high-density transcriptomic analyses of *Coccidioides* development, defining morphology-dependent transcripts and those whose expression is regulated by *RYP1*, a major regulator required for spherulation and virulence. Of approximately 9,000 predicted transcripts, we discovered 273 transcripts with consistent spherule-associated expression, 82 of which are *RYP1*-dependent, a set likely to be critical for *Coccidioides* virulence. ChIP-Seq revealed two distinct regulons of Ryp1: one shared between hyphae and spherules and the other unique to spherules. Spherulation regulation was elaborate, with the majority of 227 predicted transcription factors in *Coccidioides* displaying spherule-enriched expression. We identified provocative targets, including 20 transcripts whose expression is endospore-enriched and 14 putative secreted effectors whose expression is spherule-enriched, of which six are secreted proteases. To highlight the utility of these data, we selected a cluster of *RYP1*-dependent, arthroconidia-associated transcripts and found that they play a role in arthroconidia cell wall biology, demonstrating the power of this resource in illuminating *Coccidioides* biology and virulence.

## Abstract 2

*Coccidioides* is a neglected fungal pathogen that forms a unique structure, the spherule, to cause disease. This study provides the first high-density transcriptomic analysis of *Coccidioides* development, showing spherule-specific gene regulation, Ryp1-dependent virulence factors, and potential targets of antifungal therapy.

## Introduction

*Coccidioides* spp. are dimorphic fungal pathogens found in the soil in the Southwest United States and other desert regions in Central and South America [1]. In the soil, they grow as hyphae that generate vegetative spores known as arthroconidia. Upon inhalation by a mammalian host, arthroconidia germinate and form a unique host-associated morphology known as the spherule [2]. Mature spherules rupture, releasing hundreds of internal cells known as endospores which can each go on to form another spherule in a cycle called spherulation. Notably, *Coccidioides* can cause infection in immunocompetent and immunocompromised individuals [3]. There is currently no cure for serious disseminated infections [4,5]. Efforts to develop new treatments and prevention strategies have been hindered by a lack of molecular knowledge of the host form of *Coccidioides,* the spherule, including sparse sampling of the transcriptome during *Coccidioides* development. Prior studies have relied on microarray or low replicate number RNA-Seq at one or two timepoints during spherule formation, in different conditions varying by laboratory, using different media to induce spherules versus hyphae, and only two published datasets profiled endospores after they have been released from spherules [6–12]. The spherule transcriptome remains under-characterized, and the endospore transcriptome is essentially unknown.

Despite limited molecular insight into spherulation, the Ryp1 transcription factor (TF) is known to be a major spherulation regulator [12]. Ryp1 is a WOPR-domain containing TF whose orthologs (such as Wor1) in other fungi are regulators of morphology transitions and development [13–16]. Additionally, WOPR family proteins often regulate virulence factors [15–17] and are required for virulence in fungal pathogens [12,18–21]. In *Coccidioides*, the *ryp1∆* mutant is unable to form spherules and has an aberrant transcriptome in both spherule and hyphal conditions [12].

Here, we performed the first high-depth, high-density transcriptomic time courses of *Coccidioides* arthroconidia germinating into either hyphae or spherules that went on to release endospores. We leveraged the *ryp1∆* mutant to define genes whose transcription is regulated by *RYP1* throughout these developmental trajectories, defined morphology-specific binding targets of *RYP1*, and highlighted a particular role for *RYP1* in direct regulation of genes expressed in the spherule morphology. Additionally, we annotated TFs, identified candidate secreted effectors, and defined candidate endospore-associated genes. From these data, we selected a cluster of spore-associated genes that were *RYP1*-dependent and found that they play a role in arthroconidia cell wall biology, demonstrating the power of this transcriptomic atlas to uncover new biology. Together, these findings serve as a foundational resource for the study of this important fungal pathogen.

## Results

### Transcriptomics of *Coccidioides* spherule development

**Fig 1:**
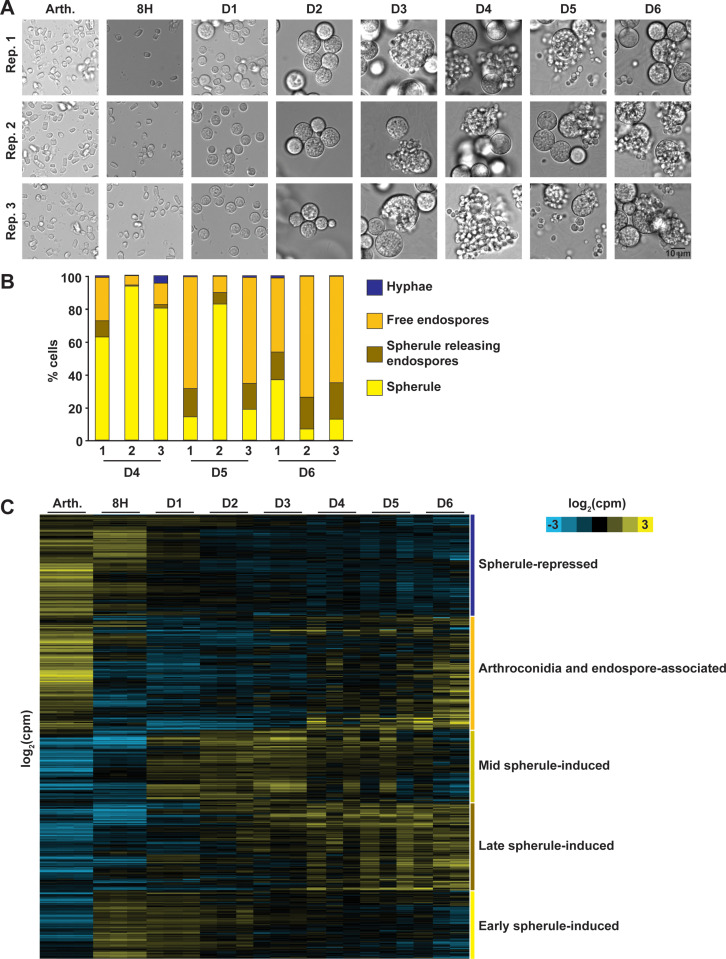
The transcriptome of arthroconidia germinating into spherules and releasing endospores. (A) Micrographs of fixed samples from each flask at the time of RNA harvest. Endospore release was first observed on day 3. (B) Quantification of the proportion of each morphology in cultures on days 4–6. *n* ≥  10 fields of view counted for each sample. Underlying data can be found in S2 Table. (C) Heatmap of transcript abundance as arthroconidia germinate into spherules and release endospores. Transcripts that had at least 10 reads detected in at least one sample were included as mean-centered rows in this heatmap. Rows are clustered using Cluster 3.0, k-means (*k*  =  5, 100 runs) using Spearman Rank Correlation similarity metric based on limma estimated counts across all datasets. Output clusters were then manually annotated. Log_2_(counts per million) indicated by yellow and blue shading.

Using the optimized spherulation conditions that we recently established [22], we germinated arthroconidia into spherules, observed morphology by light microscopy (S1A Fig), and isolated RNA for RNA-Seq at each timepoint from day 0 through day 6 (Fig 1A). We observed isotropic swelling in ~25% of arthroconidia in each replicate at 8 h (S1B Fig and S1 Table). Early spherules appeared by day 1 and continued to grow until day 3 when endospore release was first observed. On days 4–6, cultures developed into a complex mixture of maturing spherules, spherules releasing endospores, free endospores, and a small proportion of hyphae (Fig 1B, example morphology S1 Fig and S2 Table). Each replicate at each timepoint is from an independent culture, so there was some variation in the degree of endospore release in each flask (such as replicate 2, day 5). However, the overall trend is an increasing proportion of free endospores at later timepoints. As expected, spherule diameters were similar across replicates (S1C Fig and S3 Table). The transcriptome changed significantly over spherule development and as endospores were released, with 6,355 transcripts (of 8,628 total observed) undergoing at least a 2-fold change at 5% FDR at one or more timepoints throughout this experiment (Fig 1C and S4 Table). The arthroconidia stage exhibits a more divergent transcriptome than any other pairwise comparison throughout spherulation. The 8 h transcriptome demonstrates moderate correlation with the arthroconidia transcriptome and a similar degree of correlation with the day 1 transcriptome (both Pearson correlation of 0.5–0.6), consistent with a transition between the arthroconidia and spherule state (S1D Fig). Days 1–6 spherules are more correlated with each other than either the arthroconidia or 8 h timepoint, suggesting a consistent spherule signature. The number of transcripts that were significantly differential compared to the preceding timepoint decreased monotonically across spherulation, consistent with progression toward a more steady-state spherule transcriptome by the end of the experiment (S1E Fig). This is also reflected in principle component analysis demonstrating that days 4, 5, and 6 timepoints were not distinguishable from each other by whole transcriptome signature (S1F Fig). However, there is a group of transcripts that exhibit the interesting pattern of high abundance in arthroconidia and then returning to high abundance after endospores are released, suggesting these transcripts may accumulate in both spore forms (arthroconidia and endospores).

### Generating high-density transcriptomics of wildtype and mutant *Coccidioides* under spherule- and hyphal-inducing conditions

**Fig 2:**
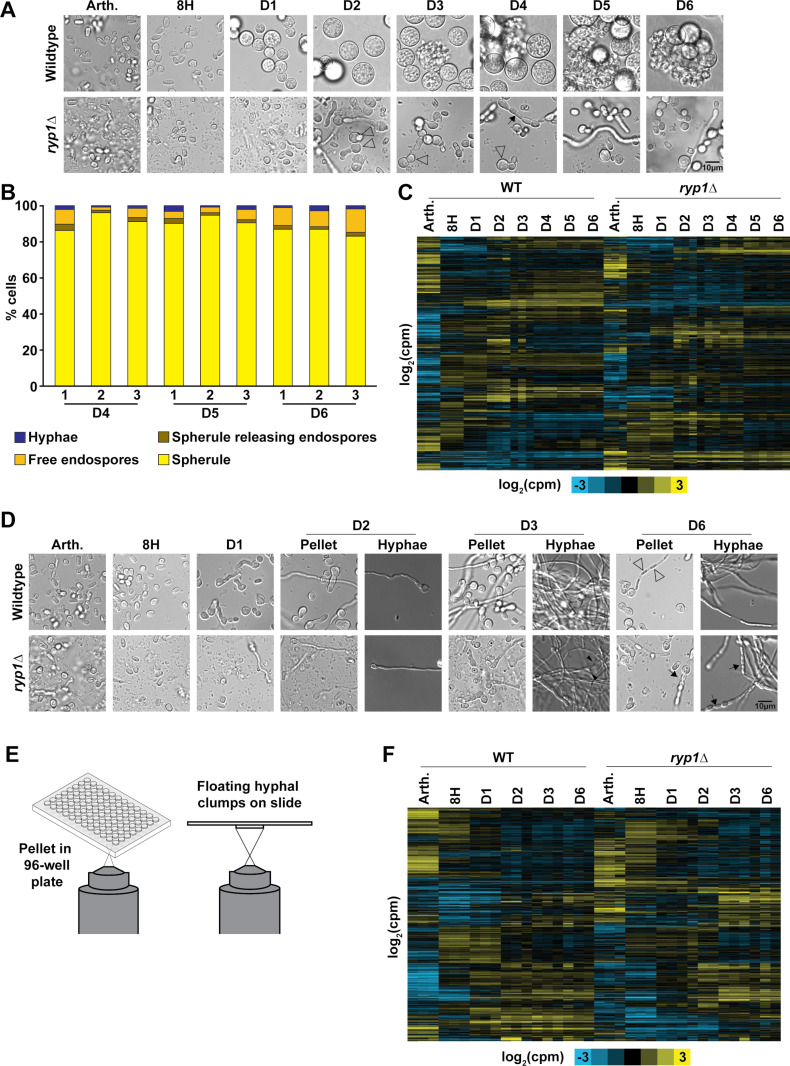
Spherule and hyphal transcriptomes are dependent on *RYP1.* (A) Micrographs of fixed samples from each flask at the time of RNA harvest for one replicate of spherule growth in Converse, 39°C, 10% CO_2_. Subsequent samples were taken from the same flask over time. Endospore release was first observed on day 3 in wildtype. As expected, the *ryp1∆* mutant did not form spherules under these conditions. Surprisingly, it did form smaller rounded structures of unclear significance (open arrowheads) in addition to hyphae and some chains of oblong cells (black arrows) which have not been reported previously in *Coccidioides* to our knowledge. (B) Quantification of the proportion of each morphology in cultures on days 4–6, *n* ≥  20 fields of view counted for each sample. Underlying data can be found in S5 Table. (C) Heatmap of transcript abundance over time in spherulation conditions. Transcripts that had at least 10 reads detected in at least three samples were included as mean-centered rows in this heatmap. Rows are clustered based on correlation across all columns. Log_2_(counts per million) indicated by yellow and blue shading. (D) Micrographs of fixed samples from each flask at the time of RNA harvest for one replicate of hyphal growth in Converse, 25°C. Subsequent samples were taken from the same flask over time. “Pellet” and “Hyphae” are the same biological samples prepared in different ways as described in E. Open arrows indicate hyphae forming initial arthroconidia. Black arrowheads indicate branching hyphae. Black arrows indicate chains of oblong cells similar to those observed for *ryp1∆* in spherulation conditions. (E) Schematic of preparation of hyphal samples for microscopy. “Pellet” samples were placed in a 96-well plate with glass bottom and pelleted at 584 × g for 2 min prior to visualization. For “hyphal” samples, 5 µL of fixed samples containing small clumps of hyphae were placed on a slide with a coverslip prior to visualization. (F) Heatmap of transcript abundance over time in hyphal conditions, displayed in the same manner and with the same criteria for inclusion as in C.

Over the course of this analysis, we used two strategies to characterize the spherule transcriptome and to identify key spherule-associated transcripts. We determined which transcripts were regulated by the critical transcriptional regulator Ryp1 (*RYP1*-dependent genes), and we compared the spherulation transcriptome to the hyphal transcriptome to identify transcripts that were associated with each morphology (morphology-dependent genes). The TF Ryp1 is required for spherulation in *Coccidioides* [12]. We reasoned that understanding the portion of the spherule transcriptome that is dependent on *RYP1* would identify transcripts whose expression is associated with spherule formation rather than the conditions used to generate spherules. First, we germinated both wildtype and *ryp1∆* arthroconidia under spherulation conditions, observed morphology by light microscopy, and performed RNA-Seq at the same timepoints as the previous experiment, now sampling from the same culture over time to increase consistency between subsequent timepoints of development (Figs 2A and S2A). Of note, the particulate matter in the *ryp1∆* cultures was present in arthroconidia stocks and likely represents cellular debris carried over into spherulation cultures. This has been observed in prior literature and likely reflects the previously reported low viability of *ryp1∆* arthroconidia [12]. To compare the wildtype spherules generated in our first and second experiments, we determined the time of endospore release, the quantity of endospore release, and spherule size. The wildtype strain did release endospores starting on day 3, but there was quantitatively less endospore release in this experiment (Fig 2B and S5 Table). Wildtype spherules achieved a similar diameter by day 6, as seen previously (S2B Fig and S6 Table). Again, the arthroconidia transcriptome was the most distinct within a genotype, with sets of transcripts being induced/repressed as spherules formed and transcripts showed substantial dependence on *RYP1* (Fig 2C and S7 Table). We also observed a similar pattern of decreased differential transcripts between subsequent timepoints as the experiment progressed (S2C Fig). Therefore, we conclude that these separate spherule development trajectories are comparable except for the endospore release stage.

To simultaneously query the transcriptome of the hyphal morphology, we germinated the same arthroconidia stocks of wildtype and *ryp1∆* in hyphal-inducing conditions. It has been common to compare spherules grown in Converse medium to hyphae grown in a different rich medium (GYE) [7,8], but, to eliminate media-specific expression effects, we generated hyphae in Converse medium (at ambient temperature without additional CO_2_). At each timepoint, we observed hyphae formation by light microscopy and the transcriptome by RNA-Seq (Figs 2D and S2D). To best capture the heterogeneity of hyphal cultures, we performed light microscopy using two different modalities (Fig 2E). Pelleted samples provided higher sensitivity for short filaments, whereas slides were used to examine longer hyphae and mature hyphal mats that did not pellet. We observed that wildtype samples formed germ tubes by day 1, with extension and early branching on day 2, followed by robust hyphal mats on day 3. We expected older hyphae to undergo arthroconidia generation and observed early evidence of arthroconidia formation on day 6 (Fig 2D, open arrows). The *ryp1∆* mutant also demonstrated rare germ tubes on day 1 but appeared to have delayed hyphal branching as we did not observe branching structures until day 3 (Figs 2D and S2D, black arrow heads). On day 6, instead of early arthroconidia development, *ryp1∆* demonstrated aberrant morphology with chains of rounded and oblong structures (Fig 2D, black arrows), similar to the morphology at late timepoints in spherulation conditions. *ryp1∆* arthroconidia (same biological samples as seen in Fig 2C) demonstrated significantly different expression compared to wildtype arthroconidia, but wildtype and mutant hyphal transcriptomes started to resemble each other more closely over time (Fig 2F and S7 Table), suggesting that *RYP1* is largely dispensable for the hyphal transcriptome. As observed with spherulation, we found a similar pattern of decreased differential transcripts between subsequent timepoints as the experiment progressed (S2E Fig).

### Identifying *RYP1*-dependent and morphology-dependent transcripts during spherule and hyphal formation

**Fig 3:**
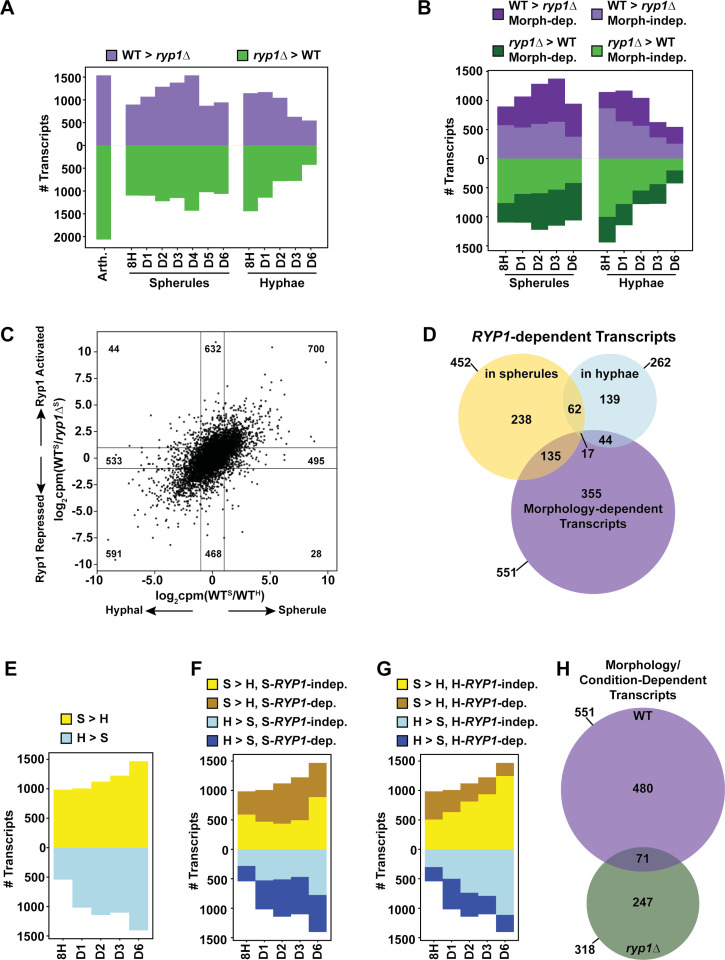
Defining *RYP*1-dependent and morphology-dependent transcripts. (A) Number of significantly differential transcripts between wildtype and the *ryp1∆* mutant at each timepoint specified. Transcripts that are induced by *RYP1* (higher in wildtype than *ryp1∆*) are in purple, and transcripts that are repressed by *RYP1* (higher in *ryp1∆* than wildtype) are in green. (B) As in A but only with timepoints where paired spherule and hyphal wildtype datasets are available to highlight morphology-dependent genes. Dark purple and dark green correspond to the number of *RYP1*-dependent transcripts that are also morphology-dependent (significantly differential between wildtype spherules and wildtype hyphae) at that timepoint. (C) Scatterplot demonstrating the expression of all detected transcripts at day 3. On the x-axis, values are the ratio of wildtype spherule over wildtype hyphal transcript abundance transformed to log_2_(counts per million). On the y-axis, values are the ratio of transcript abundance in wildtype spherules over *ryp1∆* in spherulation conditions, transformed to log_2_(counts per million). (D) Overlap between transcripts that are significantly differential between wildtype and the *ryp1∆* mutant at all timepoints of spherulation (days 1–6, excluding the 8 h timepoint since spherules and hyphae had not appeared by then), transcripts that are differentially expressed between wildtype and the *ryp1∆* mutant over all timepoints of hyphal formation (days 1, 2, 3, and 6), and transcripts that are morphology-dependent in wildtype at all comparable timepoints (days 1, 2, 3, and 6). (E) Number of significantly differential transcripts between wildtype spherules and hyphae at each timepoint specified. Transcripts with higher abundance in spherules than hyphae are yellow and transcripts with higher abundance in hyphae than spherules are blue. (F) As in E, now highlighting dark yellow and dark blue transcripts corresponding to the number of morphology-dependent transcripts that are also regulated by *RYP1* at the corresponding spherule timepoint. (G) Same graph as F except the dark yellow and dark blue transcripts now correspond to the number of morphology-dependent transcripts that are regulated by *RYP1* at the corresponding hyphal timepoint. (H) Overlap between transcripts that are significantly differential between wildtype spherules and hyphae at all comparable timepoints (days 1, 2, 3, and 6) and transcripts that are differentially expressed between the *ryp1∆* mutant in spherulation and hyphal-inducing conditions at the same timepoints.

To further refine our understanding of the *Coccidioides* transcriptome and to elucidate the molecular role of *RYP1* in *Coccidioides* development, we examined which transcripts are significantly differential in wildtype compared to the *ryp1∆* mutant at each timepoint of spherulation or hyphal growth, termed “*RYP1*-dependent” (Fig 3A). Surprisingly, the highest number of *RYP1*-dependent transcripts was in arthroconidia, where a role for Ryp1 in gene regulation has not been interrogated previously. This effect was observed regardless of arthroconidia storage conditions prior to use (S3A and S3B Fig) and indicates a previously unknown and significant role for Ryp1 in the transcriptome of arthroconidia, the infectious particle of this fungus, that bears further study.

During germination into spherules, there were increasing numbers of *RYP1*-dependent transcripts until a peak at day 4, with more transcripts induced by *RYP1* (purple) versus relatively constant numbers of transcripts repressed by *RYP1* (green) (Fig 3A). In contrast, cells in hyphal-inducing conditions trended toward fewer *RYP1*-dependent transcripts over time, indicating that the wildtype and *ryp1∆* hyphal transcriptomes converge as both genotypes differentiated into hyphae (Fig 3A). At each timepoint sampled in both spherule and hyphal conditions, we highlighted *RYP1*-dependent transcripts that were also “morphology-dependent” (significantly differential between spherules and hyphae in wildtype cultures at the same timepoint) or “morphology-independent” (Fig 3B, dark and light regions, respectively). In contrast to stable numbers of *RYP1*-dependent morphology-independent genes, there was an increase in the number of *RYP1*-dependent morphology-dependent transcripts in spherules from 8 h to day 3. This increase was largely driven by two groups of transcripts: (1) *RYP1*-activated, spherule-activated or (2) *RYP1*-repressed, hyphal-activated. This trend is more easily observed in Fig 3C, where the day 3 data are plotted, and in the global analysis in S3C and S3D Fig. In hyphal-promoting conditions, this trend was not observed, and both *RYP1*-dependent, morphology-dependent and *RYP1*-dependent morphology-independent transcripts decreased over time with no clear correlation between the hyphal-*RYP1*-dependent transcriptome and the morphology transcriptome (Figs 3B, S3C and S3D). Thus, during spherule development, *RYP1* has an impact on the morphology regulon that increases with time and peaks on day 3, as well as a morphology-independent impact with constant magnitude over time. On the contrary, in hyphal development, *RYP1* has a largely morphology-independent impact on the transcriptome that decreases over time, indicating that wildtype and *ryp1∆* hyphae converge on similar transcriptomes.

Next, to further understand the role of *RYP1* in *Coccidioides* biology, we examined the stringent set of transcripts that were *RYP1*-dependent across all spherule or hyphal timepoints (Fig 3D). There were 452 transcripts consistently *RYP1*-dependent across all six timepoints in spherulation conditions (termed “S-*RYP1*-dependent”) and 262 transcripts across all four timepoints in hyphal conditions (termed “H-*RYP1*-dependent”). Of these 452 S-*RYP1*-dependent and 262 H-*RYP1*-dependent genes, 79 were common to both sets (*p* =  1.94e-38 by Fisher exact test). While significant, this relatively low magnitude of overlap adds additional evidence for *RYP1*’s distinct regulatory roles in spherules and hyphae. We also found 551 consistently morphology-dependent transcripts by comparing wildtype spherules and hyphae. 152 (135 +  17) of these strictly morphology-dependent transcripts were also consistently S-*RYP1*-dependent (*p* =  4.97e-71 by Fisher exact test), and 61 (44 +  17) strictly morphology-dependent genes were consistently H-*RYP1*-dependent (*p* =  1.69e-18 by Fisher exact test). The 17 transcripts that are S-*RYP1*-dependent, H-*RYP1*-dependent, and morphology-dependent include the gene D8B26_005342/CIMG_00509, which is already known to be spherule-induced, *RYP1*-dependent [12], and, interestingly, in an area of genomic introgression between the two known *Coccidioides* species, *Coccidioides posadasii* and *Coccidioides immitis* [23]. This central overlap of 17 is surprisingly low, although still significantly higher than expected by chance (χ^2^ =  1019.27, *p* <  0.0001), and again implies that *RYP1* has two distinct regulatory roles in these two morphologies, more significant in spherules compared to hyphae. The high number of morphology-dependent genes that are *RYP1*-independent suggests roles for additional regulators of spherulation.

### Morphology change triggers differential expression of a core set of transcripts across all developmental time points

Next, we examined morphology-dependent transcripts at each shared timepoint of spherule and hyphal development in wildtype (Fig 3E). As expected, the number of morphology-dependent transcripts increased over time as spherules and hyphae emerged. We highlighted the morphology-dependent transcripts that were also S-*RYP1*-dependent (Fig 3F) at the same timepoints and observed an increase in the magnitude of this subset of transcripts with the exception of day 6, while morphology-dependent S-*RYP1*-independent genes remain relatively constant (with the same exception of day 6). On the contrary, when we highlighted the number of morphology-dependent genes that are H-*RYP1*-dependent at the same timepoints, that number is relatively small (Fig 3G) and does not have a clear trend. Thus, the role of *RYP1* in regulating morphology is related to its regulon in spherulation conditions, where it induces spherule-associated transcripts and suppresses hyphal-associated transcripts. In hyphae, *RYP1* regulates a small subset of transcripts, but most of these seem to be morphology-independent.

Finally, we defined a stringent set of transcripts that were consistently morphology-dependent across all shared spherule and hyphal timepoints (Fig 3H). As discussed above, 551 transcripts were consistently morphology-dependent in wildtype. A total of 318 transcripts were consistently differential in the *ryp1∆* mutant growing in spherulation conditions compared to hyphal conditions, even though the mutant forms hyphae under both these conditions. Given the uniform morphology, these 318 transcripts are likely responding to the difference in spherulation- and hyphal-inducing conditions (namely, temperature and CO_2_). Surprisingly, the overlap between the 551 morphology-dependent genes in wildtype and the 318 condition-dependent transcripts in *ryp1∆* is low in magnitude (71 transcripts total, *p* =  2.56e-20 by Fisher exact test), meaning that the majority of the 551 morphology-dependent transcripts are linked to the morphology itself.

Focusing on the 551 transcripts with morphology-dependent expression in wildtype, 273 are consistently spherule enriched (of those, 82 are also consistently S-*RYP1*-dependent), and 239 are hyphal enriched (of those, 32 are also consistently H-*RYP1*-dependent). We examined these subsets further at the gene level to better understand the molecules involved in the *Coccidioides* morphologic transition. Within the spherule-enriched set, as expected, we found the transcript encoding the best-characterized virulence factor in *Coccidioides*, SOWgp [24] (D8B26_003939), and the previously reported spherule-associated gene *PSP1* [7,8,12,25] (D8B26_002733). We also found D8B26_003869, the ortholog of *BOI2* in *Saccharomyces cerevisiae*, a gene involved in polar growth and inhibition of cytokinesis during budding [26], which may imply a role for directed vesicle fusion with the plasma membrane or a delay in cytokinesis during spherule development. Additionally, there are two TFs (D8B26_005038 and D8B26_006698) in this group that are good candidates for regulators of spherulation in addition to *RYP1*. Of note, *OPS1* [12,25] (D8B26_004398) and *ALD1* [25,27] (D8B26_007314), genes that were previously published to be spherule-biased, were found to be spherule enriched in some early timepoints but not consistently at later timepoints of morphological development, demonstrating the power of this high-density developmental time course. Finally, despite the critical role *RYP1* plays in inducing spherulation in *Coccidioides*, the *RYP1* transcript itself does not demonstrate morphology-specific expression (S3E Fig). In the consistently hyphal-enriched transcripts, we found *STU1* (D8B26_002234), the *Coccidioides* ortholog of *Aspergillus* APSES family TF *STUA* which regulates conidiation [28]. This finding matches the ortholog of *STUA* in *Histoplasma*, *STU1/EFG1*, which is extremely hyphal-biased in its expression [29]. Consistent with previous findings, the major component of the woronin body structure that plugs damaged areas of hyphal walls, *HEX1* (D8B26_006047), was up-regulated in hyphal conditions compared to spherules [7]. As expected, these hyphal-associated genes were also up-regulated in *ryp1∆* cells in both spherule- and hyphal-inducing conditions. Somewhat unexpectedly, the cytosolic catalase (D8B26_007217) was found to be consistently higher in hyphal conditions and the *ryp1∆* mutant. This gene has been previously found to have higher expression in spherules than hyphae [8,12] in studies in which the spherules and hyphae were grown in different media. Given the discordant findings between our data and previous publications, we believe nutritional cues play a key role in regulating this particular transcript. Thus, our rich dataset identifies 551 consistently morphology-dependent transcripts that are prime effector and regulatory candidates for control of the *Coccidioides* developmental program, deconvolutes the effects of change in growth conditions from change in morphology, and identifies 273 spherule-enriched genes that are likely to be involved in virulence.

### Ryp1 binds to two distinct subsets of promoters

**Fig 4:**
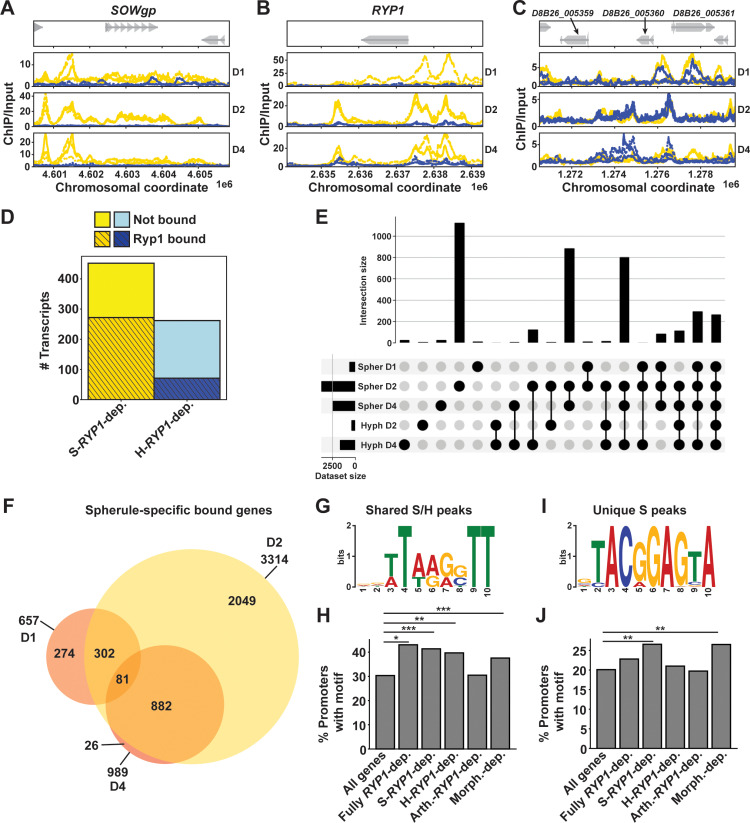
Ryp1 regulates two distinct subsets of targets. (A) Traces demonstrating chromosomal location of fold enrichment of ChIP signal/input in spherules (yellow) and hyphae (blue) at the designated timepoints relative to the annotated *SOWgp* gene. (B) As in A but demonstrating ChIP signal/input relative to the annotated *RYP1* gene. (C) As in A but demonstrating ChIP signal/input relative to D8B26_005359, D8B26_005360, and D8B26_005361 genes (genes indicated by gray arrows). (D) Barplot demonstrating the proportion of S-*RYP1*-dependent genes or H-*RYP1*-dependent genes (as defined in 3D) whose promoters have Ryp1 binding by ChIP-Seq. (E) UpSet plot demonstrating the size of each individual set of genes whose promoters are bound by Ryp1 at each designated spherule/hyphal timepoint (bottom left) and size of overlap between each of these sets (magnitude on top, overlapping sets demonstrated by connected black circles on bottom). Each gene can only be assigned to one unique category. (F) Overlap of spherule-specific peaks (genes whose promoter is bound in spherule timepoints only, without Ryp1 binding in the corresponding hyphal timepoint or *ryp1∆* mutant subjected to the same conditions as wildtype) on days 1, 2, and 4. (G) Motif enriched in DNA sequences of Ryp1 ChIP-Seq peaks found consistently in both days 2 and 4 spherule and hyphal datasets (498 sites, *p* =  4.7e-064 on day 4 and 193 sites, *p* =  3.7e-046 on day 2). (H) Percent of genes in each subset (from 3A, 3D) whose promoter regions have at least one hit for the Ryp1 binding motif in 4G. Promoters are defined as the sequence upstream of the coding sequence (CDS) start until the next upstream CDS is encountered, or 10 kb maximum. Fully *RYP1*-dependent genes are those that are significantly differential between wildtype and *ryp1∆* in all spherule timepoints (days 1–6) and all hyphal timepoints (days 1, 2, 3, and 6). * : *p* < 0.05, **: *p* < 0.005, by Fisher exact test. (I) Motif enriched in DNA sequences of Ryp1 ChIP-Seq peaks found uniquely in days 2 and 4 spherule datasets (>1,000 sites, *p* =  1.3e-044 on day 2, 215 sites, *p* =  4.5e-046 on day 4). (J) As in H but now for Ryp1 binding motif in I.

We next sought to determine which *RYP1*-dependent genes displayed association with Ryp1 using ChIP-Seq with an antibody generated against a peptide epitope of Ryp1. While we attempted to perform ChIP on arthroconidia and multiple timepoints of spherule or hyphal growth (8 h, D1, D2, D4, micrographs in S4A Fig) and one timepoint for each morphology of the *ryp1∆* mutant (micrographs in S4B Fig), consistent Ryp1 binding was only detectable for spherules on days 1, 2, and 4 and hyphae on days 2 and 4 (S8 Table). This lack of binding in wildtype may be due to less initial biomass and does not necessarily reflect a lack of Ryp1 binding at those early timepoints. In the *ryp1∆* mutant, we expected very little binding of the Ryp1 antibody, and while we did identify sporadic peaks in individual replicates, they were not reproducible and likely represented low-level off-target binding of the antibody. We focused our subsequent analyses on those later timepoints with >400 detected peaks in at least two of three replicates. As expected, we observed Ryp1 binding in spherules at the *SOWgp* promoter (Fig 4A), consistent with observations in this and prior studies [11,12,30] that have found *SOWgp* expression to be spherule-associated and *RYP1*-dependent. Additionally, we examined the *RYP1* locus itself and found that Ryp1 bound both upstream and downstream of the gene, suggesting a possible autoregulatory mechanism for *RYP1*, a known characteristic for Ryp1 orthologs in other fungi [15,16,31,32] (Fig 4B). Some promoters in *Coccidioides* are very large and demonstrate Ryp1 binding peaks far from the predicted ATG (S4C Fig), including the intergenic regions between D8B26_007678, encoding a hyphal-enriched hypothetical protein, and D8B26_007679, encoding a spherule-enriched predicted NAD kinase (S4D Fig). Finally, we found examples of Ryp1 binding in hyphae and spherules (Fig 4C), including D8B26_005360 and D8B26_005361, both *RYP1*-repressed transcripts that encode hypothetical proteins. Upon manually reviewing the 32 genes designated as bound in hyphal samples only, we found evidence of binding in spherule samples as well and believe these are instances in which macs2 did not correctly identify peaks in the paired spherule timepoint. Therefore, we do not think there are any examples of Ryp1 binding promoters in hyphal samples alone.

We determined which *RYP1*-dependent genes observed by RNA-Seq were also direct targets of Ryp1 by ChIP-Seq. We found that 60% of the 452 S-*RYP1*-dependent transcripts also demonstrated Ryp1 promoter binding in at least one spherule timepoint of our ChIP-Seq experiment (Fig 4D). In contrast, only 27% of the 262 H-*RYP1*-dependent genes had Ryp1 promoter binding in hyphae. We additionally examined the percentage of the 551 morphology-dependent genes we had previously defined (and the 786 morphology-dependent genes we found from the paired RNA-Seq from this ChIP-Seq experiment, S9 Table) that had Ryp1 binding in their promoters. We found ~55% of both subsets of morphology-dependent genes were Ryp1 targets (S4E Fig). Thus, Ryp1 plays an important role as a direct regulator of morphology in *Coccidioides*, where it seems to act specifically by binding promoters in spherule development. Although the loss of *RYP1* influences the hyphal transcriptome, the ChIP-Seq data suggest that effect is more indirect.

Next, we created an UpSet plot (Fig 4E) to group genes whose promoters had Ryp1 binding detected. This analysis revealed that most genes fell into three categories: (1) genes whose promoters are bound at the spherule day 2 timepoint only, (2) genes whose promoters are bound in both later spherule timepoints, and (3) genes whose promoters are bound in both later spherule timepoints in addition to the latest hyphal timepoint. Taken together, this likely indicates two distinct regulons for Ryp1: an exclusive spherule regulon and a shared spherule/hyphal regulon. Of note, there were minimal numbers of genes whose promoters demonstrate Ryp1 binding in hyphae only, indicating that there does not seem to be a unique Ryp1 regulon in hyphae. Given the large number of spherule Ryp1 peaks detected, we further delved into those genes that had spherule-specific promoter binding (without any observed Ryp1 peaks in hyphal samples or *ryp1∆* samples). In examining the genes containing spherule-specific peaks across the spherule timepoints (Fig 4F), we found that the Ryp1 spherule regulon appears to be dynamic, with 274 genes bound by Ryp1 only in day 1 spherules, 2049 unique genes bound by Ryp1 in day 2 spherules, and 882 genes bound by Ryp1 in both of the later spherule timepoints. Thus, Ryp1 in *Coccidioides* appears to have a complex and dynamic role in regulating multiple stages of the spherule morphology, suggesting it plays a role in a complex regulatory network like some of its orthologs in other fungi [15,33,34].

### Two distinct Ryp1 motifs in *Coccidioides* are enriched in promoters of Ryp1-bound genes

To better understand how Ryp1 can regulate two distinct subsets of genes, we performed motif searches on multiple subsets of Ryp1 peaks combined as illustrated in S4F Fig: (1) peaks found in promoters of genes in both spherules and hyphae and (2) peaks found in promoters of genes only in spherules. In the first group, we discovered a significantly enriched motif (Fig 4G) that is extremely similar to the previously published Ryp1 motif in *Histoplasma* (S4G Fig). We used MAST to search for this Ryp1 spherule/hyphal motif in all *Coccidioides* promoters in the genome with the threshold E-value of 2.08e-04, a cutoff which proved useful for this analysis in *Histoplasma* [15]. Since the motif has low information content, we found that 30% of all promoters had a hit to the Ryp1 motif (Fig 4H). The percent of promoters containing Ryp1 motif hits was highest for genes whose transcripts are *RYP1*-dependent across all timepoints studied and genes whose transcripts are *RYP1*-dependent across all spherule timepoints (S-*RYP1*-dependent). Since this motif was derived from peaks found in both spherule and hyphal morphologies, unsurprisingly, the enrichment of the Ryp1 binding motif in the promoters of H-*RYP1*-dependent genes and morphology-dependent genes was also quite high (~40%). Finally, we found no enrichment of the motif in the 3,599 *RYP1*-dependent transcripts in arthroconidia, suggesting that *RYP1* may control this regulon indirectly through a second major regulator. Together, this motif analysis indicates that the presence of the Ryp1 motif in promoters alone does not explain the varying impact of *RYP1* on spherules, hyphae, and arthroconidia that we observed by RNA-Seq. Similar to peak distribution in promoters, the motif was found distributed over a wide range of distances, with a strong bias toward proximity to the gene (S4H Fig). Next, we examined the number of Ryp1 motif hits per promoter for each of these subsets of Ryp1-motif-containing promoters (S4I Fig). Interestingly, about 40% of S-*RYP1*-dependent genes with motif hits had more than one motif hit, including some promoters with up to five total Ryp1 motif hits. This trend toward more motif hits was unique to S-*RYP1*-dependent genes and may provide a clue toward the mechanism by which Ryp1 has more impact on the transcriptome in spherules despite a shared Ryp1 DNA binding sequence in both spherules and hyphae.

Second, we performed motif searches on peaks that were found only in spherule conditions and discovered a novel motif that has not been reported before for Ryp1 association in any organism (Fig 4I). Given the extremely different sequence from the canonical Ryp1 motif described above, we hypothesize that this motif reflects recruitment of Ryp1 to these promoters via interaction with a second (unknown) regulator that binds this motif directly. Using a more stringent E-value of 1e-06 given the higher information content in this motif, we found that it was present in 20% of promoters across the genome. This motif was significantly enriched in S-*RYP1*-dependent and morphology-dependent gene promoters (Fig 4J). There was a similar distribution of the position of this motif relative to the gene compared to the canonical Ryp1 motif except for mildly decreased numbers of motif hits more than 8 kb from the gene ATG (S4J Fig). Unlike the canonical Ryp1 motif discussed above, there was not a similar trend toward increased numbers of Ryp1 motif hits per promoter in any gene subsets (S4K Fig). Interestingly, S-*RYP1*-dependent and morphology-dependent genes have significantly longer promoters than other gene subsets, which may accommodate both motifs we identified and potentially more numbers of the canonical Ryp1 motif in S-*RYP1*-dependent genes (S4L Fig). Thus, we find that distinct Ryp1-associated motifs, number of motifs per promoter, and potentially combinatorial motifs could contribute to the ability of Ryp1 to possess two distinct regulons.

### Candidate transcription factors for regulation of spherulation

**Fig 5:**
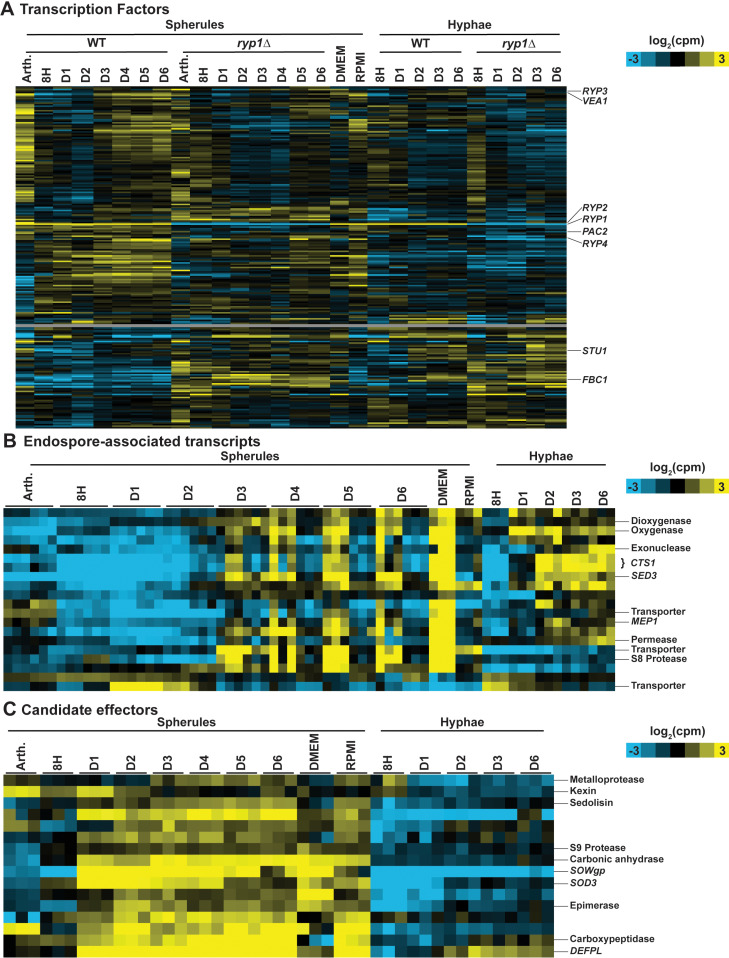
Defining *Coccidioides* transcription factors (TFs), endospore-associated genes, and candidate effectors over spherulation and hyphal development. (A) Heatmap of transcript abundance for all predicted TFs in *Coccidioides*. Expression data all from experiments described in Fig 2, with additional DMEM (Dulbecco’s Modified Eagle Medium) and RPMI (Roswell Park Memorial Institute) media conditions as described in S5A, S5B Fig. Rows are clustered based on correlation across all columns. Log_2_(counts per million) indicated by yellow and blue shading. Gray rows indicate TFs that did not have sufficient counts to pass the filter threshold for RNA-Seq analysis. (B) Heatmap of transcript abundance for endospore-associated genes. Each spherule timepoint shows six replicates: The first three are from the experiment in Fig 1 and the last three are from the experiment in Fig 2. DMEM and RPMI timepoints are as described in S5A and S5B Fig. Rows are clustered based on correlation across all columns. Log_2_(counts per million) indicated by yellow and blue shading. (C) Heatmap of transcript abundance for putative spherule-expressed secreted effectors. Expression data all from experiment described in Fig 2, with additional DMEM and RPMI conditions as described in S5A, S5B Fig. Rows are clustered based on correlation across all columns. Log_2_(counts per million) indicated by yellow and blue shading.

As the effect of Ryp1 on the transcriptome does not fully explain the morphology transition of *Coccidioides*, there are likely additional regulators involved in switching morphology and the maintenance of spherulation. To generate additional candidates, we created a list of 227 possible TFs in *Coccidioides* and examined their expression in the RNA-Seq data from the experiments in Fig 2 (Fig 5A and S10 Table). Most of the TF candidates (151) are expressed more highly in spherules compared to hyphae, including *RYP2* and *RYP4* which are part of a regulatory network that acts with *RYP1* to control morphology of the related fungus *Histoplasma* [15]. Interestingly, 86 of these spherule-expressed TFs that are highly expressed in late spherule timepoints are also highly expressed in arthroconidia, including *RYP4, PAC2* [35] (the paralog of *RYP1*), and *VEA1* (a velvet protein like *RYP2* and *RYP3).* Velvet proteins are unique to fungi and function in regulating developmental processes and secondary metabolism [36]. TFs that are expressed in hyphae more than spherules include *STU1* and *FBC1,* both known to be expressed in hyphae in *Histoplasma* [29,37] and involved in hyphal development and conidiogenesis in *Aspergillus nidulans* [38,39]. Excluding *RYP1* itself, 16 TF candidates were found in the 452 S-*RYP1*-dependent transcripts defined above, including three candidates that were also found in the 262 H-*RYP1*-dependent transcripts. Fourteen of the 16 S-*RYP1*-dependent TF candidates also exhibited Ryp1 binding in at least one spherule timepoint. Given the central role of *RYP1* in regulating the morphology transition, these 14 TFs that are direct regulatory targets of *RYP1* are good candidates to be additional members of the regulatory network that controls morphology in *Coccidioides*. Ten of these candidates are repressed by *RYP1* in spherules and include *FBC1*. The remainder of these direct *RYP1* targets, including the four that are induced by *RYP1* in spherules, are currently unannotated.

### Defining endospore-associated transcripts

Since the endospore form is even less characterized than spherules, we used the RNA-Seq data corresponding to cultures for which we observed the most endospore release to identify potential endospore-enriched transcripts. Specifically, we interrogated day 3 and later timepoints from Fig 1, and also performed additional RNA-Seq from spherules formed in DMEM +  20% FBS and harvested at day 3, when released endospores were abundant (S5A Fig). These spherules were generated from the same arthroconidia as described in Fig 2. We defined endospore-enriched transcripts as those that were all consistently differential on days 3–6 compared to days 1 and 2 in the experiment from Fig 1, all significantly differential in DMEM conditions compared to RPMI +  10% FBS conditions (S5B Fig, also generated from the same arthroconidia as described in Fig 2) which did not exhibit endospore release, and all significantly differential in DMEM conditions compared to day 1 and 2 spherules, day 1 and 2 hyphae, and arthroconidia from the same experiment. For all these differential comparisons, we enforced criteria that the direction of differential expression had to be consistent for the transcript across the comparisons made. Of the transcripts meeting the above criteria, there were 18 transcripts with an increase in expression in samples containing endospores and two transcripts that demonstrated consistent decrease in expression in samples containing endospores (Fig 5B and S11 Table). The transcripts with increased abundance included *MEP1*, a metalloprotease which is known to play a role in masking endospore recognition by the immune system [40], and *CTS1* (misannotated as two separate transcripts D8B26_000666/7 in the current genome), an endochitinase that has been previously characterized to have maximal expression when endospores are present in culture [41]. Interestingly, the endospore-enriched transcripts include two other secreted serine proteases, D8B26_003356 and D8B26_007338. Both transcripts that are consistently downregulated in endospore-containing cultures have no available annotation data. This list of genes represents the first and strongest candidates for factors intimately involved in endospore biology.

### *Coccidioides* spherule secreted effectors are enriched for proteases

Next, we set out to define a set of putative secreted effectors in *Coccidioides*, since these factors are top candidates for interaction with the host immune system. We extrapolated likely characteristics from plant fungal pathogens, where secreted effectors have been extensively studied [42,43]. We filtered for transcripts containing a predicted signal sequence (SignalP 6.0 [44]), that are cysteine-rich (predicted protein product contains ≥  4 cysteines), and whose expression is consistently higher in spherules than hyphae at all timepoints. This yielded a list of 16 genes, 4 of which were also *RYP1*-dependent at all spherule timepoints (Fig 5C and S12 Table): D8B26_003939 which encodes SOWgp, the major component of the spherule outer wall that is exclusively expressed in the spherule form; *DEFPL* (D8B26_005342) which encodes a cysteine-rich protein of unknown function previously published to be highly up-regulated in the spherule morphology [45]; D8B26_005613 which encodes a 245-aa protein of unknown function; and D8B26_005065, a serine carboxypeptidase. Surprisingly, the remaining 12 putative secreted effectors also include five additional proteases (a kexin, an M35 metalloprotease, another serine carboxypeptidase, an S8-like protease, and an S9-prolyl-peptidase). Therefore, more than 40% of the putative secreted effectors we define are secreted proteases and suggest a possible protease-based virulence strategy for *Coccidioides*.

### A cluster of six genes that demonstrate spore-associated *RYP1*-dependent expression affects arthroconidia cell wall development

**Fig 6:**
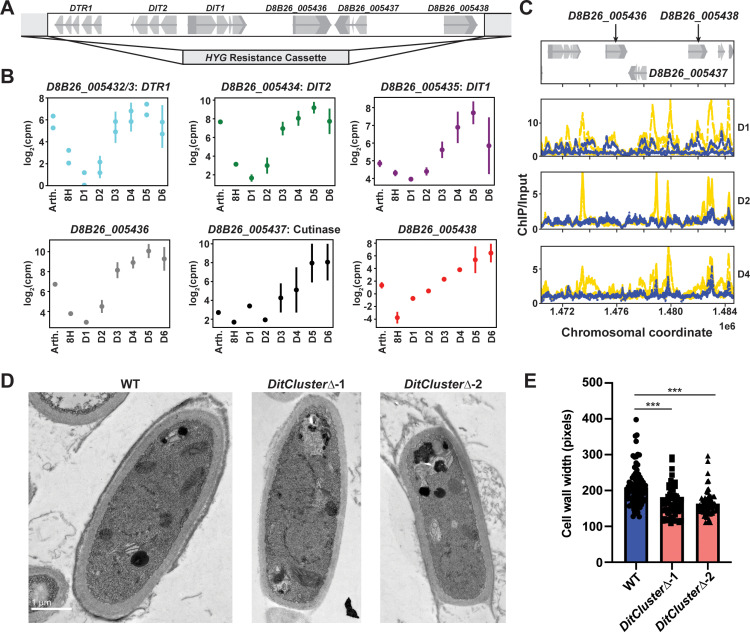
A six-gene cluster plays a role in spore development. (A) Schematic of the location of the six genes in the spore-related cluster and position of hygromycin cassette integration. (B) Plots of log_2_(counts per million) over spherulation from data in Fig 1 for each gene in the cluster. *DIT1*/*DIT2*/*DTR1* names from *S. cerevisiae* orthologs and “Cutinase” name based on Pfam hit to that domain. (C) Traces demonstrating chromosomal location of fold enrichment of Ryp1 ChIP signal/input in spherules (yellow) and hyphae (blue) at the designated timepoints relative to D8B26_005436 – D8B26_005438 genes. (D) Representative TEM images of wildtype and each *DitCluster∆* mutant. The scale is the same for all images. (E) Quantification of arthroconidia cell wall width measurements of TEM images for wildtype and each *DitCluster∆* mutant. ***: *p* < 0.0001, by unpaired *t* test. Underlying data can be found in S14 Table.

Finally, to demonstrate the ability of this extensive resource to uncover new biology, we synthesized the above data and focused on a cluster of six adjacent genes (D8B26_005432 to D8B26_005438, Fig 6A) which demonstrate high transcript accumulation in both arthroconidia and endospores, the two spore forms characterized in this study (Fig 6B). This six-gene cluster includes *DIT1* (D8B26_005435) and *DIT2* (D8B26_005434) [46], genes encoding enzymes predicted to synthesize dityrosine, and *DTR1* (misannotated as D8B26_005432/3 in the current genome) [47], which encodes a bisformyl dityrosine transporter. In *S. cerevisiae*, the orthologs of these genes are involved in the synthesis and assembly of the protective dityrosine layer of *S. cerevisiae* ascospores [48], suggesting the hypothesis that dityrosine or a derivative thereof may play an important role in *Coccidioides* arthroconidia and endospores as well. Given the major role of *RYP1* in arthroconidia biology that we determined from our transcriptomics, we examined whether this cluster of spore-associated genes was regulated by *RYP1*. In *Coccidioides*, the transcripts from this cluster demonstrate a complex dependence on *RYP1* (S13 Table). All members of the cluster are significantly *RYP1*-dependent in at least two timepoints of spherulation, and there is a trend for members of the cluster to require *RYP1* for increased transcript abundance later in spherulation, consistent with three members of the cluster exhibiting Ryp1 binding in later spherule timepoints by ChIP-Seq (Fig 6C). We interpreted these *RYP1*-dependent results as additional support for this cluster playing a role in *Coccidioides* spore biology. Therefore, we interrogated the biological role of this cluster by creating two independent deletion mutants lacking all six genes in the cluster (*DitCluster∆ −*1&2) (Fig 6A). We generated arthroconidia from these mutants and wildtype and examined them by transmission electron microscopy (TEM). We found that mutant arthroconidia exhibited thinner cell walls than wildtype (Fig 6D and 6E and S14 Table). Correlated with this, we also found a modest decrease in the number of visible cell wall layers in the mutant arthroconidia as well (S6A Fig and S14 Table). Wildtype arthroconidia in our strain background also produce a yellow pigment that is spore-associated. In the mutant lacking the full cluster of six genes, the yellow color is decreased. To further interrogate this, we also created two independent deletion mutants lacking only *DIT1*, *DIT2*, and *DTR1* (*DitClusterSmall∆ *−1&2, S6B Fig). While these mutants shared the phenotype of thinner cell walls (S6C Fig and S14 Table), they demonstrated a similar yellow color as wildtype (S6D Fig). This suggests that the three unnamed genes in this cluster play a role in producing the yellow pigment. As expected, none of the mutants had defects in hyphal growth or spherule formation (S6E, S6F, S6G, and S6H Fig and S15 Table). Thus, the criteria we applied to our rich transcriptomic and ChIP-Seq atlas of *Coccidioides* development correctly predicted a role for these genes in a specific developmental morphology of *Coccidioides*. We anticipate that this resource can be used in a similar manner for many additional targets, enabling a much deeper understanding of the biology of this important fungal pathogen [49–77].

## Materials and methods

### Strains and growth conditions

The wildtype *C. posadasii* strain Silveira (NR-48944) [49] was used for growth experiments and as the background for the generation of mutants. All manipulations of live *Coccidioides* strains were performed in a biosafety level 3 facility. Standard spherulation conditions: Polypropylene flasks containing Converse media as previously published [22] were inoculated with 1 × 10^6^/mL arthroconidia (unless otherwise stated) and placed at 39°C, 10% CO_2_, shaking at 120 rpm. Where noted, spherulation was induced under the same conditions with different media: DMEM (UCSF Media Core) containing 20% FBS (Corning) or RPMI (UCSF Media Core) containing 10% FBS. For hyphal growth, polypropylene flasks containing Converse media were inoculated with 1 × 10^6^ arthroconidia/mL and grown at 25°C (Figs 2 and S2) or room temperature (RT) (Figs 4 and S4) shaking at 120 rpm. All timepoints presented below are relative to the day of media inoculation with arthroconidia (time 0), without passaging. All growth experiments were performed in 125 mL polypropylene flasks with 50 mL of media except for experiments in Figs 2 and S2, which were performed in 1 L flasks with 350 mL of media (except for replicate 3 of *ryp1∆* mutant in spherulation conditions, which was placed in 300 mL of media given limited arthroconidia stock, to maintain the same concentration across all samples) and Figs 4 and S4, which were performed in 1 L flasks with 330 mL Converse for wildtype and 500 mL flasks with 100 mL Converse for the *ryp1∆* mutant.

### Microscopy of spherules and hyphae

At stated timepoints for light microscopy, cells were fixed in 4% paraformaldehyde (Electron Microscopy Sciences) at RT for 30 min and washed twice in PBS, pelleting cells by centrifugation for 2 min at maximum speed between washes. Cells were visualized using 40× DICII objective on a Zeiss Axiovert 200 microscope, with additional 1.6× Optovar magnification.

### *ryp1∆* deletion mutant generation

The *ryp1∆* deletion mutant was created as previously described [45]. In brief, phusion polymerase (Fisher) was used to amplify the hygromycin selection cassette (sequence from pMAD91 [50]) using Primer 1 and Primer 2 (S16 Table), with 50 bp sequence complementary to the 5′ and 3′ flanking regions of the *RYP1* gene, D8B26_000722. These primers were used to generate the initial template and, given low efficiency, another round of amplification was performed with Primer 3 and Primer 4 at Tm 58.5°C. 2 µg of repair template DNA was gel extracted and purified using the Qiagen gel extraction kit and concentrated by isopropanol precipitation for transformation. Protoplasts were generated as previously described [51] with minor alterations: 100 mL of liquid 2× GYE media (2% Dextrose [Fisher], 1% Bacto Yeast Extract [Gibco]) were inoculated with 5 × 10^8^ arthroconidia and incubated shaking at 140 rpm, 30°C for ~18 h until germ tubes were visible by light microscopy. Cells were then centrifuged at 2,800 × g for 10 min at RT, washed twice in 15 mL osmotic buffer A (OBA: 50 mM potassium citrate [Sigma], 0.6 M KCl [Fisher] at pH 5.8), and resuspended in cell wall digestion buffer (*Trichoderma harzanium* lysing enzymes 4 mg/mL [Sigma], Driselase from *Basidiomycetes* 7.5 mg/mL [Sigma] in OBA). Cell wall digestion was performed by shaking platform at 50 rpm, 30°C for 70 min. Protoplasts were pelleted by centrifugation at 900 × g for 10 min at RT and resuspended in osmotic buffer B (OBB at pH 5.8: 10 mM sodium phosphate [Fisher], 1.2 M MgSO_4_ [Fisher]). Trapping buffer at pH 7.5 (100 mM MOPS [Sigma], 0.6 M sorbitol [Sigma]) was overlaid on top of OBB, and phase separation was established through 15 min centrifugation at 2,800 × g at RT. Protoplasts were recovered from the interphase at RT and diluted 1:10 into MOPS buffer containing sorbitol at pH 6.5 (10 mM MOPS [Sigma], 1 M sorbitol [Sigma]). Protoplasts were pelleted by centrifugation at 900 × g for 10 min at RT and washed twice in MOPS buffer containing sorbitol and calcium (MSC buffer at pH 6.5: 10 mM MOPS [Sigma], 1 M sorbitol [Sigma], 20 mM CaCl_2_ [Fisher]). Cas9 ribonucleoprotein complexes targeting each end of *RYP1* were assembled in vitro immediately before use as previously described [52] using the crRNA sequences 1 and 2 (S16 Table) (IDT), the universal Alt-R tracrRNA (IDT), and Cas9 (IDT). Ribonucleoprotein complexes, 2 µg of repair template DNA, and ~10^7^ protoplasts in 100 µL MSC buffer were mixed with 30 µL 60% PEG 3350 (Spectrum) and incubated on ice for 30 min. 900 µL of 60% PEG was added followed by an additional 30 min of incubation at RT. Protoplasts were pelleted at 8,000 rpm for 15 min at RT, followed by discarding 500 µL of supernatant, then an additional 2 min of centrifugation at 8,000 rpm at RT, and removal of the remaining supernatant. The protoplast pellet was resuspended in 500 µL of MSC buffer and combined with melted GYES soft agar (1% dextrose, 0.5% Bacto yeast extract, 1 M sucrose [Sigma], 0.7% Bacto-Agar [BD]) cooled to 46°C and overlaid onto a pre-warmed GYES agar plate (1% dextrose, 0.5% Bacto yeast extract, 1 M sucrose [Sigma], 2% Bacto-Agar). Plates were incubated at 30°C for 48 h. GYE soft agar (1% dextrose, 0.5% Bacto yeast extract, 0.7% Bacto-Agar) with 75 µg/mL hygromycin (Invitrogen) was overlaid on colonies, and plates were incubated for an additional 5–7 days at 30°C until colonies appeared on the surface of the agar. Single colonies were transferred to 2× GYE plates with 75 µg/mL hygromycin and grown again at 30°C. Colonies were passaged to fresh 2× GYE plates with 75 µg/mL hygromycin every 5–7 days for nine generations.

### *DitCluster∆* and *DitClusterSmall∆* deletion mutant generation

*DitCluster∆* mutants were generated through the same procedure as described above except for the following alterations: 333 ng of synthesized repair template (S16 Table, Azenta) was used instead of generating this by PCR. Protoplasting was performed similarly but omitting the germ tube washes with OBA, digested with enzyme mixture for 40 min total, and omitting the two final washes in MSC buffer. Ribonucleoprotein complexes were assembled as described above using crRNA sequences 3 and 4 for *DitCluster∆* and crRNA sequences 3 and 5 for *DitClusterSmall∆*. A total of 6 × 10^5^ protoplasts in 100 µL MSC buffer were mixed with 25 µL 60% PEG and incubated on ice for 50 min. The remainder of the transformation was performed as described above. Colonies were passaged on 2× GYE plates with hygromycin for a total of four generations.

### gDNA extraction and *ryp1∆/DitCluster∆*/*DitClusterSmall∆* mutant verification

Hyphae were scraped from a colony and submerged in 700 µL lysis buffer (50 mM Tris pH 7.2 [Fisher], 50 mM EDTA [Fisher], 3% SDS [Fisher], 1% 2-Mercaptoethanol [Biorad]) and bead beat for 2 min at maximum speed (Biospec Mini Beadbeater), then incubated for 1 h at 65°C after which 800 µL phenol/chloroform/isoamyl alcohol (Thermo) was added to each tube and mixed by inverting several times. Tubes were centrifuged at maximum speed for 15 min, and genomic DNA was precipitated from the aqueous phase with 2-propanol (Thermo) and 0.13 M sodium acetate (Thermo), then pelleted by centrifugation at maximum speed for 2 min. The DNA pellets were washed twice with 70% ethanol (Fisher) then dried at 50°C for 5–10 min. DNA was eluted into TE (10 mM Tris-HCl pH 8.0, 1 mM EDTA) + RNAse A (0.15 mg/mL, Qiagen) then stored at −20°C until use. Mutant verification was accomplished by PCRs with primers (S16 Table) designed to query correct integration of the repair cassette at the 5′ (Primers 5/6 for *ryp1∆* and Primers 11/12 for *DitCluster∆* and *DitClusterSmall∆*) and 3′ (Primers 7/8 for *ryp1∆*, Primers 13/14 for *DitCluster∆*, Primers 14/15 for *DitClusterSmall∆)* ends of the cassette, and loss of the native gene sequence (Primers 9/10 for *ryp1∆*, Primers 16/17 for *DitCluster∆* and *DitClusterSmall∆*, and additionally Primers 18/19 for *DitCluster****∆***). Mutants were further verified by whole genome sequencing through SeqCenter (Illumina Whole Genome Sequencing, 2 GB coverage). Reads were aligned to the reference with BWA MEM 0.7.17 [53] using default settings, bedgraph files were generated using BEDTools 2.30.0 [54], and Integrative Genome Viewer [55] was used to visualize resulting coverage. This procedure verified loss of the coding sequence for each intended gene deletion and insertion of the hygromycin cassette at the site of the deleted gene with no off-target hygromycin insertions (by analyzing the position of discordant reads where one read in a pair mapped to the hygromycin cassette). Since we had one isolate of the *ryp1∆* mutant, we further audited that mutant as follows: PILON 1.23 [56] was used to generate a reference-guided assembly of the *ryp1∆* mutant genome from paired-end Illumina reads and the GCA_018416015.2 *C. posadasii* reference [49]. BWA was used to generate alignments as specified above, and those alignments were used as input to PILON in variant mode. This procedure was repeated for 15 iterations and was able to fully assemble the hygromycin cassette insertion that replaced *RYP1* (S1 Genome).

### Arthroconidia generation

Arthroconidia from wildtype or the *ryp1∆* mutant were inoculated onto 2× GYE agar with penicillin/streptomycin (100 U/mL penicillin and 100 µg/mL streptomycin, UCSF Media Core) or 2× GYE agar with 75 µg/mL hygromycin, respectively, in T225 tissue culture flasks and grown for four to six weeks at 30°C, until the hyphal mat appeared dry and flattened as previously described [57]. Antibiotics were included to prevent bacterial contamination during prolonged culture. Arthroconidia were harvested 0–2 days prior to initiating spherulation and stored at 4°C until use. Arthroconidia harvest was performed as previously described [57], by adding PBS (UCSF Media Core) to tissue culture flasks with the hyphal mat, scraping to resuspend, and filtering through a 70-micron mesh filter. Arthroconidia were then washed twice with PBS and resuspended in PBS at appropriate concentrations for downstream assays. Arthroconidia were quantified using a plastic hemacytometer sealed with nail polish.

### RNA extraction and RNA-Seq library preparation

RNA from arthroconidia was collected from the same arthroconidia stock in triplicate by placing 5 × 10^7^ arthroconidia into Trizol LS (Ambion) and bead beating for 2 min. For all other samples, at indicated timepoints, RNA was extracted by pelleting cells by centrifugation at 1,200 × g for 5 min at RT, removing supernatants, and flash-freezing cell pellets in liquid nitrogen. Cell pellets were resuspended in Trizol, thawed, and bead beat for 2 min. Samples were stored at −80°C until all samples at all timepoints in an individual experiment had been collected. RNA was extracted using the Direct-zol RNA Miniprep Plus isolation kit (Zymo) with on-column DNAse digestion step extended for 15–30 min. Sequencing libraries were prepared using the NEBNext polyA mRNA magnetic isolation module and NEBNext Ultra II Directional RNA Library Prep kit with dual-indexed multiplexing barcodes. Library quality and adapter dimer contamination were analyzed using Agilent Bioanalyzer High Sensitivity DNA Chips. An additional round of library size selection was performed using homemade Serapure size selection beads [58] for libraries containing significant adapter dimers. Final library concentrations were measured using the Qubit High Sensitivity or Broad Range reagents depending on estimated library concentration by Bioanalyzer analysis of libraries. Libraries were pooled, and sequencing was performed on a single lane of the HiSeq 4000 at the Center for Advanced Technology (UCSF) (Fig 1), or on two lanes of Novaseq S2 (Fig 2)/NextSeq 2000 P3 (S4 Fig) at the Chan Zuckerberg Biohub San Francisco. All reads were single-end, and there was a median of 9 million reads per sample. Detailed sample and sequencing information for each dataset are presented in S17 Table.

### RNA-Seq data analysis

Analysis was conducted as previously described [12] with alterations below. Briefly, estimated counts of each transcript were calculated for each sample by alignment-free comparison against the predicted mRNA for the published Silveira genome [49] using KALLISTO version 0.46.2 [59]. Further analysis was restricted to transcripts with raw counts ≥10 in at least one sample across an individual experiment. Differentially expressed genes were identified by comparing replicate means for contrasts of interest using LIMMA version 3.30.8 [60]. Genes were considered significantly differentially expressed if they were statistically significant (at 5% FDR) with an absolute log_2_ fold change ≥1 for a given contrast unless otherwise noted in the text.

### Ryp1 ChIP-Seq

50 mL of cultures were collected at the start of the experiment (arthroconidia), 8 h, days 1, 2, and 4 timepoints from spherule or hyphal growth induced as described above. Paired samples for RNA-Seq (50 mL initial culture for arthroconidia and 8 h spherules/hyphae timepoints, then 10 mL of days 1, 2, and 4 spherules/hyphae timepoints) were also collected and processed as above. Cells were immediately crosslinked with 1% formaldehyde (Neta Scientific) and incubated at RT for 20 min, mixing every 4 min. Crosslinking was then quenched with 125 mM glycine (Fisher), and samples were incubated for 5 min at RT, then frozen at −80°C. Frozen samples were collected for all timepoints in the experiment prior to downstream processing. All of the following buffers were made using autoclaved ddH_2_O in baked glassware/DNA-free plastic tubes. Samples were thawed, pelleted, and washed twice with 25 mL of TBS (20 mM Tris-HCl pH 7.5 [Fisher], 150 mM NaCl [EMD]), centrifuging at 3,000 × g for 5 min at 4°C at each step. Pellets were resuspended in 700 µL of 4°C lysis buffer (50 mM HEPES [Fisher]/KOH [Fisher], 140 mM NaCl, 1 mM EDTA [Fisher], 1% Triton X-100 [Acros Organics], 0.1% sodium deoxycholate [Sigma], 2X Halt Protease Inhibitor Cocktail [ThermoFisher], and 0.2X Halt Phosphatase Inhibitor Cocktail [ThermoFisher]). Cells were lysed by 8 × 1 min cycles of bead beating (0.5 mm Zirconia/silica beads [Biospec]) at RT with 2 min rest on ice in between each cycle. Insoluble chromatin was pelleted by centrifugation at 8,000 rpm for 10 min at 4°C, resuspended in 350 µL cold lysis buffer, and then sonicated (Diagenode Biorupter) for 15 cycles (30 s on, 30 s off). Cell debris was removed by centrifugation at 14,000 rpm at 4°C, yielding the soluble chromatin fraction. 10 µL of input DNA was removed from the sample and placed in TE with 1% SDS (Fisher). The remaining chromatin was immunoprecipitated with 5 µg of a polyclonal rabbit antibody against an epitope of Ryp1 (ID: 3878, Epitope: VYRELDKPFPPGEKKRAMKK, Bethyl laboratories) with rotation overnight at 4°C. 50 µL of a 50% slurry of protein A dynabeads (Life Technologies, washed 2× with cold TBS and 3× with cold lysis buffer) were added to each protein/antibody mixture and incubated an additional 3 h at 4°C with rotation. Beads were then pelleted on a magnetic rack and washed 2× with cold lysis buffer (without protease or phosphatase inhibitors), 2× with cold lysis buffer with 500 mM NaCl instead of 140 mM NaCl, 2× with cold wash buffer (10 mM Tris-HCl pH 8.0, 250 mM LiCl [Sigma], 0.5% NP-40 [Fluka], 0.5% sodium deoxycholate, 1 mM EDTA), and 1× with cold TE. Bound protein/DNA complexes were eluted by adding 110 µL of elution buffer (50 mM Tris-HCl pH 8.0, 10 mM EDTA, 1% SDS), vortexing, and incubating 10 min at 65°C, mixing every 2 min. Samples were placed on a magnet, and 100 µL eluate was moved to a new tube. Then, 150 µL TE with 0.65% SDS was added to the same beads, vortexed, and placed back on the magnet, allowing 150 µL to be removed and combined with previous eluate for 250 µL for each sample. 1 µL of proteinase K (20 mg/mL, Qiagen) was added to these IP samples as well as the previously collected input samples. All samples were incubated at 65°C overnight (approximately 16 h). 2 µg of RNase A (Qiagen) were added to each sample, which was incubated at 37°C for 1 h. Samples were then purified using the Zymo ChIP DNA Clean and Concentrator kit. Libraries were prepared using the NEBNext Ultra II DNA Library Prep kit, with an additional round of higher and lower size selection (calibrated to select for 150–350 bp fragment sizes) using homemade Serapure size selection beads. Library quality and adapter dimer contamination were analyzed using Agilent Bioanalyzer High Sensitivity DNA Chips. Final library concentrations were measured using the Qubit High Sensitivity or Broad Range reagents depending on estimated library concentration by Bioanalyzer analysis of libraries. Libraries were pooled, and the final pool was subjected to another round of size selection with homemade Serapure beads to remove the remaining adapter dimers. Libraries were sequenced on two lanes of Novaseq S2 at the Chan Zuckerberg Institute Biohub.

### ChIP-Seq data analysis

Reads were aligned to the Silveira genome [49] using BWA MEM 0.7.17. Peaks were called using the IP samples compared to the control input samples with macs2 version 2.2.7.1 [61], --mfold 5–60, with option --keep-dup set to all, with the nomodel option selected, and a manually set extension size of 197 for all samples based on the estimated fragment size from Bioanalyzer traces. ChIP peaks were assigned to individual genes if any part of the peak fell in the intergenic region between the stop codon of the upstream gene and before the start codon for that gene, limiting the intergenic size to a maximum of 10 kb. Subsequent gene-level analyses were performed on genes whose promoter had peaks assigned in at least two out of three replicates.

### Motif calling

Peaks that were present in all three replicate datasets were combined in an iterative manner into a minimal peak using the following criteria: (1) if the location of the maximum of peak 1 fell between the start and end of peak 2, and vice versa for the maximum of peak 2 falling between the start and end of peak 1, (2) a new combined peak was created using the minimal width possible by choosing one start and one end from the two peaks, and (3) peak 3 was then combined with this new peak using the same criteria as 1 and 2. Then, these highly reproducible peaks were compared between spherule datasets and hyphal datasets at the same timepoints (e.g., spherule day 2 and hyphal day 2), and the same overlap metric described above was used to determine if a peak was bound exclusively in spherules, exclusively in hyphae, or reproducibly in both morphologies at this timepoint. DNA sequences were extracted from these highly reproducible peak regions that fell into each of these categories and used as input for MEME [62] 5.4.1 using the flags -revcomp, -mod anr, -nmotifs 10, -w 10, -dna. Motif searches were done using MAST [63] as previously described [15].

### Generating a list of candidate transcription factors

We used the current Pfam to GO mapping from the GO Consortium (https://current.geneontology.org/ontology/external2go/pfam2go dated 2023/03/07 22:16:20) and developed a list of 246 Pfam accession numbers corresponding to GO terms containing the text “transcription factor,” “sequence-specific DNA binding,” or “regulation of DNA-templated transcription” [64,65]. We manually added additional fungal-specific TFs that were not captured by GO terms (PF04082 [66], PF02292 [66], PF04769 [66], PF09729 [67], PF11754 [68,69], PF00010 [70], PF00096 [71], PF12,756 [71], PF00808 [72], PF04438 [73], PF08618 [74], PF05368 [75], PF08581 [76], and PF01722 [77]) for a total of 260 Pfam families. We then determined which genes in *Coccidioides* had these Pfam domains based on the published genome annotation [49]. Genes with hits to 61 different TF Pfam domains are present in the Silveira genome, representing 280 genes in total. This list was evaluated manually, and 53 false positives were removed for a total of 227 candidate TFs in *Coccidioides*.

### Hyphal radial growth assay

500 arthroconidia were resuspended in 10 µL PBS and spotted in the middle of a 2× GYE agar plate with penicillin/streptomycin (100 U/mL penicillin and 100 µg/mL streptomycin) and incubated at 30°C. Between days 3 and 9, three images of the growing colonies were taken. The colony area was measured and used to calculate the radius. Hyphal growth rate is equivalent to the change in colony radius over time, and results shown are representative of the results of two independent experiments.

### *DitCluster∆* mutant transmission electron microscopy

Wildtype and mutant arthroconidia were generated as described above, except they were grown for eight weeks on 2× GYE agar with penicillin/streptomycin (100 U/mL penicillin and 100 µg/mL streptomycin) before harvest. Arthroconidia pellets were fixed in freshly prepared 2.5% glutaraldehyde (EMS) in 0.1 M cacodylate buffer pH 7.4 (EMS) at RT for 30 min, then pelleted by spinning 14,000 rpm for 1 min. RT fixative was removed, and the cells were resuspended in the same fixative, cooled to 4°C, and stored at 4°C until ready for embedding. They were then post-fixed in 1% OsO_4_ in 0.1 M cacodylate buffer for 1 h on ice and then stained with 2% uranyl acetate for 1 h on ice. The samples were dehydrated in a graded series of ethanol washes (50%–100%) once, followed by a wash with 100% ethanol and two washes with acetone for 15 min each, and then embedded with Durcupan. 70 nm sections were cut on a Leica UCT ultra-microtome and collected on 300 mesh copper grids. Sections were stained with 2% uranyl acetate for 5 min, and Sato lead stain for 1 min. Samples were viewed using a JEOL 1400-plus TEM (JEOL, Peabody, MA). TEM images were taken using a Gatan OneView 4 k × 4 k camera (Gatan, Pleasanton, CA), and the results are representative of two independent experiments.

## Discussion

We report the first transcriptomic atlas of *Coccidioides* developmental programs, from vegetative arthroconidia into spherules releasing endospores, and from arthroconidia into mature hyphal mats, with almost every transcript in the *Coccidioides* genome demonstrating significant differential expression over the conditions we interrogated. These developmental programs triggered a near-full remodeling of the transcriptome. By characterizing the regulatory targets of the major morphologic regulator Ryp1 by ChIP-Seq in *Coccidioides* for the first time, our work demonstrates a clear and specific role for Ryp1 in spherules, a significant but indirect role regulating the transcriptome in arthroconidia, and a shared morphology-independent regulatory role for some spherule and hyphal genes. Using this transcriptomic atlas, we define 20 endospore-associated genes and 16 putative secreted effectors, six of which are, remarkably, all secreted proteases.

### Spherulation is a developmental program

Our data show that spherule development requires near-complete remodeling of the transcriptome, a level of complexity on par or surpassing developmental trajectories of multicellular organisms [78–80]. In fact, the spherule form should be considered a multicellular morphology since it is filled with hundreds of endospores which each contain one or more of their own nuclei. While elegant observational work has established the spherulation cycle and associated morphologies, we have much more to learn about this cell type. We present bulk RNA-Seq here as the first step toward understanding the spherule. However, we acknowledge that there could be (and likely is based on precedent from studies of spores in other organisms) significant heterogeneity between the transcriptome of individual endospores that comprise mature spherules. Additionally, it is unknown whether the spherule retains one or more nuclei that do not develop into endospores and whether there is still a spherule-specific cytoplasm surrounding endospores that may carry out different biologic functions from the endospores themselves. The application of single-cell RNA-Seq and spatial transcriptomics may allow further insights into this aspect of spherulation.

### Ryp1 plays a major regulatory role in arthroconidia

We report the first transcriptomes of arthroconidia in *Coccidioides*, which are completely distinct from the spherule and hyphal transcriptomes. Surprisingly, using the metric of number of transcripts that are significantly differential in abundance between wildtype and *ryp1∆, RYP1* plays a much larger role in regulation of the arthroconidia transcriptome than in spherules or hyphae. We were not able to detect Ryp1 binding by ChIP-Seq in arthroconidia, likely due to too little starting material. However, we used the Ryp1 binding motifs defined in this study, searched for them in promoters of *RYP1*-regulated genes in arthroconidia as defined by RNA-Seq, and found no significant enrichment. These data strongly suggest that *RYP1* impacts a second regulator that directly binds the DNA via a distinct motif, or that a co-regulator modulates Ryp1 binding for this subset of genes. Since the *ryp1∆* mutant has decreased arthroconidia viability [12] and there is precedent that *RYP1/WOR1* is required for proper regulation of conidial development in other fungi [20,33,81], we interpret our findings as indicating that *RYP1* likely is a major but indirect regulator in *Coccidioides* arthroconidia as well.

### Ryp1 plays a more specific role regulating expression in spherules than in hyphae

**Fig 7:**
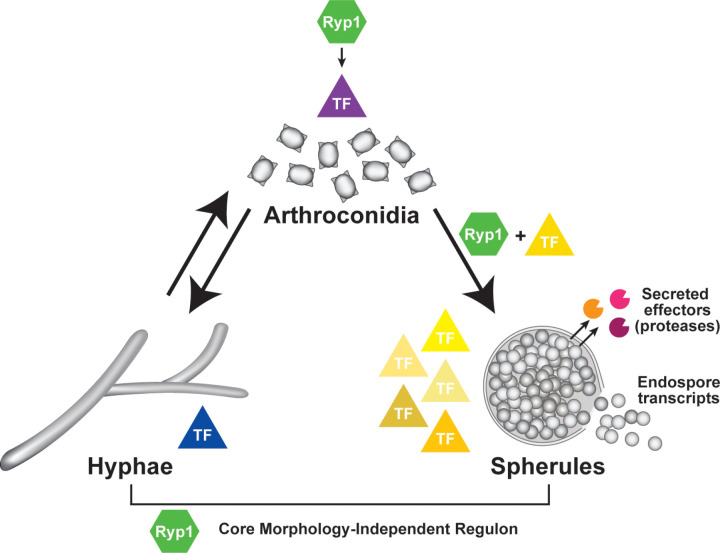
Regulation of gene expression in *Coccidioides* development. Our data uncover regulatory modules in *Coccidioides* development. The majority of *Coccidioides* transcription factors (TFs), depicted as triangles in the figure, show enhanced expression in spherules compared to hyphae. These TFs, along with Ryp1 (green hexagon) and a second regulator whose motif we report here, guide the expression of endospore-associated transcripts and secreted effectors, including proteases. Ryp1 also controls a core regulon that is expressed in both spherules and hyphae via a canonical Ryp1 motif. Finally, in arthroconidia, Ryp1 impacts the transcriptome, but likely through indirect regulation of one or more additional key regulators.

While the *ryp1∆* transcriptome significantly differs from wildtype in arthroconidia and hyphae as well as spherules, our analyses of Ryp1 binding by ChIP-Seq indicate that S-*RYP1*-dependent genes defined by RNA-Seq are much more likely to be direct regulatory targets of Ryp1 than H-*RYP1*-dependent genes. Additionally, there is a large set of genes that only exhibit Ryp1 binding in the spherule morphology and essentially no genes that exhibit Ryp1 binding exclusively in the hyphal morphology. Therefore, our data support a model in which Ryp1 regulates 4 distinct gene subsets: (1) a core set of morphology-independent genes in both spherules and hyphae, (2) a morphology-specific set of genes in spherules that it modulates through direct association with their promoters, (3) a set of hyphal genes whose RNA level is modulated by *RYP1* but likely through another regulator, as Ryp1 does not directly associate with their promoters, and (4) a large set of genes in arthroconidia, again likely through indirect effects through another regulator (Fig 7).

In particular, S-*RYP1*-dependent genes are more likely to have more Ryp1 motif hits per promoter than any of the other gene subsets we studied, which raises the interesting possibility of cooperativity of Ryp1 binding in spherule conditions. Cooperative binding [82] and stochastic switching [31,34,83] have been studied for Wor1, the Ryp1 ortholog in *Candida* species, where Wor1 regulates the white/opaque switch by promoting a specific developmental program. Since Ryp1 both induces and represses the abundance of various transcripts, we hypothesize the existence of additional co-regulators that help mediate the directionality of its regulation in addition to characteristics of the motif’s strength, number, and location within the promoter itself. One of these co-regulators may bind to the second motif discovered to be enriched in genes whose promoters were bound by Ryp1 only in spherules, and the motif sequence could be leveraged to discover the identity of that second regulator. The group of candidate TFs we have presented here can serve as a roadmap for discovering these additional major co-regulators of the morphologic switch in *Coccidioides*.

### Secreted proteases warrant further study in *Coccidioides*

As we have demonstrated, secreted proteases exhibit intriguing dynamic expression during *Coccidioides* spherulation and comprise an outsized membership of the stringent sets of genes we selected based on our transcriptomic data. Three of the 20 endospore-enriched genes we define are secreted proteases. Additionally, six of the 16 putative secreted effectors we defined are also secreted proteases. Prior genomic sequence analysis has found that two secreted protease families, the S8 serine proteases [27] and the M35 metalloproteases [84], are expanded in *Coccidioides* relative to other fungi, with the M35 family also undergoing positive selection. Given the nine secreted proteases we report as endospore-associated or putative effectors and a known role for one of these proteases, *MEP1*, in mediating host-endospore interactions [40], it is intriguing to hypothesize that these secreted proteases are involved in *Coccidioides* virulence and could be a large part of its effector armamentarium. Secreted proteases often evoke eosinophilia [85], which is a part of *Coccidioides*’ clinical presentation [86]. It is not yet known whether eosinophils are protective during *Coccidioides* infection. However, given their association with Th2-dominant immune responses that are often not protective in fungal infections [87], an intriguing hypothesis is that the numerous secreted proteases in *Coccidioides* bias toward an ineffective immune response. Interrogation of protease function via mutant generation will be key to elucidating their role in *Coccidioides* virulence.

## Supporting information

S1 Fig Early spherulation cultures are synchronous. (A) Micrograph of a spherulation culture demonstrating all four possible morphologies quantified in 1B: spherule, spherule-releasing endospores which remain associated, free endospores that have disassociated from the spherule that released them, and hyphae. (B) Percentage of cells in culture that are rounded (instead of barrel-shaped arthroconidia) 8 h post-placement in spherulation conditions. *n* ≥400 cells, quantified by hand for each sample. Baseline round cells in arthroconidia stock are likely barrel-shaped arthroconidia on end. Underlying data can be found in S1 Table. (C) Average spherule diameter at day 6 for each replicate (microns), measured manually in Fiji for ≥50 spherules per condition. Error bars show standard deviation. Underlying data can be found in S3 Table. (D) Pearson correlation coefficients, quantitatively shown by color, comparing all samples to each other (three replicates at each timepoint). (E) Number of significantly differentially regulated transcripts (2-fold change, FDR 5% using limma) for each comparison of triplicate samples to the previous timepoint. (F) Projection of RNA-Seq datasets for all samples onto principal components 1 and 2. Each replicate for the same timepoint is shown by color. Corresponding ellipse of the same color is oriented on the covariance of the replicates and scaled by three standard deviations.
(TIF)

S2 Fig Wildtype develops into different morphologies while *ryp1∆* is hyphal-locked. (A) Micrographs of fixed samples from each flask at the time of RNA harvest for all replicates of spherule growth. Replicate 1 contains the same images as shown in Fig 2A. Subsequent samples for each replicate were taken from the same flask over time. (B) Average spherule diameter at day 6 for each wildtype replicate in spherule growth (microns), measured manually in Fiji for at least 1,000 spherules per condition. Error bars show standard deviation. Underlying data can be found in S6 Table. (C) Number of significantly differential transcripts (2-fold change, FDR 5% in limma) for each stated comparison in spherule cultures. (D) Micrographs of fixed samples from each flask at the time of RNA harvest for all replicates of hyphal growth. Subsequent samples were taken from the same flask over time. Black arrowheads indicate branching hyphae. Replicate 1 contains the same images as shown in Fig 2D. (E) Number of significantly differential transcripts (2-fold change, FDR 5%) for each stated comparison in hyphal cultures.
(TIF)

S3 Fig *RYP1* induces spherule-related transcripts and represses hyphal-related transcripts. (A) Arthroconidia in Fig 3 were stored for one to two days at 4°C prior to initiating growth of spherules or hyphae, which has been shown to alter the transcriptome [22]. To determine whether storage conditions affected the *ryp1∆* arthroconidia in a different manner than wildtype arthroconidia, we repeated a limited spherulation and hyphal time course with arthroconidia that germinated immediately after harvest. Bar graph showing number of significantly differential transcripts between wildtype and the *ryp1∆* mutant at each timepoint specified. Transcripts that are induced by *RYP1* (higher in WT than *ryp1∆*) are in purple, and transcripts that are repressed by *RYP1* (higher in *ryp1∆* than WT) are in green. The number of *RYP1*-dependent transcripts remained highest in arthroconidia compared to early spherule and hyphal timepoints. (B) Scatterplot of log_2_ of the ratio of wildtype to *ryp1*∆ (counts per million) in arthroconidia from Fig 3A (x-axis) and S3A Fig (y-axis). (C) Scatterplots comparing ratios of log_2_(counts per million) for each transcript. Top row: comparing spherule wildtype/*ryp1∆* to wildtype spherule/wildtype hyphae at each specified corresponding timepoint. Bottom row: comparing hyphal wildtype/*ryp1∆* expression to wildtype spherule/wildtype hyphae at each specified corresponding timepoint. (D) Pearson correlation values from C graphed over time for the top row of C in yellow and the bottom row of C in blue. (E) Expression of the *RYP1* transcript in arthroconidia, all timepoints of spherule development, and all timepoints of hyphal growth, as log_2_(counts per million).
(TIF)

S4 Fig Reproducible Ryp1 binding by ChIP-Seq reveals multiple motifs. (A) Micrographs of fixed samples from each flask at the time of RNA and DNA harvest for all wildtype replicates of spherule and hyphal growth for paired ChIP-Seq/RNA-Seq experiment. Subsequent samples for each replicate were taken from the same flask over time. (B) Micrographs of fixed samples from each flask at the time of RNA and DNA harvest for all replicates of spherule and hyphal growth for the *ryp1∆* mutant from the same experiment as in A. (C) Histogram of the distance (in kilobases) from peak start site to the ATG of the gene to which the peak is assigned. All peaks called in all individual datasets were included except for four peaks that were very wide, resulting in distance from ATG >  15 kb. (D) Traces demonstrating chromosomal location of fold enrichment of ChIP signal/input in spherules (yellow) and hyphae (blue) at the designated timepoints relative to the intergenic region between D8B26_007678 and D8B26_007679. (E) Barplot demonstrating the proportion of morphology-regulated genes (as defined in Fig 3H for Replicate 1, same comparison for RNA-Seq generated from samples in A for Replicate 2) whose promoters have Ryp1 binding by ChIP-Seq. (F) Schematic demonstrating iterative method used to combine Ryp1 binding peaks found by ChIP-Seq, as described in the methods. (G) Previously published Ryp1 binding motif in *Histoplasma* [15]. (H) Histogram of the distance (in kilobases) from motif start sites to the ATG of the neighboring downstream gene, for the motif in Fig 4G. (I) Distribution of the number of Ryp1 motif hits per promoter for the motif in Fig 4G for each subset of genes with Ryp1 motif hits as defined in Fig 4H. (J) As in H, but now describing the location distribution for motif in Fig 4I. (K) As in I but now describing distribution of the number of Ryp1 motif hits per promoter for the motif in Fig 4I. (L) Bar plot of promoter lengths (in base pairs) for each gene subset.
(TIF)

S5 Fig DMEM and RPMI media used to generate spherules. (A) Micrographs of fixed samples from each replicate on day 3 of spherulation in DMEM +  20% FBS. Spherules were generated from the same arthroconidia stock and grown in the same conditions as spherule samples described in Fig 2. Spherules were also harvested at the same time as microscopy samples for RNA-Seq. (B) As in A spherules were generated from three days of growth in RPMI +  10% FBS.
(TIF)

S6 Fig Three genes in the six-gene cluster influence arthroconidia-associated pigment. (A) Quantification of the number of visible cell wall layers for wildtype and each *DitCluster∆* mutant. * : *p* < 0.01, **: *p* < 0.001, by unpaired *t* tes*t*. Underlying data can be found in S14 Table. (B) Schematic of the location of the regions deleted in *DitCluster∆* and *DitClusterSmall∆* mutants. (C) Quantification of arthroconidia cell wall width measurements of TEM images for wildtype and each *DitClusterSmall∆* mutant. * : *p* < 0.05, ***: *p* < 0.0005, by unpaired *t* test. Underlying data can be found in S14 Table. (D) Top: Pictures of tubes holding spore stocks for the indicated genotypes demonstrating their color. Middle: Gaussian blur applied to each picture of the tubes to create a uniform color. Bottom: CMYK color parameters for the center of each Gaussian blur, quantifying the difference in yellow pigment. (E) Hyphal radial growth at 30°C for wildtype and each *DitCluster∆* mutant. Underlying data can be found in S15 Table. (F) Micrographs of fixed samples of spherule development for wildtype and each *DitCluster∆* mutant. Images are representative of the results of two independent experiments. (G) As in E for wildtype and each *DitClusterSmall∆* mutant. Underlying data can be found in S15 Table. (H) As in F for wildtype and each *DitClusterSmall∆* mutant.
(TIF)

S1 Table Rounded morphology counts, related to S1B Fig. Raw data corresponding to the bar graph in S1B Fig. “Rounded” corresponds to round morphologies manually counted for each sample. “Total” corresponds to total number of cells counted for each sample. “Percentage” is percent of total cells that had rounded morphology.
(XLSX)

S2 Table Morphology counts for spherulation cultures, related to Fig 1B. Raw counts for each morphology (defined in S1A Fig) found in individual timepoints and replicates.
(XLSX)

S3 Table Spherule diameter measurements, related to S1C Fig. Individual spherule diameters in microns listed for each biological replicate.
(XLSX)

S4 Table Comma-delimited text file containing transcript abundance over the course of spherulation, related to Fig 1. Each row corresponds to a transcript. The columns are as follows: UNIQID: systemic gene name from Silveira genome [49]. Systematic gene names with _1, _2 appended have multiple isoforms as detected by kallisto although not all isoforms passed read count filter. NAME: short gene name from Mandel and colleagues [12]. Cp_anno: GenBank *Coccidioides posadasii* Silveira annotation. CiRS: Systematic CiRS gene name for the InParanoid-mapped *Coccidioides immitis* RS ortholog. CiRS_anno: Genbank annotation for CiRS ortholog. HcG217B: systematic HcG217B GSC gene name for the InParanoid-mapped *Histoplasma* G217B ortholog. HcG217B_anno: GSC annotation for HcG217B ortholog. The next 13 columns give limma-adjusted *p*-values for differential expression for the listed contrasts. The next 24 columns give kallisto mean row normalized counts for each sample (three replicates per timepoint of spherulation). The last 13 columns give limma-generated log_2_ fold change values for the listed contrasts. Spores refer to arthroconidia.
(CSV)

S5 Table Morphology counts for spherulation cultures, related to Fig 2B. Raw counts for each morphology (defined in S1A Fig) found in individual timepoints and replicates.
(XLSX)

S6 Table Spherule diameter measurements, related to S2B Fig. Individual spherule diameters in microns listed for each biological replicate.
(XLSX)

S7 Table Comma-delimited text file containing transcript abundance over the course of spherulation and hyphal growth for wildtype and *ryp1*∆ , related to Fig 2. Each row corresponds to a transcript. The columns are as follows: UNIQID: systemic gene name from Silveira genome [49]. Systematic gene names with _1, _2 appended have multiple isoforms as detected by kallisto although not all isoforms passed read count filter. NAME: short gene name from Mandel and colleagues [12]. Cp_anno: GenBank *Coccidioides posadasii* Silveira annotation. CiRS: systematic CiRS gene name for the InParanoid-mapped *Coccidioides immitis* RS ortholog. CiRS_anno: Genbank annotation for CiRS ortholog. HcG217B: systematic HcG217B GSC gene name for the InParanoid-mapped *Histoplasma* G217B ortholog. HcG217B_anno: GSC annotation for HcG217B ortholog. The next 57 columns give limma-adjusted *p*-values for differential expression for the listed contrasts. The next 84 columns give kallisto mean row normalized counts for each sample (three replicates per timepoint of spherulation or hyphal growth for the wildtype and *ryp1∆* mutant each). The last 57 columns give limma-generated log_2_ fold change values for the listed contrasts. For sample and comparison labels: Spores refer to arthroconidia, “Sil” refers to the Silveira wildtype, Ryp1 refers to the *ryp1∆* mutant, “spherule”/“myc” indicate spherule and hyphal morphology, and “eighth” refers to the 8 h timepoint of either spherulation or hyphal growth.
(CSV)

S8 Table Number of genes with reproducible Ryp1 binding in their promoter. Rows correspond to sample type where WT is wildtype and Ryp1 is the *ryp1∆* mutant at the listed timepoints in either spherule development (“Spher”), hyphal development (“Hyph”), or arthroconidia (“Arth”). The left side of the table lists the number of peaks found by macs2 per each replicate for each sample type. The right side of the table shows the number of genes that had at least 1 Ryp1 binding peak located within their promoters and then a tally of the number of genes whose promoters contained at least 1 Ryp1 binding peak in two of the three total replicates. Samples highlighted in yellow were those for which we continued downstream analysis.
(XLSX)

S9 Table Comma-delimited text file containing transcript abundance over the course of spherulation and hyphal growth from the same samples used for Ryp1 ChIP-Seq, related to S4 Fig. Each row corresponds to a transcript. The columns are as follows: UNIQID: systemic gene name from Silveira genome [49]. Systematic gene names with _1, _2 appended have multiple isoforms as detected by kallisto although not all isoforms passed read count filter. NAME: short gene name from Mandel and colleagues [12]. Cp_anno: GenBank *Coccidioides posadasii* Silveira annotation. CiRS: systematic CiRS gene name for the InParanoid-mapped *Coccidioides immitis* RS ortholog. CiRS_anno: Genbank annotation for CiRS ortholog. HcG217B: systematic HcG217B GSC gene name for the InParanoid-mapped *Histoplasma* G217B ortholog. HcG217B_anno: GSC annotation for HcG217B ortholog. The next 17 columns give limma-adjusted *p*-values for differential expression for the listed contrasts. The next 36 columns are kallisto mean row normalized counts for each sample (three replicates per timepoint of spherulation or hyphal growth for the wildtype and *ryp1∆* mutant each). The last 17 columns give limma-generated log_2_ fold change values for the listed contrasts. For sample and comparison labels: “Arth” refers to arthroconidia, “WT” refers to the Silveira wildtype, Ryp1 refers to the *ryp1∆* mutant, “spher”/”hyph” indicates spherule and hyphal morphology.
(CSV)

S10 Table *Coccidioides* candidate transcription factors (TFs). Each row corresponds to a predicted TF. The columns are as follows: UNIQID: systemic gene name from Silveira genome [49]. NAME: short gene name from Mandel and colleagues [12]. CiRS: systematic CiRS gene name for the InParanoid-mapped *Coccidioides immitis* RS ortholog. HcG217B: systematic HcG217B GSC gene name for the InParanoid-mapped *Histoplasma* G217B ortholog. TF_domains: The Pfam TF domains are found in each TF candidate. The next 28 columns are kallisto mean row normalized average counts for three replicates corresponding with each RNA-Seq sample (same data as S7 Table).
(CSV)

S11 Table Candidate endospore-related genes in *Coccidioides.* Each row corresponds to a predicted transcription factor. The columns are as follows: UNIQID: systemic gene name from Silveira genome [49]. Systematic gene names with _1, _2 appended have multiple isoforms as detected by kallisto although not all isoforms passed read count filter. NAME: short gene name from Mandel and colleagues [12]. Cp_anno: GenBank *Coccidioides posadasii* Silveira annotation. CiRS: systematic CiRS gene name for the InParanoid-mapped *Coccidioides immitis* RS ortholog. CiRS_anno: Genbank annotation for CiRS ortholog. HcG217B: systematic HcG217B GSC gene name for the InParanoid-mapped *Histoplasma* G217B ortholog. HcG217B_anno: GSC annotation for HcG217B ortholog. The next 69 columns are kallisto mean row normalized counts for each indicated sample. Samples are indicated to be from data presented in Figs 1, 2 or S5. Sample names are defined in S4 Table for data from Fig 1 and S7 Table for data from Fig 2.
(CSV)

S12 Table Candidate virulence effectors in *Coccidioides.* Each row corresponds to a predicted effector. The columns are as follows: UNIQID: systemic gene name from Silveira genome [49]. Systematic gene names with _1, _2 appended have multiple isoforms as detected by kallisto although not all isoforms passed read count filter. NAME: short gene name from Mandel and colleagues [12]. Cp_anno: GenBank *Coccidioides posadasii* Silveira annotation. CiRS: systematic CiRS gene name for the InParanoid-mapped *Coccidioides immitis* RS ortholog. CiRS_anno: Genbank annotation for CiRS ortholog. HcG217B: systematic HcG217B GSC gene name for the InParanoid-mapped *Histoplasma* G217B ortholog. HcG217B_anno: GSC annotation for HcG217B ortholog. The next 45 columns are kallisto mean row normalized counts for each indicated sample. Sample names are from S7 Table.
(CSV)

S13 Table *RYP1* regulation of dityrosine cluster transcripts. Each row represents a transcript from the six-gene dityrosine cluster. Each column indicates a timepoint of spherulation/hyphal growth. At each timepoint, expression of the transcript was compared between WT and *ryp1∆*. “N” means no significant difference between the level of the transcript in WT compared to the *ryp1∆* mutant at that timepoint. “−” means the transcript level in *ryp1∆* is significantly higher than in WT. “+” means the transcript level in WT is significantly higher than in the *ryp1∆* mutant.
(XLSX)

S14 Table *DitCluster*∆ and *DitClusterSmall*∆ Cell Wall measurements, related to Fig 6 and S6. The tabs labeled “DitCluster Cell Wall Width” and DitClusterSmall Cell Wall Width’ contain raw measurements for each arthroconidia cell wall measured for each sample (in pixels), for the *DitCluster∆* mutant and *DitClusterSmall∆* mutant, respectively. The tab “DitCluster Cell Wall Layers” lists the number of distinct cell wall layers observed for each arthroconidia for each sample quantified.
(XLSX)

S15 Table *DitCluster∆* and *DitClusterSmall∆* radial growth measurements, related to S6E and S6G Fig. Each tab lists the raw measurements (in pixels) of colony radius on the stated days for three colonies each for the stated genotypes.
(XLSX)

S16 Table Sequences of reagents used for genetic manipulation of *Coccidioides.* Primer sequences are listed in the first 19 rows and were ordered from IDT. Crispr Alt-R crRNA refers to the protospacer sequence used to order CRISPR-Cas9 crRNAs from IDT. Repair cassette sequences indicate the full DNA molecule synthesized by Azenta and used as the template for homologous repair during transformation of *Coccidioides*.
(XLSX)

S17 Table RNA-Seq sample and sequencing information. Each sequenced library is listed in a row by library name, with corresponding data as indicated by headers. WT is wildtype genotype, Ryp1 is *ryp1∆* mutant genotype. “# Reads pseudoaligned to unspliced transcript” are the counts generated by kallisto against unspliced mRNA sequences whereas “# Reads pseudoaligned to spliced transcript” are the counts generated by kallisto against the predicted spliced mRNA sequences (as used in all analyses described in the manuscript).
(XLSX)

S1 Genome *ryp1∆* genome assembled by PILON. PILON was used to generate a reference-guided assembly of the *ryp1*∆ mutant genome from paired-end Illumina reads and the GCA_018416015.2 *Coccidioides posadasii* reference. BWA was used to generate alignments and those alignments were used as input to PILON in variant mode. This procedure was repeated for 15 iterations and was able to fully assemble the hygromycin cassette insertion that replaced *RYP1*. The full assembly fastq is presented here.
(FASTA)

S1 Code Folder containing README document describing the scripts used to analyze the data and generate figures in this manuscript, as well as the scripts themselves and custom python three modules used in the scripts.
(ZIP)

## Abbreviations

IP: immunoprecipitation
RT: room temperature
TEM: transmission electron microscopy
TF: transcription factor
