# Supplementary figures and images for: Transcriptomic atlas throughout Coccidioides development reveals key phase-enriched transcripts of this important fungal pathogen
Source: PLoS Biol. 2025 Apr 15;23(4):e3003066. doi: 10.1371/journal.pbio.3003066 (PMC12077801; doi:10.1371/journal.pbio.3003066)

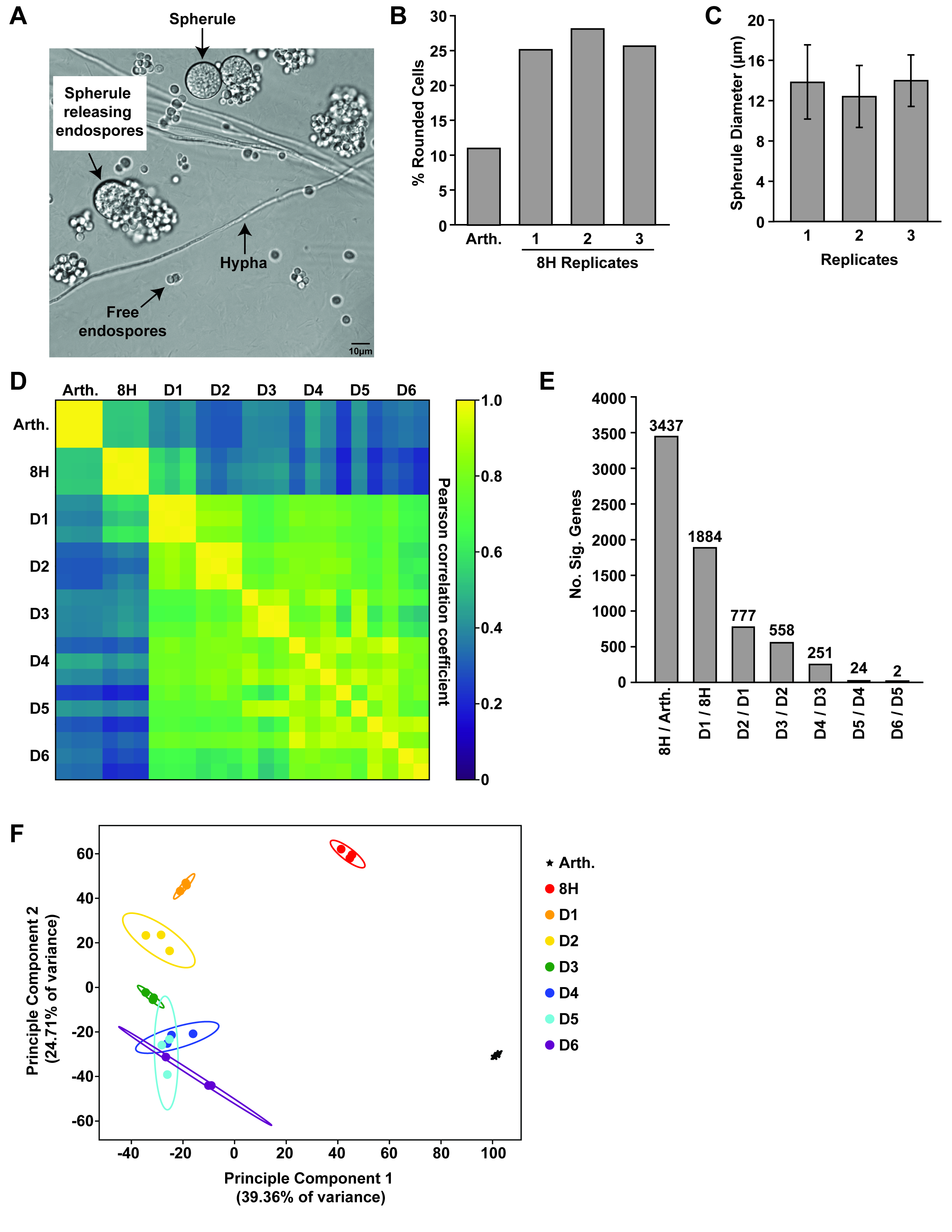

Supplement: S1 Fig — (A) Micrograph of a spherulation culture demonstrating all four possible morphologies quantified in 1B: spherule, spherule-releasing endospores which remain associated, free endospores that have disassociated from the spherule that released them, and hyphae. (B) Percentage of cells in culture that are rounded (instead of barrel-shaped arthroconidia) 8 h post-placement in spherulation conditions. n ≥400 cells, quantified by hand for each sample. Baseline round cells in arthroconidia stock are likely barrel-shaped arthroconidia on end. Underlying data can be found in S1 Table. (C) Average spherule diameter at day 6 for each replicate (microns), measured manually in Fiji for ≥50 spherules per condition. Error bars show standard deviation. Underlying data can be found in S3 Table. (D) Pearson correlation coefficients, quantitatively shown by color, comparing all samples to each other (three replicates at each timepoint). (E) Number of significantly differentially regulated transcripts (2-fold change, FDR 5% using limma) for each comparison of triplicate samples to the previous timepoint. (F) Projection of RNA-Seq datasets for all samples onto principal components 1 and 2. Each replicate for the same timepoint is shown by color. Corresponding ellipse of the same color is oriented on the covariance of the replicates and scaled by three standard deviations. (TIF) [file pbio.3003066.s001.tif]

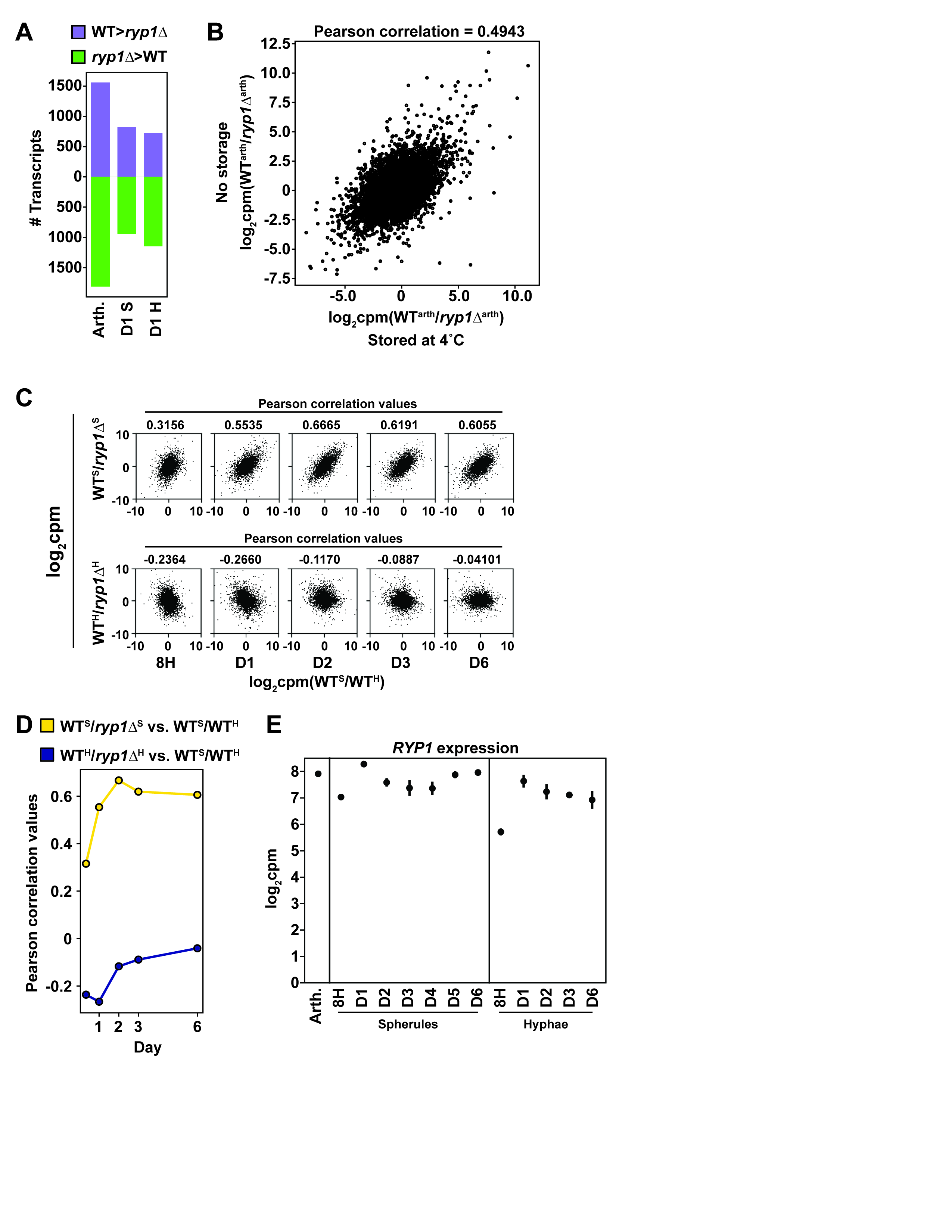

Supplement: S3 Fig — (A) Arthroconidia in Fig 3 were stored for one to two days at 4°C prior to initiating growth of spherules or hyphae, which has been shown to alter the transcriptome [22]. To determine whether storage conditions affected the ryp1∆ arthroconidia in a different manner than wildtype arthroconidia, we repeated a limited spherulation and hyphal time course with arthroconidia that germinated immediately after harvest. Bar graph showing number of significantly differential transcripts between wildtype and the ryp1∆ mutant at each timepoint specified. Transcripts that are induced by RYP1 (higher in WT than ryp1∆) are in purple, and transcripts that are repressed by RYP1 (higher in ryp1∆ than WT) are in green. The number of RYP1-dependent transcripts remained highest in arthroconidia compared to early spherule and hyphal timepoints. (B) Scatterplot of log2 of the ratio of wildtype to ryp1∆ (counts per million) in arthroconidia from Fig 3A (x-axis) and S3A Fig (y-axis). (C) Scatterplots comparing ratios of log2(counts per million) for each transcript. Top row: comparing spherule wildtype/ryp1∆ to wildtype spherule/wildtype hyphae at each specified corresponding timepoint. Bottom row: comparing hyphal wildtype/ryp1∆ expression to wildtype spherule/wildtype hyphae at each specified corresponding timepoint. (D) Pearson correlation values from C graphed over time for the top row of C in yellow and the bottom row of C in blue. (E) Expression of the RYP1 transcript in arthroconidia, all timepoints of spherule development, and all timepoints of hyphal growth, as log2(counts per million). (TIF) [file pbio.3003066.s003.tif]

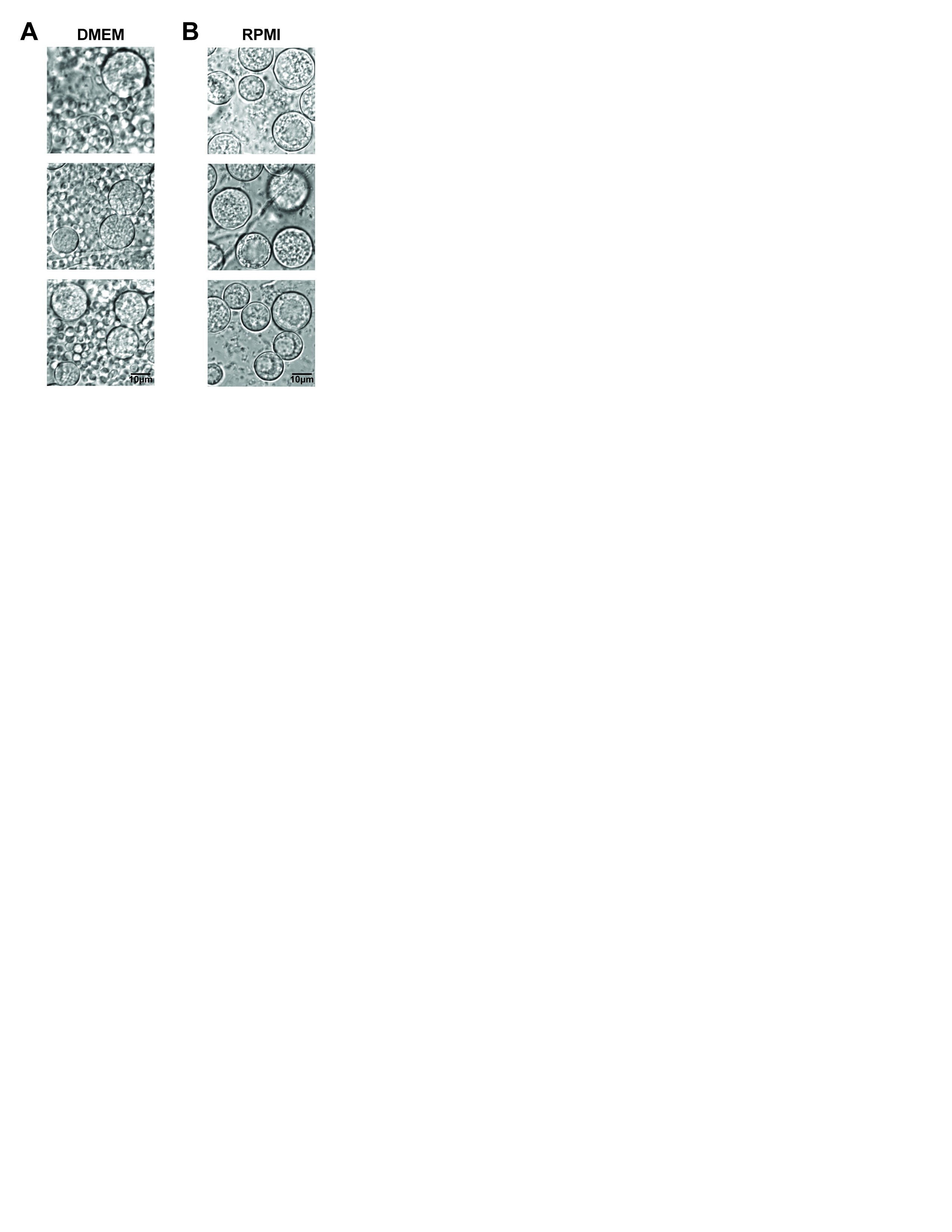

Supplement: S5 Fig — (A) Micrographs of fixed samples from each replicate on day 3 of spherulation in DMEM + 20% FBS. Spherules were generated from the same arthroconidia stock and grown in the same conditions as spherule samples described in Fig 2. Spherules were also harvested at the same time as microscopy samples for RNA-Seq. (B) As in A spherules were generated from three days of growth in RPMI + 10% FBS. (TIF) [file pbio.3003066.s005.tif]

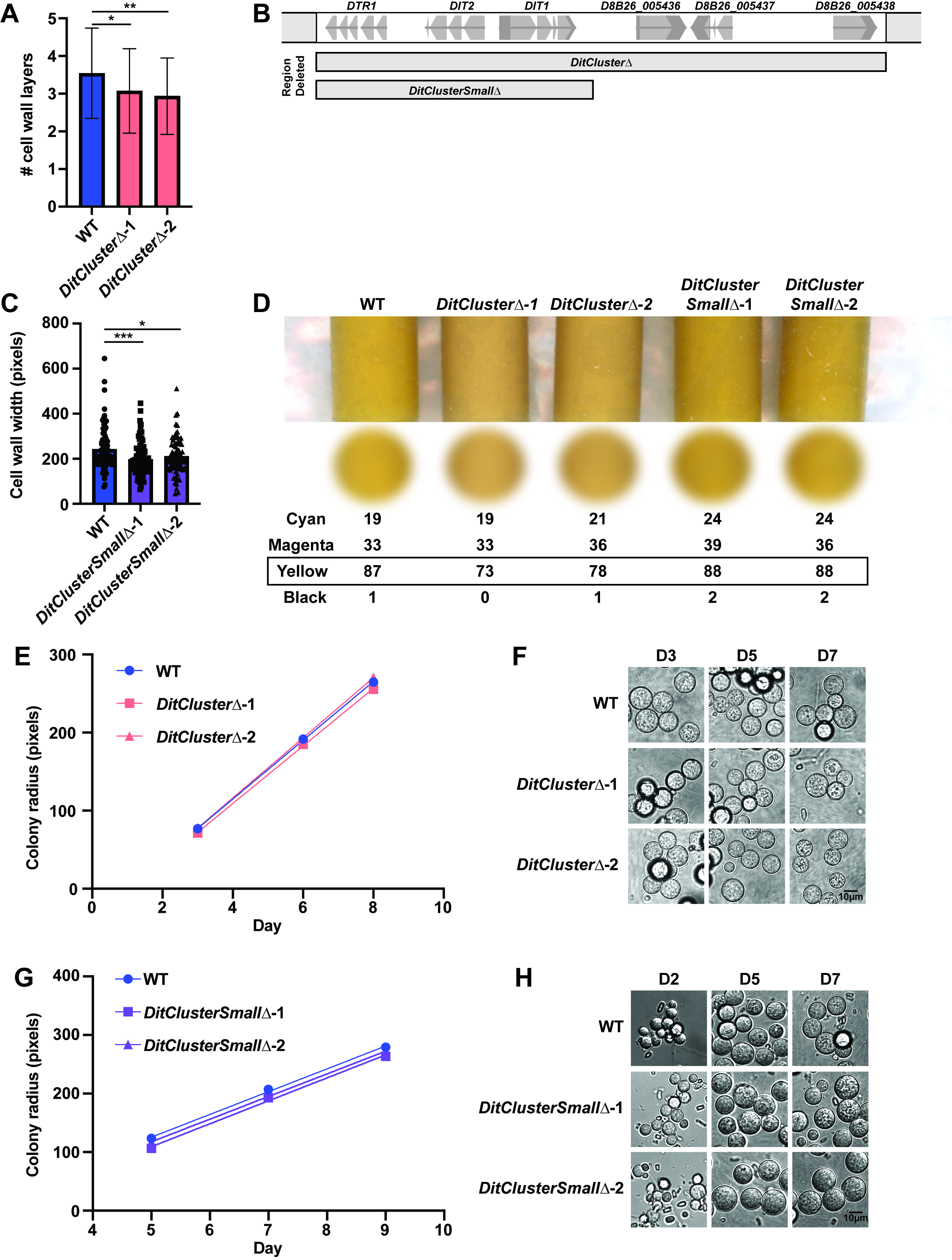

Supplement: S6 Fig — (A) Quantification of the number of visible cell wall layers for wildtype and each DitCluster∆ mutant. * : p < 0.01, **: p < 0.001, by unpaired t test. Underlying data can be found in S14 Table. (B) Schematic of the location of the regions deleted in DitCluster∆ and DitClusterSmall∆ mutants. (C) Quantification of arthroconidia cell wall width measurements of TEM images for wildtype and each DitClusterSmall∆ mutant. * : p < 0.05, ***: p < 0.0005, by unpaired t test. Underlying data can be found in S14 Table. (D) Top: Pictures of tubes holding spore stocks for the indicated genotypes demonstrating their color. Middle: Gaussian blur applied to each picture of the tubes to create a uniform color. Bottom: CMYK color parameters for the center of each Gaussian blur, quantifying the difference in yellow pigment. (E) Hyphal radial growth at 30°C for wildtype and each DitCluster∆ mutant. Underlying data can be found in S15 Table. (F) Micrographs of fixed samples of spherule development for wildtype and each DitCluster∆ mutant. Images are representative of the results of two independent experiments. (G) As in E for wildtype and each DitClusterSmall∆ mutant. Underlying data can be found in S15 Table. (H) As in F for wildtype and each DitClusterSmall∆ mutant. (TIF) [file pbio.3003066.s006.tif]
