# Supplementary material for: Transcriptomic atlas throughout Coccidioides development reveals key phase-enriched transcripts of this important fungal pathogen
Source: PLoS Biol. 2025 Apr 15;23(4):e3003066. doi: 10.1371/journal.pbio.3003066 (PMC12077801; doi:10.1371/journal.pbio.3003066)
Supplement: S1 Code — Folder containing README document describing the scripts used to analyze the data and generate figures in this manuscript, as well as the scripts themselves and custom python three modules used in the scripts. (ZIP) [file pbio.3003066.s025.zip › Custom Code/notebooks/Fig4_and_S4_without_motif_finding_and_6C.html]

Fig4\_and\_S4\_without\_motif\_finding\_and\_6C


In [1]:

```
import matplotlib.pyplot as plt
%matplotlib nbagg
```

In [2]:

```
%cd ../../Papers/Cocci_transcriptomics/data_for_code/Fig4/
```

```
/home/chomer/Papers/Cocci_transcriptomics/data_for_code/Fig4
```

In [3]:

```
from MySQLGenomeFactory import MySQLGenomeFactory
import h5py
from MacsTools import parse_MACS_xls
from glob import glob
from ConsensusSearch import intergenic_promoters
import numpy as np
from Collisions import RefCollisions
from CdtFile import CdtFile, CdtRow
from CdtAnnotator3 import annotate_CpSilveira as annotate
from matplotlib_venn import venn3, venn3_circles
import upsetplot
from HistoPlot2.browsers import Universal_Binding_browser
```

In [4]:

```
f = MySQLGenomeFactory(db="Genome4")
genome = f.getGenome("CpSilveiraV3")
```

In [5]:

```
hdf = h5py.File("Fig4_coverage.hdf5", "r")
```

In [6]:

```
sample2peaks = dict((i.replace("_peaks.xls",""),parse_MACS_xls(i,genome)) 
                    for i in glob("*.xls") if "NA" not in i)
```

# Assign peaks to individual promoters¶

In [7]:

```
intergenics = intergenic_promoters(genome, from_ATG=True)
len(intergenics), len(set((i.Locus().ref,i.Locus().start,i.Locus().stop) for i in intergenics))
#max size is 10kb
#first number is all intergenics, second is intergenics without convergent 3' intergenics
```

Out[7]:

```
(8269, 6011)
```

In [8]:

```
lengths = []
for intergenic in intergenics:
    length = abs(intergenic.Locus().stop - intergenic.Locus().start)
    lengths.append(length)
print(max(lengths), min(lengths), np.median(lengths))
```

```
9999 2 965.0
```

In [9]:

```
#Used to generate Table S10
#Make a dictionary for all peaks that are assigned to a particular gene's intergenic region
peaks_assigned_to_genes = {}
for sample in sample2peaks.keys():
    sample_prefix = "_".join(sample.split("_")[:-1])
    rep = int(sample.split("_")[-1]) - 1
    peaks = sample2peaks[sample]
    peaks_assigned_to_genes_list = RefCollisions(peaks, intergenics)
    if sample_prefix not in peaks_assigned_to_genes: 
        peaks_assigned_to_genes[sample_prefix] = [[],[],[]]
    peaks_assigned_to_genes[sample_prefix][rep] = peaks_assigned_to_genes_list
```

In [10]:

```
#Used to generate Table S10
#Make a list of genes with peaks in their promoters for 2/3 replicates
genes_with_combined_peaks = {}
for sample_prefix in peaks_assigned_to_genes.keys():
    peaks_list = peaks_assigned_to_genes[sample_prefix]
    genes_with_combined_peaks[sample_prefix] = []
    for gene in genome.Genes():
        gene = gene[0]
        list_count = 0
        gene_with_peak = False
        for peak in peaks_list[0]: 
            gene1 = peak[1].Gene().Name()
            if gene == gene1:
                gene_with_peak = True
        if gene_with_peak == True:
            list_count += 1
        gene_with_peak = False    
        for peak2 in peaks_list[1]:
            gene2 = peak2[1].Gene().Name()
            if gene == gene2:
                gene_with_peak = True
        if gene_with_peak == True:
            list_count += 1
        gene_with_peak = False 
        for peak3 in peaks_list[2]:
            gene3 = peak3[1].Gene().Name()
            if gene == gene3:
                gene_with_peak = True
        if gene_with_peak == True:
            list_count += 1        
        if list_count > 3:
            print(gene)
        if list_count >=2:
            genes_with_combined_peaks[sample_prefix].append(gene)
```

# Will focus analysis on these datasets that have a reproducibly high number of promoters bound¶

In [11]:

```
key_datasets = ["WT_D1_Spher", "WT_D2_Spher", "WT_D4_Spher","WT_D2_Hyph", "WT_D4_Hyph"]
```

In [12]:

```
#let's make a heatmap of all the peaks that are found in WT samples but not the morphology-paired Ryp1 dataset
ryp1_subtracted_peaks = {}
for sample in genes_with_combined_peaks:
    tp =  "_".join(sample.split("_")[:-1])
    morph = sample.split("_")[-1]
    if morph == "Spher" and "Ryp1" not in tp:
        paired_ryp1_sample = "Ryp1_D1_Spher"
    elif morph == "Hyph" and "Ryp1" not in tp:
        paired_ryp1_sample = "Ryp1_D1_Hyph"
    elif morph == "Arth" and "Ryp1" not in tp:
        paired_ryp1_sample = "Ryp1_Arth"
    else:
        paired_ryp1_sample = sample
    for gene in genes_with_combined_peaks[sample]:
        if gene not in genes_with_combined_peaks[paired_ryp1_sample]:
            if sample not in ryp1_subtracted_peaks:
                ryp1_subtracted_peaks[sample] =[gene]
            else:
                ryp1_subtracted_peaks[sample].append(gene)
```

In [13]:

```
#create a heatmap with all the chip-seq peaks
chip_peaks = {}
for sample in ryp1_subtracted_peaks:
    peaks = ryp1_subtracted_peaks[sample]
    for k,prefix in enumerate(key_datasets):
        if prefix == sample:
            index = k
    for peak in peaks:
        if peak in chip_peaks:
            ratios = chip_peaks[peak]
            ratios[index] = 1
            chip_peaks[peak] = ratios
        else:
            ratios = []
            for i in range(0,len(key_datasets)):
                if i == index:
                    ratios.append(1)
                else:
                    ratios.append(0)
            chip_peaks[peak] = ratios
       
chipseq_cdt = CdtFile(probes = [CdtRow(gid = sample, uniqid = sample, name=sample, ratios = chip_peaks[sample])
                                             for sample in chip_peaks],
                     fieldnames = key_datasets,
                     eweights = [1]*len(key_datasets))
chipseq_cdt = annotate(chipseq_cdt, warn=False, map_from="V3")

count_cols = [n for (n,i) in enumerate(chipseq_cdt.fieldnames) if( "WT" in i)]

tree = chipseq_cdt.cluster(cols=count_cols,dist="u",method="m")
chipseq_cdt.writeCdtGtr("chipseq_peaks.um", tree)
```

```
Warning, didn't find a GID column!  Sharing UID with GID.
Building array...
Building distance matrix...
Clustering...
```

In [14]:

```
#Fig 4E
Spher_D1 = set(genes_with_combined_peaks["WT_D1_Spher"])
Spher_D2 = set(genes_with_combined_peaks["WT_D2_Spher"])
Spher_D4 = set(genes_with_combined_peaks["WT_D4_Spher"])
Hyph_D2 = set(genes_with_combined_peaks["WT_D2_Hyph"])
Hyph_D4 = set(genes_with_combined_peaks["WT_D4_Hyph"])

dataset = upsetplot.from_contents({"Spher D1": Spher_D1, "Spher D2": Spher_D2, "Spher D4": Spher_D4, "Hyph D2":Hyph_D2, "Hyph D4":Hyph_D4, })
ax = upsetplot.plot(dataset, element_size=None, sort_categories_by='-input')
plt.savefig("chip_upsetplot.png")
```

# Generating list of peaks that are in spherule samples at a given timepoint but not in hyphal samples at that same timepoint¶

In [15]:

```
spherule_specific_genes = {}
for sample in genes_with_combined_peaks.keys():
    tp =  "_".join(sample.split("_")[:-1])
    morph = sample.split("_")[-1]
    if morph == "Spher" and "Ryp1" not in tp:
        paired_wt_sample = "{0}_Hyph".format(tp)
        paired_ryp1_sample = "Ryp1_D1_Spher"
        for gene in genes_with_combined_peaks[sample]:
            if gene not in genes_with_combined_peaks[paired_wt_sample]:
                if gene not in genes_with_combined_peaks[paired_ryp1_sample]:
                    if tp not in spherule_specific_genes:
                        spherule_specific_genes[tp] =[gene]
                    else:
                        spherule_specific_genes[tp].append(gene)
```

In [16]:

```
#Basis for Figure 4F

WT_D1_set = set(spherule_specific_genes["WT_D1"])
WT_D2_set = set(spherule_specific_genes["WT_D2"])
WT_D4_set = set(spherule_specific_genes["WT_D4"])

fig = plt.figure()
venn=venn3([WT_D1_set,WT_D2_set,WT_D4_set], ("WT_D1", "WT_D2", "WT_D4"))
plt.savefig("spherule_specific_peaks_venn_diagram.png")
```

# Looking at overlap between peaks and RNA-seq defined gene subsets¶

In [17]:

```
ryp1_spherule_combined_rna = CdtFile.fromCdt("../Fig2/Combined/limma1_ryp1dep_spherule.contrasts_um.cdt")
len(ryp1_spherule_combined_rna)
```

Out[17]:

```
452
```

In [18]:

```
ryp1_hyph_combined_rna = CdtFile.fromCdt("../Fig2/Combined/limma1_ryp1dep_mycelia.contrasts_um.cdt")
len(ryp1_hyph_combined_rna)
```

Out[18]:

```
262
```

In [19]:

```
#make a list of consistently ryp1-dependent in spherules and directly bound
dss = ["WT_D1_Spher", "WT_8h_Spher", "WT_D4_Spher", "WT_D2_Spher"]

count = 0
for row in ryp1_spherule_combined_rna:
    matched = False
    gene = row.uniqid
    for ds in dss:
        peaks = ryp1_subtracted_peaks[ds]
        if gene in peaks:
            matched = True
    if matched == True:
        count += 1
print("bound genes: {0}".format(count))
print("% bound: {0}".format(float(count)/len(ryp1_spherule_combined_rna)))
```

```
bound genes: 272
% bound: 0.6017699115044248
```

In [20]:

```
#make a list of consistently ryp1-dependent in hyphae and directly bound
dss = ["WT_D1_Hyph", "WT_8h_Hyph", "WT_D4_Hyph", "WT_D2_Hyph"]
count = 0
for row in ryp1_hyph_combined_rna:
    matched = False
    gene = row.uniqid
    for ds in dss:
        peaks = ryp1_subtracted_peaks[ds]
        if gene in peaks:
            matched = True
    if matched == True:
        count += 1
print("bound genes: {0}".format(count))
print("% bound: {0}".format(float(count)/len(ryp1_hyph_combined_rna)))
```

```
bound genes: 71
% bound: 0.27099236641221375
```

In [21]:

```
#Figure 4D
xs = ["spher", "hyph"]
rna_targets = [452,262]
bound = [272,71]
fig = plt.figure()
ax = plt.subplot(111)
barlist = ax.bar(xs,rna_targets, width=1, color='yellow', edgecolor='black')
barlist[1].set_facecolor('lightblue')
barlist1 = ax.bar(xs,bound, width=1, color='gold', edgecolor='black',hatch='//')
barlist1[1].set_facecolor('blue')
fig.savefig("morphology_ryp1_dependence_bound_relationship.svg")
```

# Look at distance of peaks from ATG¶

In [22]:

```
peak_distance_to_ATG = []
for prefix in peaks_assigned_to_genes:
    for rep in [0,1,2]: 
        peaks = peaks_assigned_to_genes[prefix][rep]
        for peak in peaks:
            peak_start = peak[0].Locus().start
            peak_end = peak[0].Locus().stop
            peak_strand = peak[0].Locus().strand
            gene = peak[1].gene
            gene_strand = gene.Locus().strand
            coords = []
            for cds in gene.CdsLoci():
                coords.append(cds.start)
                coords.append(cds.stop)
            if gene_strand == "+":
                atg = min(coords)
                distance_to_ATG = atg - peak_start
            elif gene_strand == "-":
                atg = max(coords)
                distance_to_ATG = peak_end - atg
            if distance_to_ATG > 15000:
                print(distance_to_ATG)
            elif distance_to_ATG <15001:
                peak_distance_to_ATG.append(distance_to_ATG)
```

```
54885
16712
40189
54896
```

In [23]:

```
# Figure S4C
fig = plt.figure()
ax = plt.subplot(111)
plt.hist(peak_distance_to_ATG, color='grey')
plt.savefig("peak_distance_to_ATG.svg")
```

# Genome coordinate plots of ChIP-Seq data¶

In [24]:

```
#used for between-dataset normalization
ste2_locus = genome.getGene("D8B26_006567").Locus()
```

In [25]:

```
chip_data_paper = [
 
 ['chip', "Fig4_coverage.hdf5",
 [['gold',
  [('WT_D1_Spher_1_S13', 'WT_D1_Spher_WCE_1_S31'),
  ('WT_D1_Spher_2_S14','WT_D1_Spher_WCE_2_S32'),
  ('WT_D1_Spher_3_S15','WT_D1_Spher_WCE_3_S33')]],
  ['blue',
    [('WT_D1_Hyph_1_S16', 'WT_D1_Hyph_WCE_1_S34'), 
     ('WT_D1_Hyph_2_S17','WT_D1_Hyph_WCE_2_S35'),
     ('WT_D1_Hyph_3_S18','WT_D1_Hyph_WCE_3_S36')]]],
  []],
['chip', "Fig4_coverage.hdf5",
[['gold',
 [('WT_D2_Spher_1_S43', 'WT_D2_Spher_WCE_1_S61'),
  ('WT_D2_Spher_2_S44','WT_D2_Spher_WCE_2_S62'),
  ('WT_D2_Spher_3_S45','WT_D2_Spher_WCE_3_S63')]],
 ['blue', 
 [('WT_D2_Hyph_1_S46', 'WT_D2_Hyph_WCE_1_S64'),
  ('WT_D2_Hyph_2_S47','WT_D2_Hyph_WCE_2_S65'),
  ('WT_D2_Hyph_3_S48','WT_D2_Hyph_WCE_3_S66')]]],
 []],
 ['chip', "Fig4_coverage.hdf5", 
 [['gold', 
  [('WT_D4_Spher_1_S49', 'WT_D4_Spher_WCE_1_S67'),
  ('WT_D4_Spher_2_S50','WT_D4_Spher_WCE_2_S68'),
  ('WT_D4_Spher_3_S51','WT_D4_Spher_WCE_3_S69')]],
 ['blue',
 [('WT_D4_Hyph_1_S52', 'WT_D4_Hyph_WCE_1_S70'),
 ('WT_D4_Hyph_2_S53','WT_D4_Hyph_WCE_2_S71'),
 ('WT_D4_Hyph_3_S54','WT_D4_Hyph_WCE_3_S72')]]],
  []]


 ]
```

In [26]:

```
browser_paper = Universal_Binding_browser(
    data = chip_data_paper, target = "CpSilveiraV3", control_locus = ste2_locus, average=False
)
```

### SOWgp¶

In [27]:

```
#Figure 4A
gene = "D8B26_003939"
view = browser_paper(gene)
view.fig.savefig("Sowgp_paper.eps", format='eps')
```

### Ryp1¶

In [28]:

```
#Figure 4B
view = browser_paper("D8B26_000722") #ryp1
view.fig.savefig("Ryp1_paper.eps", format='eps')
```

### Shared hyphal/spherule binding cluster¶

In [29]:

```
#Figure 4C
start = genome.getGene("D8B26_005359").Locus().start
end = genome.getGene("D8B26_005361").Locus().stop
view = browser_paper("CP075070:{0}..{1} (+)".format(start+1000,end-1000))
view.fig.savefig("HyphalCluster_paper.eps", format='eps')
```

### 10kb promoter with chip binding signal¶

In [30]:

```
browserzoom_paper = Universal_Binding_browser(
    data = chip_data_paper, target = "CpSilveiraV3", control_locus = ste2_locus, average=False, pad=200
)
```

In [31]:

```
#Figure S4D
start = genome.getGene("D8B26_007678").Locus().start
end = genome.getGene("D8B26_007678").Locus().stop
chr =(genome.getGene("D8B26_007678").Locus().ref)
view = browserzoom_paper("{2}:{0}..{1} (+)".format(start,end+11000, chr))
view.fig.savefig("D8B26_007678_paper.eps", format='eps')
```

### Ryp1 binding in arthroconidia-associated dityrosine cluster¶

In [32]:

```
#Figure 6C is second zoomed out image
view = browser_paper("D8B26_005437")
view = browser_paper(view.Locus().pad(2500))
view.fig.savefig("D8B26_005436to5438_paper.eps", format='eps')
```

In [ ]:

```

```
