# Supplementary material for: Transcriptomic atlas throughout Coccidioides development reveals key phase-enriched transcripts of this important fungal pathogen
Source: PLoS Biol. 2025 Apr 15;23(4):e3003066. doi: 10.1371/journal.pbio.3003066 (PMC12077801; doi:10.1371/journal.pbio.3003066)
Supplement: S1 Code — Folder containing README document describing the scripts used to analyze the data and generate figures in this manuscript, as well as the scripts themselves and custom python three modules used in the scripts. (ZIP) [file pbio.3003066.s025.zip › Custom Code/notebooks/FigS3A_and_S4E.html]

FigS3A\_and\_S4E


In [1]:

```
cd ../../Papers/Cocci_transcriptomics/data_for_code/FigS4/
```

```
/home/chomer/Papers/Cocci_transcriptomics/data_for_code/FigS4
```

In [2]:

```
%load_ext rpy2.ipython
from glob import glob
from MsvUtil import Table
import os.path
from CdtFile import CdtFile, CdtRow
from SafeMath import safelog
from ReadCountTools import PseudoCPMs
from PCA import PCA
import numpy as np
import matplotlib.patches as patches
import matplotlib.transforms as transforms
import matplotlib.pyplot as plt
%matplotlib nbagg
from csv import writer, excel_tab
from LimmaTools import SingleFactorFit
```

In [3]:

```
%%R
library(limma)
library(edgeR)
```

# Merge Kallisto TPMs¶

In [4]:

```
#First Index datasets

sname2kallisto = dict(
    ("_".join(i.replace(".Silv_nanopore_mRNA.rf.kallisto","").split("_")[:-1]),i)
        for i in glob("*.Silv_nanopore_mRNA.rf.kallisto") )
snames = sorted(sname2kallisto, key = lambda x: (x[4:x.rfind("_")],x[:4]))

print(len(sname2kallisto))
print(snames)
```

```
36
['WT_D1_Hyph_1', 'WT_D1_Hyph_3', 'WT_D1_Hyph_2', 'WT_D1_Spher_2', 'WT_D1_Spher_1', 'WT_D1_Spher_3', 'WT_D2_Hyph_2', 'WT_D2_Hyph_3', 'WT_D2_Hyph_1', 'WT_D2_Spher_3', 'WT_D2_Spher_1', 'WT_D2_Spher_2', 'WT_D4_Hyph_1', 'WT_D4_Hyph_2', 'WT_D4_Hyph_3', 'WT_D4_Spher_1', 'WT_D4_Spher_3', 'WT_D4_Spher_2', 'Ryp1_Arth_3', 'Ryp1_Arth_1', 'Ryp1_Arth_2', 'Ryp1_D1_Hyph_3', 'Ryp1_D1_Hyph_2', 'Ryp1_D1_Hyph_1', 'Ryp1_D1_Spher_2', 'Ryp1_D1_Spher_1', 'Ryp1_D1_Spher_3', 'WT_8h_Hyph_3', 'WT_8h_Hyph_1', 'WT_8h_Hyph_2', 'WT_8h_Spher_3', 'WT_8h_Spher_1', 'WT_8h_Spher_2', 'WT_Arth_2', 'WT_Arth_3', 'WT_Arth_1']
```

In [5]:

```
#Merge Step
genes = None
cols = []
counts = []
for i in snames:
    table = Table.fromTdt(open(os.path.join(
           sname2kallisto[i],
          "abundance.tsv")))
    if(genes is None):
        genes = table["target_id"]
    else:
        assert(genes == table["target_id"])
    cols.append([float(i) for i in table["tpm"]])
    counts.append([int(float(i)+.5) for i in table["est_counts"]])

tpm_trans = CdtFile(probes = [CdtRow(gid = i[0], uniqid = i[0], name = i[0],
                                        ratios = [safelog(j) for j in i[1:]])
                                 for i in zip(*([genes]+cols))],
                       fieldnames = snames,
                       eweights = [1]*len(snames))
tpm_trans.write(open("tpm_trans.cdt","w"))

trans_counts = CdtFile(probes = [CdtRow(gid = i[0], uniqid = i[0], name = i[0],
                                        ratios = i[1:])
                                 for i in zip(*([genes]+counts))],
                       fieldnames = snames,
                       eweights = [1]*len(snames))
trans_counts.write(open("trans_counts.cdt","w"))

len(tpm_trans), len(trans_counts)
```

Out[5]:

```
(8628, 8628)
```

In [6]:

```
merge_pseudo = PseudoCPMs.fromCounts(trans_counts)
#pseudoCPMs are log transformed
#counts are to feed directly to limma
```

In [7]:

```
mask_10 = merge_pseudo.depth_filter_mask(10,.01)
mask_10.counts.write(open("FigS4_counts_pseudoCPMs_ChIPSeq.cdt", "w"))
```

In [8]:

```
ct_counts = CdtFile.fromCdt("FigS4_counts_pseudoCPMs_ChIPSeq.cdt")
len(ct_counts)
```

Out[8]:

```
8332
```

# Limma analysis¶

In [9]:

```
#need to format ct_counts for limma input

fout = open("ct_counts.txt", "w")

fout.write("\t".join(["gene"]+ct_counts.fieldnames)+"\n")
for row in mask_10.counts:
    fout.write("\t".join([row.Uniqid()]+[str(i) for i in row])+"\n")
fout.close()
```

In [10]:

```
%%R
#Limma Single Factor Fit
# Read the count matrix, using the gene column as row names
C <- read.delim("ct_counts.txt",row.names=1)
#Convert the matrix to limma's preferred format, implicitly log2 transforming and depth normalizing to CPM values
dge <- DGEList(counts=C)
```

In [11]:

```
out = writer(open("FigS4_simple_comp_samples.txt","w"),dialect = excel_tab)
out.writerow(("run","state"))
for i in snames:
    run = i
    state = "_".join(i.split("_")[0:-1])
    out.writerow((run, state))   
del out
```

In [12]:

```
%%R -o d
samples <- read.delim("FigS4_simple_comp_samples.txt", header=TRUE, sep="\t")
print(summary(samples))
state <- samples$state
d <- model.matrix(~0+state)
colnames(d) <- gsub("state","",colnames(d))
print(colnames(d))
```

```
     run               state          
 Length:36          Length:36         
 Class :character   Class :character  
 Mode  :character   Mode  :character  
 [1] "Ryp1_Arth"     "Ryp1_D1_Hyph"  "Ryp1_D1_Spher" "WT_8h_Hyph"   
 [5] "WT_8h_Spher"   "WT_Arth"       "WT_D1_Hyph"    "WT_D1_Spher"  
 [9] "WT_D2_Hyph"    "WT_D2_Spher"   "WT_D4_Hyph"    "WT_D4_Spher"
```

In [13]:

```
%%R
# Apply between-sample TMM normalization
dge <- calcNormFactors(dge)
# Estimate the mean-variance trend via locally-linear regression and use this trend
# to assign weights to the observations (counts)
v <- voom(dge, d, plot = TRUE)
cpm <- v$E
```

In [14]:

```
%%R -o cpm,fc,cn,state
# Fit the model (classic linear regression)
fit <- lmFit(v, d)
#Generate the contrast matrix
contrast.matrix <- makeContrasts(
    Ryp1_D1_Spher - Ryp1_Arth, Ryp1_D1_Hyph - Ryp1_Arth, 
    WT_8h_Spher - WT_Arth, WT_D1_Spher - WT_Arth, WT_D2_Spher -WT_Arth, WT_D4_Spher - WT_Arth, 
    WT_8h_Hyph - WT_Arth, WT_D1_Hyph - WT_Arth, WT_D2_Hyph - WT_Arth, WT_D4_Hyph - WT_Arth, 
    WT_Arth - Ryp1_Arth, WT_D1_Spher - Ryp1_D1_Spher, WT_D1_Hyph - Ryp1_D1_Hyph,
    WT_8h_Spher - WT_8h_Hyph, WT_D1_Spher - WT_D1_Hyph, WT_D2_Spher - WT_D2_Hyph, WT_D4_Spher - WT_D4_Hyph, 
    levels=d)
# Apply the contrast matrix
fit2 <- contrasts.fit(fit, contrast.matrix)
# Apply Empirical Bayes "shrinkage"
fit2 <- eBayes(fit2)
# Simple summary of significantly differential genes with no fold change filter
print(summary(decideTests(fit2)))

fc <- fit$coefficients
cn <- colnames(fit$coefficients)
cpm <- v$E
```

```
       Ryp1_D1_Spher - Ryp1_Arth Ryp1_D1_Hyph - Ryp1_Arth WT_8h_Spher - WT_Arth
Down                        2949                     2723                  3461
NotSig                      2428                     2840                  1757
Up                          2955                     2769                  3114
       WT_D1_Spher - WT_Arth WT_D2_Spher - WT_Arth WT_D4_Spher - WT_Arth
Down                    3418                  3302                  2850
NotSig                  1497                  1608                  2040
Up                      3417                  3422                  3442
       WT_8h_Hyph - WT_Arth WT_D1_Hyph - WT_Arth WT_D2_Hyph - WT_Arth
Down                   3173                 3579                 3537
NotSig                 2177                 1560                 1503
Up                     2982                 3193                 3292
       WT_D4_Hyph - WT_Arth WT_Arth - Ryp1_Arth WT_D1_Spher - Ryp1_D1_Spher
Down                   2613                3404                        2560
NotSig                 2713                2079                        3211
Up                     3006                2849                        2561
       WT_D1_Hyph - Ryp1_D1_Hyph WT_8h_Spher - WT_8h_Hyph
Down                        2448                     2703
NotSig                      3368                     3013
Up                          2516                     2616
       WT_D1_Spher - WT_D1_Hyph WT_D2_Spher - WT_D2_Hyph
Down                       2797                     3004
NotSig                     2717                     2388
Up                         2818                     2940
       WT_D4_Spher - WT_D4_Hyph
Down                       2193
NotSig                     4162
Up                         1977
```

In [15]:

```
%%R
print(summary(decideTests(fit2,lfc=1)))
```

```
       Ryp1_D1_Spher - Ryp1_Arth Ryp1_D1_Hyph - Ryp1_Arth WT_8h_Spher - WT_Arth
Down                        1556                     1277                  2134
NotSig                      5283                     5932                  4481
Up                          1493                     1123                  1717
       WT_D1_Spher - WT_Arth WT_D2_Spher - WT_Arth WT_D4_Spher - WT_Arth
Down                    2146                  2012                  1464
NotSig                  4117                  4254                  4884
Up                      2069                  2066                  1984
       WT_8h_Hyph - WT_Arth WT_D1_Hyph - WT_Arth WT_D2_Hyph - WT_Arth
Down                   1727                 2306                 2204
NotSig                 5136                 4074                 4099
Up                     1469                 1952                 2029
       WT_D4_Hyph - WT_Arth WT_Arth - Ryp1_Arth WT_D1_Spher - Ryp1_D1_Spher
Down                   1510                1816                         946
NotSig                 4894                4954                        6562
Up                     1928                1562                         824
       WT_D1_Hyph - Ryp1_D1_Hyph WT_8h_Spher - WT_8h_Hyph
Down                        1148                      979
NotSig                      6462                     6357
Up                           722                      996
       WT_D1_Spher - WT_D1_Hyph WT_D2_Spher - WT_D2_Hyph
Down                        993                     1259
NotSig                     6057                     5651
Up                         1282                     1422
       WT_D4_Spher - WT_D4_Hyph
Down                        911
NotSig                     6395
Up                         1026
```

In [16]:

```
name2row = dict((i.Uniqid(),n+1) for (n,i) in enumerate(ct_counts))
```

In [17]:

```
fit = SingleFactorFit(fc, cpm, name2row, cn, 
                      state, obs_samples = ct_counts.fieldnames, parameter_order = ("WT_Arth","WT_8h_Spher","WT_D1_Spher", "WT_D2_Spher", "WT_D4_Spher", "WT_8h_Hyph", "WT_D1_Hyph", "WT_D2_Hyph", "WT_D4_Hyph", "Ryp1_Arth", "Ryp1_D1_Spher", "Ryp1_D1_Hyph"))
fit.toHDF5("FigS4_singlecomp_limma1.hdf5")
fit2 = SingleFactorFit.fromHDF5("FigS4_singlecomp_limma1.hdf5")
```

In [18]:

```
ct_counts.mean_normalize_rows().bicluster("ct_counts.norm.um",dist="u",method="m")
```

```
Building array...
Building distance matrix...
Clustering...
```

In [19]:

```
%%R
write.csv(cpm,"limma1.countscutoff.cpm.csv")

for(tc in colnames(fit2$coefficients)){
  print(tc)
  # Extract all genes significantly differential on this contrast for a 2x fold change cutoff and 5% FDR
  # Use write.csv rather than write.table for clean compatibility with python's csv.reader
  write.csv(topTable(fit2, coef=tc, n = 50000, lfc=1, p.value = .05),
            paste("limma1.",gsub(" ","",tc),".t0.csv",sep=""))
  # Extract the adjusted p-values for this contrast for all genes, independent of significance
  write.csv(topTable(fit2, coef=tc, n = 50000),
            paste("limma1.",gsub(" ","",tc),".t1.csv",sep=""))
}
```

```
[1] "Ryp1_D1_Spher - Ryp1_Arth"
[1] "Ryp1_D1_Hyph - Ryp1_Arth"
[1] "WT_8h_Spher - WT_Arth"
[1] "WT_D1_Spher - WT_Arth"
[1] "WT_D2_Spher - WT_Arth"
[1] "WT_D4_Spher - WT_Arth"
[1] "WT_8h_Hyph - WT_Arth"
[1] "WT_D1_Hyph - WT_Arth"
[1] "WT_D2_Hyph - WT_Arth"
[1] "WT_D4_Hyph - WT_Arth"
[1] "WT_Arth - Ryp1_Arth"
[1] "WT_D1_Spher - Ryp1_D1_Spher"
[1] "WT_D1_Hyph - Ryp1_D1_Hyph"
[1] "WT_8h_Spher - WT_8h_Hyph"
[1] "WT_D1_Spher - WT_D1_Hyph"
[1] "WT_D2_Spher - WT_D2_Hyph"
[1] "WT_D4_Spher - WT_D4_Hyph"
```

In [20]:

```
gene2cpms = dict((i[0],[float(j) for j in i[1:]]) for i in Table.fromCsv("limma1.countscutoff.cpm.csv"))
len(gene2cpms), len(ct_counts)
```

Out[20]:

```
(8332, 8332)
```

In [21]:

```
limma1_cdt = CdtFile.fromPrototype(ct_counts, 
                                   probes = [CdtRow.fromPrototype(i, ratios = gene2cpms[i.Uniqid()][:])
                                             for i in ct_counts])
limma1_cdt = limma1_cdt.mean_normalize_rows()
```

In [22]:

```
#Generate columns indicating if a comparison is significant (2-fold cutoff and 5% FDR)

gene2contrasts = dict((i.Uniqid(),[]) for i in limma1_cdt)
gene2pvals = dict((i.Uniqid(),[]) for i in limma1_cdt)
gene2sigs = dict((i.Uniqid(),[]) for i in limma1_cdt)
contrast_names = []

contrast_csvs = []
for i in glob("limma1.*.t1.csv"):
    if "spores" not in i and "week" not in i and "PBS" not in i and "4deg" not in i:
        contrast_csvs.append(i)
contrast_csvs = sorted(contrast_csvs)

for i in contrast_csvs:
    cname = i.replace("limma1.","").replace(".t1.csv","").replace("-","/")
    contrast_names.append(cname)
    siglist = set(i[0] for i in Table.fromCsv(i.replace(".t1.",".t0.")))
    print(cname,len(siglist))
    for gene in Table.fromCsv(i):
        name = gene[0]
        lfc = float(gene["logFC"])
        gene2contrasts[name].append(lfc)
        gene2pvals[name].append(gene["adj.P.Val"])
        if(name in siglist):
            if(lfc > 0):
                gene2sigs[name].append(4.)
            else:
                gene2sigs[name].append(-4.)
        else:
            gene2sigs[name].append(0.)
        
limma1_cdt = CdtFile.fromPrototype(limma1_cdt,
    probes = [CdtRow.fromPrototype(i, ratios = i.ratios+gene2contrasts[i.Uniqid()]+gene2sigs[i.Uniqid()],
                                   extra = i.extra+gene2pvals[i.Uniqid()])
              for i in limma1_cdt],
    fieldnames = limma1_cdt.fieldnames+contrast_names+["%s_sig" % i for i in contrast_names],
    eweights = limma1_cdt.eweights+[1.]*2*len(contrast_names),
    extranames = limma1_cdt.extranames+["p(%s)" % i for i in contrast_names])
```

```
Ryp1_D1_Hyph/Ryp1_Arth 2400
Ryp1_D1_Spher/Ryp1_Arth 3049
WT_8h_Hyph/WT_Arth 3196
WT_8h_Spher/WT_8h_Hyph 1975
WT_8h_Spher/WT_Arth 3851
WT_Arth/Ryp1_Arth 3378
WT_D1_Hyph/Ryp1_D1_Hyph 1870
WT_D1_Hyph/WT_Arth 4258
WT_D1_Spher/Ryp1_D1_Spher 1770
WT_D1_Spher/WT_Arth 4215
WT_D1_Spher/WT_D1_Hyph 2275
WT_D2_Hyph/WT_Arth 4233
WT_D2_Spher/WT_Arth 4078
WT_D2_Spher/WT_D2_Hyph 2681
WT_D4_Hyph/WT_Arth 3438
WT_D4_Spher/WT_Arth 3448
WT_D4_Spher/WT_D4_Hyph 1937
```

In [23]:

```
sig_cols = [n for (n,i) in enumerate(limma1_cdt.fieldnames) if(i.endswith("_sig"))]
contrast_cols = [n-len(sig_cols) for n in sig_cols]
limma1_2x = CdtFile.fromPrototype(limma1_cdt, probes = [i for i in limma1_cdt 
                                                        if(any([(i[j] != 0.) for j in sig_cols]))])
tree = limma1_2x.cluster(cols=contrast_cols,dist="u",method="m")
limma1_2x.writeCdtGtr("limma1_2x.countscutoff.ChIPSeq.contrasts_um",tree)
len(limma1_2x)
```

```
Building array...
Building distance matrix...
Clustering...
```

Out[23]:

```
7506
```

# Looking at the ryp1 vs WT arthroconidia signature in this experiment where the arthroconidia did not sit at 4C, can compare to the D1 comparisons with Ryp1 only¶

In [24]:

```
sig_cols_ryp = [] 
ryp_tp_histogram_pos = {}
ryp_tp_histogram_neg = {}
for n,i in (enumerate(limma1_2x.fieldnames)):
    if i.endswith("_sig"):
        if "WT" in i.split("/")[0]:
            if "Ryp1" in i.split("/")[1] or "ryp1" in i.split("/")[1]:
                sig_cols_ryp.append(n)
                print(i)
                ryp_tp_histogram_pos[i] = 0
                ryp_tp_histogram_neg[i] = 0
sig_cols = [n for (n,i) in enumerate(limma1_2x.fieldnames) if(i.endswith("_sig"))]
contrast_cols = [n-len(sig_cols) for n in sig_cols]

for sig_col in sig_cols_ryp: 
    for i in limma1_2x:
        if i[sig_col] == 4.0:
            ryp_tp_histogram_pos[limma1_2x.fieldnames[sig_col]] += 1
        elif i[sig_col] == -4.0:
            ryp_tp_histogram_neg[limma1_2x.fieldnames[sig_col]] += 1
```

```
WT_Arth/Ryp1_Arth_sig
WT_D1_Hyph/Ryp1_D1_Hyph_sig
WT_D1_Spher/Ryp1_D1_Spher_sig
```

In [25]:

```
#Fig S3A
pos_reordered = sorted(ryp_tp_histogram_pos.items(), key=lambda t:(t[0].split("_")[1],t[0].split("_")[0]))
pos_reordered_new = [pos_reordered[0]] + [['',0]] +[pos_reordered[2]]+ [['',0]] + [pos_reordered[1]]
pos_keys = []
pos_values = []
for entry in pos_reordered_new:
    pos_keys.append(entry[0])
    pos_values.append(entry[1])

neg_reordered = sorted(ryp_tp_histogram_neg.items(), key=lambda t:(t[0].split("_")[1],t[0].split("_")[0]))
neg_reordered_new = [neg_reordered[0]] + [['',0]] +[neg_reordered[2]]+[['',0]]+[neg_reordered[1]]
neg_keys = []
neg_values = []
for entry in neg_reordered_new:
    neg_keys.append(entry[0])
    neg_values.append(-1*entry[1])

x = range(len(neg_values))
fig = plt.figure()
ax = plt.subplot(111)
ax.bar(x,pos_values, width=1, color='mediumpurple')
ax.bar(x,neg_values, width=1, color='limegreen')
fig.savefig("ryp1_dependence_counts.svg")
```

# Define morphology-dependent genes¶

In [26]:

```
sig_cols_morph_wt = [] 
for n,i in (enumerate(limma1_2x.fieldnames)):
    if i.endswith("_sig"):
        comp = i.split("/")
        if "WT" in comp[0] and "8h" not in i:
            if "Ryp" not in comp[1] and "ryp" not in comp[1] and "Arth" not in comp[1]:
                if "pher" in comp[0]:
                    if "pher" not in comp[1]:
                        print(i)
                        sig_cols_morph_wt.append(n)

        elif "Arth" not in comp[1]:
            if "spherule" in comp[0] and "spherule" not in comp[1]:
                print(i)
                sig_cols_morph_ryp1.append(n)

sig_cols = [n for (n,i) in enumerate(limma1_2x.fieldnames) if(i.endswith("_sig"))]
contrast_cols = [n-len(sig_cols) for n in sig_cols_morph_wt]
limma1_morphdep_all_wt = CdtFile.fromPrototype(limma1_2x, probes = [i for i in limma1_2x
                                                        if(all([(i[j] != 0.) for j in sig_cols_morph_wt]))])
tree = limma1_morphdep_all_wt.cluster(cols=contrast_cols,dist="u",method="m")
limma1_morphdep_all_wt.writeCdtGtr("limma1_morphdep_all_wt.contrasts_um",tree)
print(len(limma1_morphdep_all_wt))
```

```
WT_D1_Spher/WT_D1_Hyph_sig
WT_D2_Spher/WT_D2_Hyph_sig
WT_D4_Spher/WT_D4_Hyph_sig
```

```
Building array...
Building distance matrix...
Clustering...
```

```
786
```

In [27]:

```
chipseq_peaks = CdtFile.fromCdt("../Fig4/chipseq_peaks.um.cdt")

#make a list of consistently morphology-regulated and directly bound using Fig S4 data
count = 0
for row in limma1_morphdep_all_wt:
    matched = False
    gene = row.uniqid
    for row2 in chipseq_peaks:
        if row.uniqid == row2.uniqid:
            matched = True
    if matched == True:
        count += 1
print("bound genes: {0}".format(count))
print("% bound: {0}".format(float(count)/len(limma1_morphdep_all_wt)))
print(len(limma1_morphdep_all_wt))
```

```
bound genes: 418
% bound: 0.5318066157760815
786
```

In [28]:

```
#make a list of consistently morphology-regulated and directly bound using Fig 2 data
morph_Fig2 = CdtFile.fromCdt("../Fig2/Combined/limma1_morphdep_all_wt.contrasts_um.cdt")

count = 0
for row in morph_Fig2:
    matched = False
    gene = row.uniqid
    for row2 in chipseq_peaks:
        if row.uniqid == row2.uniqid:
            matched = True
    if matched == True:
        count += 1
print("bound genes: {0}".format(count))
print("% bound: {0}".format(float(count)/len(morph_Fig2)))
print(len(morph_Fig2))
```

```
bound genes: 308
% bound: 0.558983666061706
551
```

In [29]:

```
#Fig S4E
xs = ["Fig 2", "Fig S4"]
rna_targets = [1,1]
bound = [308./551.,418./786.]
fig = plt.figure()
ax = plt.subplot(111)
barlist = ax.bar(xs,rna_targets, width=1, color='mediumpurple', edgecolor='black')
barlist1 = ax.bar(xs,bound, width=1, color='rebeccapurple', edgecolor='black',hatch='//')
fig.savefig("morphology_dependence_bound_relationship_normalized.svg")
fig.savefig("morphology_dependence_bound_relationship_normalized.png")
```

In [ ]:

```

```
