# Supplementary material for: Transcriptomic atlas throughout Coccidioides development reveals key phase-enriched transcripts of this important fungal pathogen
Source: PLoS Biol. 2025 Apr 15;23(4):e3003066. doi: 10.1371/journal.pbio.3003066 (PMC12077801; doi:10.1371/journal.pbio.3003066)
Supplement: S1 Code — Folder containing README document describing the scripts used to analyze the data and generate figures in this manuscript, as well as the scripts themselves and custom python three modules used in the scripts. (ZIP) [file pbio.3003066.s025.zip › Custom Code/notebooks/Fig2.html]

Fig2


In [1]:

```
from CdtFile import CdtFile, CdtRow
from CdtAnnotator3 import annotate_CpSilveira as annotate
from LimmaTools import SingleFactorFit
from MsvUtil import Table
from csv import writer, excel_tab
from glob import glob
from math import log, atan2, pi
import os.path
import re
from SafeMath import safelog
from PCA import PCA
import matplotlib.pyplot as plt
%matplotlib nbagg
%load_ext rpy2.ipython
from ReadCountTools import PseudoCPMs
```

In [2]:

```
%%R
library(limma)
library(edgeR)
```

# Merge Kallisto TPMs - spherule only for Fig 2C¶

In [3]:

```
cd ../../Papers/Cocci_transcriptomics/data_for_code/Fig2/Spher/
```

```
/home/chomer/Papers/Cocci_transcriptomics/data_for_code/Fig2/Spher
```

In [4]:

```
#First Index

sname2kallisto = dict(
    ("_".join(i.replace(".Silv_nanopore_mRNA.rf.kallisto","").replace("../", "").split("_")[0:-3]),i)
              for i in glob("../"+"*.Silv_nanopore_mRNA.rf.kallisto") if("myc" not in i and "DMEM" not in i and "RPMI" not in i))

snames = sorted(sname2kallisto, key = lambda x: (x[0:x.rfind("_")],x[x.rfind("_"):]), reverse=False)

print(len(sname2kallisto))
print(snames)
```

```
48
['D1_spherule_Ryp1_1', 'D1_spherule_Ryp1_2', 'D1_spherule_Ryp1_3', 'D1_spherule_Sil_1', 'D1_spherule_Sil_2', 'D1_spherule_Sil_3', 'D2_spherule_Ryp1_1', 'D2_spherule_Ryp1_2', 'D2_spherule_Ryp1_3', 'D2_spherule_Sil_1', 'D2_spherule_Sil_2', 'D2_spherule_Sil_3', 'D3_spherules_Ryp1_1', 'D3_spherules_Ryp1_2', 'D3_spherules_Ryp1_3', 'D3_spherules_Sil_1', 'D3_spherules_Sil_2', 'D3_spherules_Sil_3', 'D4_spherules_Ryp1_1', 'D4_spherules_Ryp1_2', 'D4_spherules_Ryp1_3', 'D4_spherules_Sil_1', 'D4_spherules_Sil_2', 'D4_spherules_Sil_3', 'D5_spherules_Ryp1_1', 'D5_spherules_Ryp1_2', 'D5_spherules_Ryp1_3', 'D5_spherules_Sil_1', 'D5_spherules_Sil_2', 'D5_spherules_Sil_3', 'D6_spherules_Ryp1_1', 'D6_spherules_Ryp1_2', 'D6_spherules_Ryp1_3', 'D6_spherules_Sil_1', 'D6_spherules_Sil_2', 'D6_spherules_Sil_3', 'Eighth_spherule_Sil_1', 'Eighth_spherule_Sil_2', 'Eighth_spherule_Sil_3', 'Eighth_spherule_ryp1_1', 'Eighth_spherule_ryp1_2', 'Eighth_spherule_ryp1_3', 'Spores_Ryp1_1', 'Spores_Ryp1_2', 'Spores_Ryp1_3', 'Spores_Sil_1', 'Spores_Sil_2', 'Spores_Sil_3']
```

In [5]:

```
#Merge Step
genes = None
cols = []
counts = []
for i in snames:
    #print(i)
    table = Table.fromTdt(open(os.path.join(
           sname2kallisto[i],
          "abundance.tsv")))
    if(genes is None):
        genes = table["target_id"]
    else:
        assert(genes == table["target_id"])
    cols.append([float(i) for i in table["tpm"]])
    counts.append([int(float(i)+.5) for i in table["est_counts"]])

tpm_trans = CdtFile(probes = [CdtRow(gid = i[0], uniqid = i[0], name = i[0],
                                        ratios = [safelog(j) for j in i[1:]])
                                 for i in zip(*([genes]+cols))],
                       fieldnames = snames,
                       eweights = [1]*len(snames))
tpm_trans.write(open("tpm_trans.cdt","w"))

trans_counts = CdtFile(probes = [CdtRow(gid = i[0], uniqid = i[0], name = i[0],
                                        ratios = i[1:])
                                 for i in zip(*([genes]+counts))],
                       fieldnames = snames,
                       eweights = [1]*len(snames))
trans_counts.write(open("trans_counts.cdt","w"))

len(tpm_trans), len(trans_counts)
```

Out[5]:

```
(8628, 8628)
```

In [6]:

```
merge_pseudo = PseudoCPMs.fromCounts(trans_counts)
```

In [7]:

```
#filter out transcripts that do not have at least 10 reads in at least 3 or more datasets
mask_10 = merge_pseudo.depth_filter_mask(10,.07)
mask_10.counts.write(open("Fig2C_counts_pseudoCPMs.for_paper.cdt", "w"))
```

In [8]:

```
ct_counts = CdtFile.fromCdt("Fig2C_counts_pseudoCPMs.for_paper.cdt")
len(ct_counts)
```

Out[8]:

```
8175
```

## Limma analysis¶

In [9]:

```
#need to format ct_tpm for limma input
fout = open("ct_counts.txt", "w")

fout.write("\t".join(["gene"]+ct_counts.fieldnames)+"\n")
for row in mask_10.counts:
    fout.write("\t".join([row.Uniqid()]+[str(i) for i in row])+"\n")
fout.close()
```

In [10]:

```
%%R
#Limma Single Factor Fit
# Read the count matrix, using the gene column as row names
C <- read.delim("ct_counts.txt",row.names=1)
#Convert the matrix to limma's preferred format, implicitly log2 transforming and depth normalizing to CPM values
dge <- DGEList(counts=C)
```

In [11]:

```
out = writer(open("Fig2_simple_comp_samples.txt","w"),dialect = excel_tab)
out.writerow(("run","state"))
for i in snames:
    run = i
    state = "_".join(i.split("_")[0:-1])
    out.writerow((run, state))   
del out
```

In [12]:

```
%%R -o d
samples <- read.delim("Fig2_simple_comp_samples.txt")
print(summary(samples))
state <- samples$state
d <- model.matrix(~0+state)
colnames(d) <- gsub("state","",colnames(d))
print(colnames(d))
```

```
     run               state          
 Length:48          Length:48         
 Class :character   Class :character  
 Mode  :character   Mode  :character  
 [1] "D1_spherule_Ryp1"     "D1_spherule_Sil"      "D2_spherule_Ryp1"    
 [4] "D2_spherule_Sil"      "D3_spherules_Ryp1"    "D3_spherules_Sil"    
 [7] "D4_spherules_Ryp1"    "D4_spherules_Sil"     "D5_spherules_Ryp1"   
[10] "D5_spherules_Sil"     "D6_spherules_Ryp1"    "D6_spherules_Sil"    
[13] "Eighth_spherule_ryp1" "Eighth_spherule_Sil"  "Spores_Ryp1"         
[16] "Spores_Sil"
```

In [13]:

```
%%R
# Apply between-sample TMM normalization
dge <- calcNormFactors(dge)
# Estimate the mean-variance trend via locally-linear regression and use this trend
# to assign weights to the observations (counts)
v <- voom(dge, d, plot = TRUE)
cpm <- v$E
```

In [14]:

```
%%R -o cpm,fc,cn,state
# Fit the model (classic linear regression)
fit <- lmFit(v, d)
#Generate the contrast matrix
contrast.matrix <- makeContrasts(
    Eighth_spherule_Sil - Spores_Sil, D1_spherule_Sil - Spores_Sil, D2_spherule_Sil - Spores_Sil, D3_spherules_Sil - Spores_Sil, D4_spherules_Sil - Spores_Sil, D5_spherules_Sil - Spores_Sil, D6_spherules_Sil - Spores_Sil,
    Eighth_spherule_ryp1 - Spores_Ryp1, D1_spherule_Ryp1 - Spores_Ryp1, D2_spherule_Ryp1 - Spores_Ryp1, D3_spherules_Ryp1 - Spores_Ryp1, D4_spherules_Ryp1 - Spores_Ryp1, D5_spherules_Ryp1 - Spores_Ryp1, D6_spherules_Ryp1 - Spores_Ryp1,
    Spores_Sil - Spores_Ryp1,
    Eighth_spherule_Sil - Eighth_spherule_ryp1, D1_spherule_Sil - D1_spherule_Ryp1, D2_spherule_Sil - D2_spherule_Ryp1, D3_spherules_Sil - D3_spherules_Ryp1, D4_spherules_Sil - D4_spherules_Ryp1, D5_spherules_Sil - D5_spherules_Ryp1, D6_spherules_Sil - D6_spherules_Ryp1,
    D1_spherule_Sil - Eighth_spherule_Sil, D2_spherule_Sil - D1_spherule_Sil, D3_spherules_Sil - D2_spherule_Sil, D4_spherules_Sil - D3_spherules_Sil, D5_spherules_Sil - D4_spherules_Sil, D6_spherules_Sil - D5_spherules_Sil,
    levels=d)
# Apply the contrast matrix
fit2 <- contrasts.fit(fit, contrast.matrix)
# Apply Empirical Bayes "shrinkage"
fit2 <- eBayes(fit2)
# Simple summary of significantly differential genes with no fold change filter
print(summary(decideTests(fit2)))

fc <- fit$coefficients
cn <- colnames(fit$coefficients)
cpm <- v$E
```

```
       Eighth_spherule_Sil - Spores_Sil D1_spherule_Sil - Spores_Sil
Down                               2744                         2898
NotSig                             2451                         2235
Up                                 2980                         3042
       D2_spherule_Sil - Spores_Sil D3_spherules_Sil - Spores_Sil
Down                           2735                          2843
NotSig                         2342                          2327
Up                             3098                          3005
       D4_spherules_Sil - Spores_Sil D5_spherules_Sil - Spores_Sil
Down                            2766                          2743
NotSig                          2552                          2526
Up                              2857                          2906
       D6_spherules_Sil - Spores_Sil Eighth_spherule_ryp1 - Spores_Ryp1
Down                            2617                               2497
NotSig                          2731                               3332
Up                              2827                               2346
       D1_spherule_Ryp1 - Spores_Ryp1 D2_spherule_Ryp1 - Spores_Ryp1
Down                             2717                           2779
NotSig                           2707                           2567
Up                               2751                           2829
       D3_spherules_Ryp1 - Spores_Ryp1 D4_spherules_Ryp1 - Spores_Ryp1
Down                              2657                            2565
NotSig                            2708                            2733
Up                                2810                            2877
       D5_spherules_Ryp1 - Spores_Ryp1 D6_spherules_Ryp1 - Spores_Ryp1
Down                              2580                            2471
NotSig                            2945                            3098
Up                                2650                            2606
       Spores_Sil - Spores_Ryp1 Eighth_spherule_Sil - Eighth_spherule_ryp1
Down                       2561                                       1723
NotSig                     3073                                       4326
Up                         2541                                       2126
       D1_spherule_Sil - D1_spherule_Ryp1 D2_spherule_Sil - D2_spherule_Ryp1
Down                                 2037                               2014
NotSig                               4031                               4040
Up                                   2107                               2121
       D3_spherules_Sil - D3_spherules_Ryp1
Down                                   2475
NotSig                                 3531
Up                                     2169
       D4_spherules_Sil - D4_spherules_Ryp1
Down                                   2631
NotSig                                 3107
Up                                     2437
       D5_spherules_Sil - D5_spherules_Ryp1
Down                                   1904
NotSig                                 4348
Up                                     1923
       D6_spherules_Sil - D6_spherules_Ryp1
Down                                   1962
NotSig                                 4061
Up                                     2152
       D1_spherule_Sil - Eighth_spherule_Sil D2_spherule_Sil - D1_spherule_Sil
Down                                    1693                              1579
NotSig                                  4924                              5152
Up                                      1558                              1444
       D3_spherules_Sil - D2_spherule_Sil D4_spherules_Sil - D3_spherules_Sil
Down                                 1530                                 789
NotSig                               5536                                6400
Up                                   1109                                 986
       D5_spherules_Sil - D4_spherules_Sil D6_spherules_Sil - D5_spherules_Sil
Down                                   182                                  71
NotSig                                7809                                7951
Up                                     184                                 153
```

In [15]:

```
name2row = dict((i.Uniqid(),n+1) for (n,i) in enumerate(ct_counts))
```

In [16]:

```
name2row = dict((i.Uniqid(),n+1) for (n,i) in enumerate(ct_counts))
fit = SingleFactorFit(fc, cpm, name2row, cn, 
                      state, obs_samples = ct_counts.fieldnames, 
                      parameter_order = ("Spores_Sil","Eighth_spherule_Sil","D1_spherule_Sil", "D2_spherule_Sil", "D3_spherules_Sil", "D4_spherules_Sil", "D5_spherules_Sil", "D6_spherules_Sil", "Spores_Ryp1","Eighth_spherule_ryp1","D1_spherule_Ryp1", "D2_spherule_Ryp1", "D3_spherules_Ryp1", "D4_spherules_Ryp1", "D5_spherules_Ryp1", "D6_spherules_Ryp1"))
fit.toHDF5("Fig2_spher_singlecomp_limma1.hdf5")
fit2 = SingleFactorFit.fromHDF5("Fig2_spher_singlecomp_limma1.hdf5")
```

In [17]:

```
%%R
write.csv(cpm,"limma1.countscpm.cpm.csv")
```

In [18]:

```
%%R
for(tc in colnames(fit2$coefficients)){
  print(tc)
  # Extract all genes significantly differential on this contrast for a 2x fold change cutoff and 5% FDR
  # Use write.csv rather than write.table for clean compatibility with python's csv.reader
  write.csv(topTable(fit2, coef=tc, n = 50000, lfc=1, p.value = .05),
            paste("limma1.",gsub(" ","",tc),".t0.csv",sep=""))
  # Extract the adjusted p-values for this contrast for all genes, independent of significance
  write.csv(topTable(fit2, coef=tc, n = 50000),
            paste("limma1.",gsub(" ","",tc),".t1.csv",sep=""))
}
```

```
[1] "Eighth_spherule_Sil - Spores_Sil"
[1] "D1_spherule_Sil - Spores_Sil"
[1] "D2_spherule_Sil - Spores_Sil"
[1] "D3_spherules_Sil - Spores_Sil"
[1] "D4_spherules_Sil - Spores_Sil"
[1] "D5_spherules_Sil - Spores_Sil"
[1] "D6_spherules_Sil - Spores_Sil"
[1] "Eighth_spherule_ryp1 - Spores_Ryp1"
[1] "D1_spherule_Ryp1 - Spores_Ryp1"
[1] "D2_spherule_Ryp1 - Spores_Ryp1"
[1] "D3_spherules_Ryp1 - Spores_Ryp1"
[1] "D4_spherules_Ryp1 - Spores_Ryp1"
[1] "D5_spherules_Ryp1 - Spores_Ryp1"
[1] "D6_spherules_Ryp1 - Spores_Ryp1"
[1] "Spores_Sil - Spores_Ryp1"
[1] "Eighth_spherule_Sil - Eighth_spherule_ryp1"
[1] "D1_spherule_Sil - D1_spherule_Ryp1"
[1] "D2_spherule_Sil - D2_spherule_Ryp1"
[1] "D3_spherules_Sil - D3_spherules_Ryp1"
[1] "D4_spherules_Sil - D4_spherules_Ryp1"
[1] "D5_spherules_Sil - D5_spherules_Ryp1"
[1] "D6_spherules_Sil - D6_spherules_Ryp1"
[1] "D1_spherule_Sil - Eighth_spherule_Sil"
[1] "D2_spherule_Sil - D1_spherule_Sil"
[1] "D3_spherules_Sil - D2_spherule_Sil"
[1] "D4_spherules_Sil - D3_spherules_Sil"
[1] "D5_spherules_Sil - D4_spherules_Sil"
[1] "D6_spherules_Sil - D5_spherules_Sil"
```

In [19]:

```
gene2cpms = dict((i[0],[float(j) for j in i[1:]]) for i in Table.fromCsv("limma1.countscpm.cpm.csv"))
len(gene2cpms), len(ct_counts)
```

Out[19]:

```
(8175, 8175)
```

In [20]:

```
limma1_cdt = CdtFile.fromPrototype(ct_counts, 
                                   probes = [CdtRow.fromPrototype(i, ratios = gene2cpms[i.Uniqid()][:])
                                             for i in ct_counts])
limma1_cdt = limma1_cdt.mean_normalize_rows()
```

In [21]:

```
#Generate columns indicating if a comparison is significant (2-fold cutoff and 5% FDR)

gene2contrasts = dict((i.Uniqid(),[]) for i in limma1_cdt)
gene2pvals = dict((i.Uniqid(),[]) for i in limma1_cdt)
gene2sigs = dict((i.Uniqid(),[]) for i in limma1_cdt)
contrast_names = []

contrast_csvs = sorted(glob("limma1.*.t1.csv"))
# put 8 hour first
contrast_csvs = contrast_csvs[-1:]+contrast_csvs[:-1]
for i in contrast_csvs:
    cname = i.replace("limma1.","").replace(".t1.csv","").replace("-","/")
    contrast_names.append(cname)
    siglist = set(i[0] for i in Table.fromCsv(i.replace(".t1.",".t0.")))
    print(cname,len(siglist))
    for gene in Table.fromCsv(i):
        name = gene[0]
        lfc = float(gene["logFC"])
        gene2contrasts[name].append(lfc)
        gene2pvals[name].append(gene["adj.P.Val"])
        if(name in siglist):
            if(lfc > 0):
                gene2sigs[name].append(4.)
            else:
                gene2sigs[name].append(-4.)
        else:
            gene2sigs[name].append(0.)
        
limma1_cdt = CdtFile.fromPrototype(limma1_cdt,
    probes = [CdtRow.fromPrototype(i, ratios = i.ratios+gene2contrasts[i.Uniqid()]+gene2sigs[i.Uniqid()],
                                   extra = i.extra+gene2pvals[i.Uniqid()])
              for i in limma1_cdt],
    fieldnames = limma1_cdt.fieldnames+contrast_names+["%s_sig" % i for i in contrast_names],
    eweights = limma1_cdt.eweights+[1.]*2*len(contrast_names),
    extranames = limma1_cdt.extranames+["p(%s)" % i for i in contrast_names])
```

```
Spores_Sil/Spores_Ryp1 3616
D1_spherule_Ryp1/Spores_Ryp1 3810
D1_spherule_Sil/D1_spherule_Ryp1 2200
D1_spherule_Sil/Eighth_spherule_Sil 1442
D1_spherule_Sil/Spores_Sil 4523
D2_spherule_Ryp1/Spores_Ryp1 3850
D2_spherule_Sil/D1_spherule_Sil 1393
D2_spherule_Sil/D2_spherule_Ryp1 2549
D2_spherule_Sil/Spores_Sil 4611
D3_spherules_Ryp1/Spores_Ryp1 3701
D3_spherules_Sil/D2_spherule_Sil 1242
D3_spherules_Sil/D3_spherules_Ryp1 2580
D3_spherules_Sil/Spores_Sil 4377
D4_spherules_Ryp1/Spores_Ryp1 3663
D4_spherules_Sil/D3_spherules_Sil 474
D4_spherules_Sil/D4_spherules_Ryp1 2982
D4_spherules_Sil/Spores_Sil 3972
D5_spherules_Ryp1/Spores_Ryp1 3348
D5_spherules_Sil/D4_spherules_Sil 142
D5_spherules_Sil/D5_spherules_Ryp1 1910
D5_spherules_Sil/Spores_Sil 3998
D6_spherules_Ryp1/Spores_Ryp1 3190
D6_spherules_Sil/D5_spherules_Sil 71
D6_spherules_Sil/D6_spherules_Ryp1 2060
D6_spherules_Sil/Spores_Sil 3797
Eighth_spherule_Sil/Eighth_spherule_ryp1 2050
Eighth_spherule_Sil/Spores_Sil 4289
Eighth_spherule_ryp1/Spores_Ryp1 3158
```

In [22]:

```
limma1_cdt = CdtFile.fromPrototype(limma1_cdt, 
    probes = [CdtRow.fromPrototype(i, extra = i.extra + [str(j) for j in ct_counts.GetUid(i.Uniqid())])
              for i in limma1_cdt],
    extranames = limma1_cdt.extranames+["%s_counts" % i for i in ct_counts.fieldnames])
```

In [23]:

```
#assign count columns and cluster columns
count_cols = [n for (n,i) in enumerate(limma1_cdt.fieldnames) if("/" not in i)]
print(count_cols)
#cluster on count columns
tree = limma1_cdt.cluster(cols=count_cols,dist="u",method="m")
len(limma1_cdt)
```

```
[0, 1, 2, 3, 4, 5, 6, 7, 8, 9, 10, 11, 12, 13, 14, 15, 16, 17, 18, 19, 20, 21, 22, 23, 24, 25, 26, 27, 28, 29, 30, 31, 32, 33, 34, 35, 36, 37, 38, 39, 40, 41, 42, 43, 44, 45, 46, 47]
```

```
Building array...
Building distance matrix...
Clustering...
```

Out[23]:

```
8175
```

In [24]:

```
limma1_cdt = annotate(limma1_cdt, warn = False, map_from="V3")
```

```
Warning, didn't find a GID column!  Sharing UID with GID.
```

In [25]:

```
limma1_cdt.writeCdtGtr("limma1.countscutoff.cdt", tree)
#This spreadsheet was used for heatmap in Figure 2C
```

# Merge Kallisto TPMs - hyphal only for Fig 2F¶

In [26]:

```
cd ../Hyph/
```

```
/home/chomer/Papers/Cocci_transcriptomics/data_for_code/Fig2/Hyph
```

In [27]:

```
#First Index

sname2kallisto = dict(
    ("_".join(i.replace(".Silv_nanopore_mRNA.rf.kallisto","").replace("../", "").split("_")[0:-3]),i)
              for i in glob("../"+"*.Silv_nanopore_mRNA.rf.kallisto") if("her" not in i and "DMEM" not in i and "RPMI" not in i))

snames = sorted(sname2kallisto, key = lambda x: (x[0:x.rfind("_")],x[x.rfind("_"):]), reverse=False)

print(len(sname2kallisto))
print(snames)
```

```
36
['D1_myc_Ryp1_1', 'D1_myc_Ryp1_2', 'D1_myc_Ryp1_3', 'D1_myc_Sil_1', 'D1_myc_Sil_2', 'D1_myc_Sil_3', 'D2_myc_Ryp1_1', 'D2_myc_Ryp1_2', 'D2_myc_Ryp1_3', 'D2_myc_Sil_1', 'D2_myc_Sil_2', 'D2_myc_Sil_3', 'D3_myc_Ryp1_1', 'D3_myc_Ryp1_2', 'D3_myc_Ryp1_3', 'D3_myc_Sil_1', 'D3_myc_Sil_2', 'D3_myc_Sil_3', 'D6_myc_Ryp1_1', 'D6_myc_Ryp1_2', 'D6_myc_Ryp1_3', 'D6_myc_Sil_1', 'D6_myc_Sil_2', 'D6_myc_Sil_3', 'Eighth_myc_Ryp1_1', 'Eighth_myc_Ryp1_2', 'Eighth_myc_Ryp1_3', 'Eighth_myc_Sil_1', 'Eighth_myc_Sil_2', 'Eighth_myc_Sil_3', 'Spores_Ryp1_1', 'Spores_Ryp1_2', 'Spores_Ryp1_3', 'Spores_Sil_1', 'Spores_Sil_2', 'Spores_Sil_3']
```

In [28]:

```
#Merge Step
genes = None
cols = []
counts = []
for i in snames:
    table = Table.fromTdt(open(os.path.join(
           sname2kallisto[i],
          "abundance.tsv")))
    if(genes is None):
        genes = table["target_id"]
    else:
        assert(genes == table["target_id"])
    cols.append([float(i) for i in table["tpm"]])
    counts.append([int(float(i)+.5) for i in table["est_counts"]])

tpm_trans = CdtFile(probes = [CdtRow(gid = i[0], uniqid = i[0], name = i[0],
                                        ratios = [safelog(j) for j in i[1:]])
                                 for i in zip(*([genes]+cols))],
                       fieldnames = snames,
                       eweights = [1]*len(snames))
tpm_trans.write(open("tpm_trans.cdt","w"))

trans_counts = CdtFile(probes = [CdtRow(gid = i[0], uniqid = i[0], name = i[0],
                                        ratios = i[1:])
                                 for i in zip(*([genes]+counts))],
                       fieldnames = snames,
                       eweights = [1]*len(snames))
trans_counts.write(open("trans_counts.cdt","w"))

len(tpm_trans), len(trans_counts)
```

Out[28]:

```
(8628, 8628)
```

In [29]:

```
merge_pseudo = PseudoCPMs.fromCounts(trans_counts)
```

In [30]:

```
#filter out transcripts that do not have at least 10 reads in at least 3 or more datasets
mask_10 = merge_pseudo.depth_filter_mask(10,.07)
mask_10.counts.write(open("Fig2F_counts_pseudoCPMs.for_paper.cdt", "w"))
```

In [31]:

```
ct_counts = CdtFile.fromCdt("Fig2F_counts_pseudoCPMs.for_paper.cdt")
len(ct_counts)
```

Out[31]:

```
8151
```

## Limma analysis¶

In [32]:

```
#need to format ct_tpm for limma input
fout = open("ct_counts.txt", "w")

fout.write("\t".join(["gene"]+ct_counts.fieldnames)+"\n")
for row in mask_10.counts:
    fout.write("\t".join([row.Uniqid()]+[str(i) for i in row])+"\n")
fout.close()
```

In [33]:

```
%%R
#Limma Single Factor Fit
# Read the count matrix, using the gene column as row names
C <- read.delim("ct_counts.txt",row.names=1)
#Convert the matrix to limma's preferred format, implicitly log2 transforming and depth normalizing to CPM values
dge <- DGEList(counts=C)
```

In [34]:

```
out = writer(open("Fig2_simple_comp_samples.txt","w"),dialect = excel_tab)
out.writerow(("run","state"))
for i in snames:
    run = i
    state = "_".join(i.split("_")[0:-1])
    out.writerow((run, state))   
del out
```

In [35]:

```
%%R -o d
samples <- read.delim("Fig2_simple_comp_samples.txt")
print(summary(samples))
state <- samples$state
d <- model.matrix(~0+state)
colnames(d) <- gsub("state","",colnames(d))
print(colnames(d))
```

```
     run               state          
 Length:36          Length:36         
 Class :character   Class :character  
 Mode  :character   Mode  :character  
 [1] "D1_myc_Ryp1"     "D1_myc_Sil"      "D2_myc_Ryp1"     "D2_myc_Sil"     
 [5] "D3_myc_Ryp1"     "D3_myc_Sil"      "D6_myc_Ryp1"     "D6_myc_Sil"     
 [9] "Eighth_myc_Ryp1" "Eighth_myc_Sil"  "Spores_Ryp1"     "Spores_Sil"
```

In [36]:

```
%%R
# Apply between-sample TMM normalization
dge <- calcNormFactors(dge)
# Estimate the mean-variance trend via locally-linear regression and use this trend
# to assign weights to the observations (counts)
v <- voom(dge, d, plot = TRUE)
cpm <- v$E
```

In [37]:

```
%%R -o cpm,fc,cn,state
# Fit the model (classic linear regression)
fit <- lmFit(v, d)
#Generate the contrast matrix
contrast.matrix <- makeContrasts(
    Eighth_myc_Sil - Spores_Sil, D1_myc_Sil - Spores_Sil, D2_myc_Sil - Spores_Sil, D3_myc_Sil - Spores_Sil, D6_myc_Sil - Spores_Sil,
    Eighth_myc_Ryp1 - Spores_Ryp1, D1_myc_Ryp1 - Spores_Ryp1, D2_myc_Ryp1 - Spores_Ryp1, D3_myc_Ryp1 - Spores_Ryp1, D6_myc_Ryp1 - Spores_Ryp1,
    Spores_Sil - Spores_Ryp1,
    Eighth_myc_Sil - Eighth_myc_Ryp1, D1_myc_Sil - D1_myc_Ryp1, D2_myc_Sil - D2_myc_Ryp1, D3_myc_Sil - D3_myc_Ryp1, D6_myc_Sil - D6_myc_Ryp1,
    D1_myc_Sil - Eighth_myc_Sil, D2_myc_Sil - D1_myc_Sil, D3_myc_Sil - D2_myc_Sil, D6_myc_Sil - D3_myc_Sil,
    levels=d)
# Apply the contrast matrix
fit2 <- contrasts.fit(fit, contrast.matrix)
# Apply Empirical Bayes "shrinkage"
fit2 <- eBayes(fit2)
# Simple summary of significantly differential genes with no fold change filter
print(summary(decideTests(fit2)))

fc <- fit$coefficients
cn <- colnames(fit$coefficients)
cpm <- v$E
```

```
       Eighth_myc_Sil - Spores_Sil D1_myc_Sil - Spores_Sil
Down                          2359                    2470
NotSig                        3252                    2721
Up                            2540                    2960
       D2_myc_Sil - Spores_Sil D3_myc_Sil - Spores_Sil D6_myc_Sil - Spores_Sil
Down                      2560                    2555                    2490
NotSig                    2675                    2614                    2877
Up                        2916                    2982                    2784
       Eighth_myc_Ryp1 - Spores_Ryp1 D1_myc_Ryp1 - Spores_Ryp1
Down                            2389                      2421
NotSig                          3739                      3497
Up                              2023                      2233
       D2_myc_Ryp1 - Spores_Ryp1 D3_myc_Ryp1 - Spores_Ryp1
Down                        2627                      2486
NotSig                      2957                      3116
Up                          2567                      2549
       D6_myc_Ryp1 - Spores_Ryp1 Spores_Sil - Spores_Ryp1
Down                        2505                     2509
NotSig                      3099                     3389
Up                          2547                     2253
       Eighth_myc_Sil - Eighth_myc_Ryp1 D1_myc_Sil - D1_myc_Ryp1
Down                               1774                     1493
NotSig                             4259                     4461
Up                                 2118                     2197
       D2_myc_Sil - D2_myc_Ryp1 D3_myc_Sil - D3_myc_Ryp1
Down                       1697                     1206
NotSig                     4998                     5760
Up                         1456                     1185
       D6_myc_Sil - D6_myc_Ryp1 D1_myc_Sil - Eighth_myc_Sil
Down                        864                        1137
NotSig                     6530                        5365
Up                          757                        1649
       D2_myc_Sil - D1_myc_Sil D3_myc_Sil - D2_myc_Sil D6_myc_Sil - D3_myc_Sil
Down                      2090                     171                      43
NotSig                    4774                    7768                    7997
Up                        1287                     212                     111
```

In [38]:

```
name2row = dict((i.Uniqid(),n+1) for (n,i) in enumerate(ct_counts))
```

In [39]:

```
name2row = dict((i.Uniqid(),n+1) for (n,i) in enumerate(ct_counts))
fit = SingleFactorFit(fc, cpm, name2row, cn, 
                      state, obs_samples = ct_counts.fieldnames, 
                      parameter_order = ("Spores_Sil","Eighth_myc_Sil","D1_myc_Sil", "D2_myc_Sil", "D3_myc_Sil", "D6_myc_Sil","Spores_Ryp1","Eighth_myc_Ryp1","D1_myc_Ryp1", "D2_myc_Ryp1", "D3_myc_Ryp1", "D6_myc_Ryp1"))
fit.toHDF5("Fig2_hyph_singlecomp_limma1.hdf5")
fit2 = SingleFactorFit.fromHDF5("Fig2_hyph_singlecomp_limma1.hdf5")
```

In [40]:

```
%%R
write.csv(cpm,"limma1.countscpm.cpm.csv")
```

In [41]:

```
%%R
for(tc in colnames(fit2$coefficients)){
  print(tc)
  # Extract all genes significantly differential on this contrast for a 2x fold change cutoff and 5% FDR
  # Use write.csv rather than write.table for clean compatibility with python's csv.reader
  write.csv(topTable(fit2, coef=tc, n = 50000, lfc=1, p.value = .05),
            paste("limma1.",gsub(" ","",tc),".t0.csv",sep=""))
  # Extract the adjusted p-values for this contrast for all genes, independent of significance
  write.csv(topTable(fit2, coef=tc, n = 50000),
            paste("limma1.",gsub(" ","",tc),".t1.csv",sep=""))
}
```

```
[1] "Eighth_myc_Sil - Spores_Sil"
[1] "D1_myc_Sil - Spores_Sil"
[1] "D2_myc_Sil - Spores_Sil"
[1] "D3_myc_Sil - Spores_Sil"
[1] "D6_myc_Sil - Spores_Sil"
[1] "Eighth_myc_Ryp1 - Spores_Ryp1"
[1] "D1_myc_Ryp1 - Spores_Ryp1"
[1] "D2_myc_Ryp1 - Spores_Ryp1"
[1] "D3_myc_Ryp1 - Spores_Ryp1"
[1] "D6_myc_Ryp1 - Spores_Ryp1"
[1] "Spores_Sil - Spores_Ryp1"
[1] "Eighth_myc_Sil - Eighth_myc_Ryp1"
[1] "D1_myc_Sil - D1_myc_Ryp1"
[1] "D2_myc_Sil - D2_myc_Ryp1"
[1] "D3_myc_Sil - D3_myc_Ryp1"
[1] "D6_myc_Sil - D6_myc_Ryp1"
[1] "D1_myc_Sil - Eighth_myc_Sil"
[1] "D2_myc_Sil - D1_myc_Sil"
[1] "D3_myc_Sil - D2_myc_Sil"
[1] "D6_myc_Sil - D3_myc_Sil"
```

In [42]:

```
gene2cpms = dict((i[0],[float(j) for j in i[1:]]) for i in Table.fromCsv("limma1.countscpm.cpm.csv"))
len(gene2cpms), len(ct_counts)
```

Out[42]:

```
(8151, 8151)
```

In [43]:

```
limma1_cdt = CdtFile.fromPrototype(ct_counts, 
                                   probes = [CdtRow.fromPrototype(i, ratios = gene2cpms[i.Uniqid()][:])
                                             for i in ct_counts])
limma1_cdt = limma1_cdt.mean_normalize_rows()
```

In [44]:

```
#Generate columns indicating if a comparison is significant (2-fold cutoff and 5% FDR)

gene2contrasts = dict((i.Uniqid(),[]) for i in limma1_cdt)
gene2pvals = dict((i.Uniqid(),[]) for i in limma1_cdt)
gene2sigs = dict((i.Uniqid(),[]) for i in limma1_cdt)
contrast_names = []

contrast_csvs = sorted(glob("limma1.*.t1.csv"))
# put 8 hour first
contrast_csvs = contrast_csvs[-1:]+contrast_csvs[:-1]
for i in contrast_csvs:
    cname = i.replace("limma1.","").replace(".t1.csv","").replace("-","/")
    contrast_names.append(cname)
    siglist = set(i[0] for i in Table.fromCsv(i.replace(".t1.",".t0.")))
    print(cname,len(siglist))
    for gene in Table.fromCsv(i):
        name = gene[0]
        lfc = float(gene["logFC"])
        gene2contrasts[name].append(lfc)
        gene2pvals[name].append(gene["adj.P.Val"])
        if(name in siglist):
            if(lfc > 0):
                gene2sigs[name].append(4.)
            else:
                gene2sigs[name].append(-4.)
        else:
            gene2sigs[name].append(0.)
        
limma1_cdt = CdtFile.fromPrototype(limma1_cdt,
    probes = [CdtRow.fromPrototype(i, ratios = i.ratios+gene2contrasts[i.Uniqid()]+gene2sigs[i.Uniqid()],
                                   extra = i.extra+gene2pvals[i.Uniqid()])
              for i in limma1_cdt],
    fieldnames = limma1_cdt.fieldnames+contrast_names+["%s_sig" % i for i in contrast_names],
    eweights = limma1_cdt.eweights+[1.]*2*len(contrast_names),
    extranames = limma1_cdt.extranames+["p(%s)" % i for i in contrast_names])
```

```
Spores_Sil/Spores_Ryp1 3561
D1_myc_Ryp1/Spores_Ryp1 3330
D1_myc_Sil/D1_myc_Ryp1 2255
D1_myc_Sil/Eighth_myc_Sil 1575
D1_myc_Sil/Spores_Sil 4241
D2_myc_Ryp1/Spores_Ryp1 3899
D2_myc_Sil/D1_myc_Sil 1651
D2_myc_Sil/D2_myc_Ryp1 1752
D2_myc_Sil/Spores_Sil 4255
D3_myc_Ryp1/Spores_Ryp1 3621
D3_myc_Sil/D2_myc_Sil 257
D3_myc_Sil/D3_myc_Ryp1 1348
D3_myc_Sil/Spores_Sil 4294
D6_myc_Ryp1/Spores_Ryp1 3553
D6_myc_Sil/D3_myc_Sil 112
D6_myc_Sil/D6_myc_Ryp1 918
D6_myc_Sil/Spores_Sil 4161
Eighth_myc_Ryp1/Spores_Ryp1 3079
Eighth_myc_Sil/Eighth_myc_Ryp1 2552
Eighth_myc_Sil/Spores_Sil 3747
```

In [45]:

```
limma1_cdt = CdtFile.fromPrototype(limma1_cdt, 
    probes = [CdtRow.fromPrototype(i, extra = i.extra + [str(j) for j in ct_counts.GetUid(i.Uniqid())])
              for i in limma1_cdt],
    extranames = limma1_cdt.extranames+["%s_counts" % i for i in ct_counts.fieldnames])
```

In [46]:

```
#assign count columns and cluster columns
count_cols = [n for (n,i) in enumerate(limma1_cdt.fieldnames) if("/" not in i)]
print(count_cols)
#cluster on count columns
tree = limma1_cdt.cluster(cols=count_cols,dist="u",method="m")
len(limma1_cdt)
```

```
[0, 1, 2, 3, 4, 5, 6, 7, 8, 9, 10, 11, 12, 13, 14, 15, 16, 17, 18, 19, 20, 21, 22, 23, 24, 25, 26, 27, 28, 29, 30, 31, 32, 33, 34, 35]
```

```
Building array...
Building distance matrix...
Clustering...
```

Out[46]:

```
8151
```

In [47]:

```
limma1_cdt = annotate(limma1_cdt, warn = False, map_from="V3")
```

```
Warning, didn't find a GID column!  Sharing UID with GID.
```

In [48]:

```
limma1_cdt.writeCdtGtr("limma1.countscutoff.cdt", tree)
#This spreadsheet was used for heatmap in Figure 2F
```
