# Supplementary material for: Transcriptomic atlas throughout Coccidioides development reveals key phase-enriched transcripts of this important fungal pathogen
Source: PLoS Biol. 2025 Apr 15;23(4):e3003066. doi: 10.1371/journal.pbio.3003066 (PMC12077801; doi:10.1371/journal.pbio.3003066)
Supplement: S1 Code — Folder containing README document describing the scripts used to analyze the data and generate figures in this manuscript, as well as the scripts themselves and custom python three modules used in the scripts. (ZIP) [file pbio.3003066.s025.zip › Custom Code/notebooks/Fig5.html]

Fig5


In [1]:

```
%cd ../../Papers/Cocci_transcriptomics/data_for_code/Fig5/
```

```
/home/chomer/Papers/Cocci_transcriptomics/data_for_code/Fig5
```

In [2]:

```
from LimmaTools import SingleFactorFit
from CdtFile import CdtFile, CdtRow
%load_ext rpy2.ipython
from glob import glob
from MsvUtil import Table
import os.path
from SafeMath import safelog
from ReadCountTools import PseudoCPMs
from csv import writer, excel_tab
from CdtAnnotator3 import annotate_CpSilveira as annotate
from MySQLGenomeFactory import MySQLGenomeFactory
from annotate_mysql import Annotation
```

# Adding expression data for Coccidioides Transcription Factors¶

Generated 'manual\_annotation\_Cocci\_TFs.cdt' using PFAM hits as described in methods section 'Generating a list of candidate transcription factors'.

In [3]:

```
manual_cocci_TFs = CdtFile.fromCdt("../manual_annotation_Cocci_TFs.cdt")
```

In [4]:

```
#Bring in expression data for each transcription factor
Fig2 = SingleFactorFit.fromHDF5("../Fig2/Combined/Fig3_Singlecomp_limma1.hdf5").toCdtFile(obs = False, parameters = True).mean_normalize_rows()
len(Fig2)
```

Out[4]:

```
8186
```

In [5]:

```
probes = []
count = 0
for row in manual_cocci_TFs:
    try:
        ratios = Fig2.GetUid(row.extra[1])[:]
    except KeyError:
        count += 1
        ratios = [None]*len(Fig2.fieldnames)
    probes.append(CdtRow.fromPrototype(row, ratios=ratios))
cocci_TFs = CdtFile.fromPrototype(manual_cocci_TFs, probes = probes,
                                                  fieldnames = Fig2.fieldnames[:],
                                                  eweights = Fig2.eweights[:])
```

In [6]:

```
tree = cocci_TFs.cluster(dist="u", method="m")
```

```
Building array...
Building distance matrix...
Clustering...
```

In [7]:

```
cocci_TFs.writeCdtGtr("cocci_TFs.um",tree)
```

In [8]:

```
len(cocci_TFs)
#basis for heatmap in Figure 5A after manually editing the columns to only include counts for each transcript and then clustering again
```

Out[8]:

```
227
```

# Generating Endospore-associated transcript heatmap¶

In [9]:

```
%%R
library(limma)
library(edgeR)
```

## Merge Kallisto TPMs from Fig 1 and Fig 2¶

In [10]:

```
#Fig1
datadir = "../Fig1/"

sname2kallisto = dict(
    ("_".join(i.replace(".Silv_nanopore_mRNA.rf.kallisto","").replace(datadir,"").split("_")[0:-1]),i)
              for i in glob(datadir+"*.Silv_nanopore_mRNA.rf.kallisto") if "Spores_" not in i)


#Fig2
datadir = "../Fig2/"

for i in glob(datadir+"*.Silv_nanopore_mRNA.rf.kallisto"):
        sname = "_".join(i.replace(".Silv_nanopore_mRNA.rf.kallisto","").replace(datadir,"").split("_")[0:-3])
        sname2kallisto[sname] = i

snames = sorted(sname2kallisto, key = lambda x: (x[0:x.rfind("_")],x[x.rfind("_"):]), reverse=False)
```

In [11]:

```
#Merge Step
genes = None
cols = []
counts = []
for i in snames:
    table = Table.fromTdt(open(os.path.join(
           sname2kallisto[i],
          "abundance.tsv")))
    if(genes is None):
        genes = table["target_id"]
    else:
        assert(genes == table["target_id"])
    cols.append([float(i) for i in table["tpm"]])
    counts.append([int(float(i)+.5) for i in table["est_counts"]])

tpm_trans = CdtFile(probes = [CdtRow(gid = i[0], uniqid = i[0], name = i[0],
                                        ratios = [safelog(j) for j in i[1:]])
                                 for i in zip(*([genes]+cols))],
                       fieldnames = snames,
                       eweights = [1]*len(snames))
tpm_trans.write(open("tpm_trans.cdt","w"))

trans_counts = CdtFile(probes = [CdtRow(gid = i[0], uniqid = i[0], name = i[0],
                                        ratios = i[1:])
                                 for i in zip(*([genes]+counts))],
                       fieldnames = snames,
                       eweights = [1]*len(snames))
trans_counts.write(open("trans_counts.cdt","w"))

len(tpm_trans), len(trans_counts)
```

Out[11]:

```
(8628, 8628)
```

In [12]:

```
merge_pseudo = PseudoCPMs.fromCounts(trans_counts)
#pseudoCPMs are log transformed
#counts are to feed directly to limma
```

In [13]:

```
#filter out transcripts that do not have at least 10 reads in at least 1 or more datasets
mask_10 = merge_pseudo.depth_filter_mask(10,.01)
mask_10.counts.write(open("Fig5b_counts_pseudoCPMs_for_paper.cdt", "w"))
```

In [14]:

```
ct_counts = CdtFile.fromCdt("Fig5b_counts_pseudoCPMs_for_paper.cdt")
len(ct_counts)
```

Out[14]:

```
8299
```

## Limma analysis¶

In [15]:

```
#need to format ct_tpm for limma input
fout = open("ct_counts.txt", "w")

fout.write("\t".join(["gene"]+ct_counts.fieldnames)+"\n")
for row in mask_10.counts:
    fout.write("\t".join([row.Uniqid()]+[str(i) for i in row])+"\n")
fout.close()
```

In [16]:

```
%%R
#Limma Single Factor Fit
# Read the count matrix, using the gene column as row names
C <- read.delim("ct_counts.txt",row.names=1)
#Convert the matrix to limma's preferred format, implicitly log2 transforming and depth normalizing to CPM values
dge <- DGEList(counts=C)
```

In [17]:

```
out = writer(open("Fig5b_limma_simple_comp_samples.txt","w"),dialect = excel_tab)
out.writerow(("run","state"))
for i in snames:
    run = i
    if len(i) == 7:
        state = "Spores_Sil_CHAS01"
    elif "8h" in i:
        state = "Eighth_CHAS01"
    elif "-" in i:
        state = i.split("-")[0]
    else:
        state = "_".join(i.split("_")[0:-1])
    out.writerow((run, state))   
del out
```

In [18]:

```
%%R -o d
samples <- read.delim("Fig5b_limma_simple_comp_samples.txt")
print(summary(samples))
state <- samples$state
d <- model.matrix(~0+state)
colnames(d) <- gsub("state","",colnames(d))
print(colnames(d))
```

```
     run               state          
 Length:108         Length:108        
 Class :character   Class :character  
 Mode  :character   Mode  :character  
 [1] "D1_myc_Ryp1"          "D1_myc_Sil"           "D1_spherule_Ryp1"    
 [4] "D1_spherule_Sil"      "D2_myc_Ryp1"          "D2_myc_Sil"          
 [7] "D2_spherule_Ryp1"     "D2_spherule_Sil"      "D3_myc_Ryp1"         
[10] "D3_myc_Sil"           "D3_spherules_Ryp1"    "D3_spherules_Sil"    
[13] "D4_spherules_Ryp1"    "D4_spherules_Sil"     "D5_spherules_Ryp1"   
[16] "D5_spherules_Sil"     "D6_myc_Ryp1"          "D6_myc_Sil"          
[19] "D6_spherules_Ryp1"    "D6_spherules_Sil"     "Day1"                
[22] "Day2"                 "Day3"                 "Day4"                
[25] "Day5"                 "Day6"                 "DMEM"                
[28] "Eighth_CHAS01"        "Eighth_myc_Ryp1"      "Eighth_myc_Sil"      
[31] "Eighth_spherule_ryp1" "Eighth_spherule_Sil"  "RPMI"                
[34] "Spores_Ryp1"          "Spores_Sil"           "Spores_Sil_CHAS01"
```

In [19]:

```
%%R
# Apply between-sample TMM normalization
dge <- calcNormFactors(dge)
# Estimate the mean-variance trend via locally-linear regression and use this trend
# to assign weights to the observations (counts)
v <- voom(dge, d, plot = TRUE)
cpm <- v$E
```

In [20]:

```
%%R -o cpm,fc,cn,state
# Fit the model (classic linear regression)
fit <- lmFit(v, d)
#Generate the contrast matrix
contrast.matrix <- makeContrasts(
    DMEM - Spores_Sil, DMEM - D2_spherule_Sil, DMEM - D1_spherule_Sil, DMEM - RPMI, DMEM - D2_myc_Sil, DMEM - D1_myc_Sil,
    Day3-Day2, Day4-Day2, Day5-Day2, Day6-Day2,
    Day3-Day1, Day4-Day1, Day5-Day1, Day6-Day1,
    levels=d)
# Apply the contrast matrix
fit2 <- contrasts.fit(fit, contrast.matrix)
# Apply Empirical Bayes "shrinkage"
fit2 <- eBayes(fit2)
# Simple summary of significantly differential genes with no fold change filter
print(summary(decideTests(fit2)))

fc <- fit$coefficients
cn <- colnames(fit$coefficients)
cpm <- v$E
```

```
       DMEM - Spores_Sil DMEM - D2_spherule_Sil DMEM - D1_spherule_Sil
Down                2527                   1967                   2111
NotSig              2442                   4461                   4157
Up                  3330                   1871                   2031
       DMEM - RPMI DMEM - D2_myc_Sil DMEM - D1_myc_Sil Day3 - Day2 Day4 - Day2
Down          2146              1780              2169         649         514
NotSig        4025              4675              3835        6839        7125
Up            2128              1844              2295         811         660
       Day5 - Day2 Day6 - Day2 Day3 - Day1 Day4 - Day1 Day5 - Day1 Day6 - Day1
Down          1411        1386        1545        1394        1835        1765
NotSig        5648        5516        5216        5343        4681        4699
Up            1240        1397        1538        1562        1783        1835
```

In [21]:

```
name2row = dict((i.Uniqid(),n+1) for (n,i) in enumerate(ct_counts))
```

In [22]:

```
name2row = dict((i.Uniqid(),n+1) for (n,i) in enumerate(ct_counts))
fit = SingleFactorFit(fc, cpm, name2row, cn, 
                      state, obs_samples = ct_counts.fieldnames, 
                      parameter_order = ("Spores_Sil","Eighth_spherule_Sil","D1_spherule_Sil", "D2_spherule_Sil", "D3_spherules_Sil", "D4_spherules_Sil", "D5_spherules_Sil", "D6_spherules_Sil", "Spores_Ryp1","Eighth_spherule_ryp1","D1_spherule_Ryp1", "D2_spherule_Ryp1", "D3_spherules_Ryp1", "D4_spherules_Ryp1", "D5_spherules_Ryp1", "D6_spherules_Ryp1", "DMEM", "RPMI", "Eighth_myc_Sil","D1_myc_Sil", "D2_myc_Sil", "D3_myc_Sil", "D6_myc_Sil", "Eighth_myc_Ryp1","D1_myc_Ryp1", "D2_myc_Ryp1", "D3_myc_Ryp1", "D6_myc_Ryp1"))
fit.toHDF5("Fig5b_singlecomp_limma1.hdf5")
fit2 = SingleFactorFit.fromHDF5("Fig5b_singlecomp_limma1.hdf5")
```

In [23]:

```
ct_counts.mean_normalize_rows().bicluster("ct_tpm.norm.um",dist="u",method="m")
```

```
Building array...
Building distance matrix...
Clustering...
```

In [24]:

```
%%R
write.csv(cpm,"limma1.countscutoff.cpm.csv")
```

In [25]:

```
%%R
for(tc in colnames(fit2$coefficients)){
  print(tc)
  # Extract all genes significantly differential on this contrast for a 2x fold change cutoff and 5% FDR
  # Use write.csv rather than write.table for clean compatibility with python's csv.reader
  write.csv(topTable(fit2, coef=tc, n = 50000, lfc=1, p.value = .05),
            paste("limma1.",gsub(" ","",tc),".t0.csv",sep=""))
  # Extract the adjusted p-values for this contrast for all genes, independent of significance
  write.csv(topTable(fit2, coef=tc, n = 50000),
            paste("limma1.",gsub(" ","",tc),".t1.csv",sep=""))
}
```

```
[1] "DMEM - Spores_Sil"
[1] "DMEM - D2_spherule_Sil"
[1] "DMEM - D1_spherule_Sil"
[1] "DMEM - RPMI"
[1] "DMEM - D2_myc_Sil"
[1] "DMEM - D1_myc_Sil"
[1] "Day3 - Day2"
[1] "Day4 - Day2"
[1] "Day5 - Day2"
[1] "Day6 - Day2"
[1] "Day3 - Day1"
[1] "Day4 - Day1"
[1] "Day5 - Day1"
[1] "Day6 - Day1"
```

In [26]:

```
gene2cpms = dict((i[0],[float(j) for j in i[1:]]) for i in Table.fromCsv("limma1.countscutoff.cpm.csv"))
len(gene2cpms), len(ct_counts)
```

Out[26]:

```
(8299, 8299)
```

In [27]:

```
limma1_cdt = CdtFile.fromPrototype(ct_counts, 
                                   probes = [CdtRow.fromPrototype(i, ratios = gene2cpms[i.Uniqid()][:])
                                             for i in ct_counts])
limma1_cdt = limma1_cdt.mean_normalize_rows()
```

In [28]:

```
gene2contrasts = dict((i.Uniqid(),[]) for i in limma1_cdt)
gene2pvals = dict((i.Uniqid(),[]) for i in limma1_cdt)
gene2sigs = dict((i.Uniqid(),[]) for i in limma1_cdt)
contrast_names = []

contrast_csvs = sorted(glob("limma1.*.t1.csv"))
contrast_csvs = contrast_csvs[-1:]+contrast_csvs[:-1]
for i in contrast_csvs:
    cname = i.replace("limma1.","").replace(".t1.csv","").replace("-","/")
    contrast_names.append(cname)
    siglist = set(i[0] for i in Table.fromCsv(i.replace(".t1.",".t0.")))
    print(cname,len(siglist))
    for gene in Table.fromCsv(i):
        name = gene[0]
        lfc = float(gene["logFC"])
        gene2contrasts[name].append(lfc)
        gene2pvals[name].append(gene["adj.P.Val"])
        if(name in siglist):
            if(lfc > 0):
                gene2sigs[name].append(4.)
            else:
                gene2sigs[name].append(-4.)
        else:
            gene2sigs[name].append(0.)
        
limma1_cdt = CdtFile.fromPrototype(limma1_cdt,
    probes = [CdtRow.fromPrototype(i, ratios = i.ratios+gene2contrasts[i.Uniqid()]+gene2sigs[i.Uniqid()],
                                   extra = i.extra+gene2pvals[i.Uniqid()])
              for i in limma1_cdt],
    fieldnames = limma1_cdt.fieldnames+contrast_names+["%s_sig" % i for i in contrast_names],
    eweights = limma1_cdt.eweights+[1.]*2*len(contrast_names),
    extranames = limma1_cdt.extranames+["p(%s)" % i for i in contrast_names])
```

```
Day6/Day2 1642
DMEM/D1_myc_Sil 2526
DMEM/D1_spherule_Sil 2211
DMEM/D2_myc_Sil 1871
DMEM/D2_spherule_Sil 2300
DMEM/RPMI 2295
DMEM/Spores_Sil 4534
Day3/Day1 1357
Day3/Day2 561
Day4/Day1 1418
Day4/Day2 600
Day5/Day1 1873
Day5/Day2 1160
Day6/Day1 2313
```

In [29]:

```
limma1_cdt = CdtFile.fromPrototype(limma1_cdt, 
    probes = [CdtRow.fromPrototype(i, extra = i.extra + [str(j) for j in ct_counts.GetUid(i.Uniqid())])
              for i in limma1_cdt],
    extranames = limma1_cdt.extranames+["%s_counts" % i for i in ct_counts.fieldnames])
```

In [30]:

```
limma1_cdt = annotate(limma1_cdt, warn = False, map_from="V3")
```

```
Warning, didn't find a GID column!  Sharing UID with GID.
```

In [31]:

```
limma1_cdt.write(open("limma1.countscutoff.cdt","w"))
```

In [32]:

```
sig_cols = [n for (n,i) in enumerate(limma1_cdt.fieldnames) if(i.endswith("_sig"))]
contrast_cols = [n-len(sig_cols) for n in sig_cols]
limma1_sig= CdtFile.fromPrototype(limma1_cdt, probes = [i for i in limma1_cdt 
                                                        if(any([(i[j] != 0.) for j in sig_cols]))])
tree = limma1_sig.cluster(cols=contrast_cols,dist="u",method="m")
limma1_sig.writeCdtGtr("limma1_sig.countscutoff.contrasts_um",tree)
len(limma1_sig)
```

```
Building array...
Building distance matrix...
Clustering...
```

Out[32]:

```
7031
```

## Making comparisons to define endospore-associated transcripts¶

In [33]:

```
sig_cols_endospores = [] 
for n,i in (enumerate(limma1_sig.fieldnames)):
    if i.endswith("_sig"):
        if "DMEM" in i:
            if "D2" in i:
                print(i)
                sig_cols_endospores.append(n)
            elif "D1" in i:
                print(i)
                sig_cols_endospores.append(n)
            elif "RPMI" in i:
                print(i)
                sig_cols_endospores.append(n)
            elif "Spores" in i:
                print(i)
                sig_cols_endospores.append(n)
        elif "Day" in i.split("/")[1]:
            print(i)
            sig_cols_endospores.append(n)
```

```
Day6/Day2_sig
DMEM/D1_myc_Sil_sig
DMEM/D1_spherule_Sil_sig
DMEM/D2_myc_Sil_sig
DMEM/D2_spherule_Sil_sig
DMEM/RPMI_sig
DMEM/Spores_Sil_sig
Day3/Day1_sig
Day3/Day2_sig
Day4/Day1_sig
Day4/Day2_sig
Day5/Day1_sig
Day5/Day2_sig
Day6/Day1_sig
```

In [34]:

```
#Basis for Fig 5B (top aspect - manually reordered columns and clustered)
sig_cols = [n for (n,i) in enumerate(limma1_sig.fieldnames) if(i.endswith("_sig"))]
contrast_cols = [n-len(sig_cols) for n in sig_cols]
limma1_endospore_pos = CdtFile.fromPrototype(limma1_sig, probes = [i for i in limma1_sig 
                                                        if(all([(i[j] == 4.0) for j in sig_cols_endospores]))])
tree = limma1_endospore_pos.cluster(cols=contrast_cols,dist="u",method="m")
limma1_endospore_pos.writeCdtGtr("limma1_endospore_pos.contrasts_um",tree)
len(limma1_endospore_pos)
```

```
Building array...
Building distance matrix...
Clustering...
```

Out[34]:

```
18
```

In [35]:

```
#Basis for Fig 5B (bottom aspect - manually reordered columns and clustered)
limma1_endospore_neg = CdtFile.fromPrototype(limma1_sig, probes = [i for i in limma1_sig 
                                                        if(all([(i[j] == -4.0) for j in sig_cols_endospores]))])
tree = limma1_endospore_neg.cluster(cols=contrast_cols,dist="u",method="m")
#limma1_endospore_neg = annotate(limma1_endospore_neg, map_from="V3")
limma1_endospore_neg.writeCdtGtr("limma1_endospore_neg.contrasts_um",tree)
len(limma1_endospore_neg)
```

```
Building array...
Building distance matrix...
Clustering...
```

Out[35]:

```
2
```

# Defining Candidate Effectors¶

In [36]:

```
f = MySQLGenomeFactory(assembly_caching = "eager", db = "Genome4")
genome = f.getGenome("CpSilveiraV3")
genes = [gene for (name,gene) in genome.Genes()]
```

### Get cysteine content and length of predicted proteins¶

In [37]:

```
probes = []
for (name, gene) in genome.Genes():
    seq = str(gene.ProteinSequence())
    if(len(seq) < 1):
        continue
    ratios = [seq.count("C"), len(seq)]
    gene_name = name
    probes.append(CdtRow(gid = gene.Name(), uniqid = gene.Name(), name = gene_name, ratios = ratios))
```

In [38]:

```
cys_cdt = CdtFile(probes=probes, fieldnames = ["CysCount", "AAlen"] )
cys_cdt = annotate(cys_cdt, map_from="V3")
```

```
Warning, didn't find a GID column!  Sharing UID with GID.
Unmapped UID: D8B26_001639
Unmapped UID: D8B26_003812
Unmapped UID: D8B26_000716
Unmapped UID: D8B26_000383
Unmapped UID: D8B26_006187
Unmapped UID: D8B26_000958
Unmapped UID: D8B26_005671
Unmapped UID: D8B26_001076
Unmapped UID: D8B26_004060
Unmapped UID: D8B26_007127
Unmapped UID: D8B26_004924
Unmapped UID: D8B26_006745
Unmapped UID: D8B26_004849
Unmapped UID: D8B26_006976
Unmapped UID: D8B26_006455
Unmapped UID: D8B26_002115
Unmapped UID: D8B26_005518
Unmapped UID: D8B26_002023
Unmapped UID: D8B26_003203
Unmapped UID: D8B26_001960
Unmapped UID: D8B26_007587
Unmapped UID: D8B26_007009
Unmapped UID: D8B26_005189
Unmapped UID: D8B26_007650
Unmapped UID: D8B26_000142
Unmapped UID: D8B26_008386
Unmapped UID: D8B26_006600
Unmapped UID: D8B26_005299
Unmapped UID: D8B26_000620
Unmapped UID: D8B26_000332
Unmapped UID: D8B26_007105
Unmapped UID: D8B26_002571
Unmapped UID: D8B26_008412
Unmapped UID: D8B26_000258
Unmapped UID: D8B26_001664
Unmapped UID: D8B26_000877
Unmapped UID: D8B26_007158
Unmapped UID: D8B26_005488
Unmapped UID: D8B26_003453
Unmapped UID: D8B26_007112
Unmapped UID: D8B26_004920
Unmapped UID: D8B26_005832
Unmapped UID: D8B26_005386
Unmapped UID: D8B26_007982
Unmapped UID: D8B26_005835
Unmapped UID: D8B26_006823
Unmapped UID: D8B26_008049
Unmapped UID: D8B26_004406
Unmapped UID: D8B26_005847
Unmapped UID: D8B26_001941
Unmapped UID: D8B26_007918
Unmapped UID: D8B26_001850
Unmapped UID: D8B26_001622
Unmapped UID: D8B26_001419
Unmapped UID: D8B26_001898
Unmapped UID: D8B26_004201
Unmapped UID: D8B26_007202
Unmapped UID: D8B26_007498
Unmapped UID: D8B26_004310
Unmapped UID: D8B26_001936
Unmapped UID: D8B26_005850
Unmapped UID: D8B26_000293
Unmapped UID: D8B26_005910
Unmapped UID: D8B26_008222
Unmapped UID: D8B26_001973
Unmapped UID: D8B26_000667
Unmapped UID: D8B26_002870
Unmapped UID: D8B26_002871
Unmapped UID: D8B26_002479
Unmapped UID: D8B26_008381
Unmapped UID: D8B26_003233
Unmapped UID: D8B26_004222
Unmapped UID: D8B26_007087
Unmapped UID: D8B26_002194
Unmapped UID: D8B26_004922
Unmapped UID: D8B26_004755
Unmapped UID: D8B26_001890
Unmapped UID: D8B26_008129
Unmapped UID: D8B26_000076
Unmapped UID: D8B26_003563
Unmapped UID: D8B26_001347
Unmapped UID: D8B26_006986
Unmapped UID: D8B26_001640
Unmapped UID: D8B26_005341
Unmapped UID: D8B26_000666
Unmapped UID: D8B26_002868
Unmapped UID: D8B26_000239
Unmapped UID: D8B26_007834
Unmapped UID: D8B26_007038
Unmapped UID: D8B26_001782
Unmapped UID: D8B26_007159
Unmapped UID: D8B26_000591
Unmapped UID: D8B26_007067
Unmapped UID: D8B26_004845
Unmapped UID: D8B26_003247
Unmapped UID: D8B26_005187
Unmapped UID: D8B26_007190
Unmapped UID: D8B26_007497
Unmapped UID: D8B26_000855
Unmapped UID: D8B26_005214
Unmapped UID: D8B26_005066
Unmapped UID: D8B26_005374
Unmapped UID: D8B26_002530
Unmapped UID: D8B26_000553
Unmapped UID: D8B26_004135
Unmapped UID: D8B26_005620
Unmapped UID: D8B26_001780
Unmapped UID: D8B26_005912
Unmapped UID: D8B26_005000
Unmapped UID: D8B26_002230
Unmapped UID: D8B26_007991
Unmapped UID: D8B26_004417
Unmapped UID: D8B26_000882
Unmapped UID: D8B26_000392
Unmapped UID: D8B26_005447
Unmapped UID: D8B26_005450
Unmapped UID: D8B26_008392
Unmapped UID: D8B26_001088
Unmapped UID: D8B26_003606
Unmapped UID: D8B26_007491
Unmapped UID: D8B26_004390
Unmapped UID: D8B26_003750
Unmapped UID: D8B26_006644
Unmapped UID: D8B26_005224
Unmapped UID: D8B26_004419
Unmapped UID: D8B26_002228
Unmapped UID: D8B26_008394
Unmapped UID: D8B26_002107
Unmapped UID: D8B26_004193
Unmapped UID: D8B26_006075
Unmapped UID: D8B26_007919
Unmapped UID: D8B26_008404
Unmapped UID: D8B26_006873
Unmapped UID: D8B26_005900
Unmapped UID: D8B26_008431
Unmapped UID: D8B26_002903
Unmapped UID: D8B26_005315
Unmapped UID: D8B26_005909
Unmapped UID: D8B26_001007
Unmapped UID: D8B26_007140
Unmapped UID: D8B26_007586
Unmapped UID: D8B26_002024
Unmapped UID: D8B26_000112
Unmapped UID: D8B26_001955
Unmapped UID: D8B26_003636
Unmapped UID: D8B26_002460
Unmapped UID: D8B26_001129
Unmapped UID: D8B26_004197
Unmapped UID: D8B26_000954
Unmapped UID: D8B26_005911
Unmapped UID: D8B26_006626
Unmapped UID: D8B26_000717
Unmapped UID: D8B26_005892
Unmapped UID: D8B26_003830
Unmapped UID: D8B26_000949
Unmapped UID: D8B26_000437
Unmapped UID: D8B26_008274
Unmapped UID: D8B26_007187
Unmapped UID: D8B26_007889
Unmapped UID: D8B26_008384
Unmapped UID: D8B26_006645
Unmapped UID: D8B26_002548
Unmapped UID: D8B26_003419
Unmapped UID: D8B26_008393
Unmapped UID: D8B26_003721
Unmapped UID: D8B26_005199
Unmapped UID: D8B26_001904
Unmapped UID: D8B26_004149
Unmapped UID: D8B26_008409
Unmapped UID: D8B26_004378
Unmapped UID: D8B26_005376
Unmapped UID: D8B26_006939
Unmapped UID: D8B26_005037
Unmapped UID: D8B26_006186
Unmapped UID: D8B26_001619
Unmapped UID: D8B26_007194
Unmapped UID: D8B26_006567
Unmapped UID: D8B26_007141
Unmapped UID: D8B26_007602
Unmapped UID: D8B26_000778
Unmapped UID: D8B26_004919
Unmapped UID: D8B26_006946
Unmapped UID: D8B26_002778
Unmapped UID: D8B26_000168
Unmapped UID: D8B26_008396
Unmapped UID: D8B26_007238
Unmapped UID: D8B26_007440
Unmapped UID: D8B26_006931
Unmapped UID: D8B26_001794
Unmapped UID: D8B26_008252
Unmapped UID: D8B26_000227
Unmapped UID: D8B26_000759
Unmapped UID: D8B26_005621
Unmapped UID: D8B26_002553
Unmapped UID: D8B26_005276
Unmapped UID: D8B26_007786
Unmapped UID: D8B26_005059
Unmapped UID: D8B26_007986
Unmapped UID: D8B26_000662
Unmapped UID: D8B26_002846
Unmapped UID: D8B26_004244
Unmapped UID: D8B26_007728
Unmapped UID: D8B26_005784
Unmapped UID: D8B26_007897
Unmapped UID: D8B26_003915
Unmapped UID: D8B26_008058
Unmapped UID: D8B26_004221
Unmapped UID: D8B26_007782
Unmapped UID: D8B26_000414
Unmapped UID: D8B26_004423
Unmapped UID: D8B26_002748
Unmapped UID: D8B26_004999
Unmapped UID: D8B26_002466
Unmapped UID: D8B26_007404
Unmapped UID: D8B26_002108
Unmapped UID: D8B26_000002
Unmapped UID: D8B26_005563
Unmapped UID: D8B26_002777
Unmapped UID: D8B26_005670
Unmapped UID: D8B26_004879
Unmapped UID: D8B26_006901
Unmapped UID: D8B26_002469
Unmapped UID: D8B26_005500
Unmapped UID: D8B26_001942
Unmapped UID: D8B26_002500
Unmapped UID: D8B26_001135
Unmapped UID: D8B26_006599
Unmapped UID: D8B26_007333
Unmapped UID: D8B26_004765
Unmapped UID: D8B26_004298
Unmapped UID: D8B26_007007
Unmapped UID: D8B26_004843
Unmapped UID: D8B26_007191
Unmapped UID: D8B26_008401
Unmapped UID: D8B26_007947
Unmapped UID: D8B26_007030
Unmapped UID: D8B26_003612
Unmapped UID: D8B26_002730
Unmapped UID: D8B26_003375
Unmapped UID: D8B26_008410
Unmapped UID: D8B26_006722
Unmapped UID: D8B26_001185
Unmapped UID: D8B26_004203
Unmapped UID: D8B26_007122
Unmapped UID: D8B26_008261
Unmapped UID: D8B26_008376
Unmapped UID: D8B26_006201
Unmapped UID: D8B26_003989
Unmapped UID: D8B26_005371
Unmapped UID: D8B26_004146
Unmapped UID: D8B26_006717
Unmapped UID: D8B26_004882
Unmapped UID: D8B26_000484
Unmapped UID: D8B26_006432
Unmapped UID: D8B26_001434
Unmapped UID: D8B26_004271
Unmapped UID: D8B26_008284
Unmapped UID: D8B26_007974
Unmapped UID: D8B26_005244
Unmapped UID: D8B26_002225
Unmapped UID: D8B26_002540
Unmapped UID: D8B26_005960
Unmapped UID: D8B26_007836
Unmapped UID: D8B26_006625
Unmapped UID: D8B26_005300
Unmapped UID: D8B26_002899
Unmapped UID: D8B26_008406
Unmapped UID: D8B26_006768
Unmapped UID: D8B26_001465
Unmapped UID: D8B26_001571
Unmapped UID: D8B26_008060
Unmapped UID: D8B26_005392
Unmapped UID: D8B26_008417
Unmapped UID: D8B26_000719
Unmapped UID: D8B26_001455
Unmapped UID: D8B26_004199
Unmapped UID: D8B26_008253
Unmapped UID: D8B26_006942
Unmapped UID: D8B26_000091
Unmapped UID: D8B26_005481
Unmapped UID: D8B26_005818
Unmapped UID: D8B26_000355
Unmapped UID: D8B26_001555
Unmapped UID: D8B26_007169
Unmapped UID: D8B26_006748
Unmapped UID: D8B26_004541
Unmapped UID: D8B26_003360
Unmapped UID: D8B26_000953
Unmapped UID: D8B26_006431
Unmapped UID: D8B26_000485
Unmapped UID: D8B26_002577
Unmapped UID: D8B26_005963
Unmapped UID: D8B26_007126
Unmapped UID: D8B26_006891
Unmapped UID: D8B26_007185
Unmapped UID: D8B26_005071
Unmapped UID: D8B26_003658
Unmapped UID: D8B26_000798
Unmapped UID: D8B26_002403
Unmapped UID: D8B26_006879
Unmapped UID: D8B26_000970
Unmapped UID: D8B26_005550
Unmapped UID: D8B26_002842
Unmapped UID: D8B26_008379
Unmapped UID: D8B26_000290
Unmapped UID: D8B26_007645
Unmapped UID: D8B26_003536
Unmapped UID: D8B26_007002
Unmapped UID: D8B26_007050
Unmapped UID: D8B26_003341
Unmapped UID: D8B26_007135
Unmapped UID: D8B26_003691
Unmapped UID: D8B26_005770
Unmapped UID: D8B26_001097
Unmapped UID: D8B26_007984
Unmapped UID: D8B26_003263
Unmapped UID: D8B26_006528
Unmapped UID: D8B26_000530
Unmapped UID: D8B26_008287
Unmapped UID: D8B26_007046
Unmapped UID: D8B26_006983
Unmapped UID: D8B26_004672
Unmapped UID: D8B26_008196
Unmapped UID: D8B26_008079
Unmapped UID: D8B26_002170
Unmapped UID: D8B26_005387
Unmapped UID: D8B26_004451
Unmapped UID: D8B26_005451
Unmapped UID: D8B26_000835
Unmapped UID: D8B26_002990
Unmapped UID: D8B26_007463
Unmapped UID: D8B26_007890
Unmapped UID: D8B26_004844
Unmapped UID: D8B26_003611
Unmapped UID: D8B26_000225
Unmapped UID: D8B26_006634
Unmapped UID: D8B26_004634
Unmapped UID: D8B26_000395
Unmapped UID: D8B26_001454
Unmapped UID: D8B26_007715
Unmapped UID: D8B26_000584
Unmapped UID: D8B26_007565
Unmapped UID: D8B26_004160
Unmapped UID: D8B26_000896
Unmapped UID: D8B26_005797
Unmapped UID: D8B26_007147
Unmapped UID: D8B26_006703
Unmapped UID: D8B26_003452
Unmapped UID: D8B26_003414
Unmapped UID: D8B26_005720
Unmapped UID: D8B26_005379
Unmapped UID: D8B26_002417
Unmapped UID: D8B26_000001
Unmapped UID: D8B26_003136
Unmapped UID: D8B26_004616
Unmapped UID: D8B26_002193
Unmapped UID: D8B26_004978
Unmapped UID: D8B26_000285
Unmapped UID: D8B26_008235
Unmapped UID: D8B26_007566
Unmapped UID: D8B26_002900
Unmapped UID: D8B26_006861
Unmapped UID: D8B26_000084
Unmapped UID: D8B26_005198
Unmapped UID: D8B26_008057
Unmapped UID: D8B26_007049
Unmapped UID: D8B26_005899
Unmapped UID: D8B26_006200
Unmapped UID: D8B26_002019
Unmapped UID: D8B26_007853
Unmapped UID: D8B26_004980
Unmapped UID: D8B26_007591
Unmapped UID: D8B26_006212
Unmapped UID: D8B26_002497
Unmapped UID: D8B26_001095
Unmapped UID: D8B26_004261
Unmapped UID: D8B26_006527
Unmapped UID: D8B26_007339
Unmapped UID: D8B26_006728
Unmapped UID: D8B26_007464
Unmapped UID: D8B26_004372
Unmapped UID: D8B26_004454
Unmapped UID: D8B26_003991
Unmapped UID: D8B26_007833
Unmapped UID: D8B26_001252
Unmapped UID: D8B26_007981
Unmapped UID: D8B26_007008
Unmapped UID: D8B26_002022
Unmapped UID: D8B26_007651
Unmapped UID: D8B26_005886
Unmapped UID: D8B26_006007
Unmapped UID: D8B26_002737
Unmapped UID: D8B26_000541
Unmapped UID: D8B26_000384
Unmapped UID: D8B26_003940
Unmapped UID: D8B26_003205
Unmapped UID: D8B26_005681
Unmapped UID: D8B26_004687
Unmapped UID: D8B26_005929
Unmapped UID: D8B26_002161
Unmapped UID: D8B26_003430
Unmapped UID: D8B26_005879
Unmapped UID: D8B26_007910
Unmapped UID: D8B26_001564
Unmapped UID: D8B26_001384
Unmapped UID: D8B26_001857
Unmapped UID: D8B26_005235
Unmapped UID: D8B26_008207
Unmapped UID: D8B26_006316
Unmapped UID: D8B26_003420
Unmapped UID: D8B26_003062
Unmapped UID: D8B26_006584
Unmapped UID: D8B26_002726
Unmapped UID: D8B26_005061
Unmapped UID: D8B26_003149
Unmapped UID: D8B26_002344
Unmapped UID: D8B26_002811
Unmapped UID: D8B26_006143
Unmapped UID: D8B26_002664
Unmapped UID: D8B26_005930
Unmapped UID: D8B26_005891
Unmapped UID: D8B26_001572
Unmapped UID: D8B26_007474
Unmapped UID: D8B26_002795
Unmapped UID: D8B26_000636
Unmapped UID: D8B26_000497
Unmapped UID: D8B26_004732
Unmapped UID: D8B26_001191
Unmapped UID: D8B26_002792
Unmapped UID: D8B26_000396
Unmapped UID: D8B26_004297
Unmapped UID: D8B26_001935
Unmapped UID: D8B26_003722
Unmapped UID: D8B26_000120
Unmapped UID: D8B26_006855
Unmapped UID: D8B26_003808
Unmapped UID: D8B26_006688
Unmapped UID: D8B26_001072
Unmapped UID: D8B26_007898
Unmapped UID: D8B26_000706
Unmapped UID: D8B26_007142
Unmapped UID: D8B26_008310
Unmapped UID: D8B26_002066
Unmapped UID: D8B26_004766
Unmapped UID: D8B26_006694
Unmapped UID: D8B26_008134
Unmapped UID: D8B26_004587
Unmapped UID: D8B26_005779
Unmapped UID: D8B26_001077
Unmapped UID: D8B26_007940
Unmapped UID: D8B26_000466
Unmapped UID: D8B26_005665
Unmapped UID: D8B26_000683
Unmapped UID: D8B26_000143
Unmapped UID: D8B26_005785
Unmapped UID: D8B26_003155
Unmapped UID: D8B26_005384
Unmapped UID: D8B26_004633
Unmapped UID: D8B26_002233
Unmapped UID: D8B26_007066
Unmapped UID: D8B26_002796
Unmapped UID: D8B26_005045
Unmapped UID: D8B26_007819
Unmapped UID: D8B26_004450
Unmapped UID: D8B26_007725
Unmapped UID: D8B26_004930
Unmapped UID: D8B26_006697
Unmapped UID: D8B26_002232
Unmapped UID: D8B26_003240
Unmapped UID: D8B26_007281
Unmapped UID: D8B26_004949
Unmapped UID: D8B26_007783
Unmapped UID: D8B26_007031
Unmapped UID: D8B26_004589
Unmapped UID: D8B26_006314
Unmapped UID: D8B26_008344
Unmapped UID: D8B26_001540
Unmapped UID: D8B26_005602
Unmapped UID: D8B26_001368
Unmapped UID: D8B26_000609
Unmapped UID: D8B26_001169
Unmapped UID: D8B26_001444
Unmapped UID: D8B26_005406
Unmapped UID: D8B26_003071
Unmapped UID: D8B26_007042
Unmapped UID: D8B26_007160
Unmapped UID: D8B26_005601
Unmapped UID: D8B26_006255
Unmapped UID: D8B26_006068
Unmapped UID: D8B26_005001
Unmapped UID: D8B26_005243
Unmapped UID: D8B26_003028
Unmapped UID: D8B26_000876
Unmapped UID: D8B26_007983
Unmapped UID: D8B26_000833
Unmapped UID: D8B26_005028
Unmapped UID: D8B26_000003
Unmapped UID: D8B26_001679
Unmapped UID: D8B26_000393
Unmapped UID: D8B26_007417
Unmapped UID: D8B26_000528
Unmapped UID: D8B26_002793
Unmapped UID: D8B26_004836
Unmapped UID: D8B26_005314
Unmapped UID: D8B26_006871
Unmapped UID: D8B26_002735
Unmapped UID: D8B26_002390
Unmapped UID: D8B26_000969
Unmapped UID: D8B26_004669
Unmapped UID: D8B26_000650
Unmapped UID: D8B26_007254
Unmapped UID: D8B26_005138
Unmapped UID: D8B26_004782
Unmapped UID: D8B26_002467
Unmapped UID: D8B26_002418
Unmapped UID: D8B26_007403
Unmapped UID: D8B26_006575
Unmapped UID: D8B26_004227
Unmapped UID: D8B26_002480
Unmapped UID: D8B26_004950
Unmapped UID: D8B26_007727
Unmapped UID: D8B26_005057
Unmapped UID: D8B26_007714
Unmapped UID: D8B26_006525
Unmapped UID: D8B26_001783
Unmapped UID: D8B26_007575
Unmapped UID: D8B26_002389
Unmapped UID: D8B26_000467
Unmapped UID: D8B26_005215
Unmapped UID: D8B26_000610
Unmapped UID: D8B26_008197
Unmapped UID: D8B26_006323
Unmapped UID: D8B26_000121
Unmapped UID: D8B26_000984
Unmapped UID: D8B26_006987
Unmapped UID: D8B26_007410
Unmapped UID: D8B26_005978
Unmapped UID: D8B26_007939
Unmapped UID: D8B26_007006
Unmapped UID: D8B26_007255
Unmapped UID: D8B26_003451
Unmapped UID: D8B26_000641
Unmapped UID: D8B26_008407
Unmapped UID: D8B26_007090
Unmapped UID: D8B26_005043
Unmapped UID: D8B26_002593
Unmapped UID: D8B26_008411
Unmapped UID: D8B26_000122
Unmapped UID: D8B26_000111
Unmapped UID: D8B26_008377
Unmapped UID: D8B26_001805
Unmapped UID: D8B26_000567
Unmapped UID: D8B26_003522
Unmapped UID: D8B26_002623
Unmapped UID: D8B26_000580
Unmapped UID: D8B26_001170
Unmapped UID: D8B26_008076
Unmapped UID: D8B26_004136
Unmapped UID: D8B26_007128
Unmapped UID: D8B26_000318
Unmapped UID: D8B26_005833
Unmapped UID: D8B26_005148
Unmapped UID: D8B26_004008
Unmapped UID: D8B26_000725
Unmapped UID: D8B26_004242
Unmapped UID: D8B26_006279
Unmapped UID: D8B26_002947
Unmapped UID: D8B26_007976
Unmapped UID: D8B26_008311
Unmapped UID: D8B26_002867
Unmapped UID: D8B26_003710
Unmapped UID: D8B26_004251
Unmapped UID: D8B26_007004
Unmapped UID: D8B26_003765
Unmapped UID: D8B26_000867
Unmapped UID: D8B26_006892
Unmapped UID: D8B26_002211
Unmapped UID: D8B26_004678
Unmapped UID: D8B26_003953
Unmapped UID: D8B26_005350
Unmapped UID: D8B26_008205
Unmapped UID: D8B26_002860
Unmapped UID: D8B26_005375
Unmapped UID: D8B26_005400
Unmapped UID: D8B26_008149
Unmapped UID: D8B26_004668
Unmapped UID: D8B26_004294
Unmapped UID: D8B26_006704
Unmapped UID: D8B26_000289
Unmapped UID: D8B26_007053
Unmapped UID: D8B26_001737
Unmapped UID: D8B26_001288
Unmapped UID: D8B26_000417
Unmapped UID: D8B26_003846
Unmapped UID: D8B26_006984
Unmapped UID: D8B26_003555
Unmapped UID: D8B26_004061
Unmapped UID: D8B26_004248
Unmapped UID: D8B26_004418
Unmapped UID: D8B26_000527
Unmapped UID: D8B26_002160
Unmapped UID: D8B26_007170
Unmapped UID: D8B26_002231
Unmapped UID: D8B26_002962
Unmapped UID: D8B26_003367
Unmapped UID: D8B26_002738
Unmapped UID: D8B26_005042
Unmapped UID: D8B26_001849
Unmapped UID: D8B26_006537
Unmapped UID: D8B26_005079
Unmapped UID: D8B26_005689
Unmapped UID: D8B26_001988
Unmapped UID: D8B26_004830
Unmapped UID: D8B26_007345
Unmapped UID: D8B26_003604
Unmapped UID: D8B26_001186
Unmapped UID: D8B26_007964
Unmapped UID: D8B26_004673
Unmapped UID: D8B26_001049
Unmapped UID: D8B26_002394
Unmapped UID: D8B26_000718
Unmapped UID: D8B26_006689
Unmapped UID: D8B26_005394
Unmapped UID: D8B26_005482
Unmapped UID: D8B26_006860
Unmapped UID: D8B26_004730
Unmapped UID: D8B26_008398
Unmapped UID: D8B26_004878
Unmapped UID: D8B26_007979
Unmapped UID: D8B26_008387
Unmapped UID: D8B26_003418
Unmapped UID: D8B26_000959
Unmapped UID: D8B26_001233
Unmapped UID: D8B26_001947
Unmapped UID: D8B26_006993
Unmapped UID: D8B26_007093
Unmapped UID: D8B26_004559
Unmapped UID: D8B26_006762
Unmapped UID: D8B26_004427
Unmapped UID: D8B26_008390
Unmapped UID: D8B26_004848
Unmapped UID: D8B26_003361
Unmapped UID: D8B26_007980
Unmapped UID: D8B26_004009
Unmapped UID: D8B26_005742
Unmapped UID: D8B26_002391
Unmapped UID: D8B26_004311
Unmapped UID: D8B26_008380
Unmapped UID: D8B26_004159
Unmapped UID: D8B26_003916
Unmapped UID: D8B26_007513
Unmapped UID: D8B26_004529
Unmapped UID: D8B26_005732
Unmapped UID: D8B26_004963
Unmapped UID: D8B26_002747
Unmapped UID: D8B26_003070
Unmapped UID: D8B26_001525
Unmapped UID: D8B26_005743
Unmapped UID: D8B26_005549
Unmapped UID: D8B26_006784
Unmapped UID: D8B26_003374
Unmapped UID: D8B26_003712
Unmapped UID: D8B26_002923
Unmapped UID: D8B26_005062
Unmapped UID: D8B26_007888
Unmapped UID: D8B26_002284
Unmapped UID: D8B26_007726
Unmapped UID: D8B26_004489
Unmapped UID: D8B26_004983
Unmapped UID: D8B26_005027
Unmapped UID: D8B26_007490
Unmapped UID: D8B26_005127
Unmapped UID: D8B26_004399
Unmapped UID: D8B26_004371
Unmapped UID: D8B26_000352
Unmapped UID: D8B26_006747
Unmapped UID: D8B26_006260
Unmapped UID: D8B26_008059
Unmapped UID: D8B26_003239
Unmapped UID: D8B26_006880
Unmapped UID: D8B26_007148
Unmapped UID: D8B26_002204
Unmapped UID: D8B26_005589
Unmapped UID: D8B26_007601
Unmapped UID: D8B26_001268
Unmapped UID: D8B26_000866
Unmapped UID: D8B26_006548
Unmapped UID: D8B26_001289
Unmapped UID: D8B26_006846
Unmapped UID: D8B26_007402
Unmapped UID: D8B26_000436
Unmapped UID: D8B26_007484
Unmapped UID: D8B26_002622
Unmapped UID: D8B26_008351
Unmapped UID: D8B26_007933
Unmapped UID: D8B26_004052
Unmapped UID: D8B26_008399
Unmapped UID: D8B26_007204
Unmapped UID: D8B26_002991
Unmapped UID: D8B26_005397
Unmapped UID: D8B26_003672
Unmapped UID: D8B26_001644
Unmapped UID: D8B26_002661
Unmapped UID: D8B26_005401
Unmapped UID: D8B26_008286
Unmapped UID: D8B26_001431
Unmapped UID: D8B26_004917
Unmapped UID: D8B26_001045
Unmapped UID: D8B26_004596
Unmapped UID: D8B26_005737
Unmapped UID: D8B26_000343
Unmapped UID: D8B26_005236
Unmapped UID: D8B26_007646
Unmapped UID: D8B26_004086
Unmapped UID: D8B26_005695
Unmapped UID: D8B26_005405
Unmapped UID: D8B26_005190
Unmapped UID: D8B26_006120
Unmapped UID: D8B26_008082
Unmapped UID: D8B26_006233
Unmapped UID: D8B26_007152
Unmapped UID: D8B26_007334
Unmapped UID: D8B26_007909
Unmapped UID: D8B26_003769
Unmapped UID: D8B26_008181
Unmapped UID: D8B26_002590
Unmapped UID: D8B26_001889
Unmapped UID: D8B26_006536
Unmapped UID: D8B26_004243
Unmapped UID: D8B26_001786
Unmapped UID: D8B26_007047
Unmapped UID: D8B26_008383
Unmapped UID: D8B26_004863
Unmapped UID: D8B26_004727
Unmapped UID: D8B26_004062
Unmapped UID: D8B26_002401
Unmapped UID: D8B26_005676
Unmapped UID: D8B26_004188
Unmapped UID: D8B26_005128
Unmapped UID: D8B26_001090
Unmapped UID: D8B26_000566
Unmapped UID: D8B26_005625
Unmapped UID: D8B26_003996
Unmapped UID: D8B26_000673
Unmapped UID: D8B26_002812
Unmapped UID: D8B26_000202
Unmapped UID: D8B26_006822
Unmapped UID: D8B26_006824
Unmapped UID: D8B26_004333
Unmapped UID: D8B26_007014
Unmapped UID: D8B26_005378
Unmapped UID: D8B26_008083
Unmapped UID: D8B26_008389
Unmapped UID: D8B26_004111
Unmapped UID: D8B26_002855
Unmapped UID: D8B26_005946
Unmapped UID: D8B26_003523
Unmapped UID: D8B26_002248
Unmapped UID: D8B26_002854
Unmapped UID: D8B26_007820
Unmapped UID: D8B26_008391
Unmapped UID: D8B26_008408
Unmapped UID: D8B26_007163
Unmapped UID: D8B26_006721
Unmapped UID: D8B26_004516
Unmapped UID: D8B26_001346
Unmapped UID: D8B26_000157
Unmapped UID: D8B26_007473
Unmapped UID: D8B26_008421
Unmapped UID: D8B26_002648
Unmapped UID: D8B26_005555
Unmapped UID: D8B26_004528
Unmapped UID: D8B26_006737
Unmapped UID: D8B26_000857
Unmapped UID: D8B26_006514
Unmapped UID: D8B26_008422
Unmapped UID: D8B26_000825
Unmapped UID: D8B26_003554
Unmapped UID: D8B26_005402
Unmapped UID: D8B26_006211
Unmapped UID: D8B26_006872
Unmapped UID: D8B26_005277
Unmapped UID: D8B26_008385
Unmapped UID: D8B26_001623
Unmapped UID: D8B26_006144
Unmapped UID: D8B26_007955
Unmapped UID: D8B26_006430
Unmapped UID: D8B26_006185
Unmapped UID: D8B26_007648
Unmapped UID: D8B26_001827
Unmapped UID: D8B26_006696
Unmapped UID: D8B26_005067
Unmapped UID: D8B26_008204
Unmapped UID: D8B26_004488
Unmapped UID: D8B26_008180
Unmapped UID: D8B26_000590
Unmapped UID: D8B26_001217
Unmapped UID: D8B26_004994
Unmapped UID: D8B26_007032
Unmapped UID: D8B26_004235
Unmapped UID: D8B26_002626
Unmapped UID: D8B26_004677
Unmapped UID: D8B26_001073
Unmapped UID: D8B26_004367
Unmapped UID: D8B26_006124
Unmapped UID: D8B26_000726
Unmapped UID: D8B26_004415
Unmapped UID: D8B26_002343
Unmapped UID: D8B26_005432
Unmapped UID: D8B26_008327
Unmapped UID: D8B26_002685
Unmapped UID: D8B26_006767
Unmapped UID: D8B26_005554
Unmapped UID: D8B26_008378
Unmapped UID: D8B26_007237
Unmapped UID: D8B26_005878
Unmapped UID: D8B26_000226
Unmapped UID: D8B26_008263
Unmapped UID: D8B26_001566
Unmapped UID: D8B26_008382
Unmapped UID: D8B26_005762
Unmapped UID: D8B26_006267
Unmapped UID: D8B26_006074
Unmapped UID: D8B26_007716
Unmapped UID: D8B26_008403
Unmapped UID: D8B26_005855
Unmapped UID: D8B26_007703
Unmapped UID: D8B26_007522
Unmapped UID: D8B26_003711
Unmapped UID: D8B26_000113
Unmapped UID: D8B26_003775
Unmapped UID: D8B26_004112
Unmapped UID: D8B26_004880
Unmapped UID: D8B26_002442
Unmapped UID: D8B26_001314
Unmapped UID: D8B26_006515
Unmapped UID: D8B26_003279
Unmapped UID: D8B26_000378
Unmapped UID: D8B26_006656
Unmapped UID: D8B26_002402
Unmapped UID: D8B26_001828
Unmapped UID: D8B26_001245
Unmapped UID: D8B26_005399
Unmapped UID: D8B26_000368
Unmapped UID: D8B26_000105
Unmapped UID: D8B26_000311
Unmapped UID: D8B26_006317
Unmapped UID: D8B26_000817
Unmapped UID: D8B26_004007
Unmapped UID: D8B26_002345
Unmapped UID: D8B26_003917
Unmapped UID: D8B26_007621
Unmapped UID: D8B26_005036
Unmapped UID: D8B26_000276
Unmapped UID: D8B26_004975
Unmapped UID: D8B26_004204
Unmapped UID: D8B26_003943
Unmapped UID: D8B26_006069
Unmapped UID: D8B26_003256
Unmapped UID: D8B26_001067
Unmapped UID: D8B26_002896
Unmapped UID: D8B26_004455
Unmapped UID: D8B26_002541
Unmapped UID: D8B26_007932
Unmapped UID: D8B26_000422
Unmapped UID: D8B26_001806
Unmapped UID: D8B26_005817
Unmapped UID: D8B26_004192
Unmapped UID: D8B26_003695
Unmapped UID: D8B26_000049
Unmapped UID: D8B26_007411
Unmapped UID: D8B26_001539
Unmapped UID: D8B26_000240
Unmapped UID: D8B26_003885
Unmapped UID: D8B26_007985
Unmapped UID: D8B26_002354
Unmapped UID: D8B26_000897
Unmapped UID: D8B26_002537
Unmapped UID: D8B26_006783
Unmapped UID: D8B26_008405
Unmapped UID: D8B26_003648
Unmapped UID: D8B26_002499
Unmapped UID: D8B26_005780
Unmapped UID: D8B26_007854
Unmapped UID: D8B26_007483
Unmapped UID: D8B26_000570
Unmapped UID: D8B26_005372
Unmapped UID: D8B26_006780
Unmapped UID: D8B26_004428
Unmapped UID: D8B26_004207
Unmapped UID: D8B26_002779
Unmapped UID: D8B26_003098
Unmapped UID: D8B26_005301
Unmapped UID: D8B26_004728
Unmapped UID: D8B26_001972
Unmapped UID: D8B26_001044
Unmapped UID: D8B26_007282
Unmapped UID: D8B26_002437
Unmapped UID: D8B26_000856
Unmapped UID: D8B26_001989
Unmapped UID: D8B26_001443
Unmapped UID: D8B26_004540
Unmapped UID: D8B26_008077
Unmapped UID: D8B26_006477
Unmapped UID: D8B26_004923
Unmapped UID: D8B26_004734
Unmapped UID: D8B26_004783
Unmapped UID: D8B26_007003
Unmapped UID: D8B26_001089
Unmapped UID: D8B26_001620
Unmapped UID: D8B26_005517
Unmapped UID: D8B26_000579
Unmapped UID: D8B26_003535
Unmapped UID: D8B26_008080
Unmapped UID: D8B26_004733
Unmapped UID: D8B26_005688
Unmapped UID: D8B26_003368
Unmapped UID: D8B26_000269
Unmapped UID: D8B26_002205
Unmapped UID: D8B26_005851
Unmapped UID: D8B26_008402
Unmapped UID: D8B26_006718
Unmapped UID: D8B26_004059
Unmapped UID: D8B26_003855
Unmapped UID: D8B26_004996
Unmapped UID: D8B26_000415
Unmapped UID: D8B26_005141
Unmapped UID: D8B26_003972
Unmapped UID: D8B26_000583
Unmapped UID: D8B26_006546
Unmapped UID: D8B26_007695
Unmapped UID: D8B26_003262
Unmapped UID: D8B26_002920
Unmapped UID: D8B26_006992
Unmapped UID: D8B26_001524
Unmapped UID: D8B26_005060
Unmapped UID: D8B26_005645
Unmapped UID: D8B26_008067
Unmapped UID: D8B26_005763
Unmapped UID: D8B26_005433
Unmapped UID: D8B26_001430
Unmapped UID: D8B26_008388
Unmapped UID: D8B26_004270
Unmapped UID: D8B26_001917
Unmapped UID: D8B26_002802
Unmapped UID: D8B26_003889
Unmapped UID: D8B26_003727
Unmapped UID: D8B26_004835
Unmapped UID: D8B26_007188
Unmapped UID: D8B26_003063
Unmapped UID: D8B26_002220
Unmapped UID: D8B26_005208
Unmapped UID: D8B26_007954
Unmapped UID: D8B26_000496
Unmapped UID: D8B26_000310
Unmapped UID: D8B26_006744
Unmapped UID: D8B26_001432
Unmapped UID: D8B26_005395
Unmapped UID: D8B26_005169
Unmapped UID: D8B26_008397
Unmapped UID: D8B26_000205
Unmapped UID: D8B26_001905
Unmapped UID: D8B26_008395
Unmapped UID: D8B26_000156
Unmapped UID: D8B26_007978
Unmapped UID: D8B26_002547
Unmapped UID: D8B26_003997
Unmapped UID: D8B26_005644
Unmapped UID: D8B26_003754
Unmapped UID: D8B26_007288
Unmapped UID: D8B26_001452
```

In [39]:

```
cys_sigpep_cdt = CdtFile.fromPrototype(cys_cdt, 
    probes = [CdtRow.fromPrototype(i, ratios = i.ratios + [float(Annotation(i.uniqid).signalp == "Yes"),
                                                          ])
              for i in cys_cdt],
                             fieldnames = cys_cdt.fieldnames + ["sigpep"],
                             eweights = cys_cdt.eweights + [1.])
```

### Look at spherule and hyphal expression¶

In [40]:

```
#Load Fig3 expression data
Fig3 = CdtFile.fromCdt("../Fig2/Combined/limma1_sig.countscutoff.contrasts_um.cdt")
```

In [41]:

```
D2_D3_D6_morph_indices = [[],[],[],[]]
for j,fieldname in enumerate(Fig3.fieldnames): 
    if fieldname == 'D6_spherules_Sil/D6_myc_Sil':
        D2_D3_D6_morph_indices[3] = j
    elif fieldname == 'D3_spherules_Sil/D3_myc_Sil':
        D2_D3_D6_morph_indices[2] = j
    elif fieldname == 'D2_spherule_Sil/D2_myc_Sil':
        D2_D3_D6_morph_indices[1] = j
    elif fieldname == "D1_spherule_Sil/D1_myc_Sil":
        D2_D3_D6_morph_indices[0] = j
print(D2_D3_D6_morph_indices)
```

```
[91, 102, 112, 130]
```

In [42]:

```
spherule = [i.uniqid for i in Fig3 if(i[91] >= 1 and i[102] >=1 and i[112] >=1 and i[130]>=1)]
hyphal = [i.uniqid for i in Fig3 if(i[91] <= -1 and i[102] <=-1 and i[112] <=-1 and i[130] <=-1)]
neutral = [i.uniqid for i in Fig3 if((i[91] < 1 or i[102] <1 or i[112] <1 or i[130]<1) and (i[91]>-1 or i[102] > -1 or i[112] >-1 or i[130] >-1))]
```

In [43]:

```
print(len(spherule), len(hyphal), len(neutral))
print(len(Fig3))
```

```
329 306 7409
8044
```

In [44]:

```
effectors = CdtFile.fromPrototype(cys_sigpep_cdt, probes = [i for i in cys_sigpep_cdt if (
(i.ratios[0]>=4)  and 
(i.ratios[2]>0)   and
(i.uniqid in spherule))])
```

In [45]:

```
final_effector_list = []
for i in effectors:
    print(i.uniqid)
    final_effector_list.append(i.uniqid)
```

```
D8B26_003939
D8B26_005613
D8B26_001391
D8B26_002468
D8B26_005342
D8B26_007421
D8B26_004686
D8B26_004181
D8B26_005770
D8B26_001137
D8B26_005065
D8B26_006671
D8B26_007870
D8B26_003030
D8B26_000077
D8B26_006423
```

In [46]:

```
#Generate heatmap for Fig 5c - manually edited and reordered to reflect columns in Fig 5c and then clustered again
effectors_final = CdtFile.fromPrototype(Fig3, probes = [i for i in Fig3 if i.uniqid in final_effector_list])

count_cols = [n for (n,i) in enumerate(effectors_final.fieldnames) if(("/" not in i) and ("yp" not in i) and ("edo" not in i))]
tree = effectors_final.cluster(cols=count_cols,dist="u",method="m")
effectors_final.writeCdtGtr("effectors.um", tree)
```

```
Building array...
Building distance matrix...
Clustering...
```

In [ ]:

```

```
