# Supplementary material for: Transcriptomic atlas throughout Coccidioides development reveals key phase-enriched transcripts of this important fungal pathogen
Source: PLoS Biol. 2025 Apr 15;23(4):e3003066. doi: 10.1371/journal.pbio.3003066 (PMC12077801; doi:10.1371/journal.pbio.3003066)
Supplement: S1 Code — Folder containing README document describing the scripts used to analyze the data and generate figures in this manuscript, as well as the scripts themselves and custom python three modules used in the scripts. (ZIP) [file pbio.3003066.s025.zip › Custom Code/notebooks/FigS3B.html]

FigS3B


In [1]:

```
cd ../../Papers/Cocci_transcriptomics/data_for_code/FigS3B/
```

```
/home/chomer/Papers/Cocci_transcriptomics/data_for_code/FigS3B
```

In [2]:

```
%load_ext rpy2.ipython
from glob import glob
from MsvUtil import Table
import os.path
from CdtFile import CdtFile, CdtRow
from SafeMath import safelog
from ReadCountTools import PseudoCPMs
from PCA import PCA
import numpy as np
import matplotlib.patches as patches
import matplotlib.transforms as transforms
import matplotlib.pyplot as plt
%matplotlib nbagg
from csv import writer, excel_tab
from CdtAnnotator3 import annotate_CpSilveira as annotate
import scipy.stats
```

In [3]:

```
%%R
library(limma)
library(edgeR)
```

# Merge Kallisto TPMs¶

In [4]:

```
# read in Fig 1 data
datadir = "../Fig1/"

sname2kallisto = dict(
    (i.replace(".Silv_nanopore_mRNA.rf.kallisto","").replace(datadir,"").split("_")[0],i)
              for i in glob(datadir+"*.Silv_nanopore_mRNA.rf.kallisto") if("Spores" in i))

snames = sorted(sname2kallisto, key = lambda x: (x[0:x.rfind("_")],x[x.rfind("_"):]), reverse=False)

print(len(sname2kallisto))
print(snames)
```

```
3
['Spores1', 'Spores2', 'Spores3']
```

In [5]:

```
#Merge Step
genes = None
cols = []
counts = []
for i in snames:
    table = Table.fromTdt(open(os.path.join(
           sname2kallisto[i],
          "abundance.tsv")))
    if(genes is None):
        genes = table["target_id"]
    else:
        assert(genes == table["target_id"])
    cols.append([float(i) for i in table["tpm"]])
    counts.append([int(float(i)+.5) for i in table["est_counts"]])

Fig1_tpm_trans = CdtFile(probes = [CdtRow(gid = i[0], uniqid = i[0], name = i[0],
                                        ratios = [safelog(j) for j in i[1:]])
                                 for i in zip(*([genes]+cols))],
                       fieldnames = snames,
                       eweights = [1]*len(snames))
Fig1_tpm_trans.write(open("Fig1_tpm_trans.cdt","w"))

Fig1_trans_counts = CdtFile(probes = [CdtRow(gid = i[0], uniqid = i[0], name = i[0],
                                        ratios = i[1:])
                                 for i in zip(*([genes]+counts))],
                       fieldnames = snames,
                       eweights = [1]*len(snames))
Fig1_trans_counts.write(open("Fig1_trans_counts.cdt","w"))
Fig1_trans_counts = CdtFile.fromPrototype(Fig1_trans_counts, fieldnames = ["Fig1|"+i for i in Fig1_trans_counts.fieldnames])

len(Fig1_tpm_trans), len(Fig1_trans_counts)
```

Out[5]:

```
(8628, 8628)
```

In [6]:

```
#read in Fig 2 data
datadir = "../Fig2/"

sname2kallisto = dict(
    ("_".join(i.replace(".Silv_nanopore_mRNA.rf.kallisto","").replace(datadir,"").split("_")[0:-3]),i)
              for i in glob(datadir+"*.Silv_nanopore_mRNA.rf.kallisto") if("Spores" in i and "Week" not in i and "37" not in i and "RT" not in i and "Redo" not in i))

snames = sorted(sname2kallisto, key = lambda x: (x[0:x.rfind("_")],x[x.rfind("_"):]), reverse=False)

print(len(sname2kallisto))
print(snames)
```

```
6
['Spores_Ryp1_1', 'Spores_Ryp1_2', 'Spores_Ryp1_3', 'Spores_Sil_1', 'Spores_Sil_2', 'Spores_Sil_3']
```

In [7]:

```
#Merge Step
genes = None
cols = []
counts = []
for i in snames:
    table = Table.fromTdt(open(os.path.join(
           sname2kallisto[i],
          "abundance.tsv")))
    if(genes is None):
        genes = table["target_id"]
    else:
        assert(genes == table["target_id"])
    cols.append([float(i) for i in table["tpm"]])
    counts.append([int(float(i)+.5) for i in table["est_counts"]])

Fig2_tpm_trans = CdtFile(probes = [CdtRow(gid = i[0], uniqid = i[0], name = i[0],
                                        ratios = [safelog(j) for j in i[1:]])
                                 for i in zip(*([genes]+cols))],
                       fieldnames = snames,
                       eweights = [1]*len(snames))
Fig2_tpm_trans.write(open("Fig2_tpm_trans.cdt","w"))

Fig2_trans_counts = CdtFile(probes = [CdtRow(gid = i[0], uniqid = i[0], name = i[0],
                                        ratios = i[1:])
                                 for i in zip(*([genes]+counts))],
                       fieldnames = snames,
                       eweights = [1]*len(snames))
Fig2_trans_counts.write(open("Fig2_trans_counts.cdt","w"))
Fig2_trans_counts = CdtFile.fromPrototype(Fig2_trans_counts, fieldnames = ["Fig2|"+i for i in Fig2_trans_counts.fieldnames])

len(Fig2_tpm_trans), len(Fig2_trans_counts)
```

Out[7]:

```
(8628, 8628)
```

In [8]:

```
#Merge Kallisto TPMs
#First Index
datadir = "../FigS4/"

sname2kallisto = dict(
    ("_".join(i.replace(".Silv_nanopore_mRNA.rf.kallisto","").replace(datadir,"").split("_")[:-1]),i)
        for i in glob(datadir+"*.Silv_nanopore_mRNA.rf.kallisto") if("rth" in i))
snames = sorted(sname2kallisto, key = lambda x: (x[4:x.rfind("_")],x[:4]))

print(len(sname2kallisto))
print(snames)
```

```
6
['Ryp1_Arth_3', 'Ryp1_Arth_1', 'Ryp1_Arth_2', 'WT_Arth_2', 'WT_Arth_3', 'WT_Arth_1']
```

In [9]:

```
#Merge Step
genes = None
cols = []
counts = []
for i in snames:
    table = Table.fromTdt(open(os.path.join(
           sname2kallisto[i],
          "abundance.tsv")))
    if(genes is None):
        genes = table["target_id"]
    else:
        assert(genes == table["target_id"])
    cols.append([float(i) for i in table["tpm"]])
    counts.append([int(float(i)+.5) for i in table["est_counts"]])

FigS4_tpm_trans = CdtFile(probes = [CdtRow(gid = i[0], uniqid = i[0], name = i[0],
                                        ratios = [safelog(j) for j in i[1:]])
                                 for i in zip(*([genes]+cols))],
                       fieldnames = snames,
                       eweights = [1]*len(snames))
FigS4_tpm_trans.write(open("FigS4_tpm_trans.cdt","w"))

FigS4_trans_counts = CdtFile(probes = [CdtRow(gid = i[0], uniqid = i[0], name = i[0],
                                        ratios = i[1:])
                                 for i in zip(*([genes]+counts))],
                       fieldnames = snames,
                       eweights = [1]*len(snames))
FigS4_trans_counts.write(open("FigS4_trans_counts.cdt","w"))

len(FigS4_tpm_trans), len(FigS4_trans_counts)
FigS4_trans_counts = CdtFile.fromPrototype(FigS4_trans_counts, fieldnames = ["FigS4|"+i for i in FigS4_trans_counts.fieldnames])
len(FigS4_trans_counts)
```

Out[9]:

```
8628
```

In [10]:

```
genes = sorted(i.Uniqid() for i in Fig1_trans_counts)
len(genes)
```

Out[10]:

```
8628
```

In [11]:

```
experiment_counts = Fig1_trans_counts,Fig2_trans_counts,FigS4_trans_counts
```

In [12]:

```
fieldnames = []
eweights = []
for i in experiment_counts:
    fieldnames += i.fieldnames
    eweights += i.eweights

probes = []
for i in genes:
    rows = [j.GetUid(i) for j in experiment_counts]
    ratios = []
    for j in rows:
        ratios += j.ratios
    
    probes.append(CdtRow.fromPrototype(rows[0], ratios = ratios))

merge_counts = annotate(CdtFile(fieldnames = fieldnames, eweights = eweights, extranames=experiment_counts[0].extranames,
                       probes = probes), warn = False)
len(merge_counts)
```

```
Warning, didn't find a GID column!  Sharing UID with GID.
```

Out[12]:

```
8628
```

In [13]:

```
merge_pseudo = PseudoCPMs.fromCounts(merge_counts)
#pseudoCPMs are log transformed
#counts are to feed directly to limma
```

In [14]:

```
mask_10 = merge_pseudo.depth_filter_mask(10,.01)
mask_10.counts.write(open("Merge_Fig1_Fig2_FigS4_counts_pseudoCPMs.cdt", "w"))
```

In [15]:

```
ct_counts = CdtFile.fromCdt("Merge_Fig1_Fig2_FigS4_counts_pseudoCPMs.cdt")
len(ct_counts)
```

Out[15]:

```
8274
```

# Limma analysis¶

In [16]:

```
#need to format ct_counts for limma input

fout = open("ct_counts.txt", "w")

fout.write("\t".join(["gene"]+ct_counts.fieldnames)+"\n")
for row in mask_10.counts:
    fout.write("\t".join([row.Uniqid()]+[str(i) for i in row])+"\n")
fout.close()
```

In [17]:

```
%%R
#Limma Single Factor Fit
# Read the count matrix, using the gene column as row names
C <- read.delim("ct_counts.txt",row.names=1)
#Convert the matrix to limma's preferred format, implicitly log2 transforming and depth normalizing to CPM values
dge <- DGEList(counts=C)
```

In [18]:

```
out = writer(open("Arth_comparison_simple_comp_samples.txt","w"),dialect = excel_tab)
out.writerow(("run","state"))
for i in ct_counts.fieldnames:
    run = i
    if i == "Redo_Ryp1_Spores_1":
        state = "Spores_Ryp1"
    elif i == "Redo_D2_spherule_Sil_3":
        state = "CHAS02_D2_spherule_Sil"
    else:
        state1 = i.split("|")[0]
        if state1 == "CHAS01":
            if "Spores" in i:
                state2 = [i.split("|")[-1][:-1]]
            else:
                state2 = i.split("|")[-1].split("-")[:-1]
        else:
            state2 = i.split("|")[-1].split("_")[0:-1]
        state_list = [state1]+state2
        state = "_".join(state_list)
        print(state)
    out.writerow((run, state))   
del out
```

```
Fig1
Fig1
Fig1
Fig2_Spores_Ryp1
Fig2_Spores_Ryp1
Fig2_Spores_Ryp1
Fig2_Spores_Sil
Fig2_Spores_Sil
Fig2_Spores_Sil
FigS4_Ryp1_Arth
FigS4_Ryp1_Arth
FigS4_Ryp1_Arth
FigS4_WT_Arth
FigS4_WT_Arth
FigS4_WT_Arth
```

In [19]:

```
%%R -o d
samples <- read.delim("Arth_comparison_simple_comp_samples.txt", header=TRUE, sep="\t")
print(summary(samples))
state <- samples$state
d <- model.matrix(~0+state)
colnames(d) <- gsub("state","",colnames(d))
print(colnames(d))
```

```
     run               state          
 Length:15          Length:15         
 Class :character   Class :character  
 Mode  :character   Mode  :character  
[1] "Fig1"             "Fig2_Spores_Ryp1" "Fig2_Spores_Sil"  "FigS4_Ryp1_Arth" 
[5] "FigS4_WT_Arth"
```

In [20]:

```
%%R
# Apply between-sample TMM normalization
dge <- calcNormFactors(dge)
# Estimate the mean-variance trend via locally-linear regression and use this trend
# to assign weights to the observations (counts)
v <- voom(dge, d, plot = TRUE)
cpm <- v$E
```

In [21]:

```
%%R -o cpm,fc,cn,state
# Fit the model (classic linear regression)
fit <- lmFit(v, d)
#Generate the contrast matrix
contrast.matrix <- makeContrasts(
    Fig2_Spores_Sil-Fig1,FigS4_WT_Arth- Fig1, FigS4_WT_Arth - Fig2_Spores_Sil,
    Fig2_Spores_Sil - Fig2_Spores_Ryp1,
    FigS4_WT_Arth - FigS4_Ryp1_Arth, 
    levels=d)
# Apply the contrast matrix
fit2 <- contrasts.fit(fit, contrast.matrix)
# Apply Empirical Bayes "shrinkage"
fit2 <- eBayes(fit2)
# Simple summary of significantly differential genes with no fold change filter
print(summary(decideTests(fit2)))

fc <- fit$coefficients
cn <- colnames(fit$coefficients)
cpm <- v$E
```

```
       Fig2_Spores_Sil - Fig1 FigS4_WT_Arth - Fig1
Down                     2428                 2138
NotSig                   3677                 3916
Up                       2169                 2220
       FigS4_WT_Arth - Fig2_Spores_Sil Fig2_Spores_Sil - Fig2_Spores_Ryp1
Down                              2684                               2793
NotSig                            2509                               2749
Up                                3081                               2732
       FigS4_WT_Arth - FigS4_Ryp1_Arth
Down                              2778
NotSig                            2584
Up                                2912
```

In [22]:

```
%%R
print(summary(decideTests(fit2,lfc=1)))
```

```
       Fig2_Spores_Sil - Fig1 FigS4_WT_Arth - Fig1
Down                     1322                  614
NotSig                   5956                 6644
Up                        996                 1016
       FigS4_WT_Arth - Fig2_Spores_Sil Fig2_Spores_Sil - Fig2_Spores_Ryp1
Down                              1558                               2026
NotSig                            4504                               4547
Up                                2212                               1701
       FigS4_WT_Arth - FigS4_Ryp1_Arth
Down                              1584
NotSig                            4949
Up                                1741
```

In [23]:

```
name2row = dict((i.Uniqid(),n+1) for (n,i) in enumerate(ct_counts))
```

In [24]:

```
ct_counts.mean_normalize_rows().bicluster("ct_counts.norm.um",dist="u",method="m")
```

```
Building array...
Building distance matrix...
Clustering...
```

In [25]:

```
%%R
write.csv(cpm,"limma1.countscutoff.cpm.csv")

for(tc in colnames(fit2$coefficients)){
  print(tc)
  # Extract all genes significantly differential on this contrast for a 2x fold change cutoff and 5% FDR
  # Use write.csv rather than write.table for clean compatibility with python's csv.reader
  write.csv(topTable(fit2, coef=tc, n = 50000, lfc=1, p.value = .05),
            paste("limma1.",gsub(" ","",tc),".t0.csv",sep=""))
  # Extract the adjusted p-values for this contrast for all genes, independent of significance
  write.csv(topTable(fit2, coef=tc, n = 50000),
            paste("limma1.",gsub(" ","",tc),".t1.csv",sep=""))
}
```

```
[1] "Fig2_Spores_Sil - Fig1"
[1] "FigS4_WT_Arth - Fig1"
[1] "FigS4_WT_Arth - Fig2_Spores_Sil"
[1] "Fig2_Spores_Sil - Fig2_Spores_Ryp1"
[1] "FigS4_WT_Arth - FigS4_Ryp1_Arth"
```

In [26]:

```
gene2cpms = dict((i[0],[float(j) for j in i[1:]]) for i in Table.fromCsv("limma1.countscutoff.cpm.csv"))
len(gene2cpms), len(ct_counts)
```

Out[26]:

```
(8274, 8274)
```

In [27]:

```
limma1_cdt = CdtFile.fromPrototype(ct_counts, 
                                   probes = [CdtRow.fromPrototype(i, ratios = gene2cpms[i.Uniqid()][:])
                                             for i in ct_counts])
limma1_cdt = limma1_cdt.mean_normalize_rows()
```

In [28]:

```
gene2contrasts = dict((i.Uniqid(),[]) for i in limma1_cdt)
gene2pvals = dict((i.Uniqid(),[]) for i in limma1_cdt)
gene2sigs = dict((i.Uniqid(),[]) for i in limma1_cdt)
contrast_names = []

contrast_csvs = sorted(glob("limma1.*.t1.csv"))
# put 8 hour first
contrast_csvs = contrast_csvs[-1:]+contrast_csvs[:-1]
for i in contrast_csvs:
    cname = i.replace("limma1.","").replace(".t1.csv","").replace("-","/")
    contrast_names.append(cname)
    siglist = set(i[0] for i in Table.fromCsv(i.replace(".t1.",".t0.")))
    print(cname,len(siglist))
    for gene in Table.fromCsv(i):
        name = gene[0]
        lfc = float(gene["logFC"])
        gene2contrasts[name].append(lfc)
        gene2pvals[name].append(gene["adj.P.Val"])
        if(name in siglist):
            if(lfc > 0):
                gene2sigs[name].append(4.)
            else:
                gene2sigs[name].append(-4.)
        else:
            gene2sigs[name].append(0.)
        
limma1_cdt = CdtFile.fromPrototype(limma1_cdt,
    probes = [CdtRow.fromPrototype(i, ratios = i.ratios+gene2contrasts[i.Uniqid()]+gene2sigs[i.Uniqid()],
                                   extra = i.extra+gene2pvals[i.Uniqid()])
              for i in limma1_cdt],
    fieldnames = limma1_cdt.fieldnames+contrast_names+["%s_sig" % i for i in contrast_names],
    eweights = limma1_cdt.eweights+[1.]*2*len(contrast_names),
    extranames = limma1_cdt.extranames+["p(%s)" % i for i in contrast_names])
```

```
FigS4_WT_Arth/FigS4_Ryp1_Arth 3325
Fig2_Spores_Sil/Fig1 2318
Fig2_Spores_Sil/Fig2_Spores_Ryp1 3727
FigS4_WT_Arth/Fig1 1630
FigS4_WT_Arth/Fig2_Spores_Sil 3770
```

In [29]:

```
limma1_cdt = CdtFile.fromPrototype(limma1_cdt, 
    probes = [CdtRow.fromPrototype(i, extra = i.extra + [str(j) for j in ct_counts.GetUid(i.Uniqid())])
              for i in limma1_cdt],
    extranames = limma1_cdt.extranames+["%s_counts" % i for i in ct_counts.fieldnames])
```

In [30]:

```
sig_cols = [n for (n,i) in enumerate(limma1_cdt.fieldnames) if(i.endswith("_sig"))]
contrast_cols = [n-len(sig_cols) for n in sig_cols]
limma1_2x = CdtFile.fromPrototype(limma1_cdt, probes = [i for i in limma1_cdt 
                                                        if(any([(i[j] != 0.) for j in sig_cols]))])
tree = limma1_2x.cluster(cols=contrast_cols,dist="u",method="m")
limma1_2x.writeCdtGtr("limma1_2x.countscutoff.merge.contrasts_um",tree)
len(limma1_2x)
```

```
Building array...
Building distance matrix...
Clustering...
```

Out[30]:

```
6411
```

# Looking at the WT arthroconidia signature in FigS4 where the arthroconidia did not sit at 4C compared to Fig2¶

In [31]:

```
for n,i in enumerate(limma1_cdt.fieldnames):
    if i == "Fig2_Spores_Sil/Fig2_Spores_Ryp1":
        Fig2_comp = n
    elif i == "FigS4_WT_Arth/FigS4_Ryp1_Arth":
        FigS4_comp = n

xvalues = []
yvalues = []
for row in limma1_cdt:
    xvalues.append(row.ratios[Fig2_comp])
    yvalues.append(row.ratios[FigS4_comp])
```

In [32]:

```
#Figure S3B
fig,ax = plt.subplots()
ax.scatter(xvalues, yvalues, s=5, facecolors='none', edgecolors='black')
print(round(scipy.stats.pearsonr(xvalues, yvalues)[0],5))
ax.set_box_aspect(1)
plt.show()
plt.savefig("Arth_ryp1_comparisons.eps")
```

```
0.49428
```

In [ ]:

```

```
