# Supplementary material for: Transcriptomic atlas throughout Coccidioides development reveals key phase-enriched transcripts of this important fungal pathogen
Source: PLoS Biol. 2025 Apr 15;23(4):e3003066. doi: 10.1371/journal.pbio.3003066 (PMC12077801; doi:10.1371/journal.pbio.3003066)
Supplement: S1 Code — Folder containing README document describing the scripts used to analyze the data and generate figures in this manuscript, as well as the scripts themselves and custom python three modules used in the scripts. (ZIP) [file pbio.3003066.s025.zip › Custom Code/notebooks/Fig4_and_S4_motif_analysis.html]

Fig4\_and\_S4\_motif\_analysis


In [1]:

```
import matplotlib.pyplot as plt
%matplotlib nbagg
```

In [2]:

```
%cd ../../Papers/Cocci_transcriptomics/data_for_code/Fig4/
```

```
/home/chomer/Papers/Cocci_transcriptomics/data_for_code/Fig4
```

In [3]:

```
from UnionedGenomeFactory import MySQLGenomeFactory
from glob import glob
from MacsTools import parse_MACS_xls, MacsPeak
from Gff3 import Gff3file, Gff3record
from DnaSearch import MotifHit, intergenics
from ConsensusSearch import intergenic_promoters
from Collisions import RefCollisions
from CdtFile import CdtFile, CdtRow
import numpy as np
import scipy.stats as stats
```

In [4]:

```
%load_ext rpy2.ipython
```

In [5]:

```
f = MySQLGenomeFactory(db = "Genome4", assembly_caching="eager")
genome = f.getGenome("CpSilveiraV3")
```

# Read in ChIP-Seq peaks¶

In [6]:

```
sample2peaks = dict((i.replace("_peaks.xls",""),parse_MACS_xls(i,genome)) 
                    for i in glob("*.xls") if "NA" not in i)
```

In [7]:

```
for sample in sample2peaks:
    fout = open("{0}_peak_intergenics.fasta".format(sample), "w")
    peaks = sample2peaks[sample]
    for k, peak in enumerate(peaks):
        peak_seq = peak.Locus().Sequence()
        fout.write(">seq{1}\n{0}\n".format(peak_seq, peak.Locus()))
    fout.close()
```

# Deconvolute peaks assigned to shared hyphae/spherule samples and those that are spherule specific to see if the motif differs¶

In [8]:

```
#First need to combine replicates, schematic in Figure S4F
def combine_peak_reps(prefix, sample2peaks_sorted):
    new_peak_list = []
    sample1 = sample2peaks_sorted["{0}_1".format(prefix)]
    sample2 = sample2peaks_sorted["{0}_2".format(prefix)]
    sample3 = sample2peaks_sorted["{0}_3".format(prefix)]
    for peak1 in sample1:
        s1 = peak1.Locus().start
        e1 = peak1.Locus().stop
        chrom1 = peak1.Locus().ref
        max1 = peak1.abs_summit
        for peak2 in sample2:
            s2 = peak2.Locus().start
            e2 = peak2.Locus().stop
            chrom2 = peak2.Locus().ref
            max2 = peak2.abs_summit
            if chrom1 == chrom2 and max1 > s2 and max1 < e2 and max2 > s1 and max2 < e1:
                if s1 < s2:
                    new_start = s2
                else:
                    new_start = s1
                if e1 < e2:
                    new_end = e1
                else:
                    new_end = e2
                new_peak = MacsPeak([chrom1,new_start,new_end,new_end-new_start, ((max1+max2)/2.), peak1.pileup+peak2.pileup, (peak1.pileup+peak2.pileup)/2, (peak1.lfc+peak2.lfc)/2, (peak1.q+peak2.q)/2, peak1.name+peak2.name] , genome=genome       )
                for peak3 in sample3:
                    s3 = peak3.Locus().start
                    e3 = peak3.Locus().stop
                    chrom3 = peak3.Locus().ref
                    max3 = peak3.abs_summit
                    s4 = new_peak.Locus().start
                    e4 = new_peak.Locus().stop
                    chrom4 = new_peak.Locus().ref
                    max4 = new_peak.abs_summit
                    if chrom4 == chrom3 and max3 > s4 and max3 < e4 and max4 > s3 and max4 < e3:
                        if s3 < s4:
                            new_start = s4
                        else:
                            new_start = s3
                        if e3 < e4:
                            new_end = e3
                        else:
                            new_end = e4
                        new_peak2 = MacsPeak([chrom3,new_start,new_end,new_end-new_start, ((max3+max4)/2.), peak3.pileup+new_peak.pileup, (peak3.pileup+new_peak.pileup)/2, (peak3.lfc+new_peak.lfc)/2, (peak3.q+new_peak.q)/2, peak3.name+new_peak.name], genome=genome    )
                        new_peak_list.append(new_peak2) 
    
    return new_peak_list
```

In [9]:

```
#Then determine overlap between 2 different datasets
def overlap_peaks(prefix1, prefix2, combined_peaks):
    unique_sample1 = []
    unique_sample2 = []
    overlap = []
    sample1 = combined_peaks[prefix1]
    sample2 = combined_peaks[prefix2]
    peak1_matched = []
    peak2_matched = []
    for peak1 in sample1:
        match_found = False
        s1 = peak1.Locus().start
        e1 = peak1.Locus().stop
        chrom1 = peak1.Locus().ref
        max1 = peak1.abs_summit
        for peak2 in sample2:
            s2 = peak2.Locus().start
            e2 = peak2.Locus().stop
            chrom2 = peak2.Locus().ref
            max2 = peak2.abs_summit
            if chrom1 == chrom2 and max1 > s2 and max1 < e2 and max2 > s1 and max2 < e1:
                if s1 < s2:
                    new_start = s2
                else:
                    new_start = s1
                if e1 < e2:
                    new_end = e1
                else:
                    new_end = e2
                match_found = True
                new_peak = MacsPeak([chrom1,new_start,new_end,new_end-new_start, ((max1+max2)/2.), peak1.pileup+peak2.pileup, (peak1.pileup+peak2.pileup)/2, (peak1.lfc+peak2.lfc)/2, (peak1.q+peak2.q)/2, peak1.name+peak2.name], genome=genome      )
                overlap.append(new_peak)
        if match_found == False:
            unique_sample1.append(peak1)
    for peak2 in sample2:
        match_found = False
        s2 = peak2.Locus().start
        e2 = peak2.Locus().stop
        chrom2 = peak2.Locus().ref
        max2 = peak2.abs_summit
        for peak1 in sample1:
            s1 = peak1.Locus().start
            e1 = peak1.Locus().stop
            chrom1 = peak1.Locus().ref
            max1 = peak1.abs_summit
            if chrom1 == chrom2 and max1 > s2 and max1 < e2 and max2 > s1 and max2 < e1:
                match_found = True
        if match_found == False:
            unique_sample2.append(peak2)

                
    return unique_sample1, unique_sample2, overlap
```

In [10]:

```
prefix_list = ["WT_Arth", "WT_D4_Hyph", "Ryp1_Arth", "Ryp1_D1_Hyph", "WT_D2_Hyph", "Ryp1_D1_Spher", "WT_D1_Hyph", "WT_8h_Hyph", "WT_8h_Spher", "WT_D1_Spher", "WT_D2_Spher", "WT_D4_Spher"]
```

In [11]:

```
combined_peaks = {}
for prefix in prefix_list:
    new_peak_list = combine_peak_reps(prefix, sample2peaks)
    print(prefix, len(new_peak_list))
    combined_peaks[prefix] = new_peak_list
```

```
WT_Arth 6
WT_D4_Hyph 568
Ryp1_Arth 20
Ryp1_D1_Hyph 53
WT_D2_Hyph 265
Ryp1_D1_Spher 23
WT_D1_Hyph 1
WT_8h_Hyph 10
WT_8h_Spher 1
WT_D1_Spher 157
WT_D2_Spher 3450
WT_D4_Spher 1442
```

In [12]:

```
unique_WT_D2S, unique_WT_D2H, D2S_D2H_overlap = overlap_peaks("WT_D2_Spher", "WT_D2_Hyph", combined_peaks)
fout = open("unique_WT_D2S_peak_intergenics.fasta", "w")
for peak in unique_WT_D2S:
    peak_seq = peak.Locus().Sequence()
    fout.write(">seq{1}\n{0}\n".format(peak_seq, peak.Locus()))
fout.close()  

fout = open("unique_WT_D2H_peak_intergenics.fasta", "w")
for peak in unique_WT_D2H:
    peak_seq = peak.Locus().Sequence()
    fout.write(">seq{1}\n{0}\n".format(peak_seq, peak.Locus()))
fout.close()  

fout = open("overlap_WT_D2S_D2H_intergenics.fasta", "w")
for peak in D2S_D2H_overlap:
    peak_seq = peak.Locus().Sequence()
    fout.write(">seq{1}\n{0}\n".format(peak_seq, peak.Locus()))
fout.close()
```

In [13]:

```
print(len(unique_WT_D2S),len(unique_WT_D2H), len(D2S_D2H_overlap))
```

```
3231 46 219
```

In [14]:

```
unique_WT_D4S, unique_WT_D4H, D4S_D4H_overlap = overlap_peaks("WT_D4_Spher", "WT_D4_Hyph", combined_peaks)
fout = open("unique_WT_D4S_peak_intergenics.fasta", "w")
for peak in unique_WT_D4S:
    peak_seq = peak.Locus().Sequence()
    fout.write(">seq{1}\n{0}\n".format(peak_seq, peak.Locus()))
fout.close()  

fout = open("unique_WT_D4H_peak_intergenics.fasta", "w")
for peak in unique_WT_D4H:
    peak_seq = peak.Locus().Sequence()
    fout.write(">seq{1}\n{0}\n".format(peak_seq, peak.Locus()))
fout.close()  

fout = open("overlap_WT_D4S_D4H_intergenics.fasta", "w")
for peak in D4S_D4H_overlap:
    peak_seq = peak.Locus().Sequence()
    fout.write(">seq{1}\n{0}\n".format(peak_seq, peak.Locus()))
fout.close()
```

In [15]:

```
print(len(unique_WT_D4S),len(unique_WT_D4H), len(D4S_D4H_overlap))
```

```
977 103 465
```

# Used the fasta files generated above to run meme, then continued with analysis below - First with motif found in both Spherule and Hyphal peaks¶

Invocations for scripts run on the command line:

IntergenicBackgrounds.py CpSilveiraV3

MetaMotif.py -E 0.000208 --bfile\_template CpSilveiraV3.ibg --multihit S\_H\_overlap\_motif.meme 1 S\_H\_overlap\_motif CpSilveiraV3

In [16]:

```
hits = {}
for genome in ([genome]):
    name = genome.Name()
    hits[name] = [MotifHit(i, genome) for i in Gff3file.fromFile(
            open("S_H_overlap_motif.gff3","rt"))]
len(hits["CpSilveiraV3"])
```

Out[16]:

```
4231
```

In [17]:

```
intergenics = intergenic_promoters(genome, from_ATG=True)
len(intergenics), len(set((i.Locus().ref,i.Locus().start,i.Locus().stop) for i in intergenics))
```

Out[17]:

```
(8269, 6011)
```

In [18]:

```
hitgenes = {}
hitgenes["CpSilveiraV3"] = RefCollisions([item.hit for item in hits["CpSilveiraV3"]],intergenics)
```

In [19]:

```
genes_with_hit = {}
for (name, d) in hitgenes.items():
    genes_with_hit[name] = {}
    for i in hitgenes[name]:
        if i[1].Gene().Name() not in genes_with_hit[name].keys():
            genes_with_hit[name][i[1].Gene().Name()] = [i[0]]
        else:
            j = genes_with_hit[name][i[1].Gene().Name()]
            j.append(i[0])
            genes_with_hit[name][i[1].Gene().Name()] = j
```

### Load Expression Data¶

In [20]:

```
all_genes_cdt = CdtFile.fromCdt("../Fig2/Combined/limma1.countscutoff.cdt")
print(len(all_genes_cdt))

Cp_hit_genes = set(i.Uniqid() for i in all_genes_cdt).intersection(set(genes_with_hit["CpSilveiraV3"]))
print(len(Cp_hit_genes))
```

```
8186
2394
```

In [21]:

```
strict_ryp1_cdt = CdtFile.fromCdt("../Fig2/Combined/limma1_ryp1dep_all.contrasts_um.cdt")
print(len(strict_ryp1_cdt))

Cp_strict_ryp1_hit_genes = set(i.Uniqid() for i in strict_ryp1_cdt).intersection(set(genes_with_hit["CpSilveiraV3"]))
print(len(Cp_strict_ryp1_hit_genes))
```

```
79
34
```

In [22]:

```
ryp1_spher = CdtFile.fromCdt("../Fig2/Combined/limma1_ryp1dep_spherule.contrasts_um.cdt")
print(len(ryp1_spher))

Cp_spher_ryp1_hit_genes = set(i.Uniqid() for i in ryp1_spher).intersection(set(genes_with_hit["CpSilveiraV3"]))
print(len(Cp_spher_ryp1_hit_genes))
```

```
452
187
```

In [23]:

```
ryp1_myc = CdtFile.fromCdt("../Fig2/Combined/limma1_ryp1dep_mycelia.contrasts_um.cdt")
print(len(ryp1_myc))

Cp_myc_ryp1_hit_genes = set(i.Uniqid() for i in ryp1_myc).intersection(set(genes_with_hit["CpSilveiraV3"]))
print(len(Cp_myc_ryp1_hit_genes))
```

```
262
104
```

In [24]:

```
arth = CdtFile.fromCdt("../Fig2/Combined/limma1_ryp1dep_arth.contrasts_um.cdt")
print(len(arth))

Cp_arth_ryp1_hit_genes = set(i.Uniqid() for i in arth).intersection(set(genes_with_hit["CpSilveiraV3"]))
print(len(Cp_arth_ryp1_hit_genes))
```

```
3599
1097
```

In [25]:

```
morph = CdtFile.fromCdt("../Fig2/Combined/limma1_morphdep_all_wt.contrasts_um.cdt")
print(len(morph))

Cp_morph_hit_genes = set(i.Uniqid() for i in morph).intersection(set(genes_with_hit["CpSilveiraV3"]))
print(len(Cp_morph_hit_genes))
```

```
551
207
```

### Plot histogram of distance from motif to gene start¶

In [26]:

```
 %cd S_and_H_Motif/
```

```
/home/chomer/Papers/Cocci_transcriptomics/data_for_code/Fig4/S_and_H_Motif
```

In [27]:

```
#Figure S4H
#plot histogram of distance from motif to gene start using ATG to be consistent with promoter length analysis
motif_distance_to_ATG = []
for gene in Cp_hit_genes:  
    hit_list = (genes_with_hit["CpSilveiraV3"][gene])
    gene = genome.getGene(gene)
    for item in hit_list:
        hit_strand = item.strand
        hit_start = item.start
        hit_stop = item.stop
        gene_strand = gene.Locus().strand
        coords = []
        for cds in gene.CdsLoci():
            coords.append(cds.start)
            coords.append(cds.stop)            
        if gene_strand == "+":
            atg = min(coords)
            distance_to_ATG = atg - hit_start
        elif gene_strand == "-":
            atg = max(coords)
            distance_to_ATG = hit_stop - atg

        if distance_to_ATG < 15001:
                motif_distance_to_ATG.append(distance_to_ATG)


fig = plt.figure()
ax = plt.subplot(111)
plt.hist(motif_distance_to_ATG, color='grey')
plt.show()
plt.savefig("motif_distance_to_ATG.svg")
```

### Compare number of motif hits in different gene subsets¶

In [28]:

```
#all genes with Ryp1 motif hits
all_genes_numhits = []
for gene in Cp_hit_genes:  
    hit_list = (genes_with_hit["CpSilveiraV3"][gene])
    gene = genome.getGene(gene)
    all_genes_numhits.append(len(hit_list))
    
all_genes_numhits_binned_short = [0,0,0,0,0,0]

for bin in range(0,4):
    for hit in all_genes_numhits:
        if hit == bin + 1:
            all_genes_numhits_binned_short[bin] = all_genes_numhits_binned_short[bin] + 1 
for hit in all_genes_numhits:
    if hit >11:
        all_genes_numhits_binned_short[5] = all_genes_numhits_binned_short[5] + 1
    elif hit >= 5: 
        all_genes_numhits_binned_short[4] = all_genes_numhits_binned_short[4] + 1

print(all_genes_numhits_binned_short)
nums_obs = len(all_genes_numhits)
all_genes_numhits_binned_short_norm = []
for entry in all_genes_numhits_binned_short:
    all_genes_numhits_binned_short_norm.append(entry/float(nums_obs))
```

```
[1697, 465, 131, 45, 55, 1]
```

In [29]:

```
#Ryp1-spher-regulated genes with Ryp1 motif hits
ryp1_spher_numhits = []
for gene in Cp_spher_ryp1_hit_genes:  
    hit_list = (genes_with_hit["CpSilveiraV3"][gene])
    ryp1_spher_numhits.append(len(hit_list))
    gene = genome.getGene(gene)
    
ryp1_spher_numhits_binned_short = [0,0,0,0,0,0]
for bin in range(0,4):
    for hit in ryp1_spher_numhits:
        if hit == bin + 1:
            ryp1_spher_numhits_binned_short[bin] = ryp1_spher_numhits_binned_short[bin] + 1 
for hit in ryp1_spher_numhits:
    if hit > 11:
        ryp1_spher_numhits_binned_short[5] = ryp1_spher_numhits_binned_short[5] + 1
    elif hit >=5:
        ryp1_spher_numhits_binned_short[4] = ryp1_spher_numhits_binned_short[4] + 1
print(ryp1_spher_numhits_binned_short)

nums_obs = len(ryp1_spher_numhits)
ryp1_spher_numhits_binned_short_norm = []
for entry in ryp1_spher_numhits_binned_short:
    ryp1_spher_numhits_binned_short_norm.append(entry/float(nums_obs))
```

```
[114, 45, 20, 6, 2, 0]
```

In [30]:

```
#Ryp1-myc-regulated genes with Ryp1 motif hits
ryp1_myc_numhits = []
for gene in Cp_myc_ryp1_hit_genes:  
    hit_list = (genes_with_hit["CpSilveiraV3"][gene])
    ryp1_myc_numhits.append(len(hit_list))
    gene = genome.getGene(gene)
    
ryp1_myc_numhits_binned_short = [0,0,0,0,0,0]
for bin in range(0,4):
    for hit in ryp1_myc_numhits:
        if hit == bin + 1:
            ryp1_myc_numhits_binned_short[bin] = ryp1_myc_numhits_binned_short[bin] + 1 
for hit in ryp1_myc_numhits:
    if hit > 11:
        ryp1_myc_numhits_binned_short[5] = ryp1_myc_numhits_binned_short[5] + 1
    elif hit >=5:
        ryp1_myc_numhits_binned_short[4] = ryp1_myc_numhits_binned_short[4] + 1
print(ryp1_myc_numhits_binned_short)

nums_obs = len(ryp1_myc_numhits)
ryp1_myc_numhits_binned_short_norm = []
for entry in ryp1_myc_numhits_binned_short:
    ryp1_myc_numhits_binned_short_norm.append(entry/float(nums_obs))
```

```
[79, 22, 3, 0, 0, 0]
```

In [31]:

```
#Strictly Ryp1-regulated genes with Ryp1 motif hits
ryp1_strict_numhits = []
for gene in Cp_strict_ryp1_hit_genes: 
    hit_list = (genes_with_hit["CpSilveiraV3"][gene])
    ryp1_strict_numhits.append(len(hit_list))
    gene = genome.getGene(gene)
    
ryp1_strict_numhits_binned_short = [0,0,0,0,0,0]
for bin in range(0,4):
    for hit in ryp1_strict_numhits:
        if hit == bin + 1:
            ryp1_strict_numhits_binned_short[bin] = ryp1_strict_numhits_binned_short[bin] + 1 
for hit in ryp1_strict_numhits:
    if hit > 11:
        ryp1_strict_numhits_binned_short[5] = ryp1_strict_numhits_binned_short[5] + 1
    elif hit >=5:
        ryp1_strict_numhits_binned_short[4] = ryp1_strict_numhits_binned_short[4] + 1
print(ryp1_strict_numhits_binned_short)

nums_obs = len(ryp1_strict_numhits)
ryp1_strict_numhits_binned_short_norm = []
for entry in ryp1_strict_numhits_binned_short:
    ryp1_strict_numhits_binned_short_norm.append(float(entry)/float(nums_obs))
```

```
[27, 5, 2, 0, 0, 0]
```

In [32]:

```
# Morphology-regulated genes with Ryp1 motif hits

morph_numhits = []
for gene in Cp_morph_hit_genes:  
    hit_list = (genes_with_hit["CpSilveiraV3"][gene])
    morph_numhits.append(len(hit_list))
    gene = genome.getGene(gene)

morph_numhits_binned_short = [0,0,0,0,0,0]
for bin in range(0,4):
    for hit in morph_numhits:
        if hit == bin + 1:
            morph_numhits_binned_short[bin] = morph_numhits_binned_short[bin] + 1 
for hit in morph_numhits:
    if hit > 11:
        morph_numhits_binned_short[5] = morph_numhits_binned_short[5] + 1
    elif hit >=5:
        morph_numhits_binned_short[4] = morph_numhits_binned_short[4] + 1
print(morph_numhits_binned_short)

nums_obs = len(morph_numhits)
morph_numhits_binned_short_norm = []
for entry in morph_numhits_binned_short:
    morph_numhits_binned_short_norm.append(float(entry)/float(nums_obs))
```

```
[141, 42, 16, 6, 2, 0]
```

In [33]:

```
#Figure S4I
colors = ["plum", "darkturquoise", "yellow","crimson", "grey", "black"]

bins = ["all", "ryp1_strict", "ryp1_spher", "ryp1_myc", "morph"]
hits = {}
for color in colors:
    hits[color] = []
for j, color in enumerate(colors):
    hits[color].append(all_genes_numhits_binned_short_norm[j])
    hits[color].append(ryp1_strict_numhits_binned_short_norm[j])
    hits[color].append(ryp1_spher_numhits_binned_short_norm[j])
    hits[color].append(ryp1_myc_numhits_binned_short_norm[j])
    hits[color].append(morph_numhits_binned_short_norm[j])

bottom = np.zeros(5)
fig,ax = plt.subplots()
for color in colors:
    ax.bar(bins, hits[color], 0.5, color=color, bottom=bottom)
    bottom += hits[color]

plt.show()
plt.savefig("stackedbar_ryp1motif.svg")
```

### Looking at percentage of promoters in gene subsets with this motif hit¶

In [34]:

```
perc_total_genes = len(genes_with_hit["CpSilveiraV3"]) / len(all_genes_cdt)
print(perc_total_genes)
a = len(genes_with_hit["CpSilveiraV3"])
b = len(all_genes_cdt) - a
```

```
0.30332274615196675
```

In [35]:

```
ryp1_strict_motif = set(i.Uniqid() for i in strict_ryp1_cdt)
perc_strict = len(ryp1_strict_motif.intersection(Cp_hit_genes))/len(strict_ryp1_cdt)
print(perc_strict)
c = len(ryp1_strict_motif.intersection(Cp_hit_genes))
d = len(strict_ryp1_cdt) - c
```

```
0.43037974683544306
```

In [36]:

```
%%R -i a,b,c,d
matrix = matrix(c(a,b,c,d),nrow=2)
p <- fisher.test(matrix,alternative="two.sided")
print(p)
print(p$p.value)
```

```
	Fisher's Exact Test for Count Data

data:  matrix
p-value = 0.01904
alternative hypothesis: true odds ratio is not equal to 1
95 percent confidence interval:
 0.3600320 0.9301589
sample estimates:
odds ratio 
 0.5762867 

[1] 0.01903913
```

In [37]:

```
ryp1_spher_motif = set(i.Uniqid() for i in ryp1_spher)
ryp1_myc_motif = set(i.Uniqid() for i in ryp1_myc)
perc_spher = len(ryp1_spher_motif.intersection(Cp_hit_genes))/len(ryp1_spher)
print(perc_spher)
c = len(ryp1_spher_motif.intersection(Cp_hit_genes))
d = len(ryp1_spher) - c
```

```
0.413716814159292
```

In [38]:

```
%%R -i a,b,c,d
matrix = matrix(c(a,b,c,d),nrow=2)
p <- fisher.test(matrix,alternative="two.sided")
print(p)
print(p$p.value)
```

```
	Fisher's Exact Test for Count Data

data:  matrix
p-value = 1.386e-06
alternative hypothesis: true odds ratio is not equal to 1
95 percent confidence interval:
 0.5066373 0.7526263
sample estimates:
odds ratio 
 0.6170256 

[1] 1.386309e-06
```

In [39]:

```
perc_myc = len(ryp1_myc_motif.intersection(Cp_hit_genes))/len(ryp1_myc)
print(perc_myc)
c = len(ryp1_myc_motif.intersection(Cp_hit_genes))
d = len(ryp1_myc) - c
```

```
0.3969465648854962
```

In [40]:

```
%%R -i a,b,c,d
matrix = matrix(c(a,b,c,d),nrow=2)
p <- fisher.test(matrix,alternative="two.sided")
print(p)
print(p$p.value)
```

```
	Fisher's Exact Test for Count Data

data:  matrix
p-value = 0.001681
alternative hypothesis: true odds ratio is not equal to 1
95 percent confidence interval:
 0.5107555 0.8595938
sample estimates:
odds ratio 
 0.6614846 

[1] 0.001681456
```

In [41]:

```
arth_motif = set(i.Uniqid() for i in arth)
perc_arth = len(arth_motif.intersection(Cp_hit_genes))/len(arth)
print(perc_arth)
c = len(arth_motif.intersection(Cp_hit_genes))
d = len(arth) - c
```

```
0.30480689080300083
```

In [42]:

```
%%R -i a,b,c,d
matrix = matrix(c(a,b,c,d),nrow=2)
p <- fisher.test(matrix,alternative="two.sided")
print(p)
print(p$p.value)
```

```
	Fisher's Exact Test for Count Data

data:  matrix
p-value = 0.879
alternative hypothesis: true odds ratio is not equal to 1
95 percent confidence interval:
 0.9111949 1.0824906
sample estimates:
odds ratio 
 0.9930101 

[1] 0.8790249
```

In [43]:

```
morph_motif = set(i.Uniqid() for i in morph)
perc_morph = len(morph_motif.intersection(Cp_hit_genes))/len(morph)
print(perc_morph)
c = len(morph_motif.intersection(Cp_hit_genes))
d = len(morph) - c
```

```
0.37568058076225047
```

In [44]:

```
%%R -i a,b,c,d
matrix = matrix(c(a,b,c,d),nrow=2)
p <- fisher.test(matrix,alternative="two.sided")
print(p)
print(p$p.value)
```

```
	Fisher's Exact Test for Count Data

data:  matrix
p-value = 0.0004904
alternative hypothesis: true odds ratio is not equal to 1
95 percent confidence interval:
 0.6032049 0.8695994
sample estimates:
odds ratio 
 0.7235519 

[1] 0.000490408
```

In [45]:

```
#Figure 4H
fig = plt.figure()
categories = ["all", "ryp1_strict", "ryp1_spher", "ryp1_myc", "ryp1_arth", "morph"]
heights = [perc_total_genes,perc_strict,perc_spher,perc_myc,perc_arth,perc_morph]

plt.bar(categories,heights, color='grey')
plt.show()
plt.savefig("promoter_perc_hits.svg")
```

# Same analysis for motif found in only Spherule peaks¶

Invocations for scripts run on the command line:

IntergenicBackgrounds.py CpSilveiraV3

MetaMotif.py -E 0.000001 --bfile\_template CpSilveiraV3.ibg --multihit S\_only\_motif.meme 1 S\_only\_motif CpSilveiraV3

In [46]:

```
%cd ../S_only_Motif/
```

```
/home/chomer/Papers/Cocci_transcriptomics/data_for_code/Fig4/S_only_Motif
```

In [47]:

```
hits = {}
for genome in ([genome]):
    name = genome.Name()
    hits[name] = [MotifHit(i, genome) for i in Gff3file.fromFile(
            open("../S_only_motif.gff3","rt"))]
len(hits["CpSilveiraV3"])
```

Out[47]:

```
1907
```

In [48]:

```
hitgenes = {}
hitgenes["CpSilveiraV3"] = RefCollisions([item.hit for item in hits["CpSilveiraV3"]],intergenics)
```

In [49]:

```
genes_with_hit = {}
for (name, d) in hitgenes.items():
    genes_with_hit[name] = {}
    for i in hitgenes[name]:
        if i[1].Gene().Name() not in genes_with_hit[name].keys():
            genes_with_hit[name][i[1].Gene().Name()] = [i[0]]
        else:
            j = genes_with_hit[name][i[1].Gene().Name()]
            j.append(i[0])
            genes_with_hit[name][i[1].Gene().Name()] = j
```

### Intersect with Expression Data¶

In [50]:

```
Cp_hit_genes = set(i.Uniqid() for i in all_genes_cdt).intersection(set(genes_with_hit["CpSilveiraV3"]))
print(len(Cp_hit_genes))

Cp_strict_ryp1_hit_genes = set(i.Uniqid() for i in strict_ryp1_cdt).intersection(set(genes_with_hit["CpSilveiraV3"]))
print(len(Cp_strict_ryp1_hit_genes))

Cp_spher_ryp1_hit_genes = set(i.Uniqid() for i in ryp1_spher).intersection(set(genes_with_hit["CpSilveiraV3"]))
print(len(Cp_spher_ryp1_hit_genes))

Cp_myc_ryp1_hit_genes = set(i.Uniqid() for i in ryp1_myc).intersection(set(genes_with_hit["CpSilveiraV3"]))
print(len(Cp_myc_ryp1_hit_genes))

Cp_arth_ryp1_hit_genes = set(i.Uniqid() for i in arth).intersection(set(genes_with_hit["CpSilveiraV3"]))
print(len(Cp_arth_ryp1_hit_genes))

Cp_morph_hit_genes = set(i.Uniqid() for i in morph).intersection(set(genes_with_hit["CpSilveiraV3"]))
print(len(Cp_morph_hit_genes))
```

```
1589
18
120
55
710
146
```

### Plot histogram of distance from motif to gene start¶

In [51]:

```
#Figure S4J
#plot histogram of distance from motif to gene start using ATG to be consistent with promoter length analysis
motif_distance_to_ATG = []
for gene in Cp_hit_genes:  
    hit_list = (genes_with_hit["CpSilveiraV3"][gene])
    gene = genome.getGene(gene)
    for item in hit_list:
        hit_strand = item.strand
        hit_start = item.start
        hit_stop = item.stop
        gene_strand = gene.Locus().strand
        coords = []
        for cds in gene.CdsLoci():
            coords.append(cds.start)
            coords.append(cds.stop)            
        if gene_strand == "+":
            atg = min(coords)
            distance_to_ATG = atg - hit_start
        elif gene_strand == "-":
            atg = max(coords)
            distance_to_ATG = hit_stop - atg

        if distance_to_ATG < 15001:
                motif_distance_to_ATG.append(distance_to_ATG)


fig = plt.figure()
ax = plt.subplot(111)
plt.hist(motif_distance_to_ATG, color='grey')
plt.show()
plt.savefig("motif_distance_to_ATG.svg")
```

### Compare number of motif hits in different gene subsets¶

In [52]:

```
#all genes with Spherule-only motif hits
all_genes_numhits = []
for gene in Cp_hit_genes:  
    hit_list = (genes_with_hit["CpSilveiraV3"][gene])
    gene = genome.getGene(gene)
    all_genes_numhits.append(len(hit_list))
    
all_genes_numhits_binned_short = [0,0,0,0,0,0]

for bin in range(0,4):
    for hit in all_genes_numhits:
        if hit == bin + 1:
            all_genes_numhits_binned_short[bin] = all_genes_numhits_binned_short[bin] + 1 
for hit in all_genes_numhits:
    if hit >11:
        all_genes_numhits_binned_short[5] = all_genes_numhits_binned_short[5] + 1
    elif hit >= 5: 
        all_genes_numhits_binned_short[4] = all_genes_numhits_binned_short[4] + 1

print(all_genes_numhits_binned_short)
nums_obs = len(all_genes_numhits)
all_genes_numhits_binned_short_norm = []
for entry in all_genes_numhits_binned_short:
    all_genes_numhits_binned_short_norm.append(entry/float(nums_obs))
```

```
[1174, 278, 87, 33, 17, 0]
```

In [53]:

```
#Ryp1-spher-regulated genes with Spherule-only motif hits
ryp1_spher_numhits = []
for gene in Cp_spher_ryp1_hit_genes:  
    hit_list = (genes_with_hit["CpSilveiraV3"][gene])
    ryp1_spher_numhits.append(len(hit_list))
    gene = genome.getGene(gene)
    
ryp1_spher_numhits_binned_short = [0,0,0,0,0,0]
for bin in range(0,4):
    for hit in ryp1_spher_numhits:
        if hit == bin + 1:
            ryp1_spher_numhits_binned_short[bin] = ryp1_spher_numhits_binned_short[bin] + 1 
for hit in ryp1_spher_numhits:
    if hit > 11:
        ryp1_spher_numhits_binned_short[5] = ryp1_spher_numhits_binned_short[5] + 1
    elif hit >=5:
        ryp1_spher_numhits_binned_short[4] = ryp1_spher_numhits_binned_short[4] + 1
print(ryp1_spher_numhits_binned_short)

nums_obs = len(ryp1_spher_numhits)
ryp1_spher_numhits_binned_short_norm = []
for entry in ryp1_spher_numhits_binned_short:
    ryp1_spher_numhits_binned_short_norm.append(entry/float(nums_obs))
```

```
[88, 20, 3, 4, 5, 0]
```

In [54]:

```
#Ryp1-myc-regulated genes with Spherule only motif hits
ryp1_myc_numhits = []
for gene in Cp_myc_ryp1_hit_genes:  
    hit_list = (genes_with_hit["CpSilveiraV3"][gene])
    ryp1_myc_numhits.append(len(hit_list))
    gene = genome.getGene(gene)
    
ryp1_myc_numhits_binned_short = [0,0,0,0,0,0]
for bin in range(0,4):
    for hit in ryp1_myc_numhits:
        if hit == bin + 1:
            ryp1_myc_numhits_binned_short[bin] = ryp1_myc_numhits_binned_short[bin] + 1 
for hit in ryp1_myc_numhits:
    if hit > 11:
        ryp1_myc_numhits_binned_short[5] = ryp1_myc_numhits_binned_short[5] + 1
    elif hit >=5:
        ryp1_myc_numhits_binned_short[4] = ryp1_myc_numhits_binned_short[4] + 1
print(ryp1_myc_numhits_binned_short)

nums_obs = len(ryp1_myc_numhits)
ryp1_myc_numhits_binned_short_norm = []
for entry in ryp1_myc_numhits_binned_short:
    ryp1_myc_numhits_binned_short_norm.append(entry/float(nums_obs))
```

```
[45, 6, 2, 0, 2, 0]
```

In [55]:

```
#Strictly Ryp1-regulated genes with Spherule only motif hits
ryp1_strict_numhits = []
for gene in Cp_strict_ryp1_hit_genes: 
    hit_list = (genes_with_hit["CpSilveiraV3"][gene])
    ryp1_strict_numhits.append(len(hit_list))
    gene = genome.getGene(gene)
    
ryp1_strict_numhits_binned_short = [0,0,0,0,0,0]
for bin in range(0,4):
    for hit in ryp1_strict_numhits:
        if hit == bin + 1:
            ryp1_strict_numhits_binned_short[bin] = ryp1_strict_numhits_binned_short[bin] + 1 
for hit in ryp1_strict_numhits:
    if hit > 11:
        ryp1_strict_numhits_binned_short[5] = ryp1_strict_numhits_binned_short[5] + 1
    elif hit >=5:
        ryp1_strict_numhits_binned_short[4] = ryp1_strict_numhits_binned_short[4] + 1
print(ryp1_strict_numhits_binned_short)

nums_obs = len(ryp1_strict_numhits)
ryp1_strict_numhits_binned_short_norm = []
for entry in ryp1_strict_numhits_binned_short:
    ryp1_strict_numhits_binned_short_norm.append(float(entry)/float(nums_obs))
```

```
[14, 1, 2, 0, 1, 0]
```

In [56]:

```
# Morphology-regulated genes with Spherule only motif hits

morph_numhits = []
for gene in Cp_morph_hit_genes:  
    hit_list = (genes_with_hit["CpSilveiraV3"][gene])
    morph_numhits.append(len(hit_list))
    gene = genome.getGene(gene)

morph_numhits_binned_short = [0,0,0,0,0,0]
for bin in range(0,4):
    for hit in morph_numhits:
        if hit == bin + 1:
            morph_numhits_binned_short[bin] = morph_numhits_binned_short[bin] + 1 
for hit in morph_numhits:
    if hit > 11:
        morph_numhits_binned_short[5] = morph_numhits_binned_short[5] + 1
    elif hit >=5:
        morph_numhits_binned_short[4] = morph_numhits_binned_short[4] + 1
print(morph_numhits_binned_short)

nums_obs = len(morph_numhits)
morph_numhits_binned_short_norm = []
for entry in morph_numhits_binned_short:
    morph_numhits_binned_short_norm.append(float(entry)/float(nums_obs))
```

```
[107, 25, 8, 4, 2, 0]
```

In [57]:

```
#Figure S4K
colors = ["plum", "darkturquoise", "yellow","crimson", "grey", "black"]

bins = ["all", "ryp1_strict", "ryp1_spher", "ryp1_myc", "morph"]
hits = {}
for color in colors:
    hits[color] = []
for j, color in enumerate(colors):
    hits[color].append(all_genes_numhits_binned_short_norm[j])
    hits[color].append(ryp1_strict_numhits_binned_short_norm[j])
    hits[color].append(ryp1_spher_numhits_binned_short_norm[j])
    hits[color].append(ryp1_myc_numhits_binned_short_norm[j])
    hits[color].append(morph_numhits_binned_short_norm[j])

bottom = np.zeros(5)
fig,ax = plt.subplots()
for color in colors:
    ax.bar(bins, hits[color], 0.5, color=color, bottom=bottom)
    bottom += hits[color]

plt.show()
plt.savefig("stackedbar_ryp1motif.svg")
```

### Looking at percentage of promoters in gene subsets with this motif hit¶

In [58]:

```
perc_total_genes = len(genes_with_hit["CpSilveiraV3"]) / len(all_genes_cdt)
print(perc_total_genes)
a = len(genes_with_hit["CpSilveiraV3"])
b = len(all_genes_cdt) - a
```

```
0.2008306865379917
```

In [59]:

```
ryp1_strict_motif = set(i.Uniqid() for i in strict_ryp1_cdt)
perc_strict = len(ryp1_strict_motif.intersection(Cp_hit_genes))/len(strict_ryp1_cdt)
print(perc_strict)
c = len(ryp1_strict_motif.intersection(Cp_hit_genes))
d = len(strict_ryp1_cdt) - c
```

```
0.22784810126582278
```

In [60]:

```
%%R -i a,b,c,d
matrix = matrix(c(a,b,c,d),nrow=2)
p <- fisher.test(matrix,alternative="two.sided")
print(p)
print(p$p.value)
```

```
	Fisher's Exact Test for Count Data

data:  matrix
p-value = 0.5723
alternative hypothesis: true odds ratio is not equal to 1
95 percent confidence interval:
 0.4950235 1.5356209
sample estimates:
odds ratio 
 0.8516139 

[1] 0.5722703
```

In [61]:

```
ryp1_spher_motif = set(i.Uniqid() for i in ryp1_spher)
ryp1_myc_motif = set(i.Uniqid() for i in ryp1_myc)
perc_spher = len(ryp1_spher_motif.intersection(Cp_hit_genes))/len(ryp1_spher)
print(perc_spher)
c = len(ryp1_spher_motif.intersection(Cp_hit_genes))
d = len(ryp1_spher) - c
```

```
0.26548672566371684
```

In [62]:

```
%%R -i a,b,c,d
matrix = matrix(c(a,b,c,d),nrow=2)
p <- fisher.test(matrix,alternative="two.sided")
print(p)
print(p$p.value)
```

```
	Fisher's Exact Test for Count Data

data:  matrix
p-value = 0.001196
alternative hypothesis: true odds ratio is not equal to 1
95 percent confidence interval:
 0.5584843 0.8701673
sample estimates:
odds ratio 
 0.6952939 

[1] 0.00119637
```

In [63]:

```
perc_myc = len(ryp1_myc_motif.intersection(Cp_hit_genes))/len(ryp1_myc)
print(perc_myc)
c = len(ryp1_myc_motif.intersection(Cp_hit_genes))
d = len(ryp1_myc) - c
```

```
0.2099236641221374
```

In [64]:

```
%%R -i a,b,c,d
matrix = matrix(c(a,b,c,d),nrow=2)
p <- fisher.test(matrix,alternative="two.sided")
print(p)
print(p$p.value)
```

```
	Fisher's Exact Test for Count Data

data:  matrix
p-value = 0.696
alternative hypothesis: true odds ratio is not equal to 1
95 percent confidence interval:
 0.6956929 1.3041988
sample estimates:
odds ratio 
  0.945799 

[1] 0.6959977
```

In [65]:

```
arth_motif = set(i.Uniqid() for i in arth)
perc_arth = len(arth_motif.intersection(Cp_hit_genes))/len(arth)
print(perc_arth)
c = len(arth_motif.intersection(Cp_hit_genes))
d = len(arth) - c
```

```
0.1972770213948319
```

In [66]:

```
%%R -i a,b,c,d
matrix = matrix(c(a,b,c,d),nrow=2)
p <- fisher.test(matrix,alternative="two.sided")
print(p)
print(p$p.value)
```

```
	Fisher's Exact Test for Count Data

data:  matrix
p-value = 0.6707
alternative hypothesis: true odds ratio is not equal to 1
95 percent confidence interval:
 0.9259179 1.1298954
sample estimates:
odds ratio 
   1.02254 

[1] 0.6707062
```

In [67]:

```
morph_motif = set(i.Uniqid() for i in morph)
perc_morph = len(morph_motif.intersection(Cp_hit_genes))/len(morph)
print(perc_morph)
c = len(morph_motif.intersection(Cp_hit_genes))
d = len(morph) - c
```

```
0.26497277676951
```

In [68]:

```
%%R -i a,b,c,d
matrix = matrix(c(a,b,c,d),nrow=2)
p <- fisher.test(matrix,alternative="two.sided")
print(p)
print(p$p.value)
```

```
	Fisher's Exact Test for Count Data

data:  matrix
p-value = 0.0004694
alternative hypothesis: true odds ratio is not equal to 1
95 percent confidence interval:
 0.5709133 0.8547962
sample estimates:
odds ratio 
 0.6971293 

[1] 0.0004694038
```

In [69]:

```
#Figure 4J
fig = plt.figure()
categories = ["all", "ryp1_strict", "ryp1_spher", "ryp1_myc", "ryp1_arth", "morph"]
heights = [perc_total_genes,perc_strict,perc_spher,perc_myc,perc_arth,perc_morph]

plt.bar(categories,heights, color='grey')
plt.show()
plt.savefig("promoter_perc_hits.svg")
```

# Calculating intergenic length for each gene subset¶

In [70]:

```
all_intergenic_lens = []
for intergenic in intergenics:
    locus = intergenic.Locus()
    length = abs(locus.start - locus.stop)
    all_intergenic_lens.append(length)
```

In [71]:

```
ryp1_strict_lens = []
ryp1_spherule_lens = []
ryp1_myc_lens = []
morph_lens = []
arth_lens = []

for intergenic in intergenics:
    locus = intergenic.Locus()
    gene = str(intergenic.gene)
    length = abs(locus.start - locus.stop)
    for i in strict_ryp1_cdt:
        d8b26 = "_".join(i.Uniqid().split("_")[0:2])
        if d8b26 == gene:
            ryp1_strict_lens.append(length)
    for i in ryp1_spher:
        d8b26 = "_".join(i.Uniqid().split("_")[0:2])
        if d8b26 == gene:
            ryp1_spherule_lens.append(length)
    for i in ryp1_myc:
        d8b26 = "_".join(i.Uniqid().split("_")[0:2])
        if d8b26 == gene:
            ryp1_myc_lens.append(length)
    for i in morph:
        d8b26 = "_".join(i.Uniqid().split("_")[0:2])
        if d8b26 == gene:
            morph_lens.append(length)
    for i in arth:
        d8b26 = "_".join(i.Uniqid().split("_")[0:2])
        if d8b26 == gene:
            arth_lens.append(length)
```

In [72]:

```
#Fig 4L
fig = plt.figure()
categories = ["all", "ryp1_strict", "ryp1_spher", "ryp1_myc", "morph"]
heights = [np.mean(all_intergenic_lens),np.mean(ryp1_strict_lens),np.mean(ryp1_spherule_lens),np.mean(ryp1_myc_lens), np.mean(morph_lens)]
stdev = [np.std(all_intergenic_lens), np.std(ryp1_strict_lens), np.std(ryp1_spherule_lens), np.std(ryp1_myc_lens),  np.std(morph_lens)]

plt.bar(categories,heights, color='grey')
plt.show()
plt.savefig("bar_intergeniclength_noarth.svg")
```

In [73]:

```
stats.ttest_ind(all_intergenic_lens,ryp1_spherule_lens)
```

Out[73]:

```
Ttest_indResult(statistic=-6.850634438455482, pvalue=7.850701585405382e-12)
```

In [74]:

```
stats.ttest_ind(all_intergenic_lens,ryp1_strict_lens)
```

Out[74]:

```
Ttest_indResult(statistic=-1.0723183513862793, pvalue=0.28360807195775994)
```

In [75]:

```
stats.ttest_ind(all_intergenic_lens,ryp1_myc_lens)
```

Out[75]:

```
Ttest_indResult(statistic=-0.30389201956817113, pvalue=0.7612175710615248)
```

In [76]:

```
stats.ttest_ind(all_intergenic_lens,morph_lens)
```

Out[76]:

```
Ttest_indResult(statistic=-5.969885299604651, pvalue=2.4659328945501545e-09)
```

In [ ]:

```

```
