# Supplementary material for: Transcriptomic atlas throughout Coccidioides development reveals key phase-enriched transcripts of this important fungal pathogen
Source: PLoS Biol. 2025 Apr 15;23(4):e3003066. doi: 10.1371/journal.pbio.3003066 (PMC12077801; doi:10.1371/journal.pbio.3003066)
Supplement: S1 Code — Folder containing README document describing the scripts used to analyze the data and generate figures in this manuscript, as well as the scripts themselves and custom python three modules used in the scripts. (ZIP) [file pbio.3003066.s025.zip › Custom Code/notebooks/Figure1_and_S1_and_6B.html]

Figure1\_and\_S1\_and\_6B


In [1]:

```
cd ../../Papers/Cocci_transcriptomics/data_for_code/Fig1/
```

```
/home/chomer/Papers/Cocci_transcriptomics/data_for_code/Fig1
```

In [2]:

```
%load_ext rpy2.ipython
from glob import glob
from MsvUtil import Table
import os.path
from CdtFile import CdtFile, CdtRow
from SafeMath import safelog
from ReadCountTools import PseudoCPMs
from PCA import PCA
import numpy as np
import matplotlib.patches as patches
import matplotlib.transforms as transforms
import matplotlib.pyplot as plt
%matplotlib nbagg
from csv import writer, excel_tab
from LimmaTools import SingleFactorFit
import statistics
```

In [3]:

```
%%R
library(limma)
library(edgeR)
```

# Merge Kallisto TPMs¶

In [4]:

```
#First Index datasets

sname2kallisto = dict(
    (i.replace(".Silv_nanopore_mRNA.rf.kallisto","").split("_")[0],i)
              for i in glob("*.Silv_nanopore_mRNA.rf.kallisto") if(i.find("Undetermined") < 0))
snames = sorted(sname2kallisto, key = lambda x: (x[4:x.rfind("_")],x[:4]))

print(len(sname2kallisto))
print(snames)
```

```
24
['8h-1', '8h-2', '8h-3', 'Day1-3', 'Day1-2', 'Day1-1', 'Day2-3', 'Day2-2', 'Day2-1', 'Day3-1', 'Day3-2', 'Day3-3', 'Day4-2', 'Day4-3', 'Day4-1', 'Day5-1', 'Day5-3', 'Day5-2', 'Day6-3', 'Day6-1', 'Day6-2', 'Spores1', 'Spores2', 'Spores3']
```

In [5]:

```
#Merge Step
genes = None
cols = []
counts = []
for i in snames:
    table = Table.fromTdt(open(os.path.join(
           sname2kallisto[i],
          "abundance.tsv")))
    if(genes is None):
        genes = table["target_id"]
    else:
        assert(genes == table["target_id"])
    cols.append([float(i) for i in table["tpm"]])
    counts.append([int(float(i)+.5) for i in table["est_counts"]])
#print(counts)

tpm_trans = CdtFile(probes = [CdtRow(gid = i[0], uniqid = i[0], name = i[0],
                                        ratios = [safelog(j) for j in i[1:]])
                                 for i in zip(*([genes]+cols))],
                       fieldnames = snames,
                       eweights = [1]*len(snames))
tpm_trans.write(open("tpm_trans.cdt","w"))

trans_counts = CdtFile(probes = [CdtRow(gid = i[0], uniqid = i[0], name = i[0],
                                        ratios = i[1:])
                                 for i in zip(*([genes]+counts))],
                       fieldnames = snames,
                       eweights = [1]*len(snames))
trans_counts.write(open("trans_counts.cdt","w"))

len(tpm_trans), len(trans_counts)
```

Out[5]:

```
(8628, 8628)
```

In [6]:

```
merge_pseudo = PseudoCPMs.fromCounts(trans_counts)
#pseudoCPMs are log transformed
#counts are to feed directly to limma
```

In [7]:

```
#filter out transcripts that do not have at least 10 reads in at least 1 or more datasets
mask_10 = merge_pseudo.depth_filter_mask(10,.01)
mask_10.counts.write(open("Fig1c_counts_pseudoCPMs_for_paper.cdt", "w"))
```

In [8]:

```
ct_counts = CdtFile.fromCdt("Fig1c_counts_pseudoCPMs_for_paper.cdt")
len(ct_counts)
```

Out[8]:

```
8171
```

# Create PCA plot¶

In [9]:

```
def fix_of_none_value(i):
    if i is None:
        return 0.
    return i
```

In [10]:

```
centered_rows = mask_10.cpms.mean_normalize_rows()
fixed_cdt = CdtFile.fromPrototype(centered_rows, probes = [CdtRow.fromPrototype(i, ratios=[fix_of_none_value(j) for j in i]) for i in centered_rows])
```

In [11]:

```
pca = PCA.fromCdt(fixed_cdt.transpose(), normalization=None)
fig = pca.plot_relative_variance()
```

In [12]:

```
color_dict = {"8h": "red", "Day1": "orange", "Day2": "gold", "Day3": "green", "Day4": "blue", "Day5": "cyan", "Day6": "purple", "Spores": "black","Spore": "black"}
highlights = {}

for i in snames:
    if i[0:3] == "Spo":
        tp = i[:-1]
        marker = '*'
    else:
        tp = i.split("-")[0]
        if int(i.split("-")[-1]) == 1:
            marker = 'o'
        elif int(i.split("-")[-1]) == 2: 
            marker = 'o'
        elif int(i.split("-")[-1]) == 3:
            marker = "o"
    color = color_dict[tp]
    
    highlights[i] = (color,marker)
```

In [13]:

```
def maxDiff(a):
    vmin = a[0]
    dmax = 0
    for i in range(len(a)):
        if (a[i] < vmin):
            vmin = a[i]
        elif (a[i] - vmin > dmax):
            dmax = a[i] - vmin
    return dmax
```

In [14]:

```
#from matplotlib example library
def confidence_ellipse(x, y, ax, n_std=3.0, facecolor='none', **kwargs):
    """
    Create a plot of the covariance confidence ellipse of *x* and *y*.

    Parameters
    ----------
    x, y : array-like, shape (n, )
        Input data.

    ax : matplotlib.axes.Axes
        The Axes object to draw the ellipse into.

    n_std : float
        The number of standard deviations to determine the ellipse's radiuses.

    **kwargs
        Forwarded to `~matplotlib.patches.Ellipse`

    Returns
    -------
    matplotlib.patches.Ellipse
    """
    if x.size != y.size:
        raise ValueError("x and y must be the same size")

    cov = np.cov(x, y)
    pearson = cov[0, 1]/np.sqrt(cov[0, 0] * cov[1, 1])
    # Using a special case to obtain the eigenvalues of this
    # two-dimensional dataset.
    ell_radius_x = np.sqrt(1 + pearson)
    ell_radius_y = np.sqrt(1 - pearson)
    ellipse = patches.Ellipse((0, 0), width=ell_radius_x * 2, height=ell_radius_y * 2,
                      facecolor=facecolor, **kwargs)

    # Calculating the standard deviation of x from
    # the squareroot of the variance and multiplying
    # with the given number of standard deviations.
    scale_x = np.sqrt(cov[0, 0]) * n_std
    mean_x = np.mean(x)

    # calculating the standard deviation of y ...
    scale_y = np.sqrt(cov[1, 1]) * n_std
    mean_y = np.mean(y)

    transf = transforms.Affine2D() \
        .rotate_deg(45) \
        .scale(scale_x, scale_y) \
        .translate(mean_x, mean_y)

    ellipse.set_transform(transf + ax.transData)
    return ax.add_patch(ellipse)
```

In [15]:

```
#Figure S1F
x=0
y=1
previous_name = "8h"
circle_points_x = []
circle_points_y = []
fig = pca.plot_2D_projection(x=x,y=y,marker=".",color="black")
for (n, name) in enumerate(pca.rows):
    (color,marker) = highlights[name]
    pca.plot_2D_projection(fig.axes[0], x = x, y = y, color = color, marker = marker, rows = [n])
    X=pca.projected[n,0]
    Y=pca.projected[n,1]
    if name[:-2] == previous_name:
        circle_points_x.append(X)
        circle_points_y.append(Y)
    else:
        confidence_ellipse(np.array(circle_points_x),np.array(circle_points_y),fig.axes[0],edgecolor=color_dict[previous_name])
        circle_points_x = [X]
        circle_points_y = [Y]
    previous_name = name[:-2]

    
confidence_ellipse(np.array(circle_points_x),np.array(circle_points_y),fig.axes[0], edgecolor=color_dict[previous_name])  
plt.show()    
fig.savefig("PCA0_vs_1.svg")
```

# Limma analysis¶

In [16]:

```
#need to format ct_counts for limma input

fout = open("ct_counts.txt", "w")

fout.write("\t".join(["gene"]+ct_counts.fieldnames)+"\n")
for row in mask_10.counts:
    fout.write("\t".join([row.Uniqid()]+[str(i) for i in row])+"\n")
fout.close()
```

In [17]:

```
%%R
#Limma Single Factor Fit
# Read the count matrix, using the gene column as row names
C <- read.delim("ct_counts.txt",row.names=1)
#Convert the matrix to limma's preferred format, implicitly log2 transforming and depth normalizing to CPM values
dge <- DGEList(counts=C)
```

In [18]:

```
out = writer(open("Fig1_simple_comp_samples.txt","w"),dialect = excel_tab)
out.writerow(("run","state"))
for i in snames:
    run = i
    if i[0:3] == "Spo":
        state = i[:-1]
    elif i[0] == "8":
        state = "EightHour"
    else:
        state = i.split("-")[0]
    out.writerow((run, state))    
del out
```

In [19]:

```
%%R -o d
samples <- read.delim("Fig1_simple_comp_samples.txt", header=TRUE, sep="\t")
print(summary(samples))
state <- samples$state
d <- model.matrix(~0+state)
colnames(d) <- gsub("state","",colnames(d))
print(colnames(d))
```

```
     run               state          
 Length:24          Length:24         
 Class :character   Class :character  
 Mode  :character   Mode  :character  
[1] "Day1"      "Day2"      "Day3"      "Day4"      "Day5"      "Day6"     
[7] "EightHour" "Spores"
```

In [20]:

```
%%R
# Apply between-sample TMM normalization
dge <- calcNormFactors(dge)
# Estimate the mean-variance trend via locally-linear regression and use this trend
# to assign weights to the observations (counts)
v <- voom(dge, d, plot = TRUE)
cpm <- v$E
```

In [21]:

```
%%R -o cpm,fc,cn,state
# Fit the model (classic linear regression)
fit <- lmFit(v, d)
#Generate the contrast matrix
contrast.matrix <- makeContrasts(
    EightHour - Spores, Day1 - Spores, Day2 - Spores, Day3 - Spores, Day4 - Spores, Day5 - Spores, Day6 - Spores,
    Day1 - EightHour, Day2 - Day1, Day3 - Day2, Day4 - Day3, Day5 - Day4, Day6 - Day5, 
    levels=d)
# Apply the contrast matrix
fit2 <- contrasts.fit(fit, contrast.matrix)
# Apply Empirical Bayes "shrinkage"
fit2 <- eBayes(fit2)
# Simple summary of significantly differential genes with no fold change filter
print(summary(decideTests(fit2)))

fc <- fit$coefficients
cn <- colnames(fit$coefficients)
cpm <- v$E
```

```
       EightHour - Spores Day1 - Spores Day2 - Spores Day3 - Spores
Down                 2983          3147          3110          3025
NotSig               2484          2070          2098          2113
Up                   2704          2954          2963          3033
       Day4 - Spores Day5 - Spores Day6 - Spores Day1 - EightHour Day2 - Day1
Down            2796          2873          2416             2153        1257
NotSig          2479          2325          3137             3957        5792
Up              2896          2973          2618             2061        1122
       Day3 - Day2 Day4 - Day3 Day5 - Day4 Day6 - Day5
Down           739         292          42           0
NotSig        6520        7507        8115        8169
Up             912         372          14           2
```

In [22]:

```
%%R
print(summary(decideTests(fit2,lfc=1)))
#Used for Fig S1E
```

```
       EightHour - Spores Day1 - Spores Day2 - Spores Day3 - Spores
Down                 1864          2120          2056          1973
NotSig               4734          4131          4146          4227
Up                   1573          1920          1969          1971
       Day4 - Spores Day5 - Spores Day6 - Spores Day1 - EightHour Day2 - Day1
Down            1741          1826          1633              815         304
NotSig          4526          4323          4635             6287        7394
Up              1904          2022          1903             1069         473
       Day3 - Day2 Day4 - Day3 Day5 - Day4 Day6 - Day5
Down           171          75          18           0
NotSig        7613        7920        8147        8169
Up             387         176           6           2
```

In [23]:

```
name2row = dict((i.Uniqid(),n+1) for (n,i) in enumerate(ct_counts))
```

In [24]:

```
fit = SingleFactorFit(fc, cpm, name2row, cn, 
                      state, obs_samples = ct_counts.fieldnames, parameter_order = ("Spores","EightHour","Day1", "Day2", "Day3", "Day4", "Day5", "Day6"))
fit.toHDF5("Fig1_singlecomp_limma1.hdf5")
fit2 = SingleFactorFit.fromHDF5("Fig1_singlecomp_limma1.hdf5")
#used as input for Figure 6B
```

In [25]:

```
%%R
write.csv(cpm,"limma1.countscutoff.cpm.csv")

for(tc in colnames(fit2$coefficients)){
  print(tc)
  # Extract all genes significantly differential on this contrast for a 2x fold change cutoff and 5% FDR
  # Use write.csv rather than write.table for clean compatibility with python's csv.reader
  write.csv(topTable(fit2, coef=tc, n = 50000, lfc=1, p.value = .05),
            paste("limma1.",gsub(" ","",tc),".t0.csv",sep=""))
  # Extract the adjusted p-values for this contrast for all genes, independent of significance
  write.csv(topTable(fit2, coef=tc, n = 50000),
            paste("limma1.",gsub(" ","",tc),".t1.csv",sep=""))
}
```

```
[1] "EightHour - Spores"
[1] "Day1 - Spores"
[1] "Day2 - Spores"
[1] "Day3 - Spores"
[1] "Day4 - Spores"
[1] "Day5 - Spores"
[1] "Day6 - Spores"
[1] "Day1 - EightHour"
[1] "Day2 - Day1"
[1] "Day3 - Day2"
[1] "Day4 - Day3"
[1] "Day5 - Day4"
[1] "Day6 - Day5"
```

In [26]:

```
gene2cpms = dict((i[0],[float(j) for j in i[1:]]) for i in Table.fromCsv("limma1.countscutoff.cpm.csv"))
len(gene2cpms), len(ct_counts)
```

Out[26]:

```
(8171, 8171)
```

In [27]:

```
limma1_cdt = CdtFile.fromPrototype(ct_counts, 
                                   probes = [CdtRow.fromPrototype(i, ratios = gene2cpms[i.Uniqid()][:])
                                             for i in ct_counts])
limma1_cdt = limma1_cdt.mean_normalize_rows()
#This spreadsheet was used for heatmap 1C after clustering with k-means in Cluster3.0
```

In [28]:

```
#Generate columns indicating if a comparison is significant (2-fold cutoff and 5% FDR)

gene2contrasts = dict((i.Uniqid(),[]) for i in limma1_cdt)
gene2pvals = dict((i.Uniqid(),[]) for i in limma1_cdt)
gene2sigs = dict((i.Uniqid(),[]) for i in limma1_cdt)
contrast_names = []

contrast_csvs = sorted(glob("limma1.*.t1.csv"))
# put 8 hour first
contrast_csvs = contrast_csvs[-1:]+contrast_csvs[:-1]
for i in contrast_csvs:
    cname = i.replace("limma1.","").replace(".t1.csv","").replace("-","/")
    contrast_names.append(cname)
    siglist = set(i[0] for i in Table.fromCsv(i.replace(".t1.",".t0.")))
    print(cname,len(siglist))
    for gene in Table.fromCsv(i):
        name = gene[0]
        lfc = float(gene["logFC"])
        gene2contrasts[name].append(lfc)
        gene2pvals[name].append(gene["adj.P.Val"])
        if(name in siglist):
            if(lfc > 0):
                gene2sigs[name].append(4.)
            else:
                gene2sigs[name].append(-4.)
        else:
            gene2sigs[name].append(0.)
        
limma1_cdt = CdtFile.fromPrototype(limma1_cdt,
    probes = [CdtRow.fromPrototype(i, ratios = i.ratios+gene2contrasts[i.Uniqid()]+gene2sigs[i.Uniqid()],
                                   extra = i.extra+gene2pvals[i.Uniqid()])
              for i in limma1_cdt],
    fieldnames = limma1_cdt.fieldnames+contrast_names+["%s_sig" % i for i in contrast_names],
    eweights = limma1_cdt.eweights+[1.]*2*len(contrast_names),
    extranames = limma1_cdt.extranames+["p(%s)" % i for i in contrast_names])
```

```
EightHour/Spores 3437
Day1/EightHour 1884
Day1/Spores 4040
Day2/Day1 777
Day2/Spores 4025
Day3/Day2 558
Day3/Spores 3944
Day4/Day3 251
Day4/Spores 3645
Day5/Day4 24
Day5/Spores 3848
Day6/Day5 2
Day6/Spores 3536
```

In [29]:

```
#This heatmap includes the list of genes used in downstream analysis to generate candidate endospore-related transcripts
sig_cols = [n for (n,i) in enumerate(limma1_cdt.fieldnames) if(i.endswith("_sig"))]
contrast_cols = [n-len(sig_cols) for n in sig_cols]
limma1_2x = CdtFile.fromPrototype(limma1_cdt, probes = [i for i in limma1_cdt 
                                                        if(any([(i[j] != 0.) for j in sig_cols]))])
tree = limma1_2x.cluster(cols=contrast_cols,dist="u",method="m")
limma1_2x.writeCdtGtr("limma1_2x.countscutoff.contrasts_um",tree)
len(limma1_2x)
```

```
Building array...
Building distance matrix...
Clustering...
```

Out[29]:

```
6355
```

# Make Pearson Correlation plots between each dataset¶

In [30]:

```
%%R
library(pheatmap)
```

In [31]:

```
%%R
#Limma Single Factor Fit
# Read the count matrix, using the gene column as row names
C <- read.delim("ct_counts.txt",row.names=1)
```

In [32]:

```
%%R -o corr_coeff
new_order = sort(colnames(C))
C <- C[, sort(colnames(C))]
C <- C[, c((ncol(C)-5):ncol(C),1:(ncol(C)-6))]
corr_coeff <- cor(C, method = "pearson")
```

In [33]:

```
#Fig S1D
fig = plt.figure()
plt.imshow(corr_coeff, vmin=0, vmax=1)
plt.colorbar()
plt.tight_layout()
plt.show()
plt.savefig("FigS1_pearson_correlation.svg")
```

# Graphing expression of dityrosine cluster¶

In [34]:

```
#Figure 6B
gene_list = [[[("D8B26_005432", "cyan"), ("D8B26_005433", "cyan")], [("D8B26_005434", "green")],[("D8B26_005435", "purple")]], [[("D8B26_005436", "grey")], [("D8B26_005437", "black")], [("D8B26_005438", "red")]]]
fig,ax = plt.subplots(2,3,figsize = (10,5))
offset = 0
j=0
while j <2:
    for k,sub_plots in enumerate(gene_list[j]):
        for (gene, color)in sub_plots:
            fit2 = fit.get_transcript(gene)
            parameter_order = fit2.fit.parameter_order
            xticklabels = parameter_order
            plot_cols = fit2.fit.plot_cols
            colors = [color]*len(parameter_order)

            for (x,(i,c)) in enumerate(zip(plot_cols,colors)):
                # Plot data 
                ys = fit2.obs[i]
                y_stdev = statistics.stdev(ys)

                # Plot fit parameters                                                                                                                                                                                                                    
                y = fit2.parameters[i]
                ax[j][k].plot(x+offset,y,"o", color=c)
                ax[j][k].errorbar(x+offset, y, yerr = y_stdev, color=c)
        offset += 0.1
    j += 1

ax[0][0].set_ylim(0,8)
plt.tight_layout()
plt.savefig("dits_dtr1_and_neighboring_cluster_graphed_together.svg")
```

In [ ]:

```

```
