# Supplementary material for: Transcriptomic atlas throughout Coccidioides development reveals key phase-enriched transcripts of this important fungal pathogen
Source: PLoS Biol. 2025 Apr 15;23(4):e3003066. doi: 10.1371/journal.pbio.3003066 (PMC12077801; doi:10.1371/journal.pbio.3003066)
Supplement: S1 Code — Folder containing README document describing the scripts used to analyze the data and generate figures in this manuscript, as well as the scripts themselves and custom python three modules used in the scripts. (ZIP) [file pbio.3003066.s025.zip › Custom Code/notebooks/Fig3_and_S2_and_S3.html]

Fig3\_and\_S2\_and\_S3


In [1]:

```
cd ../../Papers/Cocci_transcriptomics/data_for_code/Fig2/Combined
```

```
/home/chomer/Papers/Cocci_transcriptomics/data_for_code/Fig2/Combined
```

In [2]:

```
from CdtFile import CdtFile, CdtRow
from CdtAnnotator3 import annotate_CpSilveira as annotate
from LimmaTools import SingleFactorFit
from MsvUtil import Table
from csv import writer, excel_tab
from glob import glob
from math import log, atan2, pi
import os.path
import re
from SafeMath import safelog
from PCA import PCA
import matplotlib.pyplot as plt
%matplotlib nbagg
%load_ext rpy2.ipython
from ReadCountTools import PseudoCPMs
from matplotlib_venn import venn3, venn3_circles
import statistics
```

In [3]:

```
%%R
library(limma)
library(edgeR)
```

# Merge Kallisto TPMs - combined spherule and hyphae Fig 3¶

In [4]:

```
#First Index

sname2kallisto = dict(
    ("_".join(i.replace(".Silv_nanopore_mRNA.rf.kallisto","").replace("../", "").split("_")[0:-3]),i)
              for i in glob("../"+"*.Silv_nanopore_mRNA.rf.kallisto") )

snames = sorted(sname2kallisto, key = lambda x: (x[0:x.rfind("_")],x[x.rfind("_"):]), reverse=False)

print(len(sname2kallisto))
print(snames)
```

```
84
['D1_myc_Ryp1_1', 'D1_myc_Ryp1_2', 'D1_myc_Ryp1_3', 'D1_myc_Sil_1', 'D1_myc_Sil_2', 'D1_myc_Sil_3', 'D1_spherule_Ryp1_1', 'D1_spherule_Ryp1_2', 'D1_spherule_Ryp1_3', 'D1_spherule_Sil_1', 'D1_spherule_Sil_2', 'D1_spherule_Sil_3', 'D2_myc_Ryp1_1', 'D2_myc_Ryp1_2', 'D2_myc_Ryp1_3', 'D2_myc_Sil_1', 'D2_myc_Sil_2', 'D2_myc_Sil_3', 'D2_spherule_Ryp1_1', 'D2_spherule_Ryp1_2', 'D2_spherule_Ryp1_3', 'D2_spherule_Sil_1', 'D2_spherule_Sil_2', 'D2_spherule_Sil_3', 'D3_myc_Ryp1_1', 'D3_myc_Ryp1_2', 'D3_myc_Ryp1_3', 'D3_myc_Sil_1', 'D3_myc_Sil_2', 'D3_myc_Sil_3', 'D3_spherules_Ryp1_1', 'D3_spherules_Ryp1_2', 'D3_spherules_Ryp1_3', 'D3_spherules_Sil_1', 'D3_spherules_Sil_2', 'D3_spherules_Sil_3', 'D4_spherules_Ryp1_1', 'D4_spherules_Ryp1_2', 'D4_spherules_Ryp1_3', 'D4_spherules_Sil_1', 'D4_spherules_Sil_2', 'D4_spherules_Sil_3', 'D5_spherules_Ryp1_1', 'D5_spherules_Ryp1_2', 'D5_spherules_Ryp1_3', 'D5_spherules_Sil_1', 'D5_spherules_Sil_2', 'D5_spherules_Sil_3', 'D6_myc_Ryp1_1', 'D6_myc_Ryp1_2', 'D6_myc_Ryp1_3', 'D6_myc_Sil_1', 'D6_myc_Sil_2', 'D6_myc_Sil_3', 'D6_spherules_Ryp1_1', 'D6_spherules_Ryp1_2', 'D6_spherules_Ryp1_3', 'D6_spherules_Sil_1', 'D6_spherules_Sil_2', 'D6_spherules_Sil_3', 'DMEM_1', 'DMEM_2', 'DMEM_3', 'Eighth_myc_Ryp1_1', 'Eighth_myc_Ryp1_2', 'Eighth_myc_Ryp1_3', 'Eighth_myc_Sil_1', 'Eighth_myc_Sil_2', 'Eighth_myc_Sil_3', 'Eighth_spherule_Sil_1', 'Eighth_spherule_Sil_2', 'Eighth_spherule_Sil_3', 'Eighth_spherule_ryp1_1', 'Eighth_spherule_ryp1_2', 'Eighth_spherule_ryp1_3', 'RPMI_1', 'RPMI_2', 'RPMI_3', 'Spores_Ryp1_1', 'Spores_Ryp1_2', 'Spores_Ryp1_3', 'Spores_Sil_1', 'Spores_Sil_2', 'Spores_Sil_3']
```

In [5]:

```
#Merge Step
genes = None
cols = []
counts = []
for i in snames:
    table = Table.fromTdt(open(os.path.join(
           sname2kallisto[i],
          "abundance.tsv")))
    if(genes is None):
        genes = table["target_id"]
    else:
        assert(genes == table["target_id"])
    cols.append([float(i) for i in table["tpm"]])
    counts.append([int(float(i)+.5) for i in table["est_counts"]])

tpm_trans = CdtFile(probes = [CdtRow(gid = i[0], uniqid = i[0], name = i[0],
                                        ratios = [safelog(j) for j in i[1:]])
                                 for i in zip(*([genes]+cols))],
                       fieldnames = snames,
                       eweights = [1]*len(snames))
tpm_trans.write(open("tpm_trans.cdt","w"))

trans_counts = CdtFile(probes = [CdtRow(gid = i[0], uniqid = i[0], name = i[0],
                                        ratios = i[1:])
                                 for i in zip(*([genes]+counts))],
                       fieldnames = snames,
                       eweights = [1]*len(snames))
trans_counts.write(open("trans_counts.cdt","w"))

len(tpm_trans), len(trans_counts)
```

Out[5]:

```
(8628, 8628)
```

In [6]:

```
merge_pseudo = PseudoCPMs.fromCounts(trans_counts)
```

In [7]:

```
mask_10 = merge_pseudo.depth_filter_mask(10,.07)
mask_10.counts.write(open("Fig3_counts_pseudoCPMs.for_paper.cdt", "w"))
```

In [8]:

```
ct_counts = CdtFile.fromCdt("Fig3_counts_pseudoCPMs.for_paper.cdt")
len(ct_counts)
```

Out[8]:

```
8186
```

## Limma analysis¶

In [9]:

```
#need to format ct_tpm for limma input
fout = open("ct_counts.txt", "w")

fout.write("\t".join(["gene"]+ct_counts.fieldnames)+"\n")
for row in mask_10.counts:
    fout.write("\t".join([row.Uniqid()]+[str(i) for i in row])+"\n")
fout.close()
```

In [10]:

```
%%R
#Limma Single Factor Fit
# Read the count matrix, using the gene column as row names
C <- read.delim("ct_counts.txt",row.names=1)
#Convert the matrix to limma's preferred format, implicitly log2 transforming and depth normalizing to CPM values
dge <- DGEList(counts=C)
```

In [11]:

```
out = writer(open("Fig3_simple_comp_samples.txt","w"),dialect = excel_tab)
out.writerow(("run","state"))
for i in snames:
    run = i
    state = "_".join(i.split("_")[0:-1])
    out.writerow((run, state))   
del out
```

In [12]:

```
%%R -o d
samples <- read.delim("Fig3_simple_comp_samples.txt")
print(summary(samples))
state <- samples$state
d <- model.matrix(~0+state)
colnames(d) <- gsub("state","",colnames(d))
print(colnames(d))
```

```
     run               state          
 Length:84          Length:84         
 Class :character   Class :character  
 Mode  :character   Mode  :character  
 [1] "D1_myc_Ryp1"          "D1_myc_Sil"           "D1_spherule_Ryp1"    
 [4] "D1_spherule_Sil"      "D2_myc_Ryp1"          "D2_myc_Sil"          
 [7] "D2_spherule_Ryp1"     "D2_spherule_Sil"      "D3_myc_Ryp1"         
[10] "D3_myc_Sil"           "D3_spherules_Ryp1"    "D3_spherules_Sil"    
[13] "D4_spherules_Ryp1"    "D4_spherules_Sil"     "D5_spherules_Ryp1"   
[16] "D5_spherules_Sil"     "D6_myc_Ryp1"          "D6_myc_Sil"          
[19] "D6_spherules_Ryp1"    "D6_spherules_Sil"     "DMEM"                
[22] "Eighth_myc_Ryp1"      "Eighth_myc_Sil"       "Eighth_spherule_ryp1"
[25] "Eighth_spherule_Sil"  "RPMI"                 "Spores_Ryp1"         
[28] "Spores_Sil"
```

In [13]:

```
%%R
# Apply between-sample TMM normalization
dge <- calcNormFactors(dge)
# Estimate the mean-variance trend via locally-linear regression and use this trend
# to assign weights to the observations (counts)
v <- voom(dge, d, plot = TRUE)
cpm <- v$E
```

In [14]:

```
%%R -o cpm,fc,cn,state
# Fit the model (classic linear regression)
fit <- lmFit(v, d)
#Generate the contrast matrix
contrast.matrix <- makeContrasts(
    Eighth_spherule_Sil - Spores_Sil, D1_spherule_Sil - Spores_Sil, D2_spherule_Sil - Spores_Sil, D3_spherules_Sil - Spores_Sil, D4_spherules_Sil - Spores_Sil, D5_spherules_Sil - Spores_Sil, D6_spherules_Sil - Spores_Sil,
    Eighth_spherule_ryp1 - Spores_Ryp1, D1_spherule_Ryp1 - Spores_Ryp1, D2_spherule_Ryp1 - Spores_Ryp1, D3_spherules_Ryp1 - Spores_Ryp1, D4_spherules_Ryp1 - Spores_Ryp1, D5_spherules_Ryp1 - Spores_Ryp1, D6_spherules_Ryp1 - Spores_Ryp1,
    Spores_Sil - Spores_Ryp1,
    Eighth_spherule_Sil - Eighth_spherule_ryp1, D1_spherule_Sil - D1_spherule_Ryp1, D2_spherule_Sil - D2_spherule_Ryp1, D3_spherules_Sil - D3_spherules_Ryp1, D4_spherules_Sil - D4_spherules_Ryp1, D5_spherules_Sil - D5_spherules_Ryp1, D6_spherules_Sil - D6_spherules_Ryp1,
    D1_spherule_Sil - Eighth_spherule_Sil, D2_spherule_Sil - D1_spherule_Sil, D3_spherules_Sil - D2_spherule_Sil, D4_spherules_Sil - D3_spherules_Sil, D5_spherules_Sil - D4_spherules_Sil, D6_spherules_Sil - D5_spherules_Sil,
    Eighth_myc_Sil - Spores_Sil, D1_myc_Sil - Spores_Sil, D2_myc_Sil - Spores_Sil, D3_myc_Sil - Spores_Sil, D6_myc_Sil - Spores_Sil,
    Eighth_myc_Ryp1 - Spores_Ryp1, D1_myc_Ryp1 - Spores_Ryp1, D2_myc_Ryp1 - Spores_Ryp1, D3_myc_Ryp1 - Spores_Ryp1, D6_myc_Ryp1 - Spores_Ryp1,
    Spores_Sil - Spores_Ryp1,
    Eighth_myc_Sil - Eighth_myc_Ryp1, D1_myc_Sil - D1_myc_Ryp1, D2_myc_Sil - D2_myc_Ryp1, D3_myc_Sil - D3_myc_Ryp1, D6_myc_Sil - D6_myc_Ryp1,
    D1_myc_Sil - Eighth_myc_Sil, D2_myc_Sil - D1_myc_Sil, D3_myc_Sil - D2_myc_Sil, D6_myc_Sil - D3_myc_Sil,
    Eighth_spherule_Sil - Eighth_myc_Sil, D1_spherule_Sil - D1_myc_Sil, D2_spherule_Sil - D2_myc_Sil, D3_spherules_Sil - D3_myc_Sil, D6_spherules_Sil - D6_myc_Sil,
    Eighth_spherule_ryp1 - Eighth_myc_Ryp1, D1_spherule_Ryp1 - D1_myc_Ryp1, D2_spherule_Ryp1 - D2_myc_Ryp1, D3_spherules_Ryp1 - D3_myc_Ryp1, D6_spherules_Ryp1 - D6_myc_Ryp1,

    levels=d)
# Apply the contrast matrix
fit2 <- contrasts.fit(fit, contrast.matrix)
# Apply Empirical Bayes "shrinkage"
fit2 <- eBayes(fit2)
# Simple summary of significantly differential genes with no fold change filter
print(summary(decideTests(fit2)))

fc <- fit$coefficients
cn <- colnames(fit$coefficients)
cpm <- v$E
```

```
       Eighth_spherule_Sil - Spores_Sil D1_spherule_Sil - Spores_Sil
Down                               2627                         2798
NotSig                             2585                         2395
Up                                 2974                         2993
       D2_spherule_Sil - Spores_Sil D3_spherules_Sil - Spores_Sil
Down                           2727                          2653
NotSig                         2485                          2462
Up                             2974                          3071
       D4_spherules_Sil - Spores_Sil D5_spherules_Sil - Spores_Sil
Down                            2568                          2563
NotSig                          2736                          2690
Up                              2882                          2933
       D6_spherules_Sil - Spores_Sil Eighth_spherule_ryp1 - Spores_Ryp1
Down                            2504                               2396
NotSig                          2866                               3549
Up                              2816                               2241
       D1_spherule_Ryp1 - Spores_Ryp1 D2_spherule_Ryp1 - Spores_Ryp1
Down                             2715                           2842
NotSig                           2892                           2741
Up                               2579                           2603
       D3_spherules_Ryp1 - Spores_Ryp1 D4_spherules_Ryp1 - Spores_Ryp1
Down                              2748                            2749
NotSig                            2902                            2873
Up                                2536                            2564
       D5_spherules_Ryp1 - Spores_Ryp1 D6_spherules_Ryp1 - Spores_Ryp1
Down                              2559                            2473
NotSig                            3118                            3315
Up                                2509                            2398
       Spores_Sil - Spores_Ryp1 Eighth_spherule_Sil - Eighth_spherule_ryp1
Down                       2639                                       1740
NotSig                     3229                                       4558
Up                         2318                                       1888
       D1_spherule_Sil - D1_spherule_Ryp1 D2_spherule_Sil - D2_spherule_Ryp1
Down                                 2022                               2006
NotSig                               4228                               4229
Up                                   1936                               1951
       D3_spherules_Sil - D3_spherules_Ryp1
Down                                   2145
NotSig                                 3691
Up                                     2350
       D4_spherules_Sil - D4_spherules_Ryp1
Down                                   2301
NotSig                                 3249
Up                                     2636
       D5_spherules_Sil - D5_spherules_Ryp1
Down                                   1833
NotSig                                 4544
Up                                     1809
       D6_spherules_Sil - D6_spherules_Ryp1
Down                                   1873
NotSig                                 4321
Up                                     1992
       D1_spherule_Sil - Eighth_spherule_Sil D2_spherule_Sil - D1_spherule_Sil
Down                                    1663                              1587
NotSig                                  5093                              5370
Up                                      1430                              1229
       D3_spherules_Sil - D2_spherule_Sil D4_spherules_Sil - D3_spherules_Sil
Down                                 1126                                 735
NotSig                               5793                                6662
Up                                   1267                                 789
       D5_spherules_Sil - D4_spherules_Sil D6_spherules_Sil - D5_spherules_Sil
Down                                   134                                  55
NotSig                                7911                                8007
Up                                     141                                 124
       Eighth_myc_Sil - Spores_Sil D1_myc_Sil - Spores_Sil
Down                          2574                    2754
NotSig                        3029                    2528
Up                            2583                    2904
       D2_myc_Sil - Spores_Sil D3_myc_Sil - Spores_Sil D6_myc_Sil - Spores_Sil
Down                      2704                    2629                    2594
NotSig                    2533                    2482                    2735
Up                        2949                    3075                    2857
       Eighth_myc_Ryp1 - Spores_Ryp1 D1_myc_Ryp1 - Spores_Ryp1
Down                            2439                      2554
NotSig                          3557                      3265
Up                              2190                      2367
       D2_myc_Ryp1 - Spores_Ryp1 D3_myc_Ryp1 - Spores_Ryp1
Down                        2830                      2713
NotSig                      2760                      2911
Up                          2596                      2562
       D6_myc_Ryp1 - Spores_Ryp1 Spores_Sil - Spores_Ryp1
Down                        2697                     2639
NotSig                      2927                     3229
Up                          2562                     2318
       Eighth_myc_Sil - Eighth_myc_Ryp1 D1_myc_Sil - D1_myc_Ryp1
Down                               2136                     1984
NotSig                             4032                     4171
Up                                 2018                     2031
       D2_myc_Sil - D2_myc_Ryp1 D3_myc_Sil - D3_myc_Ryp1
Down                       1927                     1284
NotSig                     4675                     5454
Up                         1584                     1448
       D6_myc_Sil - D6_myc_Ryp1 D1_myc_Sil - Eighth_myc_Sil
Down                       1043                        1445
NotSig                     6251                        5011
Up                          892                        1730
       D2_myc_Sil - D1_myc_Sil D3_myc_Sil - D2_myc_Sil D6_myc_Sil - D3_myc_Sil
Down                      2087                     287                      70
NotSig                    4480                    7582                    8005
Up                        1619                     317                     111
       Eighth_spherule_Sil - Eighth_myc_Sil D1_spherule_Sil - D1_myc_Sil
Down                                   1344                         1941
NotSig                                 5111                         4498
Up                                     1731                         1747
       D2_spherule_Sil - D2_myc_Sil D3_spherules_Sil - D3_myc_Sil
Down                           1864                          2124
NotSig                         4536                          3863
Up                             1786                          2199
       D6_spherules_Sil - D6_myc_Sil Eighth_spherule_ryp1 - Eighth_myc_Ryp1
Down                            2168                                   1154
NotSig                          3434                                   5841
Up                              2584                                   1191
       D1_spherule_Ryp1 - D1_myc_Ryp1 D2_spherule_Ryp1 - D2_myc_Ryp1
Down                             1685                           1836
NotSig                           4817                           4789
Up                               1684                           1561
       D3_spherules_Ryp1 - D3_myc_Ryp1 D6_spherules_Ryp1 - D6_myc_Ryp1
Down                              1835                            2087
NotSig                            4435                            3823
Up                                1916                            2276
```

In [15]:

```
%%R
print(summary(decideTests(fit2,lfc=1)))
#Used for Fig S2C and Fig S2E
```

```
       Eighth_spherule_Sil - Spores_Sil D1_spherule_Sil - Spores_Sil
Down                               2008                         2153
NotSig                             3925                         3710
Up                                 2253                         2323
       D2_spherule_Sil - Spores_Sil D3_spherules_Sil - Spores_Sil
Down                           2145                          1926
NotSig                         3624                          3853
Up                             2417                          2407
       D4_spherules_Sil - Spores_Sil D5_spherules_Sil - Spores_Sil
Down                            1716                          1746
NotSig                          4261                          4215
Up                              2209                          2225
       D6_spherules_Sil - Spores_Sil Eighth_spherule_ryp1 - Spores_Ryp1
Down                            1644                               1684
NotSig                          4422                               5087
Up                              2120                               1415
       D1_spherule_Ryp1 - Spores_Ryp1 D2_spherule_Ryp1 - Spores_Ryp1
Down                             2022                           1988
NotSig                           4427                           4374
Up                               1737                           1824
       D3_spherules_Ryp1 - Spores_Ryp1 D4_spherules_Ryp1 - Spores_Ryp1
Down                              1905                            1849
NotSig                            4520                            4562
Up                                1761                            1775
       D5_spherules_Ryp1 - Spores_Ryp1 D6_spherules_Ryp1 - Spores_Ryp1
Down                              1635                            1570
NotSig                            4875                            5022
Up                                1676                            1594
       Spores_Sil - Spores_Ryp1 Eighth_spherule_Sil - Eighth_spherule_ryp1
Down                       2063                                       1098
NotSig                     4587                                       6192
Up                         1536                                        896
       D1_spherule_Sil - D1_spherule_Ryp1 D2_spherule_Sil - D2_spherule_Ryp1
Down                                 1101                               1223
NotSig                               6017                               5678
Up                                   1068                               1285
       D3_spherules_Sil - D3_spherules_Ryp1
Down                                   1152
NotSig                                 5658
Up                                     1376
       D4_spherules_Sil - D4_spherules_Ryp1
Down                                   1430
NotSig                                 5221
Up                                     1535
       D5_spherules_Sil - D5_spherules_Ryp1
Down                                   1025
NotSig                                 6292
Up                                      869
       D6_spherules_Sil - D6_spherules_Ryp1
Down                                   1062
NotSig                                 6180
Up                                      944
       D1_spherule_Sil - Eighth_spherule_Sil D2_spherule_Sil - D1_spherule_Sil
Down                                     634                               508
NotSig                                  6763                              6864
Up                                       789                               814
       D3_spherules_Sil - D2_spherule_Sil D4_spherules_Sil - D3_spherules_Sil
Down                                  560                                 247
NotSig                               6980                                7743
Up                                    646                                 196
       D5_spherules_Sil - D4_spherules_Sil D6_spherules_Sil - D5_spherules_Sil
Down                                    70                                  22
NotSig                                8058                                8118
Up                                      58                                  46
       Eighth_myc_Sil - Spores_Sil D1_myc_Sil - Spores_Sil
Down                          1908                    2044
NotSig                        4377                    3886
Up                            1901                    2256
       D2_myc_Sil - Spores_Sil D3_myc_Sil - Spores_Sil D6_myc_Sil - Spores_Sil
Down                      1997                    1953                    1972
NotSig                    3904                    3859                    3983
Up                        2285                    2374                    2231
       Eighth_myc_Ryp1 - Spores_Ryp1 D1_myc_Ryp1 - Spores_Ryp1
Down                            1743                      1836
NotSig                          5053                      4795
Up                              1390                      1555
       D2_myc_Ryp1 - Spores_Ryp1 D3_myc_Ryp1 - Spores_Ryp1
Down                        2080                      1865
NotSig                      4239                      4508
Up                          1867                      1813
       D6_myc_Ryp1 - Spores_Ryp1 Spores_Sil - Spores_Ryp1
Down                        1830                     2063
NotSig                      4613                     4587
Up                          1743                     1536
       Eighth_myc_Sil - Eighth_myc_Ryp1 D1_myc_Sil - D1_myc_Ryp1
Down                               1442                     1145
NotSig                             5599                     5872
Up                                 1145                     1169
       D2_myc_Sil - D2_myc_Ryp1 D3_myc_Sil - D3_myc_Ryp1
Down                        782                      777
NotSig                     6360                     6782
Up                         1044                      627
       D6_myc_Sil - D6_myc_Ryp1 D1_myc_Sil - Eighth_myc_Sil
Down                        426                         580
NotSig                     7215                        6535
Up                          545                        1071
       D2_myc_Sil - D1_myc_Sil D3_myc_Sil - D2_myc_Sil D6_myc_Sil - D3_myc_Sil
Down                       651                     154                      24
NotSig                    6512                    7863                    8078
Up                        1023                     169                      84
       Eighth_spherule_Sil - Eighth_myc_Sil D1_spherule_Sil - D1_myc_Sil
Down                                    543                         1018
NotSig                                 6659                         6161
Up                                      984                         1007
       D2_spherule_Sil - D2_myc_Sil D3_spherules_Sil - D3_myc_Sil
Down                           1143                          1103
NotSig                         5923                          5860
Up                             1120                          1223
       D6_spherules_Sil - D6_myc_Sil Eighth_spherule_ryp1 - Eighth_myc_Ryp1
Down                            1404                                    460
NotSig                          5314                                   7026
Up                              1468                                    700
       D1_spherule_Ryp1 - D1_myc_Ryp1 D2_spherule_Ryp1 - D2_myc_Ryp1
Down                              819                            845
NotSig                           6511                           6377
Up                                856                            964
       D3_spherules_Ryp1 - D3_myc_Ryp1 D6_spherules_Ryp1 - D6_myc_Ryp1
Down                              1126                            1106
NotSig                            6118                            5848
Up                                 942                            1232
```

In [16]:

```
name2row = dict((i.Uniqid(),n+1) for (n,i) in enumerate(ct_counts))
```

In [17]:

```
name2row = dict((i.Uniqid(),n+1) for (n,i) in enumerate(ct_counts))
fit = SingleFactorFit(fc, cpm, name2row, cn, 
                      state, obs_samples = ct_counts.fieldnames, 
                      parameter_order = ("Spores_Sil","Eighth_spherule_Sil","D1_spherule_Sil", "D2_spherule_Sil", "D3_spherules_Sil", "D4_spherules_Sil", "D5_spherules_Sil", "D6_spherules_Sil", "Spores_Ryp1","Eighth_spherule_ryp1","D1_spherule_Ryp1", "D2_spherule_Ryp1", "D3_spherules_Ryp1", "D4_spherules_Ryp1", "D5_spherules_Ryp1", "D6_spherules_Ryp1","DMEM", "RPMI", "Eighth_myc_Sil", "D1_myc_Sil","D2_myc_Sil","D3_myc_Sil","D6_myc_Sil", "Eighth_myc_Ryp1", "D1_myc_Ryp1","D2_myc_Ryp1","D3_myc_Ryp1","D6_myc_Ryp1"))
fit.toHDF5("Fig3_Singlecomp_limma1.hdf5")
fit2 = SingleFactorFit.fromHDF5("Fig3_Singlecomp_limma1.hdf5")
```

In [18]:

```
ct_counts.mean_normalize_rows().bicluster("ct_tpm.norm.um",dist="u",method="m")
```

```
Building array...
Building distance matrix...
Clustering...
```

In [19]:

```
%%R
write.csv(cpm,"limma1.countscpm.cpm.csv")
```

In [20]:

```
%%R
for(tc in colnames(fit2$coefficients)){
  print(tc)
  # Extract all genes significantly differential on this contrast for a 2x fold change cutoff and 5% FDR
  # Use write.csv rather than write.table for clean compatibility with python's csv.reader
  write.csv(topTable(fit2, coef=tc, n = 50000, lfc=1, p.value = .05),
            paste("limma1.",gsub(" ","",tc),".t0.csv",sep=""))
  # Extract the adjusted p-values for this contrast for all genes, independent of significance
  write.csv(topTable(fit2, coef=tc, n = 50000),
            paste("limma1.",gsub(" ","",tc),".t1.csv",sep=""))
}
```

```
[1] "Eighth_spherule_Sil - Spores_Sil"
[1] "D1_spherule_Sil - Spores_Sil"
[1] "D2_spherule_Sil - Spores_Sil"
[1] "D3_spherules_Sil - Spores_Sil"
[1] "D4_spherules_Sil - Spores_Sil"
[1] "D5_spherules_Sil - Spores_Sil"
[1] "D6_spherules_Sil - Spores_Sil"
[1] "Eighth_spherule_ryp1 - Spores_Ryp1"
[1] "D1_spherule_Ryp1 - Spores_Ryp1"
[1] "D2_spherule_Ryp1 - Spores_Ryp1"
[1] "D3_spherules_Ryp1 - Spores_Ryp1"
[1] "D4_spherules_Ryp1 - Spores_Ryp1"
[1] "D5_spherules_Ryp1 - Spores_Ryp1"
[1] "D6_spherules_Ryp1 - Spores_Ryp1"
[1] "Spores_Sil - Spores_Ryp1"
[1] "Eighth_spherule_Sil - Eighth_spherule_ryp1"
[1] "D1_spherule_Sil - D1_spherule_Ryp1"
[1] "D2_spherule_Sil - D2_spherule_Ryp1"
[1] "D3_spherules_Sil - D3_spherules_Ryp1"
[1] "D4_spherules_Sil - D4_spherules_Ryp1"
[1] "D5_spherules_Sil - D5_spherules_Ryp1"
[1] "D6_spherules_Sil - D6_spherules_Ryp1"
[1] "D1_spherule_Sil - Eighth_spherule_Sil"
[1] "D2_spherule_Sil - D1_spherule_Sil"
[1] "D3_spherules_Sil - D2_spherule_Sil"
[1] "D4_spherules_Sil - D3_spherules_Sil"
[1] "D5_spherules_Sil - D4_spherules_Sil"
[1] "D6_spherules_Sil - D5_spherules_Sil"
[1] "Eighth_myc_Sil - Spores_Sil"
[1] "D1_myc_Sil - Spores_Sil"
[1] "D2_myc_Sil - Spores_Sil"
[1] "D3_myc_Sil - Spores_Sil"
[1] "D6_myc_Sil - Spores_Sil"
[1] "Eighth_myc_Ryp1 - Spores_Ryp1"
[1] "D1_myc_Ryp1 - Spores_Ryp1"
[1] "D2_myc_Ryp1 - Spores_Ryp1"
[1] "D3_myc_Ryp1 - Spores_Ryp1"
[1] "D6_myc_Ryp1 - Spores_Ryp1"
[1] "Spores_Sil - Spores_Ryp1"
[1] "Eighth_myc_Sil - Eighth_myc_Ryp1"
[1] "D1_myc_Sil - D1_myc_Ryp1"
[1] "D2_myc_Sil - D2_myc_Ryp1"
[1] "D3_myc_Sil - D3_myc_Ryp1"
[1] "D6_myc_Sil - D6_myc_Ryp1"
[1] "D1_myc_Sil - Eighth_myc_Sil"
[1] "D2_myc_Sil - D1_myc_Sil"
[1] "D3_myc_Sil - D2_myc_Sil"
[1] "D6_myc_Sil - D3_myc_Sil"
[1] "Eighth_spherule_Sil - Eighth_myc_Sil"
[1] "D1_spherule_Sil - D1_myc_Sil"
[1] "D2_spherule_Sil - D2_myc_Sil"
[1] "D3_spherules_Sil - D3_myc_Sil"
[1] "D6_spherules_Sil - D6_myc_Sil"
[1] "Eighth_spherule_ryp1 - Eighth_myc_Ryp1"
[1] "D1_spherule_Ryp1 - D1_myc_Ryp1"
[1] "D2_spherule_Ryp1 - D2_myc_Ryp1"
[1] "D3_spherules_Ryp1 - D3_myc_Ryp1"
[1] "D6_spherules_Ryp1 - D6_myc_Ryp1"
```

In [21]:

```
gene2cpms = dict((i[0],[float(j) for j in i[1:]]) for i in Table.fromCsv("limma1.countscpm.cpm.csv"))
len(gene2cpms), len(ct_counts)
```

Out[21]:

```
(8186, 8186)
```

In [22]:

```
limma1_cdt = CdtFile.fromPrototype(ct_counts, 
                                   probes = [CdtRow.fromPrototype(i, ratios = gene2cpms[i.Uniqid()][:])
                                             for i in ct_counts])
limma1_cdt = limma1_cdt.mean_normalize_rows()
```

In [23]:

```
gene2contrasts = dict((i.Uniqid(),[]) for i in limma1_cdt)
gene2pvals = dict((i.Uniqid(),[]) for i in limma1_cdt)
gene2sigs = dict((i.Uniqid(),[]) for i in limma1_cdt)
contrast_names = []

contrast_csvs = sorted(glob("limma1.*.t1.csv"))
# put 8 hour first
contrast_csvs = contrast_csvs[-1:]+contrast_csvs[:-1]
for i in contrast_csvs:
    cname = i.replace("limma1.","").replace(".t1.csv","").replace("-","/")
    contrast_names.append(cname)
    siglist = set(i[0] for i in Table.fromCsv(i.replace(".t1.",".t0.")))
    print(cname,len(siglist))
    for gene in Table.fromCsv(i):
        name = gene[0]
        lfc = float(gene["logFC"])
        gene2contrasts[name].append(lfc)
        gene2pvals[name].append(gene["adj.P.Val"])
        if(name in siglist):
            if(lfc > 0):
                gene2sigs[name].append(4.)
            else:
                gene2sigs[name].append(-4.)
        else:
            gene2sigs[name].append(0.)
        
limma1_cdt = CdtFile.fromPrototype(limma1_cdt,
    probes = [CdtRow.fromPrototype(i, ratios = i.ratios+gene2contrasts[i.Uniqid()]+gene2sigs[i.Uniqid()],
                                   extra = i.extra+gene2pvals[i.Uniqid()])
              for i in limma1_cdt],
    fieldnames = limma1_cdt.fieldnames+contrast_names+["%s_sig" % i for i in contrast_names],
    eweights = limma1_cdt.eweights+[1.]*2*len(contrast_names),
    extranames = limma1_cdt.extranames+["p(%s)" % i for i in contrast_names])
```

```
Spores_Sil/Spores_Ryp1 3599
D1_myc_Ryp1/Spores_Ryp1 3391
D1_myc_Sil/D1_myc_Ryp1 2314
D1_myc_Sil/Eighth_myc_Sil 1651
D1_myc_Sil/Spores_Sil 4300
D1_spherule_Ryp1/D1_myc_Ryp1 1675
D1_spherule_Ryp1/Spores_Ryp1 3759
D1_spherule_Sil/D1_myc_Sil 2025
D1_spherule_Sil/D1_spherule_Ryp1 2169
D1_spherule_Sil/Eighth_spherule_Sil 1423
D1_spherule_Sil/Spores_Sil 4476
D2_myc_Ryp1/Spores_Ryp1 3947
D2_myc_Sil/D1_myc_Sil 1674
D2_myc_Sil/D2_myc_Ryp1 1826
D2_myc_Sil/Spores_Sil 4282
D2_spherule_Ryp1/D2_myc_Ryp1 1809
D2_spherule_Ryp1/Spores_Ryp1 3812
D2_spherule_Sil/D1_spherule_Sil 1322
D2_spherule_Sil/D2_myc_Sil 2263
D2_spherule_Sil/D2_spherule_Ryp1 2508
D2_spherule_Sil/Spores_Sil 4562
D3_myc_Ryp1/Spores_Ryp1 3678
D3_myc_Sil/D2_myc_Sil 323
D3_myc_Sil/D3_myc_Ryp1 1404
D3_myc_Sil/Spores_Sil 4327
D3_spherules_Ryp1/D3_myc_Ryp1 2068
D3_spherules_Ryp1/Spores_Ryp1 3666
D3_spherules_Sil/D2_spherule_Sil 1206
D3_spherules_Sil/D3_myc_Sil 2326
D3_spherules_Sil/D3_spherules_Ryp1 2528
D3_spherules_Sil/Spores_Sil 4333
D4_spherules_Ryp1/Spores_Ryp1 3624
D4_spherules_Sil/D3_spherules_Sil 443
D4_spherules_Sil/D4_spherules_Ryp1 2965
D4_spherules_Sil/Spores_Sil 3925
D5_spherules_Ryp1/Spores_Ryp1 3311
D5_spherules_Sil/D4_spherules_Sil 128
D5_spherules_Sil/D5_spherules_Ryp1 1894
D5_spherules_Sil/Spores_Sil 3971
D6_myc_Ryp1/Spores_Ryp1 3573
D6_myc_Sil/D3_myc_Sil 108
D6_myc_Sil/D6_myc_Ryp1 971
D6_myc_Sil/Spores_Sil 4203
D6_spherules_Ryp1/D6_myc_Ryp1 2338
D6_spherules_Ryp1/Spores_Ryp1 3164
D6_spherules_Sil/D5_spherules_Sil 68
D6_spherules_Sil/D6_myc_Sil 2872
D6_spherules_Sil/D6_spherules_Ryp1 2006
D6_spherules_Sil/Spores_Sil 3764
Eighth_myc_Ryp1/Spores_Ryp1 3133
Eighth_myc_Sil/Eighth_myc_Ryp1 2587
Eighth_myc_Sil/Spores_Sil 3809
Eighth_spherule_Sil/Eighth_myc_Sil 1527
Eighth_spherule_Sil/Eighth_spherule_ryp1 1994
Eighth_spherule_Sil/Spores_Sil 4261
Eighth_spherule_ryp1/Eighth_myc_Ryp1 1160
Eighth_spherule_ryp1/Spores_Ryp1 3099
```

In [24]:

```
limma1_cdt = CdtFile.fromPrototype(limma1_cdt, 
    probes = [CdtRow.fromPrototype(i, extra = i.extra + [str(j) for j in ct_counts.GetUid(i.Uniqid())])
              for i in limma1_cdt],
    extranames = limma1_cdt.extranames+["%s_counts" % i for i in ct_counts.fieldnames])
```

In [25]:

```
limma1_cdt.writeCdt("limma1.countscutoff.cdt")
```

In [26]:

```
sig_cols = [n for (n,i) in enumerate(limma1_cdt.fieldnames) if(i.endswith("_sig"))]
contrast_cols = [n-len(sig_cols) for n in sig_cols]
limma1_sig= CdtFile.fromPrototype(limma1_cdt, probes = [i for i in limma1_cdt 
                                                        if(any([(i[j] != 0.) for j in sig_cols]))])
tree = limma1_sig.cluster(cols=contrast_cols,dist="u",method="m")
limma1_sig.writeCdtGtr("limma1_sig.countscutoff.contrasts_um",tree)
len(limma1_sig)
```

```
Building array...
Building distance matrix...
Clustering...
```

Out[26]:

```
8044
```

# Note everything from here down is filtered on significant p-value with 2x fold-cutoff change for stated comparisons¶

In [27]:

```
#Looking at transcripts that are completely Ryp1-dependent D1-D6 in spherules and hyphae both
sig_cols_ryp = [] 
for n,i in (enumerate(limma1_sig.fieldnames)):
    if i.endswith("_sig"):
        if "Sil" in i.split("/")[0] or "sil" in i.split("/")[0]:
            if "Ryp1" in i.split("/")[1] or "ryp1" in i.split("/")[1]:
                if "Spores" not in i:
                        if "Eight" not in i:
                            sig_cols_ryp.append(n)
                            print(i)
sig_cols = [n for (n,i) in enumerate(limma1_sig.fieldnames) if(i.endswith("_sig"))]
contrast_cols = [n-len(sig_cols) for n in sig_cols]
limma1_ryp1dep_all_minus_arth = CdtFile.fromPrototype(limma1_sig, probes = [i for i in limma1_sig 
                                                        if(all([(i[j] != 0.) for j in sig_cols_ryp]))])
tree = limma1_ryp1dep_all_minus_arth.cluster(cols=contrast_cols,dist="u",method="m")
limma1_ryp1dep_all_minus_arth.writeCdtGtr("limma1_ryp1dep_all.contrasts_um",tree)
len(limma1_ryp1dep_all_minus_arth)
```

```
D1_myc_Sil/D1_myc_Ryp1_sig
D1_spherule_Sil/D1_spherule_Ryp1_sig
D2_myc_Sil/D2_myc_Ryp1_sig
D2_spherule_Sil/D2_spherule_Ryp1_sig
D3_myc_Sil/D3_myc_Ryp1_sig
D3_spherules_Sil/D3_spherules_Ryp1_sig
D4_spherules_Sil/D4_spherules_Ryp1_sig
D5_spherules_Sil/D5_spherules_Ryp1_sig
D6_myc_Sil/D6_myc_Ryp1_sig
D6_spherules_Sil/D6_spherules_Ryp1_sig
```

```
Building array...
Building distance matrix...
Clustering...
```

Out[27]:

```
79
```

## Next, look at ryp1-regulated genes over spherulation or hyphal growth¶

In [28]:

```
#transcripts that are ryp1-dependent D1-D6 in spherules
sig_cols_ryp_spher = [] 
for n,i in (enumerate(limma1_sig.fieldnames)):
    if i.endswith("_sig"):
        if "Sil" in i.split("/")[0] or "sil" in i.split("/")[0]:
            if "Ryp1" in i.split("/")[1] or "ryp1" in i.split("/")[1]:
                if "spherule" in i and "Eight" not in i:
                    sig_cols_ryp_spher.append(n)
                    print(i)
sig_cols = [n for (n,i) in enumerate(limma1_sig.fieldnames) if(i.endswith("_sig"))]
contrast_cols = [n-len(sig_cols) for n in sig_cols]
limma1_ryp1dep_spherule = CdtFile.fromPrototype(limma1_sig, probes = [i for i in limma1_sig 
                                                        if(all([(i[j] != 0.) for j in sig_cols_ryp_spher]))])
tree = limma1_ryp1dep_spherule.cluster(cols=contrast_cols,dist="u",method="m")
limma1_ryp1dep_spherule.writeCdtGtr("limma1_ryp1dep_spherule.contrasts_um",tree)
len(limma1_ryp1dep_spherule)
```

```
D1_spherule_Sil/D1_spherule_Ryp1_sig
D2_spherule_Sil/D2_spherule_Ryp1_sig
D3_spherules_Sil/D3_spherules_Ryp1_sig
D4_spherules_Sil/D4_spherules_Ryp1_sig
D5_spherules_Sil/D5_spherules_Ryp1_sig
D6_spherules_Sil/D6_spherules_Ryp1_sig
```

```
Building array...
Building distance matrix...
Clustering...
```

Out[28]:

```
452
```

In [29]:

```
#which of these spherule-ryp1-regulated genes have spherule-associated expression?
sig_cols_ryp_spher = [] 
for n,i in (enumerate(limma1_sig.fieldnames)):
    if i.endswith("_sig"):
        if "Sil" in i.split("/")[0] or "sil" in i.split("/")[0]:
            if "Ryp1" in i.split("/")[1] or "ryp1" in i.split("/")[1]:
                if "spherule" in i and "Eight" not in i:
                    sig_cols_ryp_spher.append(n)
                    print(i)

   
sig_cols_morph_wt = [] 
for n,i in (enumerate(limma1_sig.fieldnames)):
    #print(i)
    if i.endswith("_sig") and 'ight' not in i:
        comp = i.split("/")
        #print(comp)
        if "Sil" in comp[0] or "sil" in comp[0]:
            if "Ryp" not in comp[1] and "ryp" not in comp[1] and "Spores" not in comp[1]:
                if "pher" in comp[0]:
                    if "pher" not in comp[1]:
                        print(i)
                        sig_cols_morph_wt.append(n)
                
sig_cols = [n for (n,i) in enumerate(limma1_sig.fieldnames) if(i.endswith("_sig"))]
contrast_cols = [n-len(sig_cols) for n in sig_cols]
limma1_ryp1dep_spherule_spherassoc = CdtFile.fromPrototype(limma1_sig, probes = [i for i in limma1_sig 
                                                        if(all([(i[j] != 0.) for j in sig_cols_ryp_spher]) and 
                                                              all([(i[k] > 0.) for k in sig_cols_morph_wt]))])
tree = limma1_ryp1dep_spherule_spherassoc.cluster(cols=contrast_cols,dist="u",method="m")
limma1_ryp1dep_spherule_spherassoc.writeCdtGtr("limma1_ryp1dep_spherule_spherassoc.contrasts_um",tree)
len(limma1_ryp1dep_spherule_spherassoc)
```

```
D1_spherule_Sil/D1_spherule_Ryp1_sig
D2_spherule_Sil/D2_spherule_Ryp1_sig
D3_spherules_Sil/D3_spherules_Ryp1_sig
D4_spherules_Sil/D4_spherules_Ryp1_sig
D5_spherules_Sil/D5_spherules_Ryp1_sig
D6_spherules_Sil/D6_spherules_Ryp1_sig
D1_spherule_Sil/D1_myc_Sil_sig
D2_spherule_Sil/D2_myc_Sil_sig
D3_spherules_Sil/D3_myc_Sil_sig
D6_spherules_Sil/D6_myc_Sil_sig
```

```
Building array...
Building distance matrix...
Clustering...
```

Out[29]:

```
82
```

In [30]:

```
#transcripts that are ryp1-dependent D1-D6 in hyphae

sig_cols_ryp_myc = [] 
for n,i in (enumerate(limma1_sig.fieldnames)):
    if i.endswith("_sig"):
        if "Sil" in i.split("/")[0] or "sil" in i.split("/")[0] :
            if "Ryp1" in i.split("/")[1] or "ryp1" in i.split("/")[1]:
                if "myc" in i and "Eight" not in i:
                    sig_cols_ryp_myc.append(n)
                    print(i)
sig_cols = [n for (n,i) in enumerate(limma1_sig.fieldnames) if(i.endswith("_sig"))]
contrast_cols = [n-len(sig_cols) for n in sig_cols]
limma1_ryp1dep_myc = CdtFile.fromPrototype(limma1_sig, probes = [i for i in limma1_sig
                                                        if(all([(i[j] != 0.) for j in sig_cols_ryp_myc]))])
tree = limma1_ryp1dep_myc.cluster(cols=contrast_cols,dist="u",method="m")
limma1_ryp1dep_myc.writeCdtGtr("limma1_ryp1dep_mycelia.contrasts_um",tree)
len(limma1_ryp1dep_myc)
```

```
D1_myc_Sil/D1_myc_Ryp1_sig
D2_myc_Sil/D2_myc_Ryp1_sig
D3_myc_Sil/D3_myc_Ryp1_sig
D6_myc_Sil/D6_myc_Ryp1_sig
```

```
Building array...
Building distance matrix...
Clustering...
```

Out[30]:

```
262
```

In [31]:

```
#which of these hyphal-ryp1-regulated genes have hyphal-associated expression?
sig_cols_ryp_myc = [] 
for n,i in (enumerate(limma1_sig.fieldnames)):
    if i.endswith("_sig"):
        if "Sil" in i.split("/")[0] or "sil" in i.split("/")[0] :
            if "Ryp1" in i.split("/")[1] or "ryp1" in i.split("/")[1]:
                if "myc" in i and "Eight" not in i:
                    sig_cols_ryp_myc.append(n)
                    print(i)

   
sig_cols_morph_wt = [] 
for n,i in (enumerate(limma1_sig.fieldnames)):
    #print(i)
    if i.endswith("_sig") and 'ight' not in i:
        comp = i.split("/")
        #print(comp)
        if "Sil" in comp[0] or "sil" in comp[0]:
            if "Ryp" not in comp[1] and "ryp" not in comp[1] and "Spores" not in comp[1]:
                if "pher" in comp[0]:
                    if "pher" not in comp[1]:
                        print(i)
                        sig_cols_morph_wt.append(n)
                
sig_cols = [n for (n,i) in enumerate(limma1_sig.fieldnames) if(i.endswith("_sig"))]
contrast_cols = [n-len(sig_cols) for n in sig_cols]
limma1_ryp1dep_spherule_spherassoc = CdtFile.fromPrototype(limma1_sig, probes = [i for i in limma1_sig 
                                                        if(all([(i[j] != 0.) for j in sig_cols_ryp_myc]) and 
                                                              all([(i[k] < 0.) for k in sig_cols_morph_wt]))])
tree = limma1_ryp1dep_spherule_spherassoc.cluster(cols=contrast_cols,dist="u",method="m")
limma1_ryp1dep_spherule_spherassoc.writeCdtGtr("limma1_ryp1dep_myc_mycassoc.contrasts_um",tree)
len(limma1_ryp1dep_spherule_spherassoc)
```

```
D1_myc_Sil/D1_myc_Ryp1_sig
D2_myc_Sil/D2_myc_Ryp1_sig
D3_myc_Sil/D3_myc_Ryp1_sig
D6_myc_Sil/D6_myc_Ryp1_sig
D1_spherule_Sil/D1_myc_Sil_sig
D2_spherule_Sil/D2_myc_Sil_sig
D3_spherules_Sil/D3_myc_Sil_sig
D6_spherules_Sil/D6_myc_Sil_sig
```

```
Building array...
Building distance matrix...
Clustering...
```

Out[31]:

```
32
```

## Now looking at morphology-dependent genes¶

In [32]:

```
# which transcripts are differential between spherule-inducing and hyphal-inducing conditions in WT and ryp1∆
sig_cols_morph_wt = [] 
sig_cols_morph_ryp1 = []
for n,i in (enumerate(limma1_sig.fieldnames)):
    if i.endswith("_sig") and "ight" not in i:
        comp = i.split("/")
        if "Sil" in comp[0] or "sil" in comp[0]:
            if "Ryp" not in comp[1] and "ryp" not in comp[1] and "Spores" not in comp[1]:
                if "pher" in comp[0]:
                    if "pher" not in comp[1]:
                        print(i)
                        sig_cols_morph_wt.append(n)

        elif "Spores" not in comp[1]:
            if "spherule" in comp[0] and "spherule" not in comp[1]:
                print(i)
                sig_cols_morph_ryp1.append(n)

        
print(sig_cols_morph_wt)

sig_cols = [n for (n,i) in enumerate(limma1_sig.fieldnames) if(i.endswith("_sig"))]
contrast_cols = [n-len(sig_cols) for n in sig_cols_morph_wt]
limma1_morphdep_all_wt = CdtFile.fromPrototype(limma1_sig, probes = [i for i in limma1_sig
                                                        if(all([(i[j] != 0.) for j in sig_cols_morph_wt]))])
tree = limma1_morphdep_all_wt.cluster(cols=contrast_cols,dist="u",method="m")
limma1_morphdep_all_wt.writeCdtGtr("limma1_morphdep_all_wt.contrasts_um",tree)
print(len(limma1_morphdep_all_wt))

sig_cols = [n for (n,i) in enumerate(limma1_sig.fieldnames) if(i.endswith("_sig"))]
contrast_cols = [n-len(sig_cols) for n in sig_cols_morph_ryp1]
limma1_morphdep_all_ryp1 = CdtFile.fromPrototype(limma1_sig, probes = [i for i in limma1_sig
                                                        if(all([(i[j] != 0.) for j in sig_cols_morph_ryp1]))])
tree = limma1_morphdep_all_ryp1.cluster(cols=contrast_cols,dist="u",method="m")
limma1_morphdep_all_ryp1.writeCdtGtr("limma1_morphdep_all_ryp1.contrasts_um",tree)
print(len(limma1_morphdep_all_ryp1))
```

```
D1_spherule_Ryp1/D1_myc_Ryp1_sig
D1_spherule_Sil/D1_myc_Sil_sig
D2_spherule_Ryp1/D2_myc_Ryp1_sig
D2_spherule_Sil/D2_myc_Sil_sig
D3_spherules_Ryp1/D3_myc_Ryp1_sig
D3_spherules_Sil/D3_myc_Sil_sig
D6_spherules_Ryp1/D6_myc_Ryp1_sig
D6_spherules_Sil/D6_myc_Sil_sig
[148, 159, 169, 187]
```

```
Building array...
Building distance matrix...
Clustering...
```

```
551
318
```

```
Building array...
Building distance matrix...
Clustering...
```

## Now enforce directionality of the morphology gene change¶

In [33]:

```
sig_cols_morph_wt = [] 
sig_cols_morph_ryp1 = []
for n,i in (enumerate(limma1_sig.fieldnames)):
    if i.endswith("_sig") and 'ight' not in i:
        comp = i.split("/")
        if "Sil" in comp[0] or "sil" in comp[0]:
            if "Ryp" not in comp[1] and "ryp" not in comp[1] and "Spores" not in comp[1]:
                if "pher" in comp[0]:
                    if "pher" not in comp[1]:
                        print(i)
                        sig_cols_morph_wt.append(n)

        elif "Spores" not in comp[1]:
            if "spherule" in comp[0] and "spherule" not in comp[1]:
                print(i)
                sig_cols_morph_ryp1.append(n)

        
print(sig_cols_morph_wt)

sig_cols = [n for (n,i) in enumerate(limma1_sig.fieldnames) if(i.endswith("_sig"))]
contrast_cols = [n-len(sig_cols) for n in sig_cols_morph_wt]
limma1_morphdep_all_wt_ind = CdtFile.fromPrototype(limma1_sig, probes = [i for i in limma1_sig
                                                        if(all([(i[j] == 4.0) for j in sig_cols_morph_wt]))])
tree = limma1_morphdep_all_wt_ind.cluster(cols=contrast_cols,dist="u",method="m")
limma1_morphdep_all_wt_ind.writeCdtGtr("limma1_morphdep_all_wt_induced.contrasts_um",tree)
print(len(limma1_morphdep_all_wt_ind))
limma1_morphdep_all_wt_rep = CdtFile.fromPrototype(limma1_sig, probes = [i for i in limma1_sig
                                                        if(all([(i[j] == -4.0) for j in sig_cols_morph_wt]))])
tree = limma1_morphdep_all_wt_rep.cluster(cols=contrast_cols,dist="u",method="m")
limma1_morphdep_all_wt_rep.writeCdtGtr("limma1_morphdep_all_wt_repressed.contrasts_um",tree)
print(len(limma1_morphdep_all_wt_rep))

sig_cols = [n for (n,i) in enumerate(limma1_sig.fieldnames) if(i.endswith("_sig"))]
contrast_cols = [n-len(sig_cols) for n in sig_cols_morph_ryp1]
limma1_morphdep_all_ryp1_ind = CdtFile.fromPrototype(limma1_sig, probes = [i for i in limma1_sig
                                                        if(all([(i[j] == 4.0) for j in sig_cols_morph_ryp1]))])
tree = limma1_morphdep_all_ryp1_ind.cluster(cols=contrast_cols,dist="u",method="m")
limma1_morphdep_all_ryp1_ind.writeCdtGtr("limma1_morphdep_all_ryp1_induced.contrasts_um",tree)
print(len(limma1_morphdep_all_ryp1_ind))
limma1_morphdep_all_ryp1_rep = CdtFile.fromPrototype(limma1_sig, probes = [i for i in limma1_sig
                                                        if(all([(i[j] == -4.0) for j in sig_cols_morph_ryp1]))])
tree = limma1_morphdep_all_ryp1_rep.cluster(cols=contrast_cols,dist="u",method="m")
limma1_morphdep_all_ryp1_rep.writeCdtGtr("limma1_morphdep_all_ryp1_repressed.contrasts_um",tree)
print(len(limma1_morphdep_all_ryp1_rep))
```

```
D1_spherule_Ryp1/D1_myc_Ryp1_sig
D1_spherule_Sil/D1_myc_Sil_sig
D2_spherule_Ryp1/D2_myc_Ryp1_sig
D2_spherule_Sil/D2_myc_Sil_sig
D3_spherules_Ryp1/D3_myc_Ryp1_sig
D3_spherules_Sil/D3_myc_Sil_sig
D6_spherules_Ryp1/D6_myc_Ryp1_sig
D6_spherules_Sil/D6_myc_Sil_sig
[148, 159, 169, 187]
273
```

```
Building array...
Building distance matrix...
Clustering...
Building array...
Building distance matrix...
Clustering...
```

```
239
172
133
```

```
Building array...
Building distance matrix...
Clustering...
Building array...
Building distance matrix...
Clustering...
```

### Ryp1-regulated genes in arthroconidia¶

In [34]:

```
sig_col_arth = []
for n,i in (enumerate(limma1_sig.fieldnames)):
    if i.endswith("_sig"):
        if "Spore" in i.split("/")[0]:
            if "Spore" in i.split("/")[1]:
                sig_col_arth.append(n)
                print(i)

print(sig_col_arth)

sig_cols = [n for (n,i) in enumerate(limma1_sig.fieldnames) if(i.endswith("_sig"))]
contrast_cols = [n-len(sig_cols) for n in sig_cols]
probes = []
for i in limma1_sig:
        if all([(i[j] != 0.) for j in sig_col_arth]):
            probes.append(i)

limma1_ryp1_arth = CdtFile.fromPrototype(limma1_sig, probes = probes)   
tree = limma1_ryp1_arth.cluster(cols=contrast_cols,dist="u",method="m")
limma1_ryp1_arth.writeCdtGtr("limma1_ryp1dep_arth.contrasts_um",tree)
len(limma1_ryp1_arth)
```

```
Spores_Sil/Spores_Ryp1_sig
[141]
```

```
Building array...
Building distance matrix...
Clustering...
```

Out[34]:

```
3599
```

# Venn diagram section¶

In [35]:

```
#Basis for Fig 3D
plt.figure()
morph_WT = set(i.uniqid for i in limma1_morphdep_all_wt)
spher_ryp1 = set(i.uniqid for i in limma1_ryp1dep_spherule)
myc_ryp1 = set(i.uniqid for i in limma1_ryp1dep_myc)
venn = venn3([spher_ryp1,myc_ryp1, morph_WT], ("Spher_ryp1", "Myc_ryp1", "morph"))
print(len(morph_WT), len(spher_ryp1), len(myc_ryp1))
```

```
551 452 262
```

In [36]:

```
# What genes are dependent on Ryp1 in all conditions and also differential between spherule/mycelia in WT?
morph_ryp1_overlap_no_arth = [ ] 
for row in limma1_morphdep_all_wt:
    gene_morph = row.uniqid
    for row2 in limma1_ryp1dep_all_minus_arth:
        gene_ryp = row2.uniqid
        if gene_morph == gene_ryp:
            morph_ryp1_overlap_no_arth.append(gene_ryp)
            
print(len(morph_ryp1_overlap_no_arth))
print(sorted(morph_ryp1_overlap_no_arth))
```

```
17
['D8B26_002505', 'D8B26_002647', 'D8B26_002779', 'D8B26_003449_1', 'D8B26_003981', 'D8B26_004329', 'D8B26_005043', 'D8B26_005065', 'D8B26_005342', 'D8B26_005798', 'D8B26_006094', 'D8B26_006286', 'D8B26_006627_1', 'D8B26_006995', 'D8B26_007175', 'D8B26_007313', 'D8B26_008269']
```

# Looking at overlaps between spherule-ryp1-regulated genes and morphology genes¶

## What is the overlap between ryp1-induced genes (in spherules) and spherule-induced genes?¶

In [37]:

```
sig_cols_ryp_spher = [] 
for n,i in (enumerate(limma1_sig.fieldnames)):
    if i.endswith("_sig"):
        if "Sil" in i.split("/")[0] or "sil" in i.split("/")[0]:
            if "Ryp1" in i.split("/")[1] or "ryp1" in i.split("/")[1]:
                if "spherule" in i and "Eight" not in i:
                    sig_cols_ryp_spher.append(n)
                    print(i)
sig_cols = [n for (n,i) in enumerate(limma1_sig.fieldnames) if(i.endswith("_sig"))]
contrast_cols = [n-len(sig_cols) for n in sig_cols]
limma1_ryp1dep_spherule_ind = CdtFile.fromPrototype(limma1_sig, probes = [i for i in limma1_sig 
                                                        if(all([(i[j] == 4.0) for j in sig_cols_ryp_spher]))])
tree = limma1_ryp1dep_spherule_ind.cluster(cols=contrast_cols,dist="u",method="m")
limma1_ryp1dep_spherule_ind.writeCdtGtr("limma1_ryp1dep_spherule_induced.contrasts_um",tree)

ryp1spher_ind_spher_ind_overlap = [ ] 
for row in limma1_morphdep_all_wt_ind:
    gene_morph = row.uniqid
    for row2 in limma1_ryp1dep_spherule_ind:
        gene_ryp = row2.uniqid
        if gene_morph == gene_ryp:
            ryp1spher_ind_spher_ind_overlap.append(gene_ryp)
            
print(len(ryp1spher_ind_spher_ind_overlap))

limma1_ryp1spher_ind_spher_ind_overlap = CdtFile.fromPrototype(limma1_sig, probes = [i for i in limma1_sig
                                                        if i.uniqid in ryp1spher_ind_spher_ind_overlap]) 
tree = limma1_ryp1spher_ind_spher_ind_overlap.cluster(cols=contrast_cols,dist="u",method="m")
limma1_ryp1spher_ind_spher_ind_overlap.writeCdtGtr("limma1_ryp1spher_ind_spher_ind_overlap.contrasts_um",tree)

limma1_ryp1dep_spherule_rep = CdtFile.fromPrototype(limma1_sig, probes = [i for i in limma1_sig 
                                                        if(all([(i[j] == -4.0) for j in sig_cols_ryp_spher]))])
tree = limma1_ryp1dep_spherule_rep.cluster(cols=contrast_cols,dist="u",method="m")
limma1_ryp1dep_spherule_rep.writeCdtGtr("limma1_ryp1dep_spherule_repressed.contrasts_um",tree)
```

```
D1_spherule_Sil/D1_spherule_Ryp1_sig
D2_spherule_Sil/D2_spherule_Ryp1_sig
D3_spherules_Sil/D3_spherules_Ryp1_sig
D4_spherules_Sil/D4_spherules_Ryp1_sig
D5_spherules_Sil/D5_spherules_Ryp1_sig
D6_spherules_Sil/D6_spherules_Ryp1_sig
79
```

```
Building array...
Building distance matrix...
Clustering...
Building array...
Building distance matrix...
Clustering...
Building array...
Building distance matrix...
Clustering...
```

# Now quantify ryp1-dependent transcripts at each timepoint¶

In [38]:

```
sig_cols_ryp = [] 
ryp_tp_histogram_pos = {}
ryp_tp_histogram_neg = {}
for n,i in (enumerate(limma1_sig.fieldnames)):
    if i.endswith("_sig"):
        if "Sil" in i.split("/")[0] or "sil" in i.split("/")[0]:
            if "Ryp1" in i.split("/")[1] or "ryp1" in i.split("/")[1]:
                sig_cols_ryp.append(n)
                print(i)
                ryp_tp_histogram_pos[i] = 0
                ryp_tp_histogram_neg[i] = 0
sig_cols = [n for (n,i) in enumerate(limma1_sig.fieldnames) if(i.endswith("_sig"))]
contrast_cols = [n-len(sig_cols) for n in sig_cols]

for sig_col in sig_cols_ryp: 
    for i in limma1_sig:
        if i[sig_col] == 4.0:
            ryp_tp_histogram_pos[limma1_sig.fieldnames[sig_col]] += 1
        elif i[sig_col] == -4.0:
            ryp_tp_histogram_neg[limma1_sig.fieldnames[sig_col]] += 1
        
    
print(ryp_tp_histogram_pos)
print(ryp_tp_histogram_neg)
```

```
Spores_Sil/Spores_Ryp1_sig
D1_myc_Sil/D1_myc_Ryp1_sig
D1_spherule_Sil/D1_spherule_Ryp1_sig
D2_myc_Sil/D2_myc_Ryp1_sig
D2_spherule_Sil/D2_spherule_Ryp1_sig
D3_myc_Sil/D3_myc_Ryp1_sig
D3_spherules_Sil/D3_spherules_Ryp1_sig
D4_spherules_Sil/D4_spherules_Ryp1_sig
D5_spherules_Sil/D5_spherules_Ryp1_sig
D6_myc_Sil/D6_myc_Ryp1_sig
D6_spherules_Sil/D6_spherules_Ryp1_sig
Eighth_myc_Sil/Eighth_myc_Ryp1_sig
Eighth_spherule_Sil/Eighth_spherule_ryp1_sig
{'Spores_Sil/Spores_Ryp1_sig': 1536, 'D1_myc_Sil/D1_myc_Ryp1_sig': 1169, 'D1_spherule_Sil/D1_spherule_Ryp1_sig': 1068, 'D2_myc_Sil/D2_myc_Ryp1_sig': 1044, 'D2_spherule_Sil/D2_spherule_Ryp1_sig': 1285, 'D3_myc_Sil/D3_myc_Ryp1_sig': 627, 'D3_spherules_Sil/D3_spherules_Ryp1_sig': 1376, 'D4_spherules_Sil/D4_spherules_Ryp1_sig': 1535, 'D5_spherules_Sil/D5_spherules_Ryp1_sig': 869, 'D6_myc_Sil/D6_myc_Ryp1_sig': 545, 'D6_spherules_Sil/D6_spherules_Ryp1_sig': 944, 'Eighth_myc_Sil/Eighth_myc_Ryp1_sig': 1145, 'Eighth_spherule_Sil/Eighth_spherule_ryp1_sig': 896}
{'Spores_Sil/Spores_Ryp1_sig': 2063, 'D1_myc_Sil/D1_myc_Ryp1_sig': 1145, 'D1_spherule_Sil/D1_spherule_Ryp1_sig': 1101, 'D2_myc_Sil/D2_myc_Ryp1_sig': 782, 'D2_spherule_Sil/D2_spherule_Ryp1_sig': 1223, 'D3_myc_Sil/D3_myc_Ryp1_sig': 777, 'D3_spherules_Sil/D3_spherules_Ryp1_sig': 1152, 'D4_spherules_Sil/D4_spherules_Ryp1_sig': 1430, 'D5_spherules_Sil/D5_spherules_Ryp1_sig': 1025, 'D6_myc_Sil/D6_myc_Ryp1_sig': 426, 'D6_spherules_Sil/D6_spherules_Ryp1_sig': 1062, 'Eighth_myc_Sil/Eighth_myc_Ryp1_sig': 1442, 'Eighth_spherule_Sil/Eighth_spherule_ryp1_sig': 1098}
```

In [39]:

```
#Figure 3A
pos_reordered = sorted(ryp_tp_histogram_pos.items(), key=lambda t:(t[0].split("_")[1],t[0].split("_")[0]))
pos_reordered_new = [pos_reordered[0]] + [['',0]] +[pos_reordered[8]]+pos_reordered[6:8] + pos_reordered[9:]+ [['',0]] + [pos_reordered[5]]+pos_reordered[1:5]
pos_keys = []
pos_values = []
for entry in pos_reordered_new:
    pos_keys.append(entry[0])
    pos_values.append(entry[1])

neg_reordered = sorted(ryp_tp_histogram_neg.items(), key=lambda t:(t[0].split("_")[1],t[0].split("_")[0]))
neg_reordered_new = [neg_reordered[0]] + [['',0]] +[neg_reordered[8]]+neg_reordered[6:8] + neg_reordered[9:]+[['',0]]+[neg_reordered[5]]+neg_reordered[1:5]
neg_keys = []
neg_values = []
for entry in neg_reordered_new:
    neg_keys.append(entry[0])
    neg_values.append(-1*entry[1])

x = range(len(neg_values))
fig = plt.figure()
ax = plt.subplot(111)
ax.bar(x,pos_values, width=1, color='mediumpurple')
ax.bar(x,neg_values, width=1, color='limegreen')
fig.savefig("ryp1_dependence_counts.svg")
```

### Let's highlight morphology-dependent genes within this¶

In [40]:

```
sig_cols_ryp = [] 
ryp_tp_histogram_pos = {}
ryp_tp_histogram_morph_pos = {}
ryp_tp_histogram_neg = {}
ryp_tp_histogram_morph_neg = {}
for n,i in (enumerate(limma1_sig.fieldnames)):
    if i.endswith("_sig"):
        if "Sil" in i.split("/")[0] or "sil" in i.split("/")[0]:
            if "Ryp1" in i.split("/")[1] or "ryp1" in i.split("/")[1]:
                sig_cols_ryp.append(n)
                ryp_tp_histogram_pos[i] = 0
                ryp_tp_histogram_neg[i] = 0
                ryp_tp_histogram_morph_pos[i] = 0
                ryp_tp_histogram_morph_neg[i] = 0
sig_cols = [n for (n,i) in enumerate(limma1_sig.fieldnames) if(i.endswith("_sig"))]
contrast_cols = [n-len(sig_cols) for n in sig_cols]

for sig_col in sig_cols_ryp: 
    for i in limma1_sig:
        if i[sig_col] == 4.0:
            if "Spores" not in limma1_sig.fieldnames[sig_col] and "D4" not in limma1_sig.fieldnames[sig_col] and "D5" not in limma1_sig.fieldnames[sig_col]:
                if "D3" in limma1_sig.fieldnames[sig_col] or "D6" in limma1_sig.fieldnames[sig_col]:
                    morph_dep_fieldname = "{0}_spherules_Sil/{0}_myc_Sil_sig".format(limma1_sig.fieldnames[sig_col].split("_")[0])
                else:
                    morph_dep_fieldname = "{0}_spherule_Sil/{0}_myc_Sil_sig".format(limma1_sig.fieldnames[sig_col].split("_")[0])
                for m,field in enumerate(limma1_sig.fieldnames):
                    if field.endswith("_sig"):
                        if field == morph_dep_fieldname:
                            if i[m] != 0.:
                                ryp_tp_histogram_morph_pos[limma1_sig.fieldnames[sig_col]] += 1
                                ryp_tp_histogram_pos[limma1_sig.fieldnames[sig_col]] += 1
                            else:
                                ryp_tp_histogram_pos[limma1_sig.fieldnames[sig_col]] += 1
            else:
                ryp_tp_histogram_pos[limma1_sig.fieldnames[sig_col]] += 1 
        elif i[sig_col] == -4.0:
            if "Spores" not in limma1_sig.fieldnames[sig_col] and "D4" not in limma1_sig.fieldnames[sig_col] and "D5" not in limma1_sig.fieldnames[sig_col]:
                if "D3" in limma1_sig.fieldnames[sig_col] or "D6" in limma1_sig.fieldnames[sig_col]:
                    morph_dep_fieldname = "{0}_spherules_Sil/{0}_myc_Sil_sig".format(limma1_sig.fieldnames[sig_col].split("_")[0])
                else:
                    morph_dep_fieldname = "{0}_spherule_Sil/{0}_myc_Sil_sig".format(limma1_sig.fieldnames[sig_col].split("_")[0])
                for m,field in enumerate(limma1_sig.fieldnames):
                    if field.endswith("_sig"):
                        if field == morph_dep_fieldname:
                            if i[m] != 0.:
                                ryp_tp_histogram_morph_neg[limma1_sig.fieldnames[sig_col]] += 1
                                ryp_tp_histogram_neg[limma1_sig.fieldnames[sig_col]] += 1
                            else:
                                ryp_tp_histogram_neg[limma1_sig.fieldnames[sig_col]] += 1
            else:
                ryp_tp_histogram_neg[limma1_sig.fieldnames[sig_col]] += 1
```

In [41]:

```
#Figure 3B
pos_reordered = sorted(ryp_tp_histogram_pos.items(), key=lambda t:(t[0].split("_")[1],t[0].split("_")[0]))
pos_reordered_new = [pos_reordered[0]] + [['',0]] +[pos_reordered[8]]+pos_reordered[6:8] + pos_reordered[9:]+ [['',0]] + [pos_reordered[5]]+pos_reordered[1:5]

pos_keys = []
pos_values = []
pos_morph_values = []
for z, entry in enumerate(pos_reordered_new):
    pos_keys.append(entry[0])
    pos_values.append(entry[1])
    if entry[0] != '':
        pos_morph_values.append(entry[1] - ryp_tp_histogram_morph_pos[entry[0]])
    else:
        pos_morph_values.append(0)
        
neg_reordered = sorted(ryp_tp_histogram_neg.items(), key=lambda t:(t[0].split("_")[1],t[0].split("_")[0]))
neg_reordered_new = [neg_reordered[0]] + [['',0]] +[neg_reordered[8]]+neg_reordered[6:8] + neg_reordered[9:]+[['',0]]+[neg_reordered[5]]+neg_reordered[1:5]
print(neg_reordered_new)
neg_keys = []
neg_values = []
neg_morph_values = []
for y,entry in enumerate(neg_reordered_new):
    neg_keys.append(entry[0])
    neg_values.append(-1*entry[1])
    if entry[0] != "":
        neg_morph_values.append(-1*(entry[1] - ryp_tp_histogram_morph_neg[entry[0]]))
    else:
        neg_morph_values.append(0)
    
x = range(len(neg_values))
fig = plt.figure()
ax = plt.subplot(111)
ax.bar(x,pos_values, width=1, color='rebeccapurple')
ax.bar(x,pos_morph_values, width=1, color='mediumpurple')
ax.bar(x,neg_values, width=1, color='darkgreen')
ax.bar(x,neg_morph_values, width=1, color="limegreen")
fig.savefig("ryp1_dependence_counts_with_morph_relationship.svg")
```

```
[('Spores_Sil/Spores_Ryp1_sig', 2063), ['', 0], ('Eighth_spherule_Sil/Eighth_spherule_ryp1_sig', 1098), ('D1_spherule_Sil/D1_spherule_Ryp1_sig', 1101), ('D2_spherule_Sil/D2_spherule_Ryp1_sig', 1223), ('D3_spherules_Sil/D3_spherules_Ryp1_sig', 1152), ('D4_spherules_Sil/D4_spherules_Ryp1_sig', 1430), ('D5_spherules_Sil/D5_spherules_Ryp1_sig', 1025), ('D6_spherules_Sil/D6_spherules_Ryp1_sig', 1062), ['', 0], ('Eighth_myc_Sil/Eighth_myc_Ryp1_sig', 1442), ('D1_myc_Sil/D1_myc_Ryp1_sig', 1145), ('D2_myc_Sil/D2_myc_Ryp1_sig', 782), ('D3_myc_Sil/D3_myc_Ryp1_sig', 777), ('D6_myc_Sil/D6_myc_Ryp1_sig', 426)]
```

# Now look at morphology-dependent transcripts in WT at each timepoint¶

In [42]:

```
sig_cols_morph = []
morph_tp_histogram_pos = {}
morph_tp_histogram_ryp_spher_pos = {}
morph_tp_histogram_ryp_myc_pos = {}
morph_tp_histogram_neg = {}
morph_tp_histogram_ryp_spher_neg = {}
morph_tp_histogram_ryp_myc_neg = {}
for n,i in (enumerate(limma1_sig.fieldnames)):
    if i.endswith("_sig"):
        comp = i.split("/")
        if "Sil" in comp[0] or "sil" in comp[0]:
            if "Ryp" not in comp[1] and "ryp" not in comp[1] and "Spores" not in comp[1]:
                if "spherule" in comp[0] and "spherule" not in comp[1]:
                        print(i)
                        sig_cols_morph.append(n)
                        morph_tp_histogram_pos[i] = 0
                        morph_tp_histogram_ryp_spher_pos[i] = 0
                        morph_tp_histogram_ryp_myc_pos[i] = 0
                        morph_tp_histogram_neg[i] = 0
                        morph_tp_histogram_ryp_spher_neg[i] = 0
                        morph_tp_histogram_ryp_myc_neg[i] = 0
        elif "Spores" not in comp[1]:
            if "spherule" in comp[0] and "spherule" not in comp[1]:
                print(i)
                sig_cols_morph.append(n)
                morph_tp_histogram_pos[i] = 0
                morph_tp_histogram_ryp_spher_pos[i] = 0
                morph_tp_histogram_ryp_myc_pos[i] = 0
                morph_tp_histogram_neg[i] = 0
                morph_tp_histogram_ryp_spher_neg[i] = 0
                morph_tp_histogram_ryp_myc_neg[i] = 0   
                    
for sig_col in sig_cols_morph: 
    for i in limma1_sig:
        if i[sig_col] == 4.0:
            if "D3" in limma1_sig.fieldnames[sig_col] or "D6" in limma1_sig.fieldnames[sig_col]:
                ryp_spher_fieldname = "{0}_spherules_Sil/{0}_spherules_Ryp1_sig".format(limma1_sig.fieldnames[sig_col].split("_")[0])
            elif "ighth" in limma1_sig.fieldnames[sig_col]:
                ryp_spher_fieldname = "{0}_spherule_Sil/{0}_spherule_ryp1_sig". format(limma1_sig.fieldnames[sig_col].split("_")[0])
            elif "D2" in limma1_sig.fieldnames[sig_col] or "D1" in limma1_sig.fieldnames[sig_col]:
                ryp_spher_fieldname = "{0}_spherule_Sil/{0}_spherule_Ryp1_sig".format(limma1_sig.fieldnames[sig_col].split("_")[0])
            else:
                ryp_spher_fieldname = "{0}_spherule_Sil/{0}_spherules_Ryp1_sig".format(limma1_sig.fieldnames[sig_col].split("_")[0])
            ryp_myc_fieldname = "{0}_myc_Sil/{0}_myc_Ryp1_sig".format(limma1_sig.fieldnames[sig_col].split("_")[0])
                
            for m,field in enumerate(limma1_sig.fieldnames):
                if field.endswith("_sig"):
                    if field == ryp_spher_fieldname:
                        if i[m] != 0.:
                            morph_tp_histogram_ryp_spher_pos[limma1_sig.fieldnames[sig_col]] += 1
                            morph_tp_histogram_pos[limma1_sig.fieldnames[sig_col]] += 1
                        else:
                            morph_tp_histogram_pos[limma1_sig.fieldnames[sig_col]] += 1
                    elif field == ryp_myc_fieldname:
                        if i[m] != 0.:
                            morph_tp_histogram_ryp_myc_pos[limma1_sig.fieldnames[sig_col]] += 1
        
        elif i[sig_col] == -4.0:
            if "D3" in limma1_sig.fieldnames[sig_col] or "D6" in limma1_sig.fieldnames[sig_col]:
                ryp_spher_fieldname = "{0}_spherules_Sil/{0}_spherules_Ryp1_sig".format(limma1_sig.fieldnames[sig_col].split("_")[0])
            elif "ighth" in limma1_sig.fieldnames[sig_col]:
                ryp_spher_fieldname = "{0}_spherule_Sil/{0}_spherule_ryp1_sig". format(limma1_sig.fieldnames[sig_col].split("_")[0])
            elif "D2" in limma1_sig.fieldnames[sig_col] or "D1" in limma1_sig.fieldnames[sig_col]:
                ryp_spher_fieldname = "{0}_spherule_Sil/{0}_spherule_Ryp1_sig".format(limma1_sig.fieldnames[sig_col].split("_")[0])
            else:
                ryp_spher_fieldname = "{0}_spherule_Sil/{0}_spherules_Ryp1_sig".format(limma1_sig.fieldnames[sig_col].split("_")[0])
            ryp_myc_fieldname = "{0}_myc_Sil/{0}_myc_Ryp1_sig".format(limma1_sig.fieldnames[sig_col].split("_")[0])
                
            for m,field in enumerate(limma1_sig.fieldnames):
                if field.endswith("_sig"):
                    if field == ryp_spher_fieldname:
                        if i[m] != 0.:
                            morph_tp_histogram_ryp_spher_neg[limma1_sig.fieldnames[sig_col]] += 1
                            morph_tp_histogram_neg[limma1_sig.fieldnames[sig_col]] += 1
                        else:
                            morph_tp_histogram_neg[limma1_sig.fieldnames[sig_col]] += 1
                    elif field == ryp_myc_fieldname:
                        if i[m] != 0.:
                            morph_tp_histogram_ryp_myc_neg[limma1_sig.fieldnames[sig_col]] += 1
```

```
D1_spherule_Ryp1/D1_myc_Ryp1_sig
D1_spherule_Sil/D1_myc_Sil_sig
D2_spherule_Ryp1/D2_myc_Ryp1_sig
D2_spherule_Sil/D2_myc_Sil_sig
D3_spherules_Ryp1/D3_myc_Ryp1_sig
D3_spherules_Sil/D3_myc_Sil_sig
D6_spherules_Ryp1/D6_myc_Ryp1_sig
D6_spherules_Sil/D6_myc_Sil_sig
Eighth_spherule_Sil/Eighth_myc_Sil_sig
Eighth_spherule_ryp1/Eighth_myc_Ryp1_sig
```

In [43]:

```
pos_reordered = sorted(morph_tp_histogram_pos.items(), key=lambda t:(t[0].split("_")[-2],t[0].split("_")[0]))
pos_reordered_new = [pos_reordered[9]]+pos_reordered[5:9] + [["", 0]] +[pos_reordered[4]] + pos_reordered[:4]
pos_keys = []
pos_values = []
pos_ryp_spher_values = []
pos_ryp_myc_values = []
for entry in pos_reordered_new:
    pos_keys.append(entry[0])
    pos_values.append(entry[1])
    if entry[0] != '':
        pos_ryp_spher_values.append(entry[1] - morph_tp_histogram_ryp_spher_pos[entry[0]])
        pos_ryp_myc_values.append(entry[1] - morph_tp_histogram_ryp_myc_pos[entry[0]])
    else:
        pos_ryp_spher_values.append(0)
        pos_ryp_myc_values.append(0)

neg_reordered = sorted(morph_tp_histogram_neg.items(), key=lambda t:(t[0].split("_")[-2],t[0].split("_")[0]))
neg_reordered_new = [neg_reordered[9]]+neg_reordered[5:9] + [["", 0]] +[neg_reordered[4]] + neg_reordered[:4]
neg_keys = []
neg_values = []
neg_ryp_spher_values = []
neg_ryp_myc_values = []
for entry in neg_reordered_new:
    neg_keys.append(entry[0])
    neg_values.append(-1*entry[1])
    if entry[0] != '':
        neg_ryp_spher_values.append(-1*(entry[1] - morph_tp_histogram_ryp_spher_neg[entry[0]]))
        neg_ryp_myc_values.append(-1*(entry[1] - morph_tp_histogram_ryp_myc_neg[entry[0]]))
    else:
        neg_ryp_spher_values.append(0)
        neg_ryp_myc_values.append(0)
```

In [44]:

```
#Fig 3E
neg_values_for_wt = neg_values[0:5]
pos_values_for_wt = pos_values[0:5]
neg_ryp_spher_values_for_wt = neg_ryp_spher_values[0:5]
pos_ryp_spher_values_for_wt = pos_ryp_spher_values[0:5]


neg_values_for_ryp1 = neg_values[6:]
pos_values_for_ryp1 = pos_values[6:]
pos_ryp_spher_values_for_ryp1 = pos_ryp_spher_values[6:]
neg_ryp_spher_values_for_ryp1 = neg_ryp_spher_values[6:]

#Graph just WT without highlighting ryp1-dependent
x = range(len(neg_ryp_spher_values_for_wt))
fig = plt.figure(figsize=(3,6))
ax = plt.subplot(111)
ax.bar(x,pos_values_for_wt, width=1, color='yellow')
ax.bar(x,neg_values_for_wt, width=1, color='lightblue')
fig.tight_layout()
fig.savefig("morph_dependence_counts_wt_only.svg")
```

In [45]:

```
#Fig 3F
neg_values_for_wt = neg_values[0:5]
pos_values_for_wt = pos_values[0:5]
neg_ryp_spher_values_for_wt = neg_ryp_spher_values[0:5]
pos_ryp_spher_values_for_wt = pos_ryp_spher_values[0:5]

#Graph
x = range(len(neg_ryp_spher_values_for_wt))
fig = plt.figure(figsize=(3,6))
ax = plt.subplot(111)
ax.bar(x,pos_values_for_wt, width=1, color='darkgoldenrod')
ax.bar(x,pos_ryp_spher_values_for_wt, width=1, color = 'yellow')
ax.bar(x,neg_values_for_wt, width=1, color='blue')
ax.bar(x,neg_ryp_spher_values_for_wt, width=1, color = 'lightblue')
fig.tight_layout()
fig.savefig("morph_dependence_counts_with_ryp1_spher_wt_only.svg")
```

In [46]:

```
#Fig 3G
neg_values_for_wt = neg_values[0:5]
pos_values_for_wt = pos_values[0:5]
neg_ryp_myc_values_for_wt = neg_ryp_myc_values[0:5]
pos_ryp_myc_values_for_wt = pos_ryp_myc_values[0:5]


#Graph
x = range(len(neg_ryp_myc_values_for_wt))
fig = plt.figure(figsize=(3,6))
ax = plt.subplot(111)
ax.bar(x,pos_values_for_wt, width=1, color='darkgoldenrod')
ax.bar(x,pos_ryp_myc_values_for_wt, width=1, color = 'yellow')
ax.bar(x,neg_values_for_wt, width=1, color='blue')
ax.bar(x,neg_ryp_myc_values_for_wt, width=1, color = 'lightblue')
fig.tight_layout()
fig.savefig("morph_dependence_counts_with_ryp1_myc_wt_only.svg")
```

# Graph Ryp1 Expression in WT¶

In [47]:

```
data = fit2.get_transcript("D8B26_000722")
graph_cols = []
for l,parameter in enumerate(data.fit.parameter_names):
    if "yp1" not in parameter and "DMEM" not in parameter and "RPMI" not in parameter:
        graph_cols.append(l)
```

In [48]:

```
xticklabels = ['Spores_Sil',
               'Eighth_spherule_Sil',
               'D1_spherule_Sil',
               'D2_spherule_Sil',
               'D3_spherules_Sil',
               'D4_spherules_Sil', 
               'D5_spherules_Sil',  
               'D6_spherules_Sil',
               'Eighth_myc_Sil',
               'D1_myc_Sil',  
               'D2_myc_Sil',  
               'D3_myc_Sil', 
               'D6_myc_Sil',  ]
```

In [49]:

```
yvalues = []
xs = []
x = 1
stdevs = []
for label in xticklabels:
    for j, label2 in enumerate(data.fit.parameter_names):
        if label == label2:
            yvalues.append(data.parameters[j])
            std = statistics.stdev(data.obs[j])
            stdevs.append(std)
            xs.append(x)
            x += 1
```

In [50]:

```
#Fig S3E
fit = plt.figure()
plt.plot(xs, yvalues, marker="o", linestyle = "none", color="black")
plt.errorbar(xs, yvalues, yerr = stdevs, color='black', linestyle='none')
plt.ylim(0,8.5)
plt.axvline(8.5, color='black')
plt.savefig("ryp1_wt_expression.svg")
```

# Next, look at scatter plots of WT spher/hyph vs WT/ryp1 in spherulation conditions (or hyph conditions) at 8h, D1, D2, D3, and D6¶

In [51]:

```
scatter_morphology = {}
scatter_ryp1_hyph = {}
scatter_ryp1_spher = {}

#get list of columns for WT spher/myc comparison log2 values
log2cpm_cols_morph = []
for n,i in (enumerate(limma1_cdt.fieldnames)):
    if "sig" not in i and "/" in i:
        comp = i.split("/")
        if "pher" in comp[0] and "myc" in comp[1]:
            if "il" in i:
                log2cpm_cols_morph.append(n)

#get log2(cpm) comparisons for morphology comparisons for all genes
for col in log2cpm_cols_morph:
    cpms = []
    for i in limma1_cdt:
        tp = limma1_cdt.fieldnames[col].split("_")[0]
        cpms.append(i[col])
    scatter_morphology[tp] = cpms
                    
#get list of columns for WT/ryp1 comparison in spherule timepoints
log2cpm_cols_ryp1_spher = []
for n,i in (enumerate(limma1_cdt.fieldnames)):
    if "sig" not in i and "/" in i:
        comp = i.split("/")
        if "pher" in comp[0] and "pher" in comp[1]:
            if "il" in comp[0] and "yp" in comp[1]:
                if "D4" not in i and "D5" not in i:
                    log2cpm_cols_ryp1_spher.append(n)
                    
#get log2(cpm) comparisons for spherule ryp1-dependent comparisons for all genes
for col in log2cpm_cols_ryp1_spher:
    cpms = []
    for i in limma1_cdt:
        tp = limma1_cdt.fieldnames[col].split("_")[0]
        cpms.append(i[col])
    scatter_ryp1_spher[tp] = cpms
    
#get list of columns for WT/ryp1 comparison in hyph timepoints
log2cpm_cols_ryp1_hyph = []
for n,i in (enumerate(limma1_cdt.fieldnames)):
    if "sig" not in i and "/" in i:
        comp = i.split("/")
        if "yc" in comp[0] and "yc" in comp[1]:
            if "il" in comp[0] and "yp" in comp[1]:
                if "D4" not in i and "D5" not in i:
                    log2cpm_cols_ryp1_hyph.append(n)
                    
#get log2(cpm) comparisons for hyphal ryp1-dependent comparisons for all genes
for col in log2cpm_cols_ryp1_hyph:
    cpms = []
    for i in limma1_cdt:
        tp = limma1_cdt.fieldnames[col].split("_")[0]
        cpms.append(i[col])
    scatter_ryp1_hyph[tp] = cpms
```

In [52]:

```
#Looking at morphology-regulated genes correlation with spherule-ryp1-reg, hyphal-ryp1-reg, and arthr-ryp1-regulated
#Basis for Fig S3C
rvalues = [[],[]]

import scipy.stats
tps = ["Eighth","D1", "D2","D3", "D6"]

rows = 2
cols = 5
fig, ax = plt.subplots(rows,cols, sharex='col', sharey='row')
for row in range(rows):
    for col in range(cols):
        scatter_x = scatter_morphology[tps[col]]
        if row == 0:
            scatter_y = scatter_ryp1_spher[tps[col]]
        elif row == 1:
            scatter_y = scatter_ryp1_hyph[tps[col]]
        else: 
            scatter_y = scatter_ryp1_arth
        ax[row,col].plot(scatter_x, scatter_y, "k,")
        rvalue = scipy.stats.pearsonr(scatter_x,scatter_y)[0]
        ax[row,col].text(-6,14,round(rvalue,5))
        if row <2:
            rvalues[row].append(rvalue)
        ax[row,col].set_ylim(-10,10)
        ax[row,col].set_xlim(-10,10)
        ax[row,col].set_box_aspect(1)
ax[0,0].set_ylabel("S ryp1-reg")
ax[1,0].set_ylabel("H ryp1-reg")
plt.show()
plt.savefig("Arth_ryp1_reg_morphology_correlations_with_pearson.svg")
```

In [53]:

```
#Used to label quadrants in Fig 3C
fig = plt.figure()
xs = scatter_morphology["D3"]
ys = scatter_ryp1_spher["D3"]
plt.scatter(xs,ys, edgecolors='black', s=1)
plt.xlabel("spherule/hyphae")
plt.ylabel("S WT/ryp1")
plt.axhline(1, color="black", linewidth = .5)
plt.axhline(-1, color="black", linewidth = .5)
plt.axvline(1, color="black", linewidth = .5)
plt.axvline(-1, color="black", linewidth = .5)
plt.text(-0.5, 9.5, "632")
plt.text(9.5, -0.5, "495")
plt.text(-9.5,-0.5, "533")
plt.text(-0.5,-9.5, "468")
plt.text(9.5,9.5, "700")
plt.text(-9.5,-9.5,"591")
plt.text(9.5,-9.5, "28")
plt.text(-9.5,9.5,"44")
plt.show()
plt.savefig("D3_scatter_labels.png")
```

In [54]:

```
#Basis for Fig 3C
fig = plt.figure()
xs = scatter_morphology["D3"]
ys = scatter_ryp1_spher["D3"]
plt.scatter(xs,ys, edgecolors='black', s=1)
plt.xlabel("spherule/hyphae")
plt.ylabel("S WT/ryp1")
plt.axhline(1, color="black", linewidth = .5)
plt.axhline(-1, color="black", linewidth = .5)
plt.axvline(1, color="black", linewidth = .5)
plt.axvline(-1, color="black", linewidth = .5)
fig.axes[0].set_box_aspect(1)
plt.show()
plt.savefig("D3_scatter.png")
```

In [55]:

```
#Fig S3D
xvalues = [0.333333,1,2,3,6]
fig = plt.figure(figsize=[2,4])
plt.xticks([1,2,3,6])
plt.plot(xvalues,rvalues[0], marker='o', color='gold',linestyle='-', markeredgecolor='k')
plt.plot(xvalues,rvalues[1], marker='o', color='darkblue',linestyle='-', markeredgecolor='k')
plt.show()
plt.savefig("Correlation_tc_rvalues.eps")
```

# Looking at transcription factors and overlap with Ryp1-dependence and spherule-enriched¶

Analysis described in the text section on transcription factors

In [56]:

```
txn_factors = CdtFile.fromCdt("../../Cocci_TFs.cdt")

txn_factors_cpsilv3 = []
for tf_id in txn_factors.uids:
    row = txn_factors.GetUid(tf_id)
    txn_factors_cpsilv3.append(row.extra[1])
```

In [57]:

```
txn_factors_set = set(txn_factors_cpsilv3)
ryp1_spherule_set = set(limma1_ryp1dep_spherule.uids)
ryp1_myc_set = set(limma1_ryp1dep_myc.uids)
```

In [58]:

```
print(len(txn_factors_set.intersection(ryp1_spherule_set)))
print(txn_factors_set.intersection(ryp1_spherule_set))
print(len(txn_factors_set.intersection(ryp1_myc_set)))
print(txn_factors_set.intersection(ryp1_myc_set))
```

```
17
{'D8B26_005519', 'D8B26_007097', 'D8B26_007094', 'D8B26_000961', 'D8B26_005176', 'D8B26_000722', 'D8B26_002774', 'D8B26_000901', 'D8B26_008426', 'D8B26_003963', 'D8B26_001218', 'D8B26_007356', 'D8B26_000204', 'D8B26_004558', 'D8B26_001067', 'D8B26_007762', 'D8B26_005734'}
4
{'D8B26_000722', 'D8B26_005519', 'D8B26_007356', 'D8B26_005734'}
```

In [ ]:

```

```
